# Supplementary material for: Assessing preferences for HIV pre-exposure prophylaxis (PrEP) delivery services via online pharmacies in Kenya: protocol for a discrete choice experiment
Source: BMJ Open. 2023 Apr 3;13(4):e069195. doi: 10.1136/bmjopen-2022-069195 (PMC10083853; doi:10.1136/bmjopen-2022-069195)
Supplement: Supplementary data [file bmjopen-2022-069195supp001.pdf]

Supplement 1: Cognitive Interview Guide

Online-pharmacy for the delivery of pre-exposure prophylaxis (PrEP) medicines

Discrete Choice Experiment

Cognitive Interview

Introduction

Thank you for taking the time to speak with me. My name is ______. We would like to speak to you about pre-exposure prophylaxis or PrEP. PrEP is a medicine you can take to reduce your risk of getting HIV. We have asked you to participate in this exercise because we are interested in learning how best to delivery PrEP using an online pharmacy. This means a pharmacy will deliver PrEP medication to clients using a courier, so clients do not need to travel to a pharmacy. The aim of this session is to ask you about pictures that are related to PrEP delivery and how they can be improved for our survey instrument. We will also ask you for feedback about other section of our survey to make sure they are clear. This interview should take about 60 minutes. If it's OK with you, I would like to record the interview, so I don't miss any important details in your answers. Feel free to let me know if you need a break at any time. You can also stop the interview if you do not want to continue. Before we begin do you have any questions? Can I start recording?

[Prompt: Start recording]

Part 1. Presentation of Attributes and Levels

Online PrEP delivery includes different services, including an assessment to determine if you are at HIV risk, HIV testing to confirm you are HIV-negative, and talking to a medical provider to make sure PrEP is safe for you.

We will describe the characteristics of obtaining PrEP through online pharmacy in the order you would experience them.

Part 1, a.

PrEP Eligibility Assessment [No need to read the header out loud]

Please tell me how you would interpret these images. [Show the images. Alternative text: What do these images mean to you?]

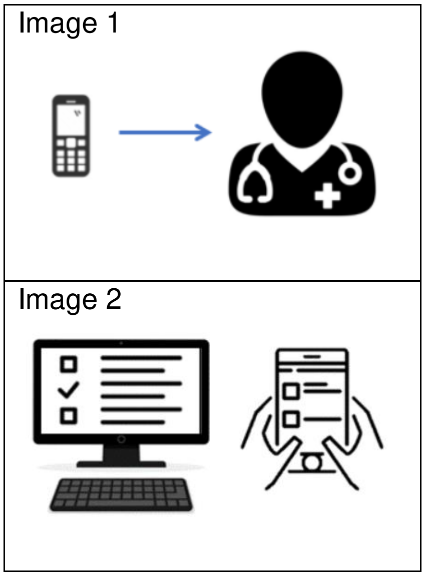

Now I'm going to read you the description and possible ways to have a PrEP Eligibility assessment

PrEP Eligibility Assessment
First, you would complete an **eligibility assessment** to see if PrEP is a good fit for you. This is done by answering questions about your sexual behavior and partners. You can do this in **two ways:** One way is with a provider asking you these questions over the phone or WhatsApp.

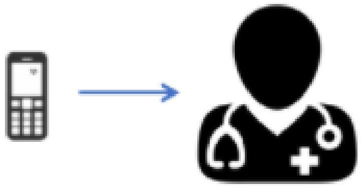

The second way is by answering these questions by yourself on your computer or phone. You will have a phone number to call in case of questions.

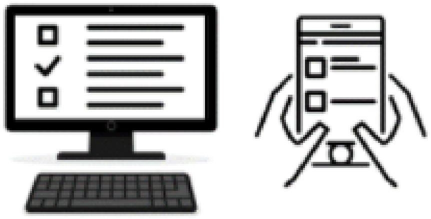

Questions:

- Is the description of "PrEP eligibility Assessment" clear to you? [If necessary, read the description again]
    - Is there any other information we should include to make the description clearer?
- Do the images represent the descriptions well? How could we modify the image to make it clearer?
    - Do you think another person would understand the meaning of this image?
- Are there other ways to assess PrEP eligibility that you think we should include?

Part 1, b.

HIV Test Type [No need to read the header out loud]

Please tell me what you think these images mean. [Show the images]

Image 1

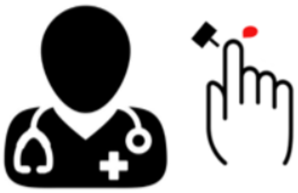

Image 2

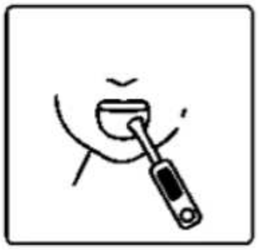

Image 3

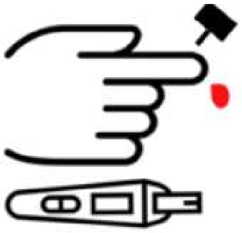

BMJ Publishing Group Limited (BMJ) disclaims all liability and responsibility arising from any reliance placed on this supplemental material which has been supplied by the author(s)

Now I'm going to read you the description and possible ways to have an HIV Test

HIV Test Type

Next you would take an HIV test to make sure you are HIV-negative before starting PrEP. You can test for HIV in one of 3 ways.

One way is to have a healthcare provider meet you at a setting of your choice to conduct HIV test by taking blood from your finger.

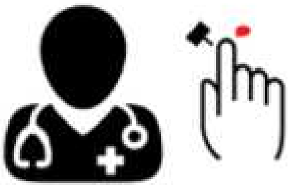

The second way is testing yourself using an HIV self-test kit that uses oral fluid that you collect by swabbing your cheek and gums.

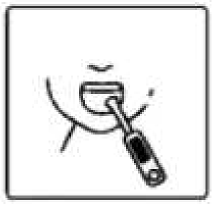

And the third way is testing yourself using a HIV self-test kit that uses blood that you collect from your finger (through a finger prick). Both self-testing kits would be delivered to a setting of your choice and would show a test result after about 20 minutes.

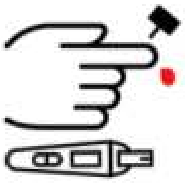

Questions:

- Is the description of "HIV Test Type" clear to you?
    - Is there any other information we should include to make the description clearer?
- Do the images represent the descriptions well? How could we modify the image to make it clearer?
    - Do you think another person would understand the meaning of this image?
- Are there other HIV Test Types that you think we should include?

Is any of the following images a better match for the descriptions I showed you before?

[Repeat the description of the ways to obtain HIV Test if necessary]

<table>
<tr><th>New</th><th>Original</th></tr>
<tr><td>Image 1
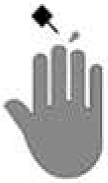
[Self-testing]</td><td rowspan="2">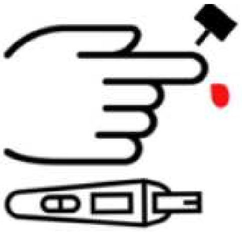</td></tr>
<tr><td>Image 2
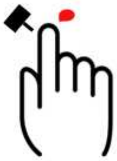
[Self-testing]</td></tr>
<tr><td>Image 3
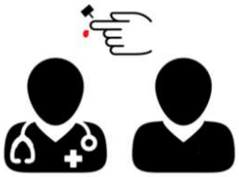
[Blood -based testing]</td><td rowspan="2">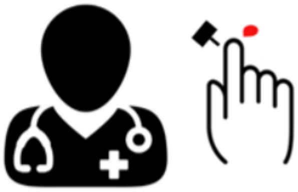</td></tr>
<tr><td>Image 3
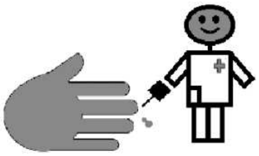
[Blood -based testing]</td></tr>
</table>

Part 1, c.

Clinical Consultation [No need to read the header out loud]

Please tell me how you would interpret these images. [Show the images]

Image 1 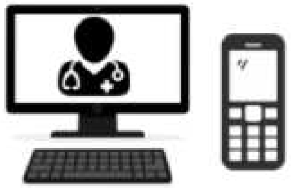
Image 2 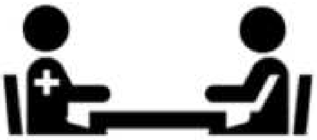

Now I'm going to read you the description and different possible ways to have a Clinical Consultation

Clinical Consultation

You would then have a clinical consultation with a medical provider to make sure PrEP is safe for you to use. This can be done over the phone or a video chat.

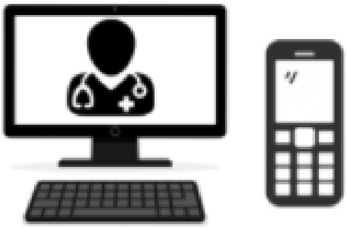

Another option is to see a provider in person at a setting of your choice.

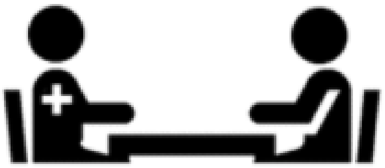

Questions:

- Is the description of "Clinical Consultation" clear to you?
    - Is there any other information we should include to make the description clearer?
- Do the images represent the descriptions well? How could we modify the image to make it clearer?
    - Do you think another person would understand the meaning of this image?
- Are there other ways to provide Clinical consultation that you think we should include?

Is any of the following images a better match for the descriptions I showed you before? [Repeat the description of the ways to provide Clinical Consultation if necessary]

New	Original
Image 1 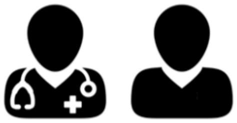 [In-person consultation]	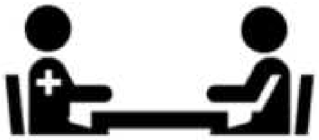
Image 2 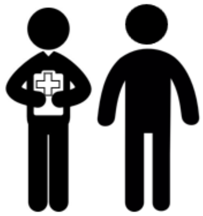 [In-person consultation]	

Part 1, d.

User Support Options [No need to read the header out loud]

Please tell me how you would interpret these images. [Show the images]

Image 1 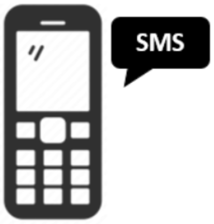
Image 2 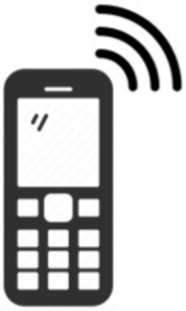
Image 3 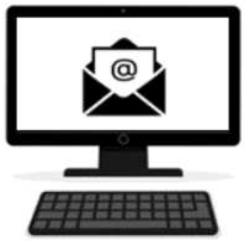

Now I'm going to read you the description and different ways to get User Support

User Support Options

In case you have any questions about PrEP, there are **three different options** for you to discuss your questions with a healthcare provider. The first is using SMS to talk to a provider.

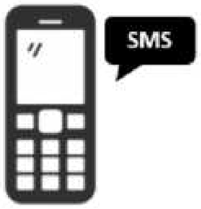

The second option is talking to a provider over the phone, using either voice or video call.

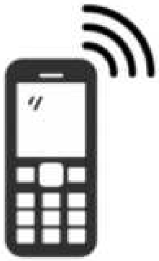

The third is using email to talk to a provider.

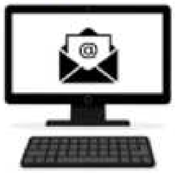

Questions:

- Is the description of "User Support Options" clear to you?
    - Is there any other information we should include to make the description clearer?
- Do the images represent the descriptions well? How could we modify the image to make it clearer?
    - Do you think another person would understand the meaning of this image?
- Are there other ways to provide user support that you think we should include?

Is any of the following images a better match for the descriptions I showed you before? [Repeat the description of the ways to provide User Support if necessary]

**New**	**Original**
Image 1 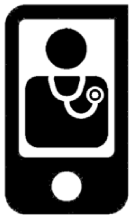 [Video call]	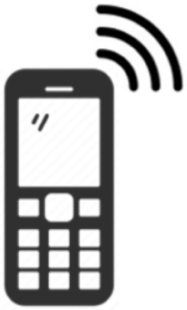
Image 2 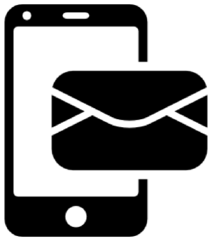 [Email]	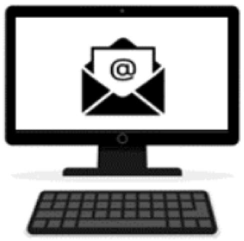

Part 1, e. Summary Table

**Characteristics**<br>*Description*	**Option 1**	**Option 2**	**Option 3**
**PrEP eligibility assessment**<br>*Method for conducting client eligibility assessment for PrEP*	*Online self-assessment **on your computer or phone** using screening questions (phone number in case of questions)*<br>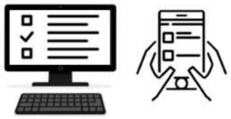	*Guided assessment with a remote clinical provider (via a phone call or WhatsApp)*<br>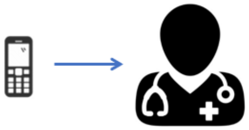	
**HIV test type**<br>*Type of HIV test delivered for PrEP initiation*	*Oral-fluid HIV self-test (at setting of your choice)*<br>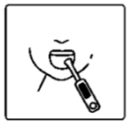	*Blood-based HIV self-test (at setting of your choice)*<br>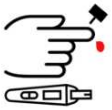	*Provider-administered blood-based rapid diagnostic test (at setting of your choice)*<br>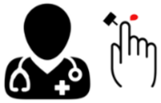
**Clinical consultation for prescribing PrEP**<br>*Clinical consultation needed to prescribe PrEP*	*Remote clinical consultation with provider (via a phone call or video chat)*<br>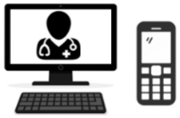	*In-person clinical consultation with provider after completing HIV testing (at a setting of your choice)*<br>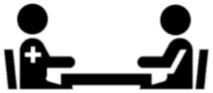	
**User support options for PrEP**<br>*Method for discussing your questions for PrEP with a healthcare provider*	*SMS*<br>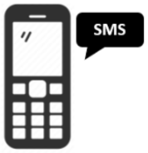	*Phone/video call*<br>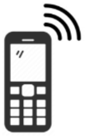	*Email*<br>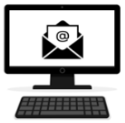

Questions:

We want to make sure we have included all the characteristics of online PrEP delivery that would be important to you

- Are any of the characteristics of PrEP delivery that we showed you today not important or don't add relevant information?
- Is there anything else about an online PrEP delivery service that would be important to you that we did not ask you about?

Part 2. DCE Choice Task Example

First Take:

I will now ask you about how you would like to get PrEP from an online pharmacy. Please remember there are no wrong answers, we want to know your preferences. When answering each scenario, imagine that you are planning to get PrEP using an online pharmacy.

When making your choice, please consider ONLY the characteristics shown.

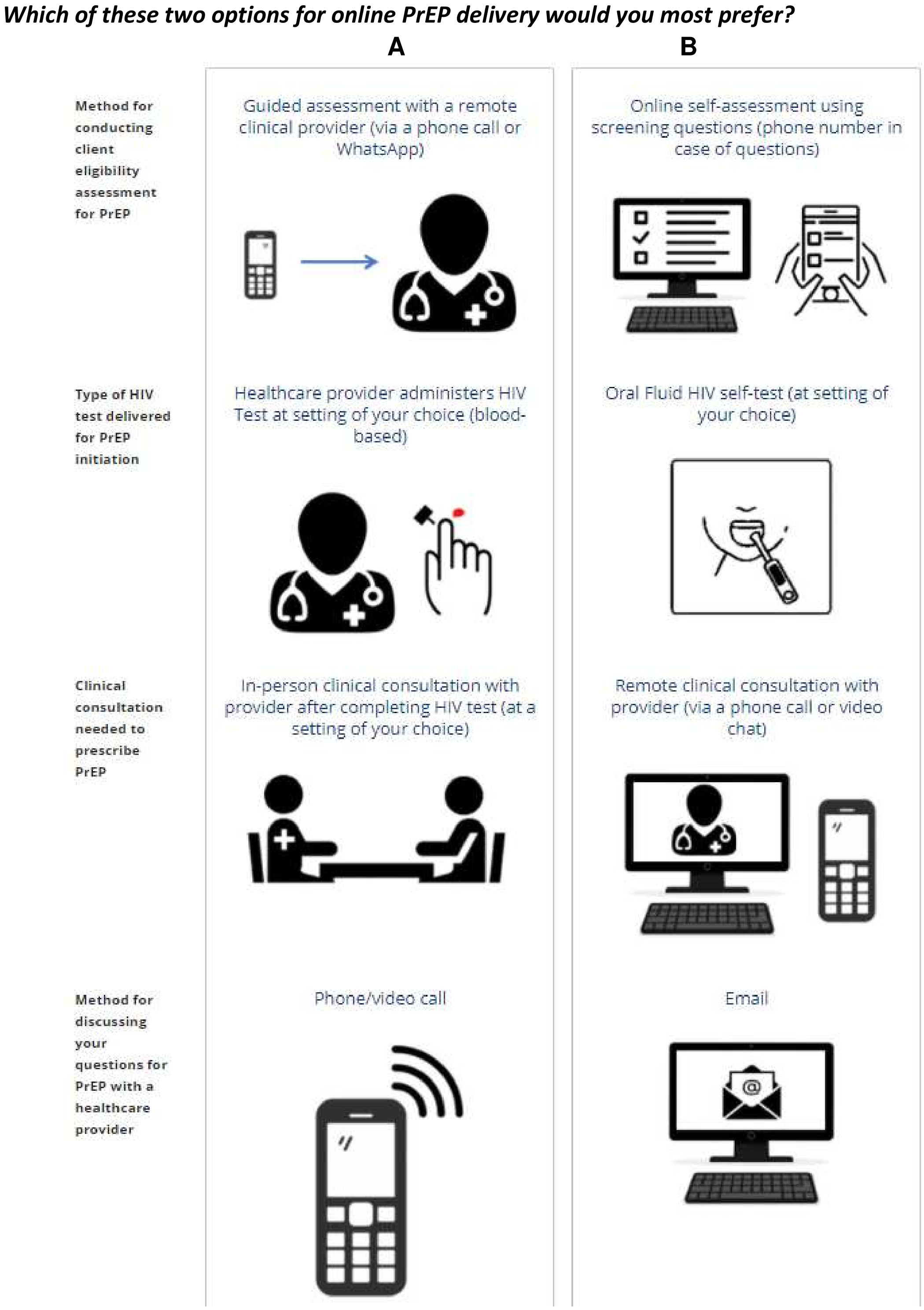
Which of these two options for online PrEP delivery would you most prefer?
A
B
Method for conducting client eligibility assessment for PrEP
Guided assessment with a remote clinical provider (via a phone call or WhatsApp)
Online self-assessment using screening questions (phone number in case of questions)
Type of HIV test delivered for PrEP initiation
Healthcare provider administers HIV Test at setting of your choice (blood-based)
Oral Fluid HIV self-test (at setting of your choice)
Clinical consultation needed to prescribe PrEP
In-person clinical consultation with provider after completing HIV test (at a setting of your choice)
Remote clinical consultation with provider (via a phone call or video chat)
Method for discussing your questions for PrEP with a healthcare provider
Phone/video call
Email

Questions:

- How hard did you find this exercise?
- How could we modify the exercise to make it easier?
- Do you think another person would understand this exercise?
- Is there anything in the question for the exercise that we should modify to make it clearer? [If necessary, read the question again "Which of these two options for online PrEP delivery would you most prefer?"]
- When I said, "When making your choice, please consider ONLY the characteristics I presented before.", what does that mean to you? [The objective of that sentence is to ignore any other characteristics of online pharmacy (e.g., the brand of the drug or the company providing the service) that are not included in the table. Check if the participant understood that.]
- Can you tell me how you made your decision? [Check if the participant is paying attention to all characteristics, if he doesn't mention one, ask about it. For example: I noticed you didn't mention "Clinical consultation", did you consider the option for that characteristic?"]

Second take:

I will now ask you about how you would like to get PrEP from an online pharmacy. Please remember there are no wrong answers, we want to know your preferences. When answering each scenario, imagine that you are planning to get PrEP using an online pharmacy. I will also ask you if you would choose to get PrEP using the option you selected. If you would not choose to get PrEP if only these two options were available, you can answer no to this question.

When making your choice, please consider ONLY the characteristics shown

Which of these two options for online PrEP delivery would you most prefer?

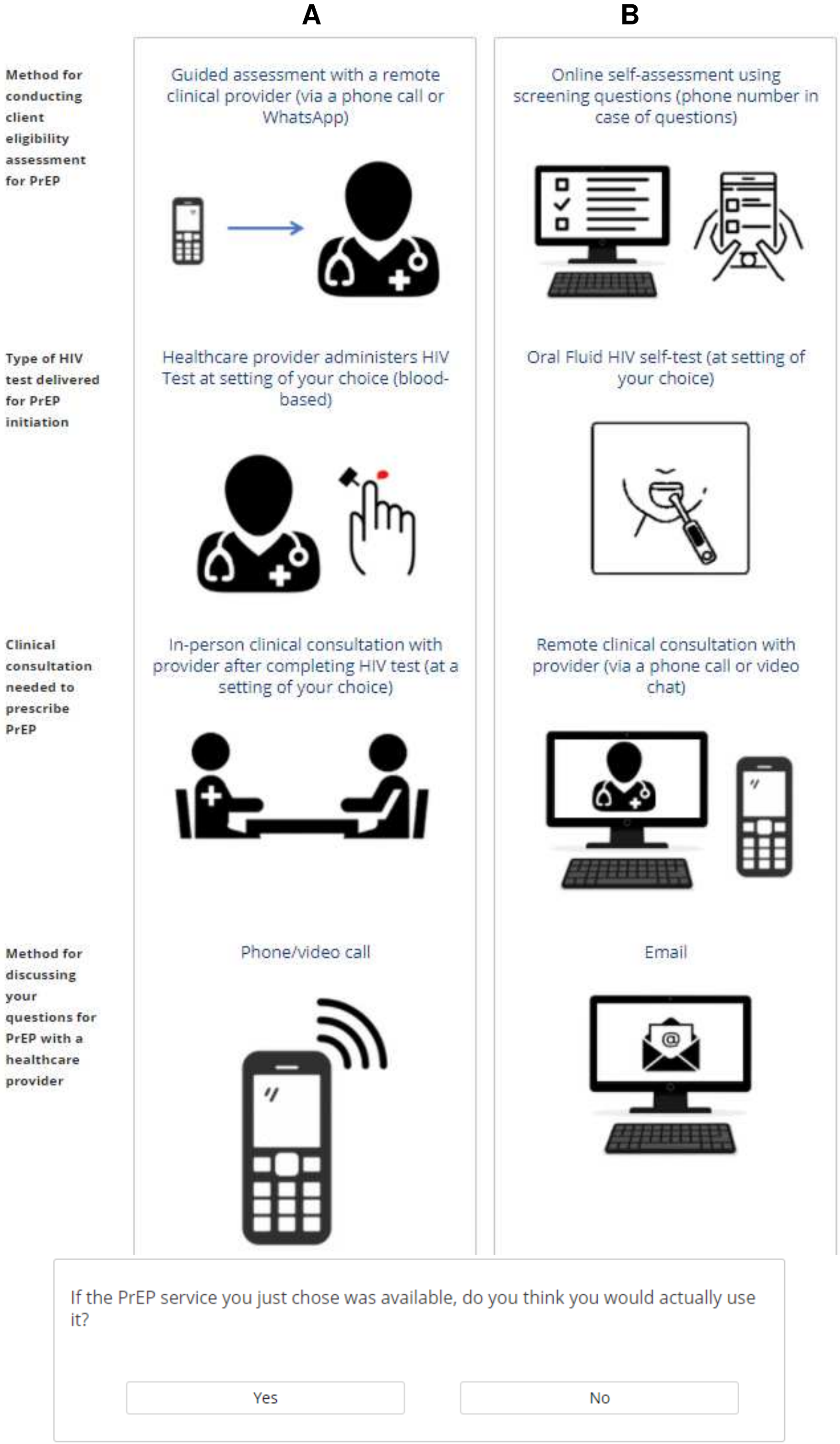

Questions:

- How different was this time choosing an alternative compared to the first time? [Now the participant has the option to say if they would choose to get PrEP based on their previous selection or not]
- Was does the question "Would you choose to get PrEP using this service if it were available?" mean in your own words? What do you think is asking from you?
    - Our goal in asking this question is to understand if you would really choose to get this service if it were available to you. Do you think the question is clearly asking that?
    - If not, how can we make this question clearer? [If participant said they don't know, that's fine, move to the next section]
- How many of these tasks do you think you could answer before getting tired and/or bored?

Part c. Willingness to Pay Questions

The next set of questions will ask about how much you would like to pay for different parts of online PrEP delivery. For each part, please provide the lowest price, the maximum price and the ideal price you are willing to pay. For the lowest price, please tell us the lowest amount you think the service should cost because otherwise you would be concerned about its quality, and you would not purchase the service. In answering these questions, please consider your usual expenses. Remember that there are no right or wrong answers. We're interested in your preference. You can write zero if you feel that most accurately reflects your preference.

For a blood-based HIV self-test, delivered via a courier to a location of your choice (one-time cost):

What is the lowest price you are willing to pay? ______ KSH

What is the highest price you are willing to pay? ______ KSH

What price do you consider ideal? ______ KSH

For an oral HIV self-test, delivered via a courier to a location of your choice (one-time cost):

What is the lowest price you are willing to pay? ______ KSH

What is the highest price you are willing to pay? ______ KSH

What price do you consider ideal? ______ KSH

For remote/online clinical consultation to obtain a prescription for PrEP based on your HIV self-test results (one-time cost):

What is the lowest price you are willing to pay? ______ KSH

What is the highest price you are willing to pay? ______ KSH

What price do you consider ideal? ______ KSH

For delivery of PrEP medicines (delivery fee plus 1 month supply of medicines):

What is the lowest price you are willing to pay? ______ KSH

What is the highest price you are willing to pay? ______ KSH

What price do you consider ideal? ______ KSH

Questions:

- Overall, is it clear what the objective of the questions is? Would you mind explaining it to me in your own words?
- Please select one of the questions and give your answers. Would you explain me how did you select the amounts?
  [Pay attention to the order in which the participant finds the amounts. Which one comes first, the ideal, the lowest, or the highest?]
  - In this process, did you consider your usual expenses on medicines or healthcare?
  - What other expenses did you consider, if any?
  - Did you consider the cost options presented in the previous section?
- Is the instruction "For the lowest price, please tell us the lowest amount you think the service should cost because otherwise you would be concerned about its quality, and you would not purchase the service." clear enough? Would you tell me in your own words what does it mean?
  - Would you be concerned about the quality of a product that is free? Why or why not?
- Do you think that all questions are unique or some of them ask for the same information as others?

End of the interview

Do you have any questions or final thoughts you would like to share?

Thank you very much for your time and dedication in giving me your answers, I'm sure your information will help us create a better survey.

[Stop the recording]
