# Supplementary material for: Assessing preferences for HIV pre-exposure prophylaxis (PrEP) delivery services via online pharmacies in Kenya: protocol for a discrete choice experiment
Source: BMJ Open. 2023 Apr 3;13(4):e069195. doi: 10.1136/bmjopen-2022-069195 (PMC10083853; doi:10.1136/bmjopen-2022-069195)
Supplement: Supplementary data [file bmjopen-2022-069195supp002.pdf]

BMJ Publishing Group Limited (BMJ) disclaims all liability and responsibility arising from any reliance placed on this supplemental material which has been supplied by the author(s)

Supplement 2: Training Materials

EPharmacy PrEP: Pilot Study Discrete Choice Experiment Training

24th-25th January 2022

BMJ Publishing Group Limited (BMJ) disclaims all liability and responsibility arising from any reliance placed on this supplemental material which has been supplied by the author(s)

Introductions

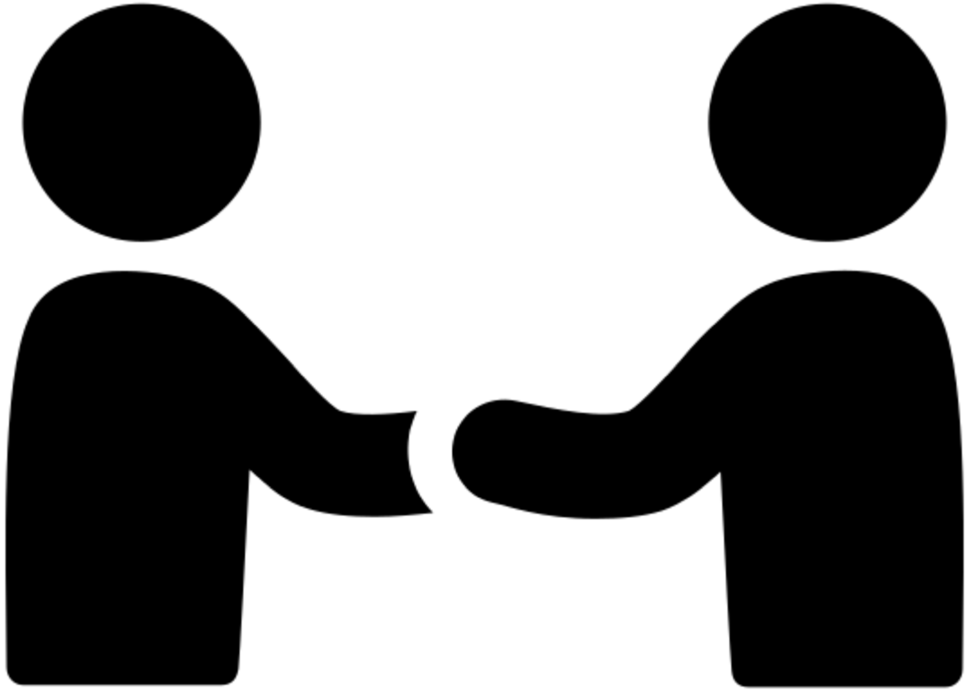

Overview of this session

- PrEP
- PrEP delivery strategies
- ePharmacy PrEP delivery
- Discrete Choice Experiments

BMJ Publishing Group Limited (BMJ) disclaims all liability and responsibility arising from any reliance placed on this supplemental material which has been supplied by the author(s)

What is PrEP?

- PrEP is safe in pregnancy and for breastfeeding mothers
- It takes approximately **SEVEN** days after starting treatment for PrEP to be optimally effective.
- With poor adherence effectiveness becomes lower

Recommended medication for PrEP use

PREFERRED	ALTERNATIVE
Tenofovir (TDF) + Emtricitabine (FTC)<br>300mg 200mg	Tenofovir (TDF) 300 mg
	Tenofovir (TDF) + Lamivudine (3TC)<br>300mg 300mg

Does PrEP have side effects?

PrEP side effects

No major safety issues have been observed with use of PrEP.

About 1 in 10 people who use PrEP experience **minor**, **temporary** side effects, such as:

- Headache
- Weight loss
- Nausea
- Vomiting
- Abdominal discomfort

If side effects occur, they typically **start in the first few days or weeks** of PrEP use and **last a few days**—almost always less than 1 month.

References: AVAC (Nov. 2020); Tetteh et al. (2017)

BMJ Publishing Group Limited (BMJ) disclaims all liability and responsibility arising from any reliance placed on this supplemental material which has been supplied by the author(s)

What PrEP does and doesn’t prevent

PrEP reduces risk of getting HIV by at least 90%

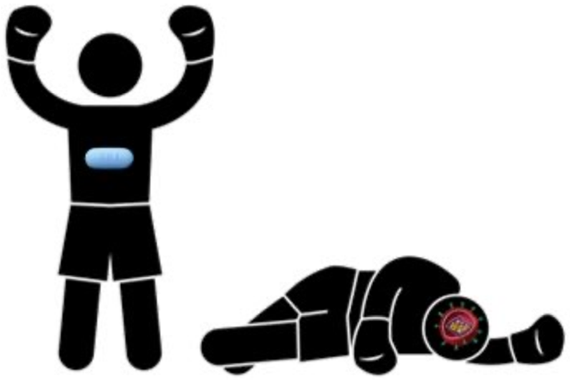

PrEP does **not** prevent sexually transmitted infections (STIs) or pregnancy.

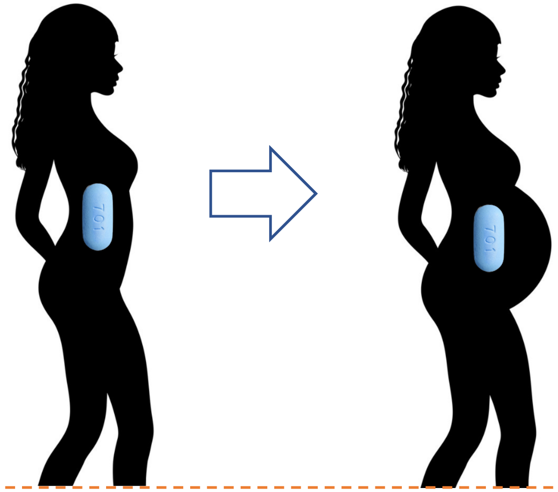

Prospective PrEP users should be advised that if they wish to protect themselves against STIs and/or pregnancy, they should **use PrEP in combination with other methods**, like condoms and contraception.

BMJ Publishing Group Limited (BMJ) disclaims all liability and responsibility arising from any reliance placed on this supplemental material which has been supplied by the author(s)

Aside from protecting against HIV, does PrEP have any other benefits?

Other PrEP benefits

Additional benefits of PrEP:

- Decreased anxiety or fear of contracting HIV
- Increased sense of empowerment and control over HIV
- Increased sense of pleasure and intimacy during sex
- Increased communication and trust with sex partners

Benefits specific to serodiscordant couples:

- Increased ability to cope with and accept serodiscordance
  (e.g., sense of solidarity taking daily medication)
- Ability to safely conceive a child

Sources: Gilmore et al. 2014; Ware et al. 2012; Ware et al. 2014

BMJ Publishing Group Limited (BMJ) disclaims all liability and responsibility arising from any reliance placed on this supplemental material which has been supplied by the author(s)

PrEP eligibility criteria

Per Kenya national guidelines

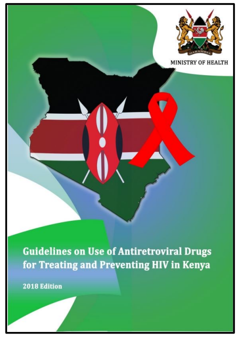

- Age 16+
- Confirmed HIV-negative
- Substantial, ongoing risk of HIV infection
- No signs of acute HIV infection in past month
- No contraindications to PrEP medications
- Willing to use PrEP as prescribed

*Specifically, HIV vaccine or HIV prevention studies

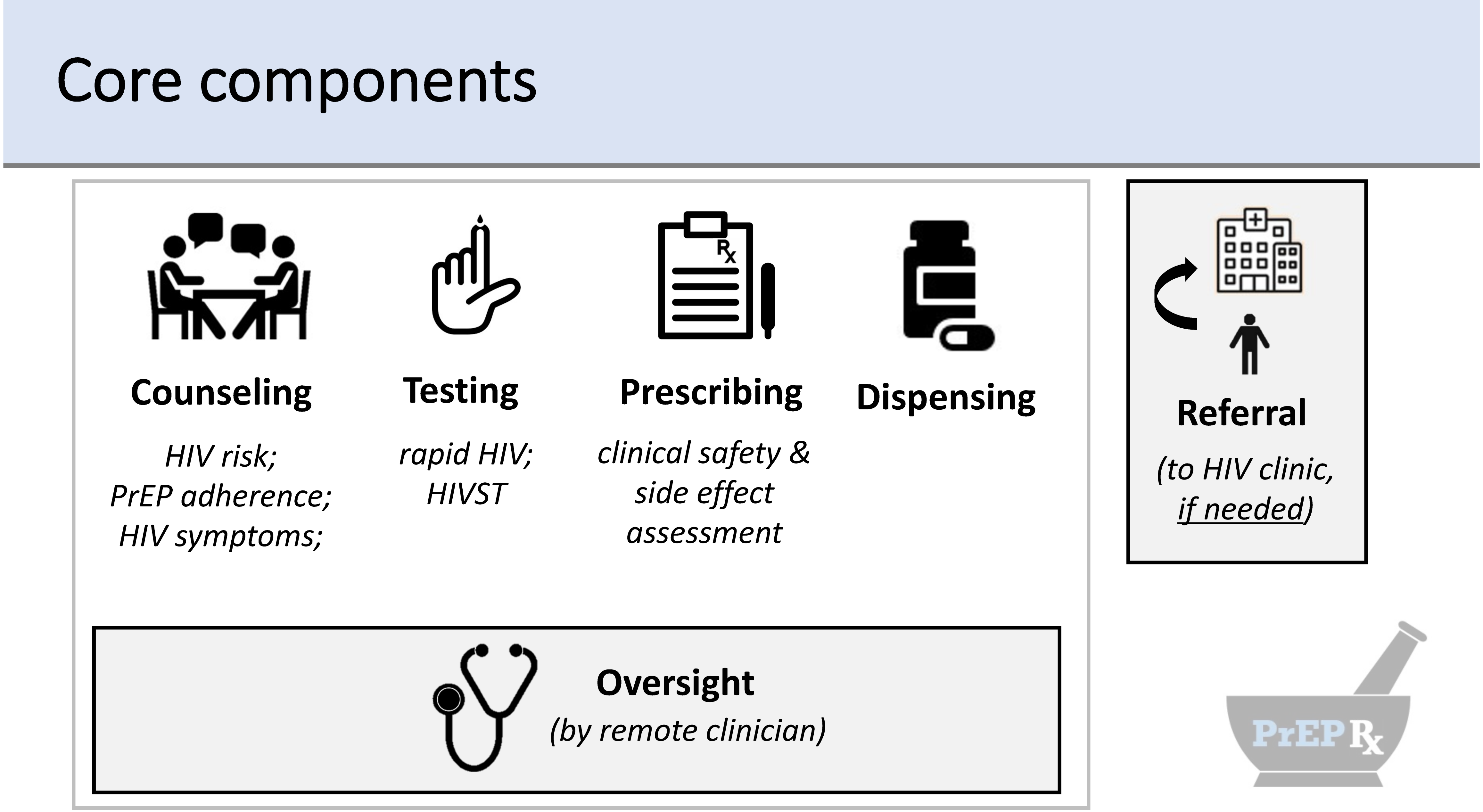
Core components
Counseling
HIV risk;
PrEP adherence;
HIV symptoms;
Testing
rapid HIV;
HIVST
Prescribing
clinical safety &
side effect
assessment
Dispensing
Referral
(to HIV clinic,
if needed)
Oversight
(by remote clinician)
PrEP Rx

PrEP use over time using retail pharmacies: Pilot study

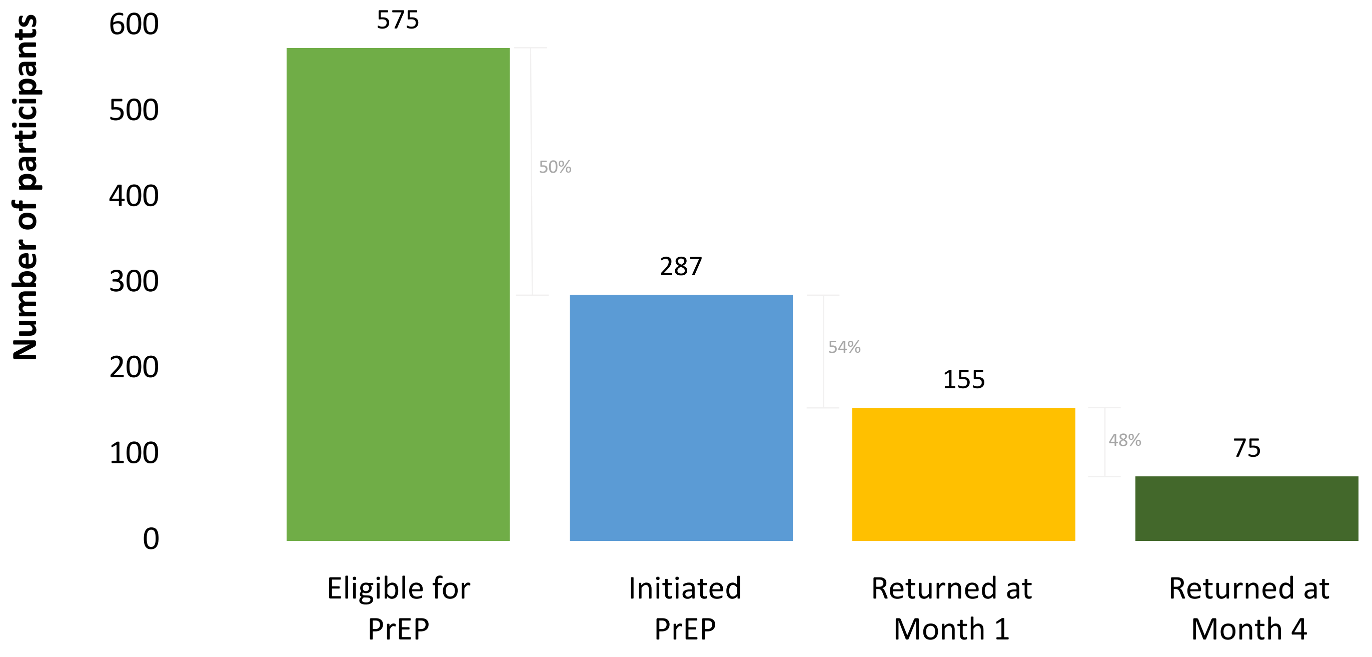

BMJ Publishing Group Limited (BMJ) disclaims all liability and responsibility arising from any reliance placed on this supplemental material which has been supplied by the author(s)

Challenges with delivering PrEP at retail pharmacies

1 PrEP services too **expensive** for some clients.

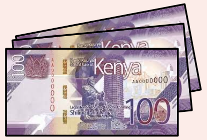

2 HIV risk assessment **time-consuming** & some clients **uncomfortable** doing it out loud.

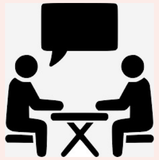

3 Some prospective clients **uncomfortable** testing for HIV at pharmacy, especially if unsure of status.

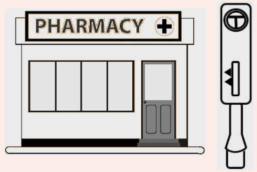

Can PrEP be delivered through E-pharmacies?

Possible benefits

- Convenient: Clients can get PrEP delivered to their home without having to go to a clinic or pharmacy
- Greater confidentiality (decreased stigma)

PrEP Rx

Steps for getting PrEP from E-pharmacy

1) HIV risk screening
2) HIV testing
3) Clinical consultation (medical assessment)
4) PrEP drugs delivered via courier
5) Ongoing user support (clients can talk to a provider to ask questions about their PrEP)

BMJ Publishing Group Limited (BMJ) disclaims all liability and responsibility arising from any reliance placed on this supplemental material which has been supplied by the author(s)

Step 1: HIV Risk assessment

Assessing HIV risk

Example questions from the RAST

In the past 6 months, have you had sex without a condom with anyone?<br><br>*Select "yes" even if this only happened once in the past 6 months.*	☐ Yes	☐ No	☐ Unsure

Assessing HIV risk

Example questions from the RAST

In the past 6 months, have you had sex without a condom with anyone?<br><br>*Select "yes" even if this only happened once in the past 6 months.*	☐ Yes	☐ No	☐ Unsure
In the past 6 months, have you been diagnosed with or treated for a sexually transmitted infection (STI)?<br><br>*Examples of sexually transmitted infections include chlamydia and gonorrhea.*	☐ Yes	☐ No	☐ Unsure

Assessing HIV risk

Example questions from the RAST

In the past 6 months, have you had sex without a condom with anyone?<br><br>*Select "yes" even if this only happened once in the past 6 months.*	☐ Yes	☐ No	☐ Unsure
In the past 6 months, have you been diagnosed with or treated for a sexually transmitted infection (STI)?<br><br>*Examples of sexually transmitted infections include chlamydia and gonorrhea.*	☐ Yes	☐ No	☐ Unsure
Do you have any sex partners who are HIV-positive and:<br>• are not currently taking antiretroviral therapy (ART),<br>• have been taking antiretroviral therapy (ART) for less than 6 months,<br>• have been missing doses of their antiretroviral therapy (ART),<br>• have a detectable HIV viral load, or<br>• the two of you are trying to get pregnant?<br><br>*Select "yes" if any of the above statements are true about any of your sex partners who are HIV-positive.*	☐ Yes	☐ No	☐ Unsure

Assessing HIV risk

Example questions from the RAST

In the past 6 months, have you had sex without a condom with anyone?<br><br>*Select "yes" even if this only happened once in the past 6 months.*	☐ Yes	☐ No	☐ Unsure
In the past 6 months, have you been diagnosed with or treated for a sexually transmitted infection (STI)?<br><br>*Examples of sexually transmitted infections include chlamydia and gonorrhea.*	☐ Yes	☐ No	☐ Unsure
Do you have any sex partners who are HIV-positive and:<br>• are not currently taking antiretroviral therapy (ART),<br>• have been taking antiretroviral therapy (ART) for less than 6 months,<br>• have been missing doses of their antiretroviral therapy (ART),<br>• have a detectable HIV viral load, or<br>• the two of you are trying to get pregnant?<br><br>*Select "yes" if any of the above statements are true about any of your sex partners who are HIV-positive.*	☐ Yes	☐ No	☐ Unsure

If client answers "yes" to any RAST question, s/he is considered to have substantial ongoing risk of HIV.

Step 2: HIV testing

Importance of HIV testing

- PrEP is for HIV **prevention**, not treatment.
- Therefore, PrEP is only for **HIV-negative** individuals.
  - HIV-*positive* individuals should take ART, not PrEP.
  - Starting an HIV-*positive* individual on PrEP could make their HIV virus harder to treat.

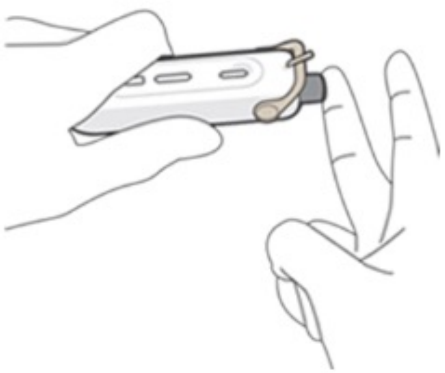

So before starting someone on PrEP,
it is critical to confirm that they are HIV-negative.

PrEP users must also get ongoing
periodic HIV testing to confirm that they are still HIV-negative (usually every 1-3 months.

HIV testing schedule

Kenya national PrEP guidelines

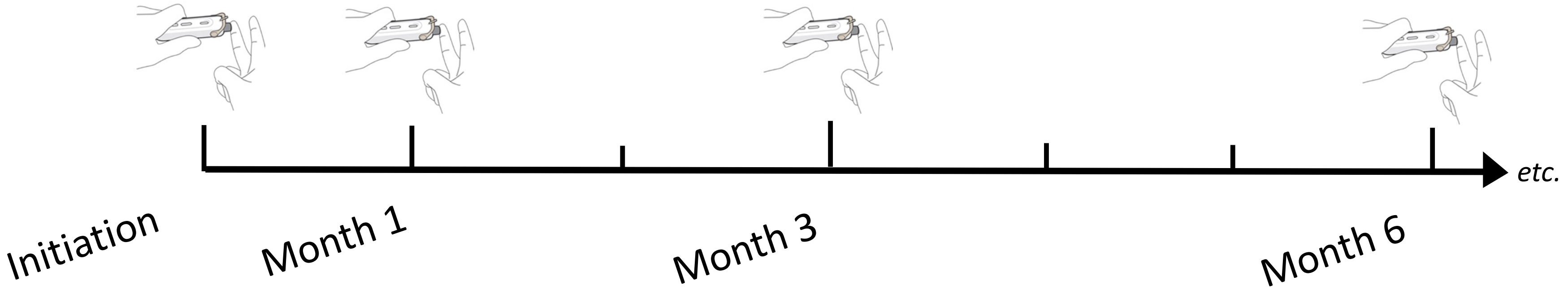

What is HIVST?

A process whereby an individual collects his or her own specimen (oral fluid or blood), performs an HIV rapid diagnostic test and interprets the result, often in a private setting, either alone or with someone she trusts.

Benefits:

- Promotes access to HIV testing services
- Increases autonomy
- Assures confidentiality/privacy
- Empowers
- Convenient

BMJ Publishing Group Limited (BMJ) disclaims all liability and responsibility arising from any reliance placed on this supplemental material which has been supplied by the author(s)

Step 3: Clinical consultation

Medical safety assessment

Signs & symptoms of acute HIV infection?	☐ Yes	☐ No
Current or past liver disease?	☐ Yes	☐ No
Current or past kidney disease?	☐ Yes	☐ No
Diabetic?	☐ Yes	☐ No

Medical safety assessment

Signs & symptoms of acute HIV infection?	☐ Yes	☐ No

Provider asks the client if they have had any high-risk sexual contact **in past month** and subsequently experienced any of the following:

- Fever
- Sore throat
- Muscle or joint pains
- Swollen glands
- Diarrhoea
- Headache

If client answers "yes", provider does not prescribe PrEP. Refers client to clinic.

BMJ Publishing Group Limited (BMJ) disclaims all liability and responsibility arising from any reliance placed on this supplemental material which has been supplied by the author(s)

Medical safety assessment

Current or past liver disease?	☐ Yes	☐ No

Asks if client has ever had problems with their liver, such as hepatitis, cancer, or cirrhosis.

If client answers "yes", provider <u>does not</u> prescribe PrEP. Refers client to clinic.

Medical safety assessment

Current or past kidney disease?	☐ Yes	☐ No

Asks if client has ever had problems with their kidneys, such as acute or chronic kidney injury or kidney failure.

If client answers "yes", provider <u>does not</u> prescribe PrEP. Refers client to clinic.

BMJ Publishing Group Limited (BMJ) disclaims all liability and responsibility arising from any reliance placed on this supplemental material which has been supplied by the author(s)

Medical safety assessment

Diabetic?	☐ Yes	☐ No

If client is diabetic, provider does <u>not</u> start PrEP.

- Diabetes is a risk factor for kidney disease. So diabetic clients must be evaluated for kidney disease before starting PrEP.

Provider refers client to clinic

BMJ Publishing Group Limited (BMJ) disclaims all liability and responsibility arising from any reliance placed on this supplemental material which has been supplied by the author(s)

Step 4: PrEP delivery via courier

BMJ Publishing Group Limited (BMJ) disclaims all liability and responsibility arising from any reliance placed on this supplemental material which has been supplied by the author(s)

PrEP delivery

- Delivered by courier to location of client's choice (e.g. home or other location)
- Discreet packaging

BMJ Publishing Group Limited (BMJ) disclaims all liability and responsibility arising from any reliance placed on this supplemental material which has been supplied by the author(s)

Step 5: Ongoing user support

BMJ Publishing Group Limited (BMJ) disclaims all liability and responsibility arising from any reliance placed on this supplemental material which has been supplied by the author(s)

User support

- Client can ask a provider questions about PrEP use, side effects, etc
- Options for user support
  - Phone calls
  - WhatsApp/SMS
  - Email

BMJ Publishing Group Limited (BMJ) disclaims all liability and responsibility arising from any reliance placed on this supplemental material which has been supplied by the author(s)

Pilot study of E-pharmacy PrEP delivery

Online model: pilot study

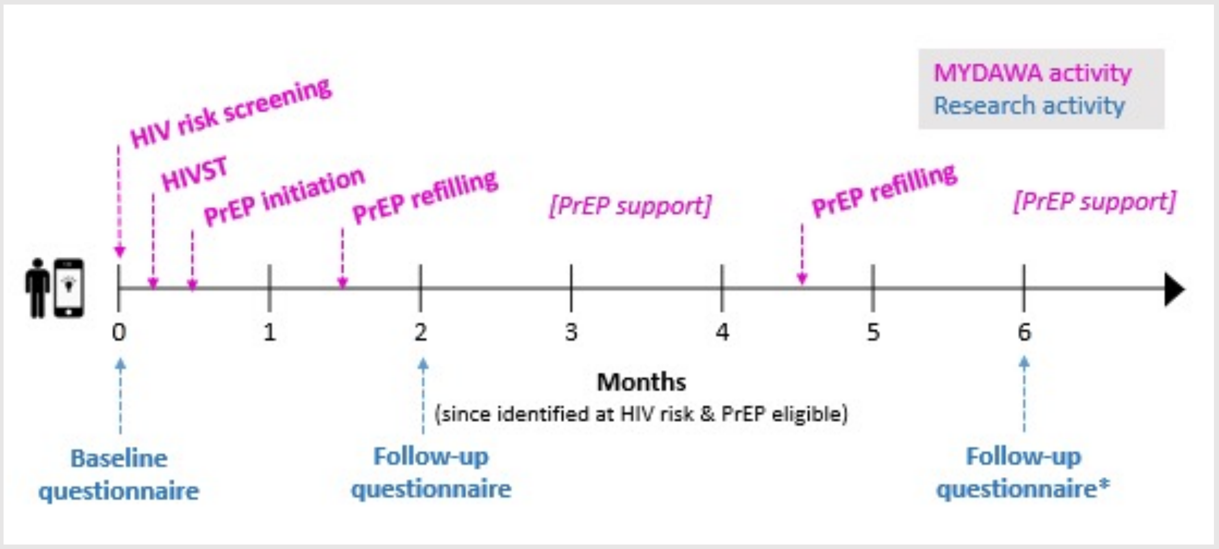

**Client characteristics**	**Demographics**	Month: 0
	**HIV risk profile**	Months: 0 & 2
	**History of PrEP use**	Month: 0
	**Contraceptive use**	Month: 0
**Effectiveness outcomes**	**PrEP initiation** *(primary)*	Month: 2
	**PrEP continuation**	Months: 2 & 6
**Process outcomes**	**Awareness of online PrEP**	Month: 0
	**Modality of HIV risk screening**	Month: 0
	**HIVST uptake**	Month: 2
	**Chatbot/clinical officer use**	N/A
	**HIVST/PrEP delivery setting**	N/A
	**Selection of support reminders**	N/A

What are client preferences for E-Pharmacy PrEP delivery?

- Risk assessment
- Clinical consultation
- Type of HIV test
- User support
- Cost

Understanding client preferences can help increase PrEP coverage among those at risk of HIV

BMJ Publishing Group Limited (BMJ) disclaims all liability and responsibility arising from any reliance placed on this supplemental material which has been supplied by the author(s)

Discrete Choice Experiments (DCEs)

- Quantitative method used to understand participants preferences for a service without directly asking them to state their options.
- Participants given a series of alternative hypothetical scenarios with variables or “attributes”, each with variations or “levels” and asked to choose their preferred scenario.
- Surveys usually contain 5-10 scenarios.
- Increasingly used in healthcare settings
- Especially useful for new interventions that are not currently available

Why use a DCE instead of a qualitative interview?

- Qualitative interviews to understand preferences for straightforward decisions but DCEs are useful for more complex interventions. DCEs provide quantitative estimate of preferences, estimate trade-offs between preferences

Analysis

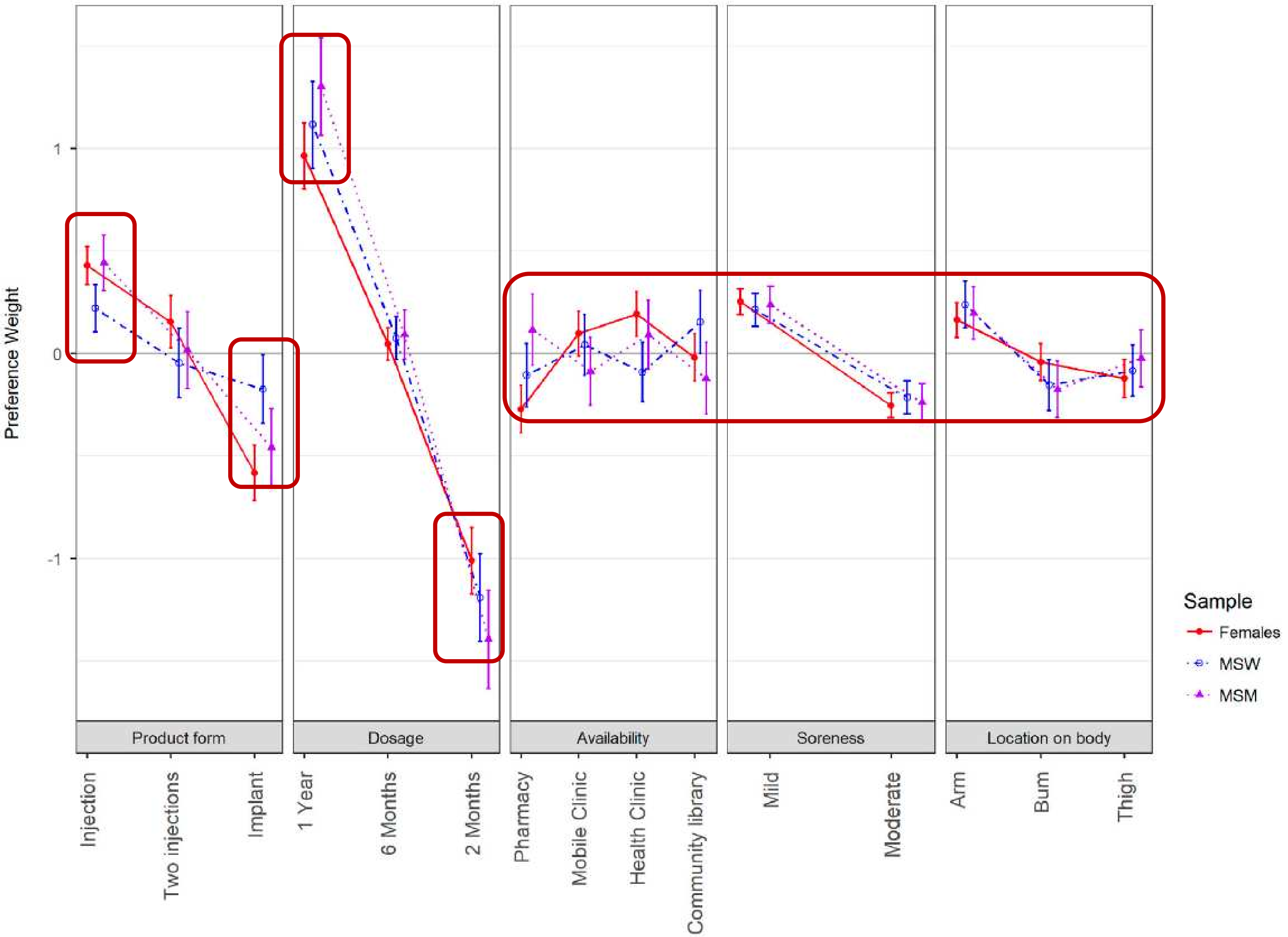

Can quantify preferences to determine most important characteristics
Can stratify by participant type

Using DCEs to quantify trade-offs

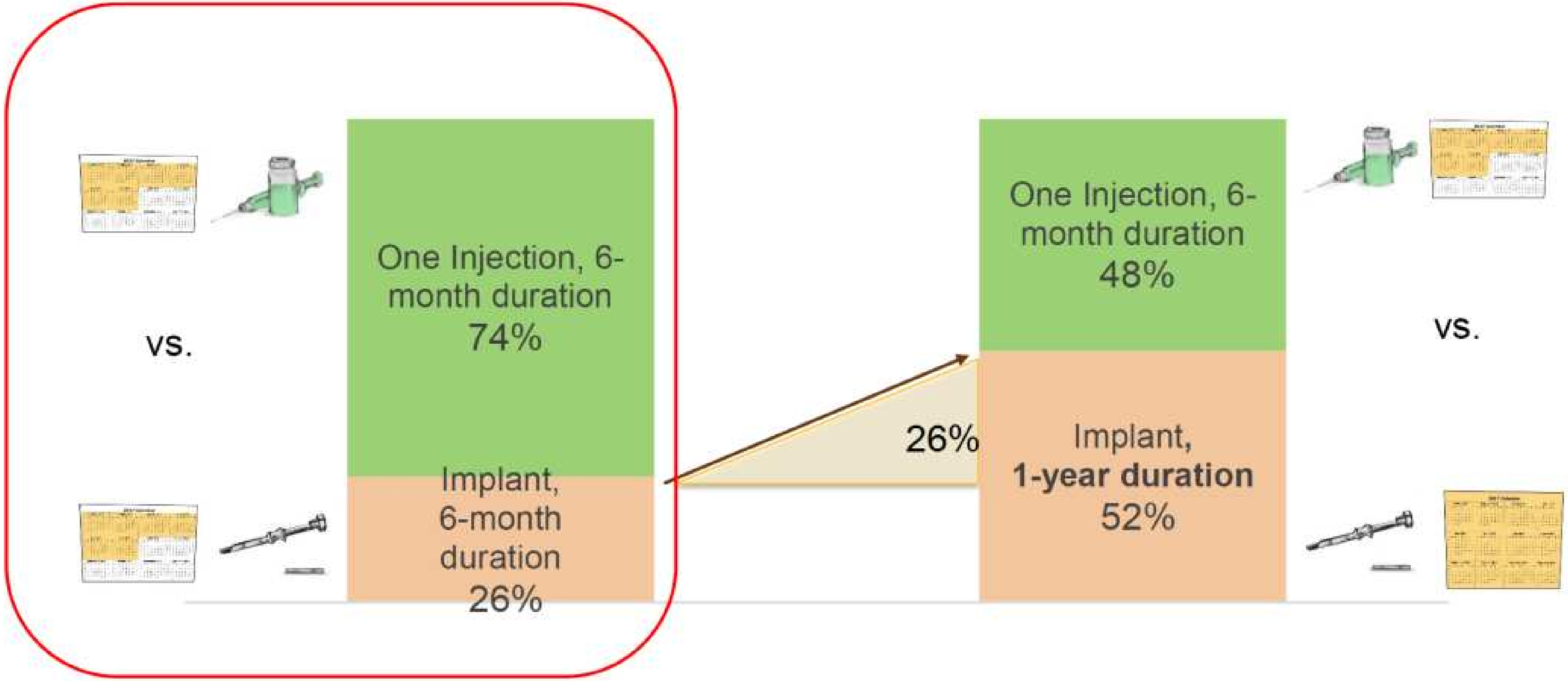

Steps for Conducting a DCE

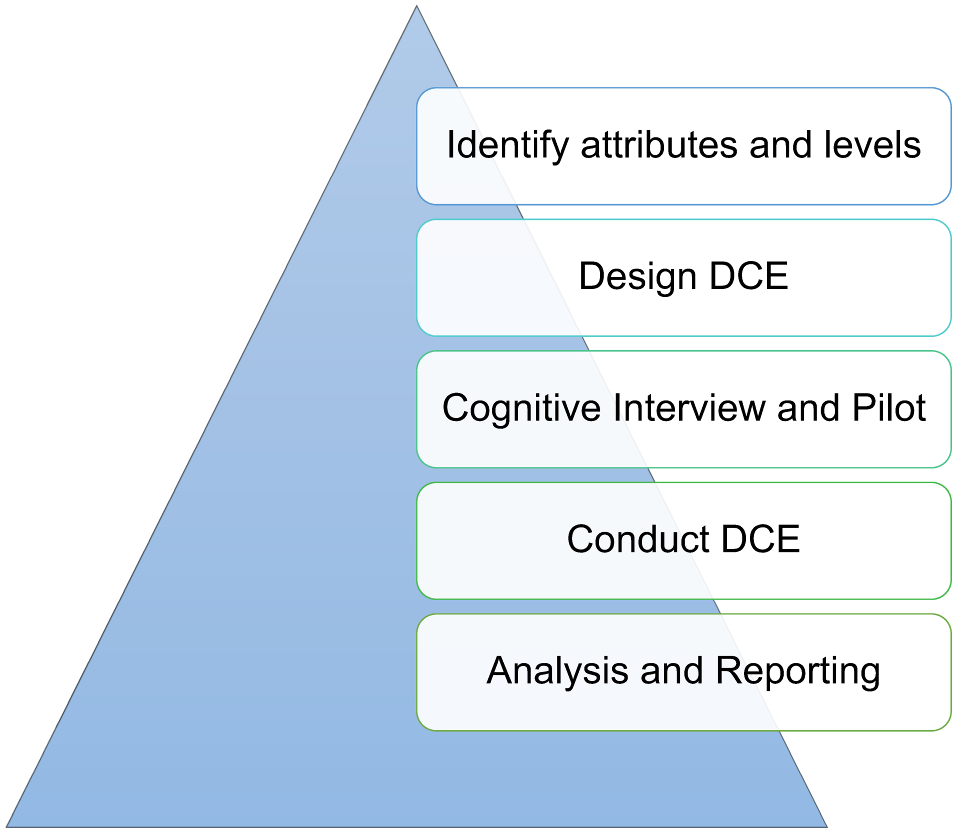

Attributes and Levels of ePharm DCE

Attribute	Description	Levels
**PrEP eligibility assessment**	Method for conducting client eligibility assessment for PrEP	• Online self-assessment using screening questions (phone number in case of questions)<br>• Guided assessment with a remote clinical provider (via a phone call or WhatsApp)
**HIV test type**	Type of HIV test delivered for PrEP initiation	• Oral Fluid HIV self-test (at setting of your choice)<br>• Blood-based HIV self-test (at setting of your choice)<br>• Healthcare provider administers HIV Test at setting of your choice (blood-based)
**Clinical consultation for prescribing PrEP**	Clinical consultation needed to prescribe PrEP	• Remote clinical consultation with provider (via a phone call or video chat)<br>• In-person clinical consultation with provider after completing HIV test (at a setting of your choice)
**User support options for PrEP**	Method for discussing your questions for PrEP with a healthcare provider	• SMS<br>• Phone/video call<br>• Email
**Cost of PrEP delivery**	Total cost of the PrEP delivery visit (including HIV self-test delivery and support, the remote clinician consultation, and PrEP delivery (one month of PrEP)	• 500 KES<br>• 2000 KES<br>• 4000 KES

Virtual PrEP delivery model

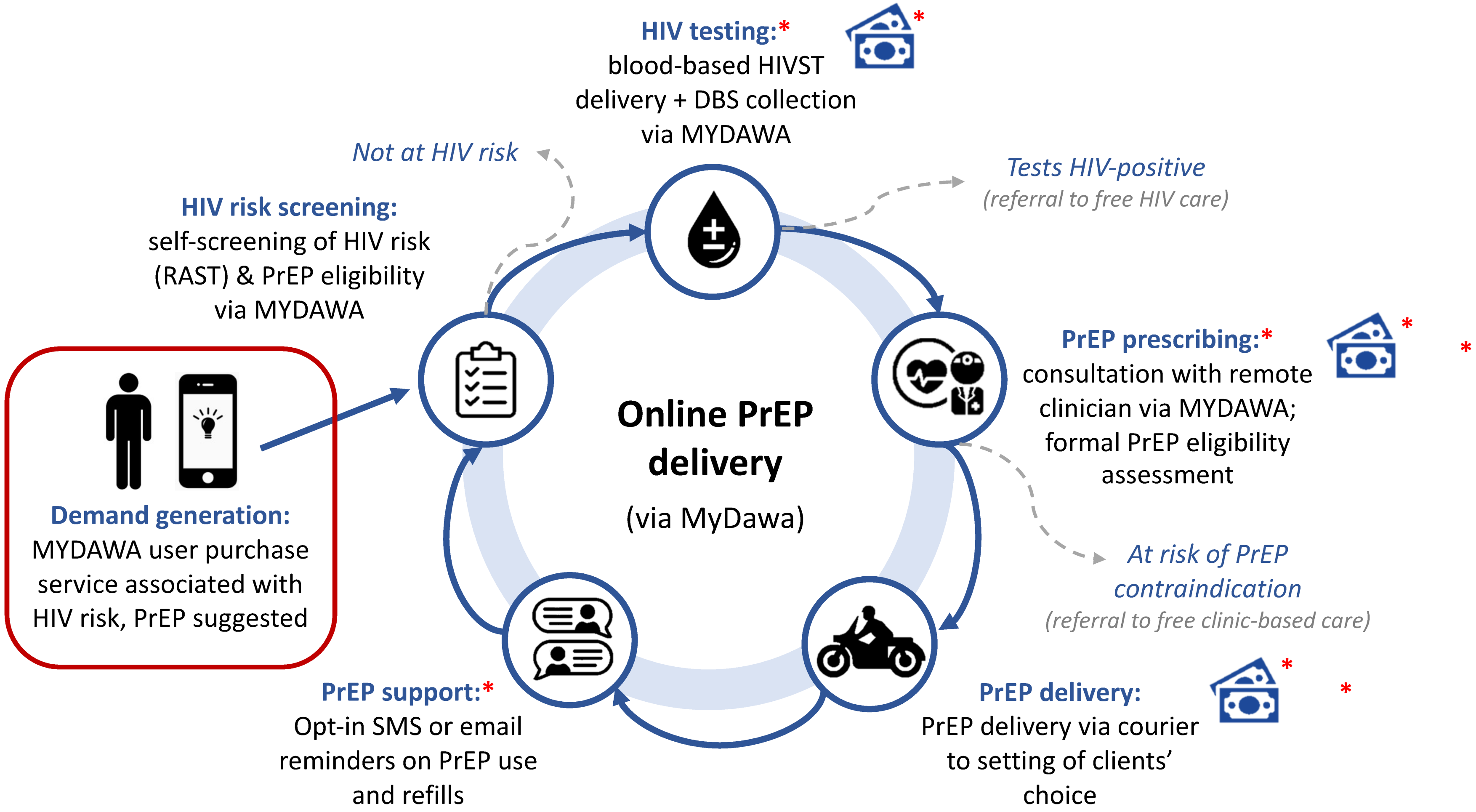

Fee potentially associated with the delivery of this model component

* *Implementation strategy likely to be updated with finding on client preferences from the DCE*

K. Ortblad 2021 ©

EPharmacy PrEP: Pilot Study Discrete Choice Experiment Training

Day 1 Session 2

24th-25th January 2022

BMJ Publishing Group Limited (BMJ) disclaims all liability and responsibility arising from any reliance placed on this supplemental material which has been supplied by the author(s)

Overview of study procedures

- Recruitment
- Pre-screening
- Consent
- Questionnaire administration (in-person)

Recruitment

- Target MYDAWA clients accessing products related to HIV/sexual health
- Banner ads on MYDAWA website, flyers included with product deliveries
- Potential participants will call study phone number or SMS/WhatsApp with the word #GenN if interested

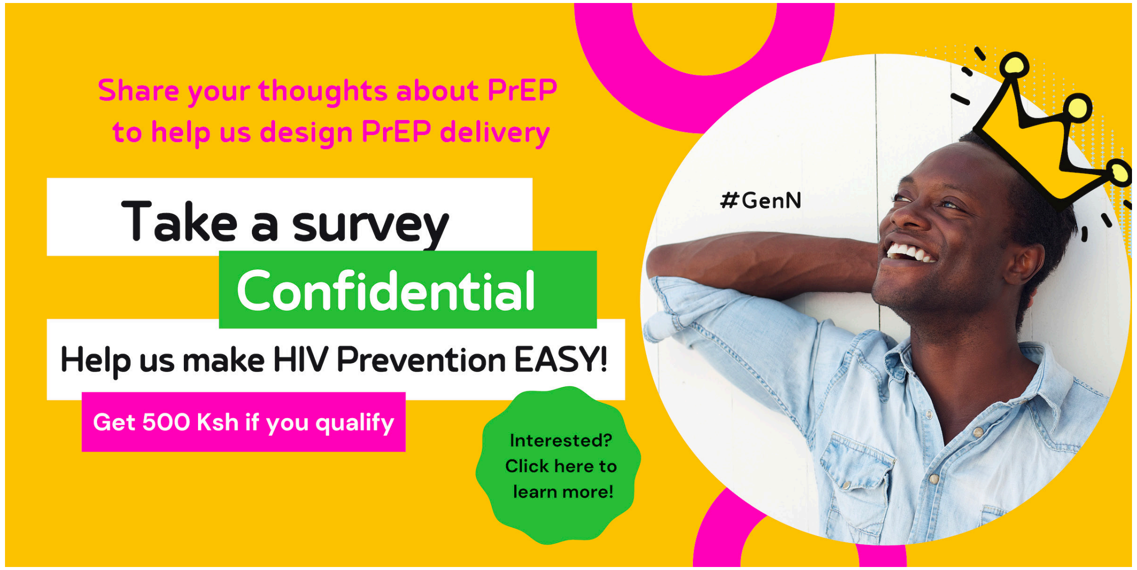

Pre-screening

- Pre-screening will take place by phone
- Study staff will complete REDCap pre-screening data entry form
  1. Eligibility assessment
  2. Schedule in-person visit

Eligibility criteria

- Participants are eligible to enroll in the study if they are:

  1. Age 18 or older
  2. HIV-negative or unknown HIV status
  3. Respond ‘yes’ to at least one of eleven screening questions about HIV risk
  4. Able and willing to provide informed consent

Eligibility Assessment

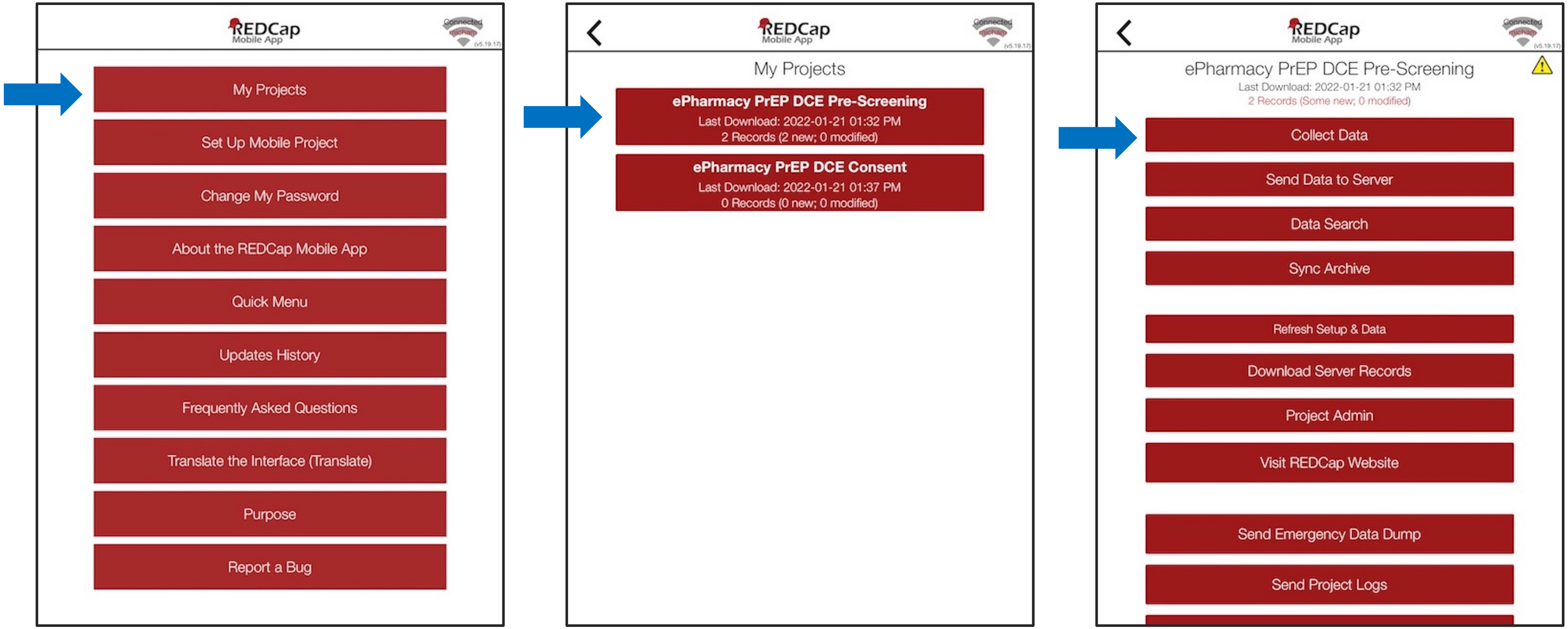

Eligibility Assessment

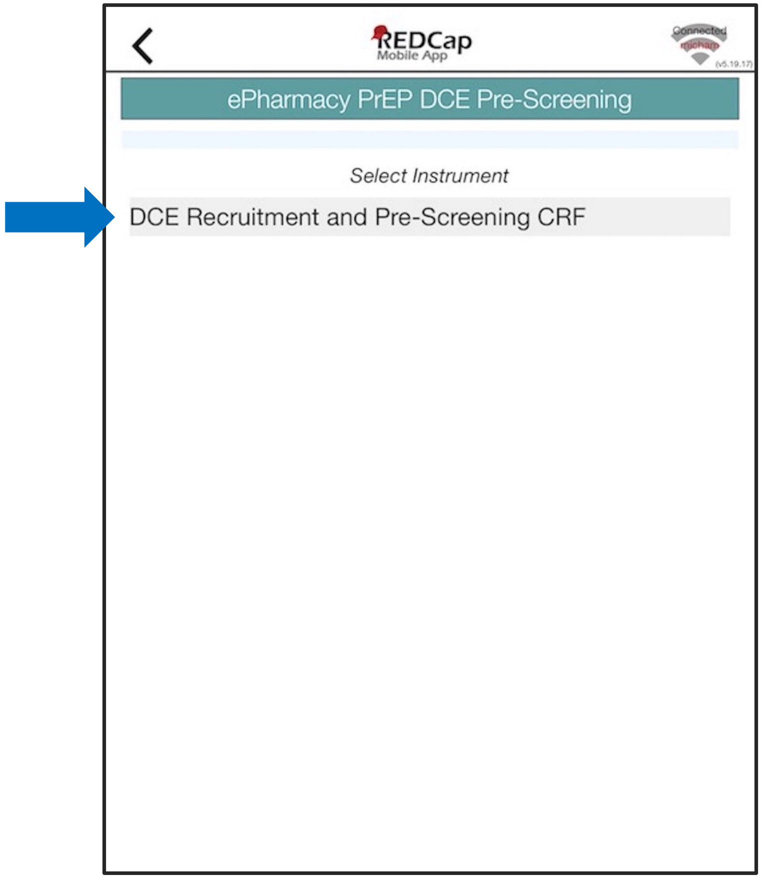

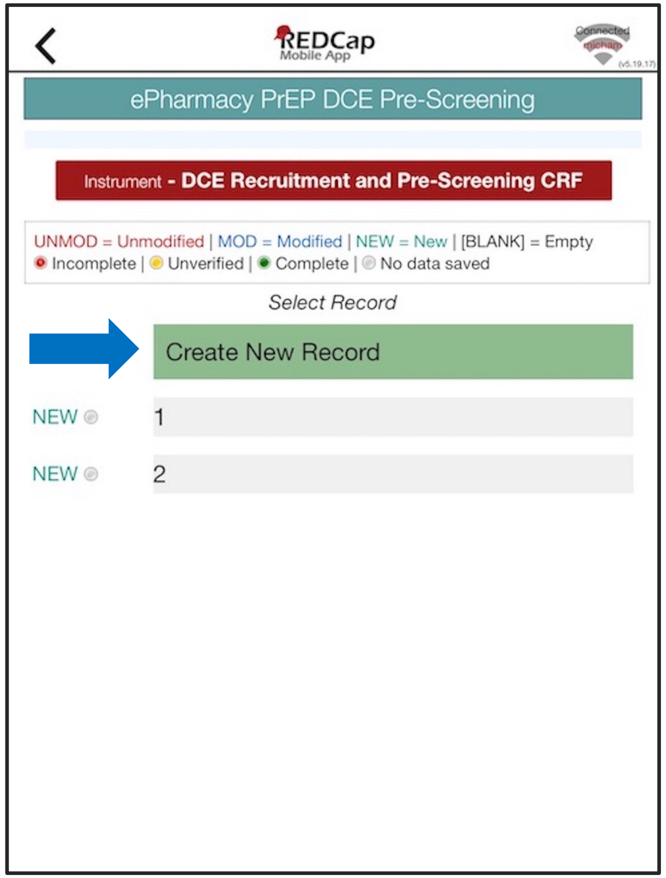

Eligibility Assessment

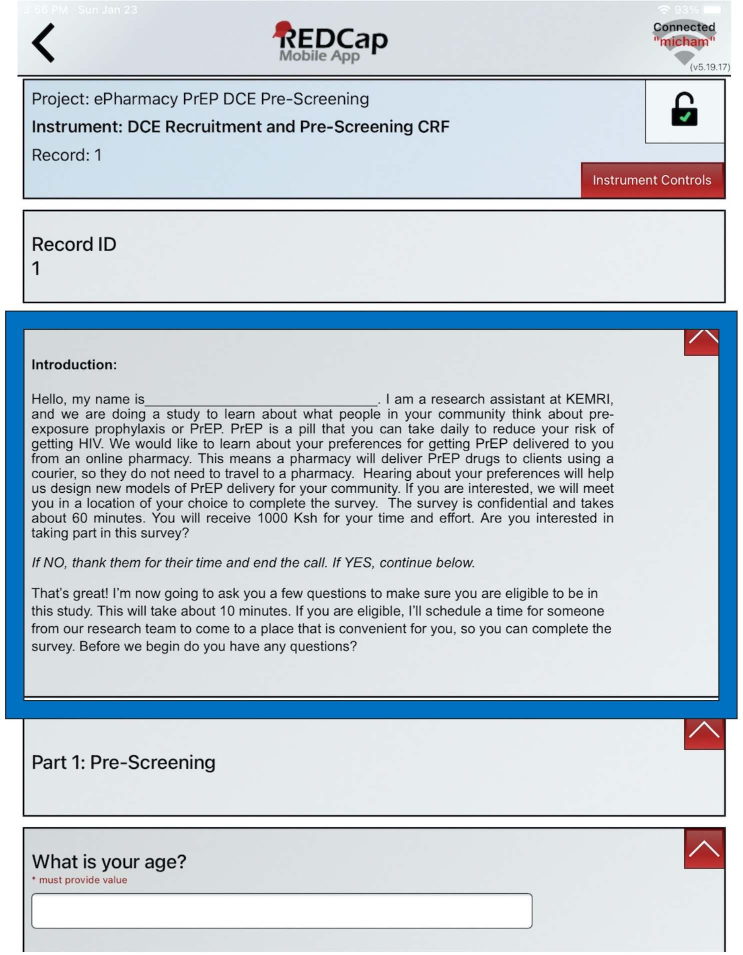

Eligibility Assessment

Introduction:

Hello, my name is______________________________. I am a research assistant at KEMRI, and we are doing a study to learn about what people in your community think about pre-exposure prophylaxis or PrEP. PrEP is a pill that you can take daily to reduce your risk of getting HIV. We would like to learn about your preferences for getting PrEP delivered to you from an online pharmacy. This means a pharmacy will deliver PrEP drugs to clients using a courier, so they do not need to travel to a pharmacy. Hearing about your preferences will help us design new models of PrEP delivery for your community. If you are interested, we will meet you in a location of your choice to complete the survey. The survey is confidential and takes about 60 minutes. You will receive 1000 Ksh for your time and effort. Are you interested in taking part in this survey?

If NO, thank them for their time and end the call. If YES, continue below.

That's great! I'm now going to ask you a few questions to make sure you are eligible to be in this study. This will take about 10 minutes. If you are eligible, I'll schedule a time for someone from our research team to come to a place that is convenient for you, so you can complete the survey. Before we begin do you have any questions?

BMJ Publishing Group Limited (BMJ) disclaims all liability and responsibility arising from any reliance placed on this supplemental material which has been supplied by the author(s)

Eligibility Assessment – Not eligible

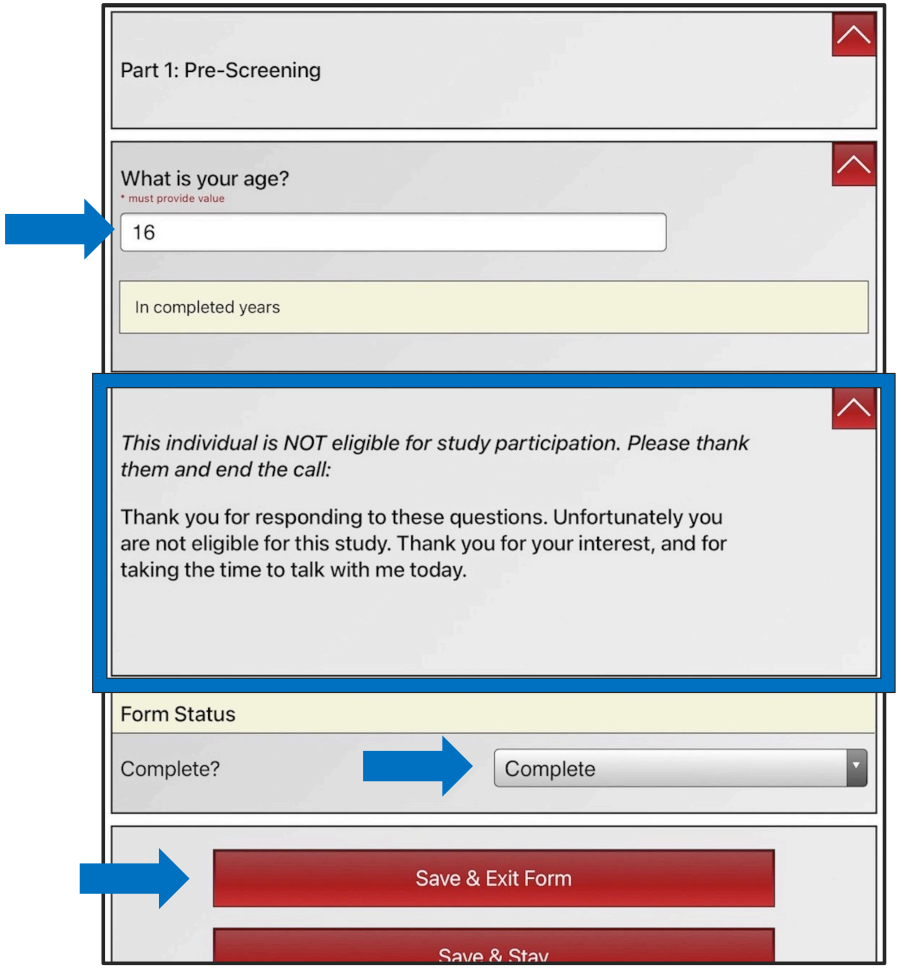

Eligibility Assessment – Not eligible

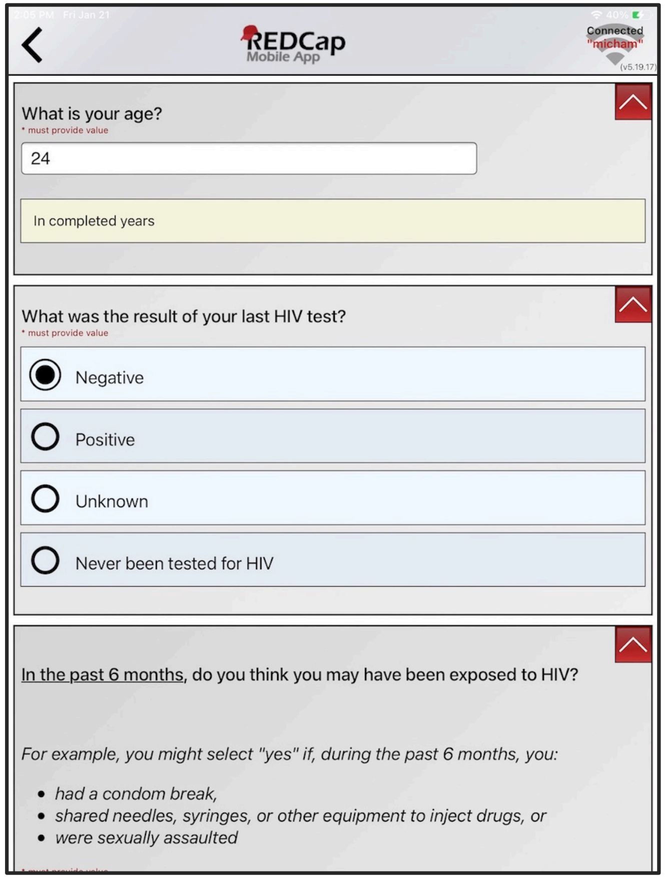

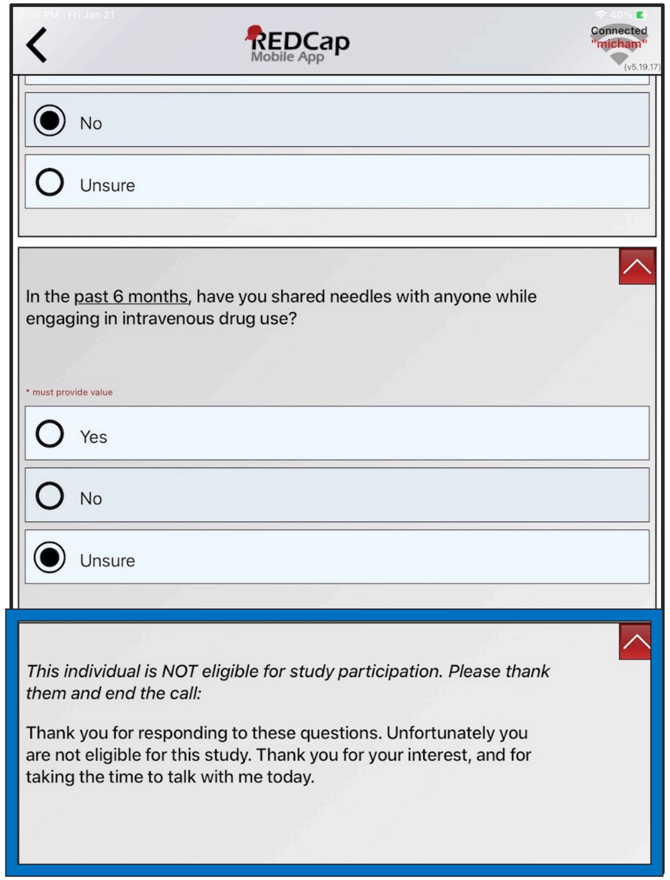

BMJ Publishing Group Limited (BMJ) disclaims all liability and responsibility arising from any reliance placed on this supplemental material which has been supplied by the author(s)

Eligibility Assessment – Eligible

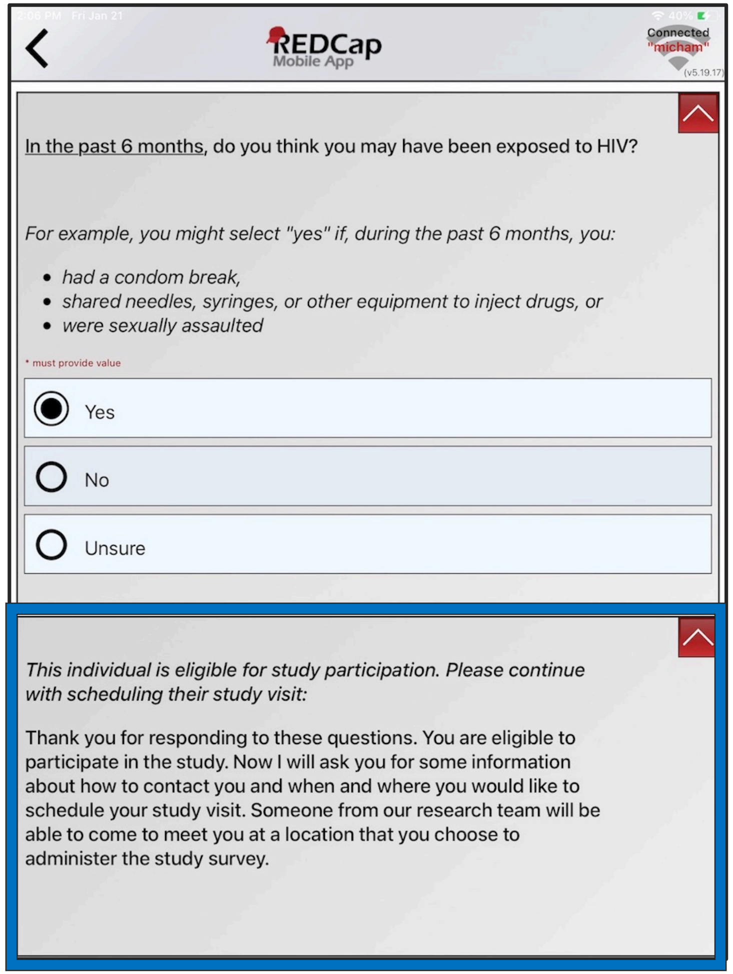

BMJ Publishing Group Limited (BMJ) disclaims all liability and responsibility arising from any reliance placed on this supplemental material which has been supplied by the author(s)

Eligibility Assessment – Scheduling and Contact Info

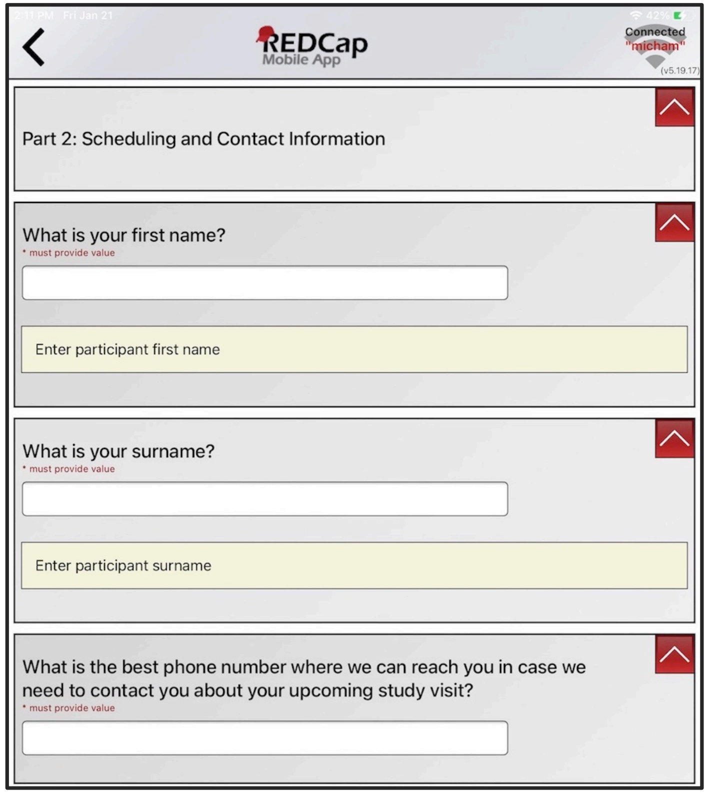

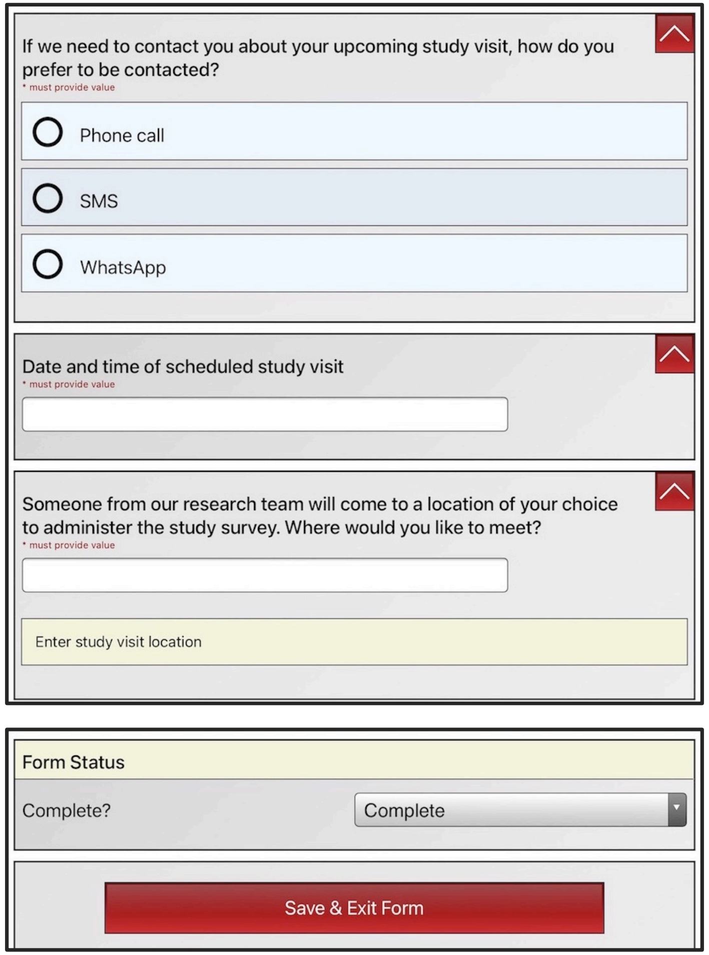

Consent procedures

- Research assistant will meet participant with tablet to administer ICF and questionnaire
- eConsent via REDCap app
- Review ICF with participant and answer questions
- Participant signs ICF on tablet

eConsent

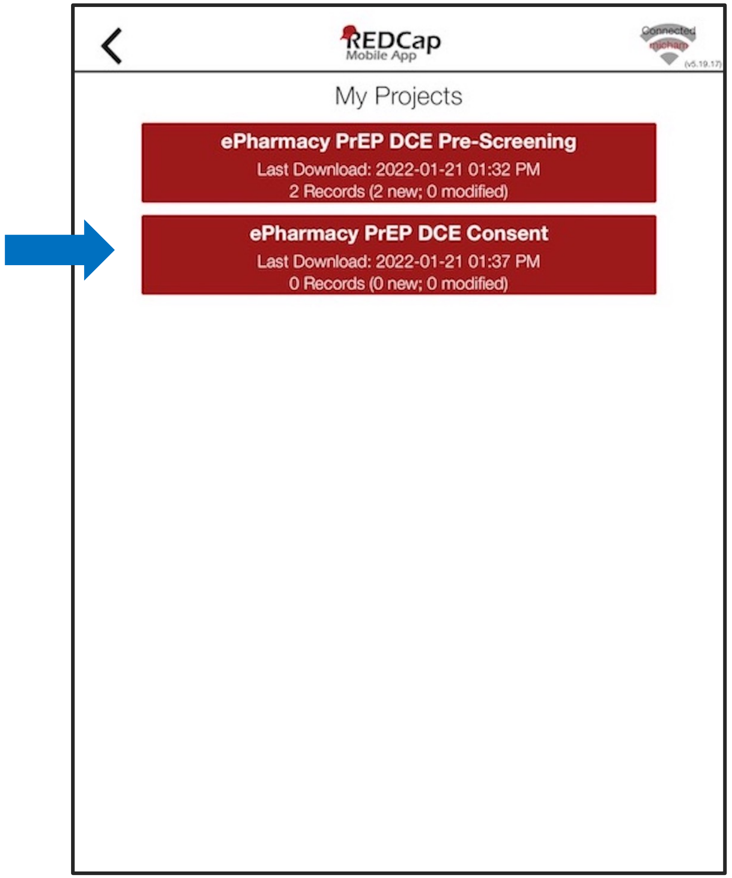
REDCap
Mobile App
My Projects
ePharmacy PrEP DCE Pre-Screening
Last Download: 2022-01-21 01:32 PM
2 Records (2 new; 0 modified)
ePharmacy PrEP DCE Consent
Last Download: 2022-01-21 01:37 PM
0 Records (0 new; 0 modified)

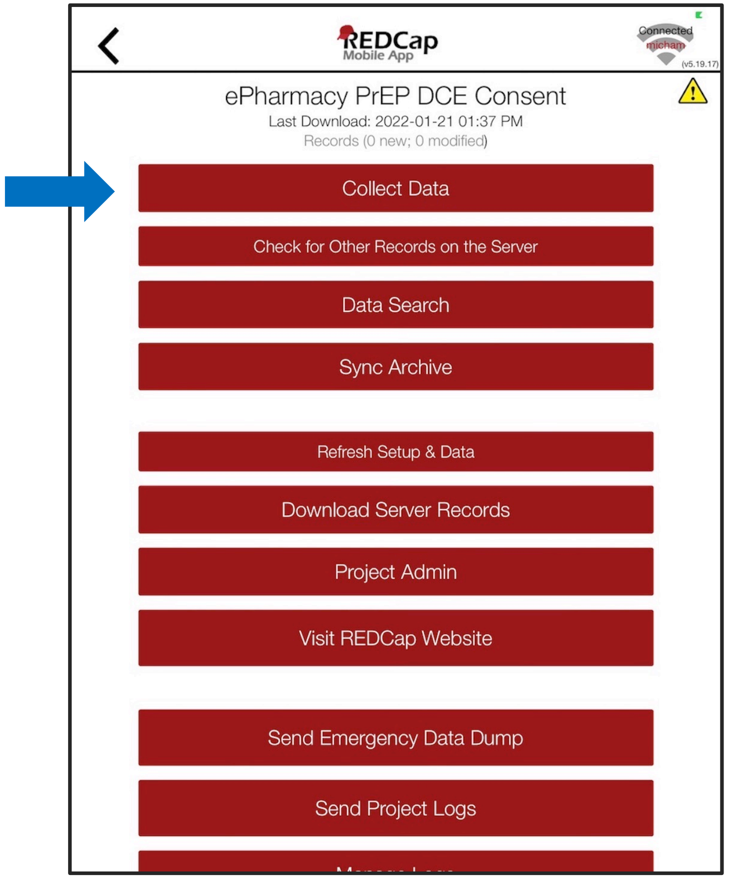
REDCap
Mobile App
ePharmacy PrEP DCE Consent
Last Download: 2022-01-21 01:37 PM
Records (0 new; 0 modified)
Collect Data
Check for Other Records on the Server
Data Search
Sync Archive
Refresh Setup & Data
Download Server Records
Project Admin
Visit REDCap Website
Send Emergency Data Dump
Send Project Logs

BMJ Publishing Group Limited (BMJ) disclaims all liability and responsibility arising from any reliance placed on this supplemental material which has been supplied by the author(s)

eConsent

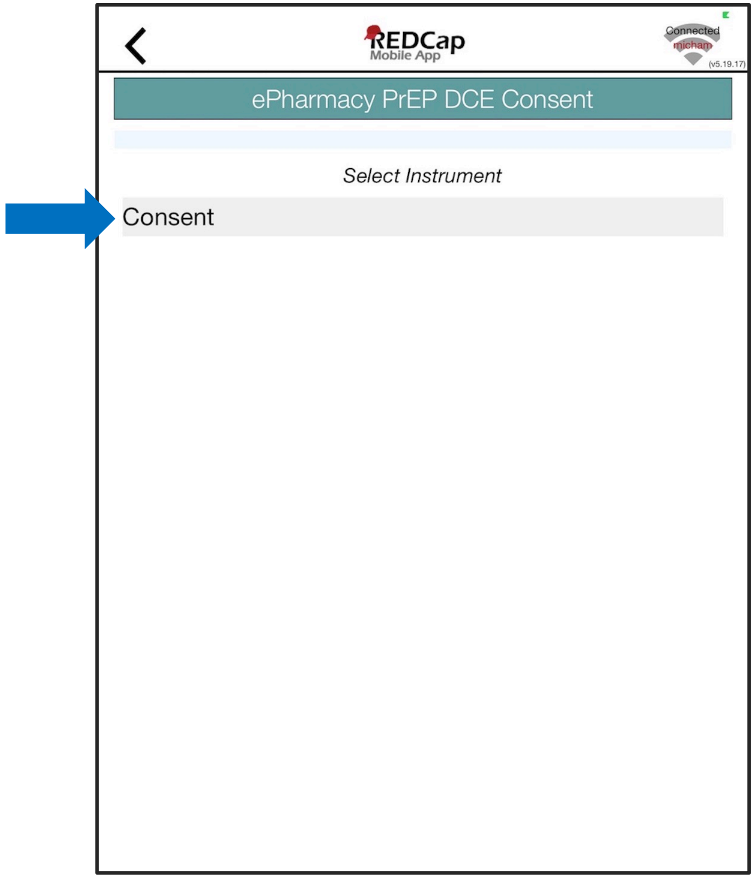

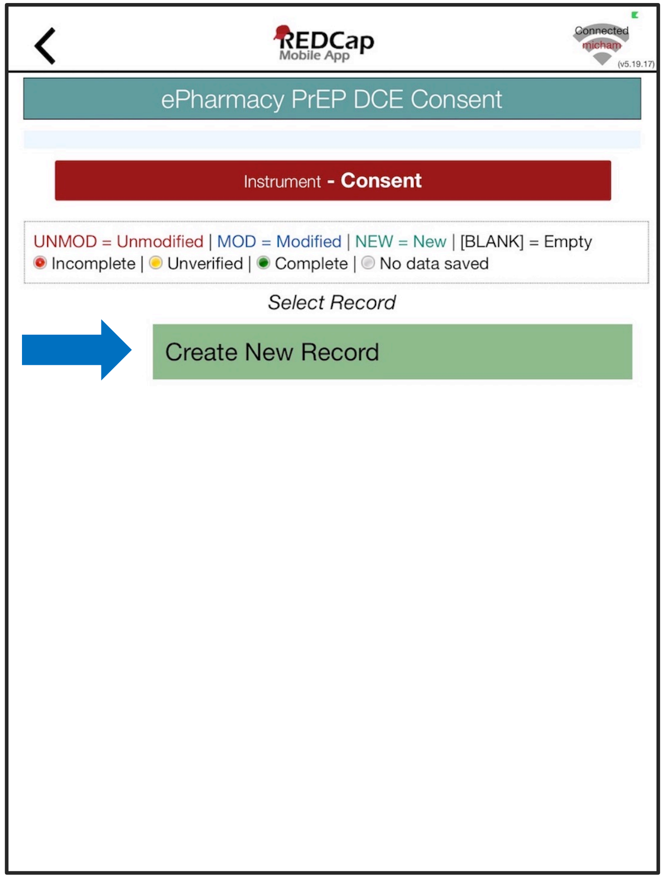

BMJ Publishing Group Limited (BMJ) disclaims all liability and responsibility arising from any reliance placed on this supplemental material which has been supplied by the author(s)

Sawtooth: types of questions

- Labels to be read
- Multiple-Choice
- Checklist
- Entering a Number or Text or Date
- DCE Scenarios

Sawtooth: labels to be read

- Labels do not require data entry
- Labels will have information for you and/or the participant
- In some cases, labels can be a script that can read aloud to participants
- For example, in **Questionnaire: Introduction;**

Introduction

Thank you for taking time to talk with me. We would like to speak to you about pre-exposure prophylaxis or PrEP. PrEP is a medicine you can take to reduce your risk of getting HIV. We have asked you to participate in this survey because we are interested in learning how best to delivery PrEP using an online pharmacy. This means a pharmacy will deliver PrEP medication to clients using a courier, so clients do not need to travel to a pharmacy. We would like to understand your preferences for PrEP delivery through an online pharmacy. This survey should take about 60 minutes. Feel free to let me know if you need a break at any time. You can also stop the survey if you do not want to continue. Before we begin do you have any questions?

Next

Sawtooth: multiple-choice questions

- You can only select one answer
- Example, **Questionnaire: Part 1: Eligibility Assessment;**

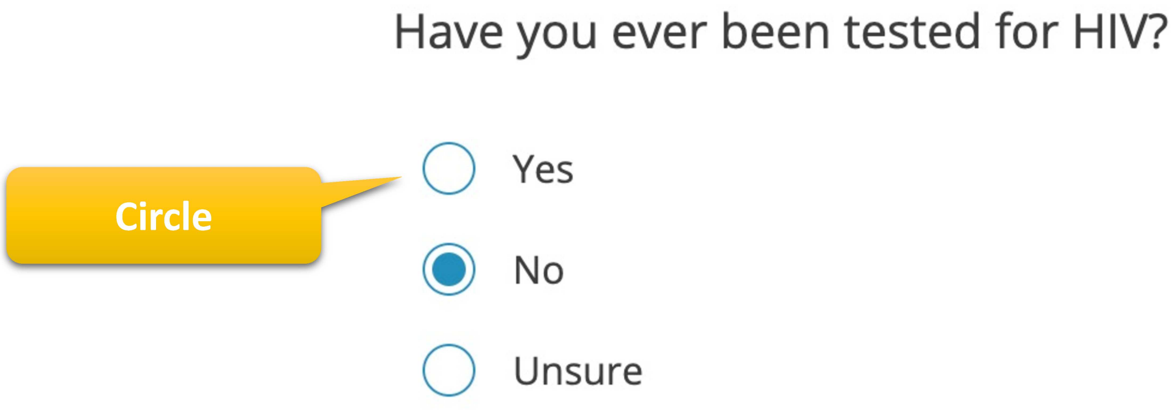

Sawtooth: checklist questions

- Here, you may select as many options as you like
- You should select all the answers that apply
- Example, **Questionnaire: Part 6: E-Pharmacy Engagement and HIV Self-Testing**

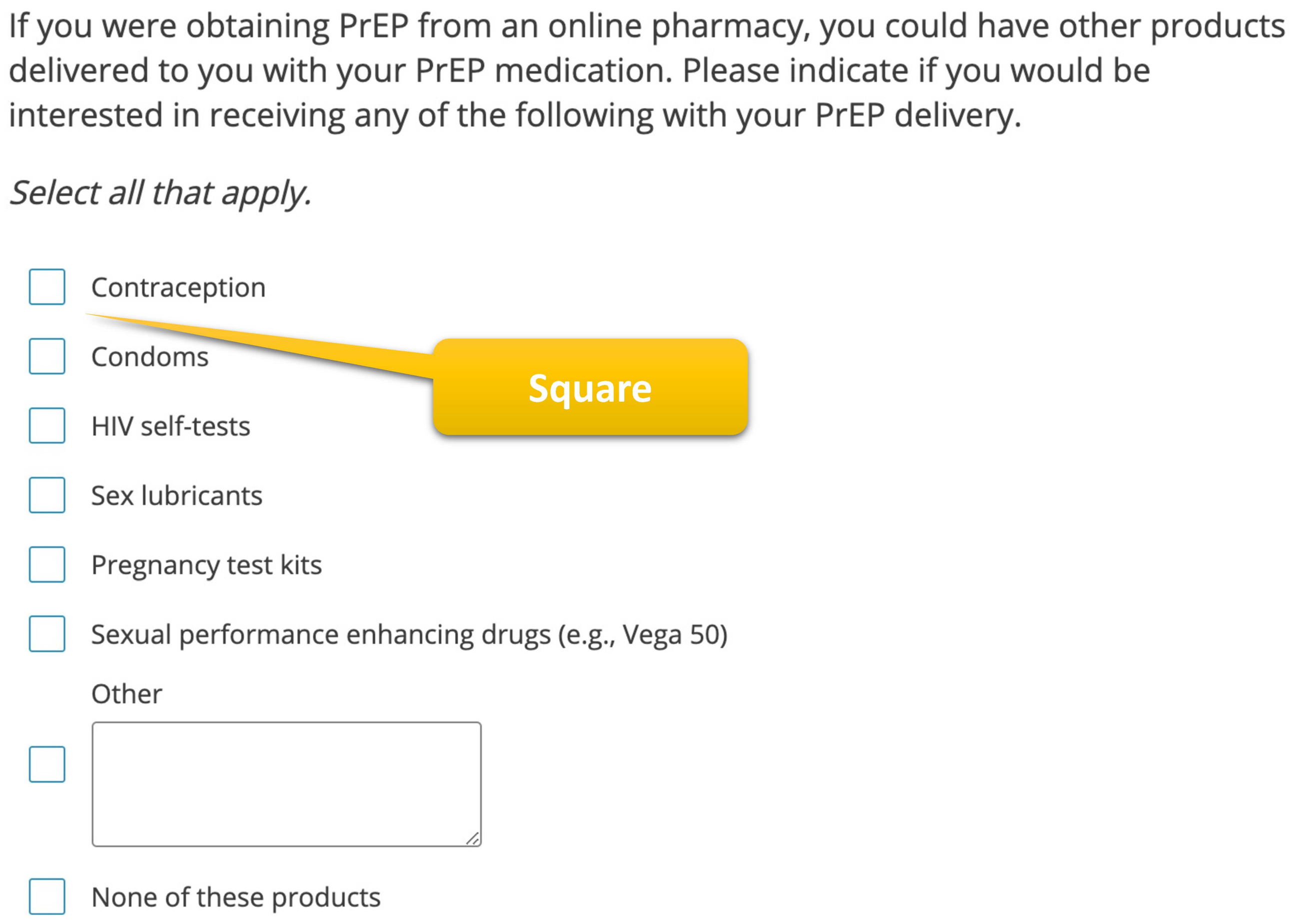
If you were obtaining PrEP from an online pharmacy, you could have other products delivered to you with your PrEP medication. Please indicate if you would be interested in receiving any of the following with your PrEP delivery.

Select all that apply.

- [ ] Contraception
- [ ] Condoms
- [ ] HIV self-tests
- [ ] Sex lubricants
- [ ] Pregnancy test kits
- [ ] Sexual performance enhancing drugs (e.g., Vega 50)
- [ ] Other
- [ ] None of these products

Sawtooth: entering numbers

- Entering number examples

Questionnaire: Part 1: Eligibility Assessment;

What is your age in years? [ ]

Questionnaire: Part 6: E-Pharmacy Engagement and HIV Self-Testing;

For a blood-based HIV self-test, delivered via a courier to a location of your choice (one-time cost):

What is the lowest price you are willing to pay? [ ] KSH

What is the highest price you are willing to pay? [ ] KSH

What price do you consider ideal? [ ] KSH

Sawtooth: entering text

- Entering text example
- Text questions will most often appear to clarify “Other”

If you were obtaining PrEP from an online pharmacy, you could have other products delivered to you with your PrEP medication. Please indicate if you would be interested in receiving any of the following with your PrEP delivery.

Select all that apply.

- ☐ Contraception
- ☐ Condoms
- ☐ HIV self-tests
- ☐ Sex lubricants
- ☐ Pregnancy test kits
- ☐ Sexual performance enhancing drugs (e.g., Vega 50)
- ☐ Other
- ☐ None of these products

Sawtooth: entering date

- Entering date examples
- Date questions will have drop-down menus

Questionnaire: Part 1: Eligibility Assessment

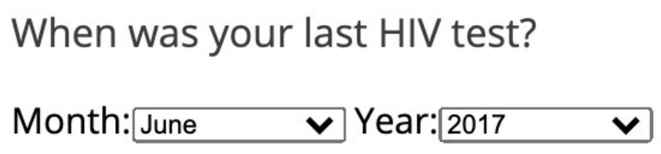

Questionnaire: Part 2: PrEP Knowledge and Interest

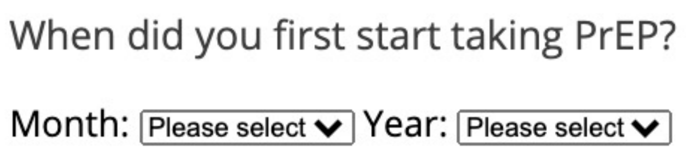

Sawtooth: required questions

- Almost every question requires an answer
- If you try to move on without providing an answer, a red screen will appear with a reminder that the response is required.

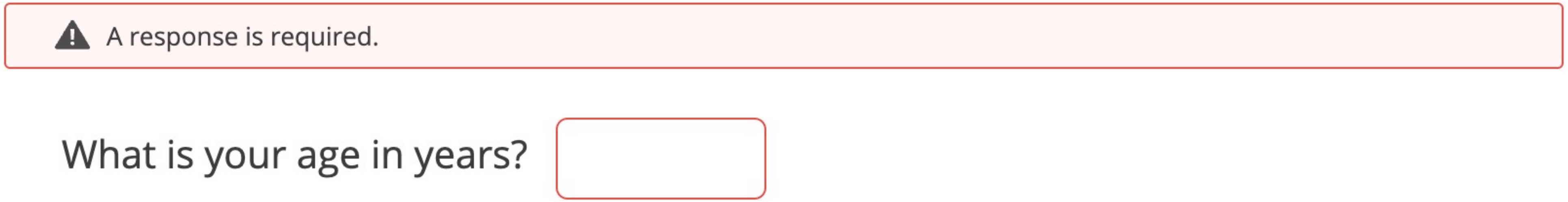

Sawtooth: DCE scenarios

- Explain in the next section

Structure of the Questionnaire

- Introduction
- Part 1: Eligibility Assessment
- Part 2: PrEP Knowledge and Interest
- Part 3: Introduction to Discrete Choice Experiment
- Part 4: Definition of Characteristics
- Part 5: Scenarios
- Part 6: E-Pharmacy Engagement and HIV Self-Testing
- Part 7: Participant Demographics
- Part 8: Sexual Behavior

Structure of the Questionnaire

- **Introduction**
- **Part 1: Eligibility Assessment**
- **Part 2: PrEP Knowledge and Interest**
- Part 3: Introduction to Discrete Choice Experiment
- Part 4: Definition of Characteristics
- Part 5: Scenarios
- Part 6: E-Pharmacy Engagement and HIV Self-Testing
- Part 7: Participant Demographics
- Part 8: Sexual Behavior

Introduction

Introduction

Thank you for taking time to talk with me. We would like to speak to you about pre-exposure prophylaxis or PrEP. PrEP is a medicine you can take to reduce your risk of getting HIV. We have asked you to participate in this survey because we are interested in learning how best to delivery PrEP using an online pharmacy. This means a pharmacy will deliver PrEP medication to clients using a courier, so clients do not need to travel to a pharmacy. We would like to understand your preferences for PrEP delivery through an online pharmacy. This survey should take about 60 minutes. Feel free to let me know if you need a break at any time. You can also stop the survey if you do not want to continue. Before we begin do you have any questions?

Next

Participant ID

- Participant ID should be entered twice
- Same PTID as ICF – links questionnaire responses to ICF

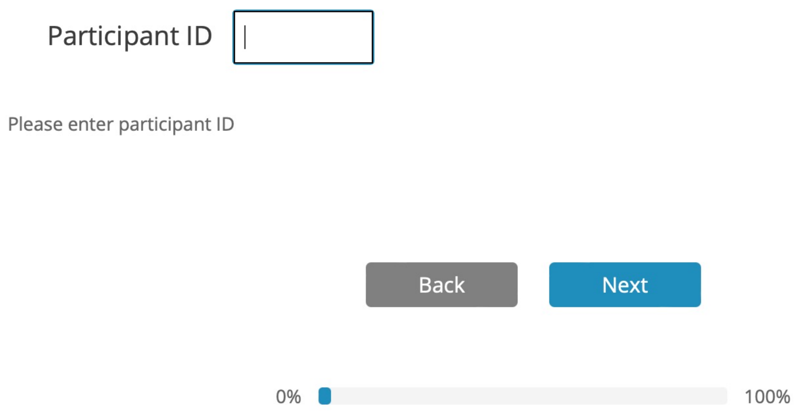

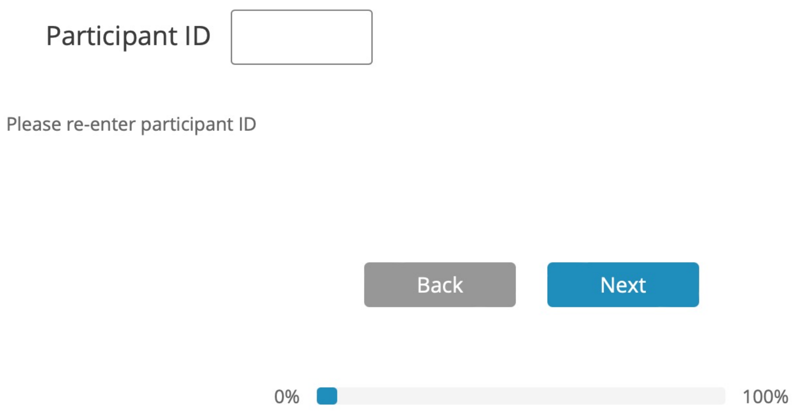

Part 1: Eligibility Assessment

- Eligibility assessment to be completed again at time of questionnaire administration

Part 1: Eligibility Assessment

What is your age in years? [ ]

Back | Next

In the past 6 months, do you think you may have been exposed to HIV?

For example, you might select "yes" if, during the past 6 months, you:

• had a condom break

• shared needles, syringes, or other equipment to inject drugs, or

• were sexually assaulted

- Yes
- No
- Unsure

In the past 6 months, have you had sex with more than one person?

- Yes
- No
- Unsure

Part 2: PrEP Knowledge and Interest

Part 2: PrEP Knowledge and Interest

Have you ever heard of pre-exposure prophylaxis, or PrEP, for HIV prevention before today?

- ○ Yes
- ○ No

Back Next

BMJ Publishing Group Limited (BMJ) disclaims all liability and responsibility arising from any reliance placed on this supplemental material which has been supplied by the author(s)

Structure of the Questionnaire

- Introduction
- Part 1: Eligibility Assessment
- Part 2: PrEP Knowledge and Interest
- **Part 3: Introduction to Discrete Choice Experiment**
- **Part 4: Definition of Characteristics**
- **Part 5: Scenarios**
- **Part 6: E-Pharmacy Engagement and HIV Self-Testing**
- Part 7: Participant Demographics
- Part 8: Sexual Behavior

Part 3: Introduction to Discrete Choice Experiment

As we mentioned, we're designing a new way to deliver PrEP through an online pharmacy. We would like to understand your preferences to help us design this service. Online PrEP delivery includes different services, including an assessment to determine if you are at risk for HIV, HIV testing to confirm you are HIV-negative, and talking to a medical provider to make sure PrEP is safe for you. To determine your preferences for these services, we will show you a series of **9** scenarios with **2** options for online PrEP service delivery. For each of these scenarios, we will ask you to choose the delivery strategy you most prefer.

Back Next

Part 4:
Definition of
Characteristics

Here we describe the characteristics of obtaining PrEP through online pharmacy in the order you would experience them. These are the characteristics we will ask you to consider when making a decision about which option you prefer. Please consider ONLY these characteristics when making your choices.

Clinical Consultation

You would then have a clinical consultation with a medical provider to make sure PrEP is safe for you to use. This can be done over the phone or a video chat.

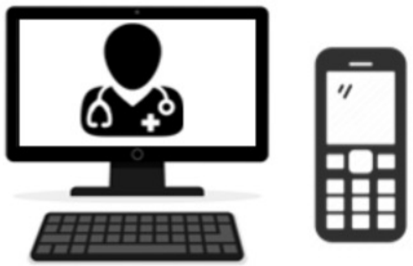

Another option is to see a provider in person at a setting of your choice.

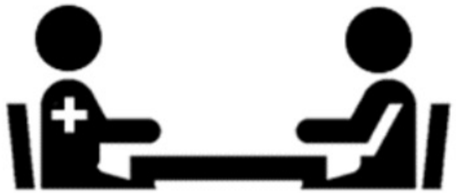

Part 5: Scenarios

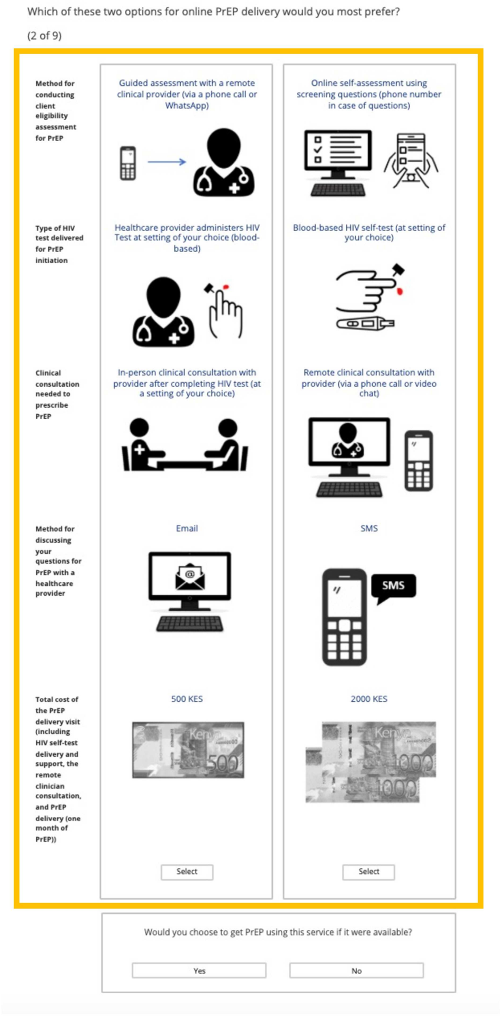

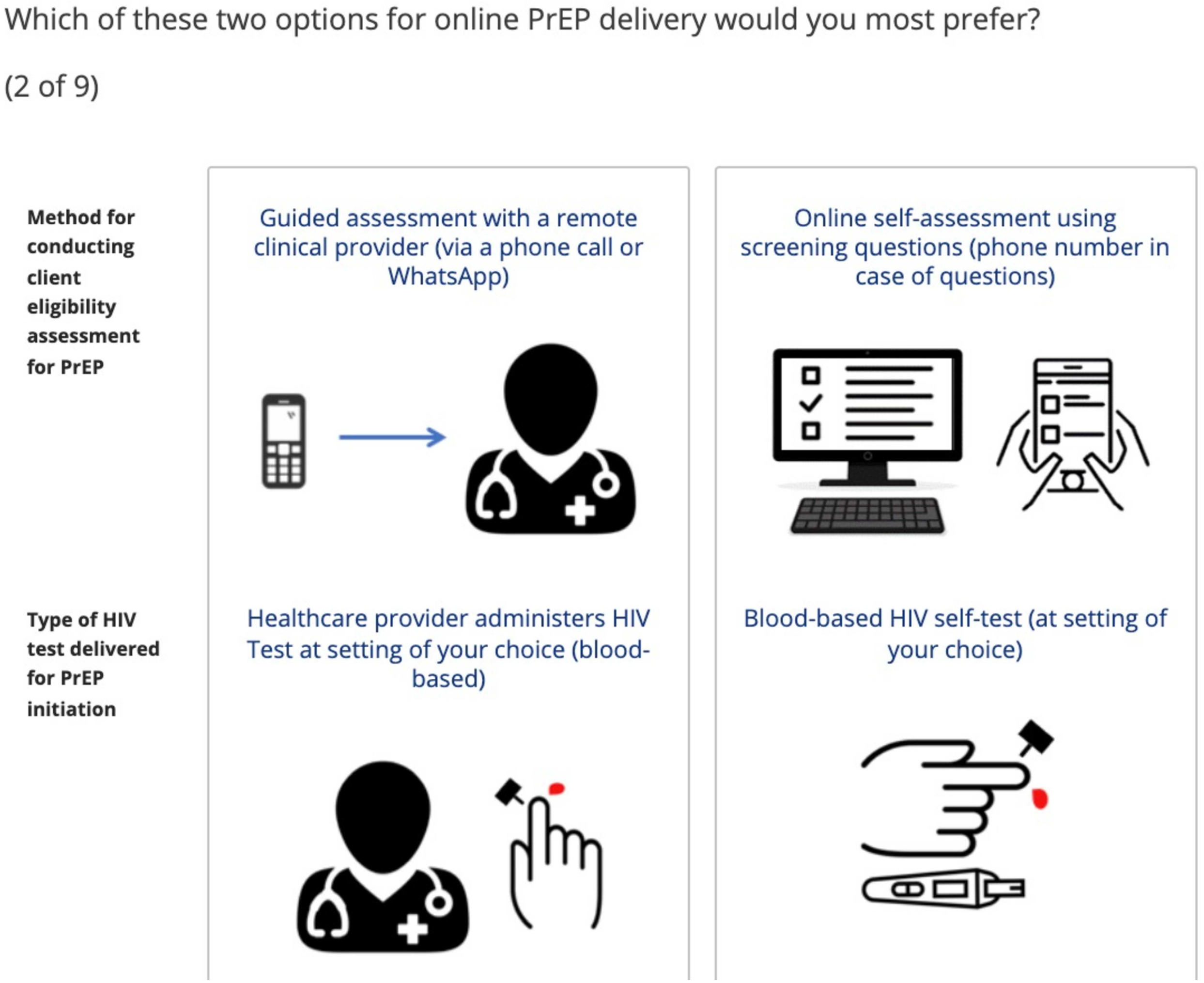

Part 5: Scenarios

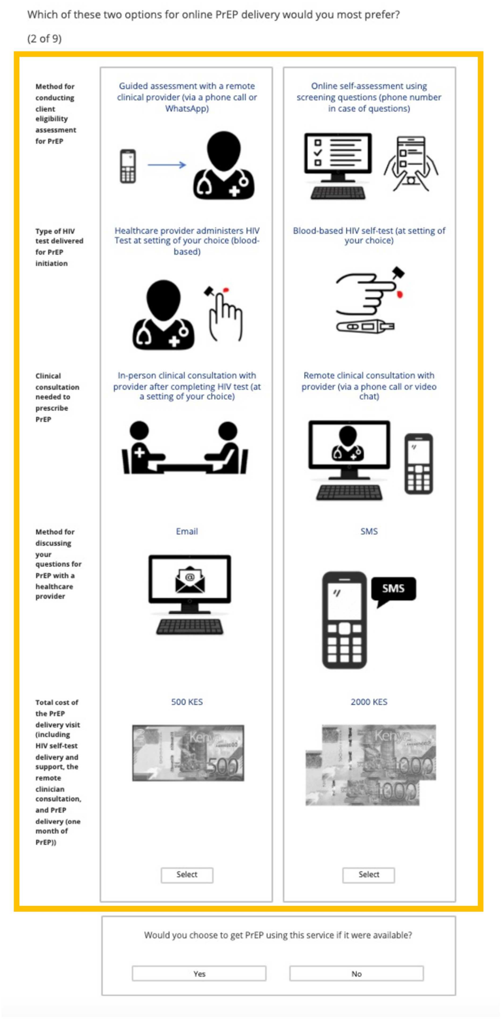

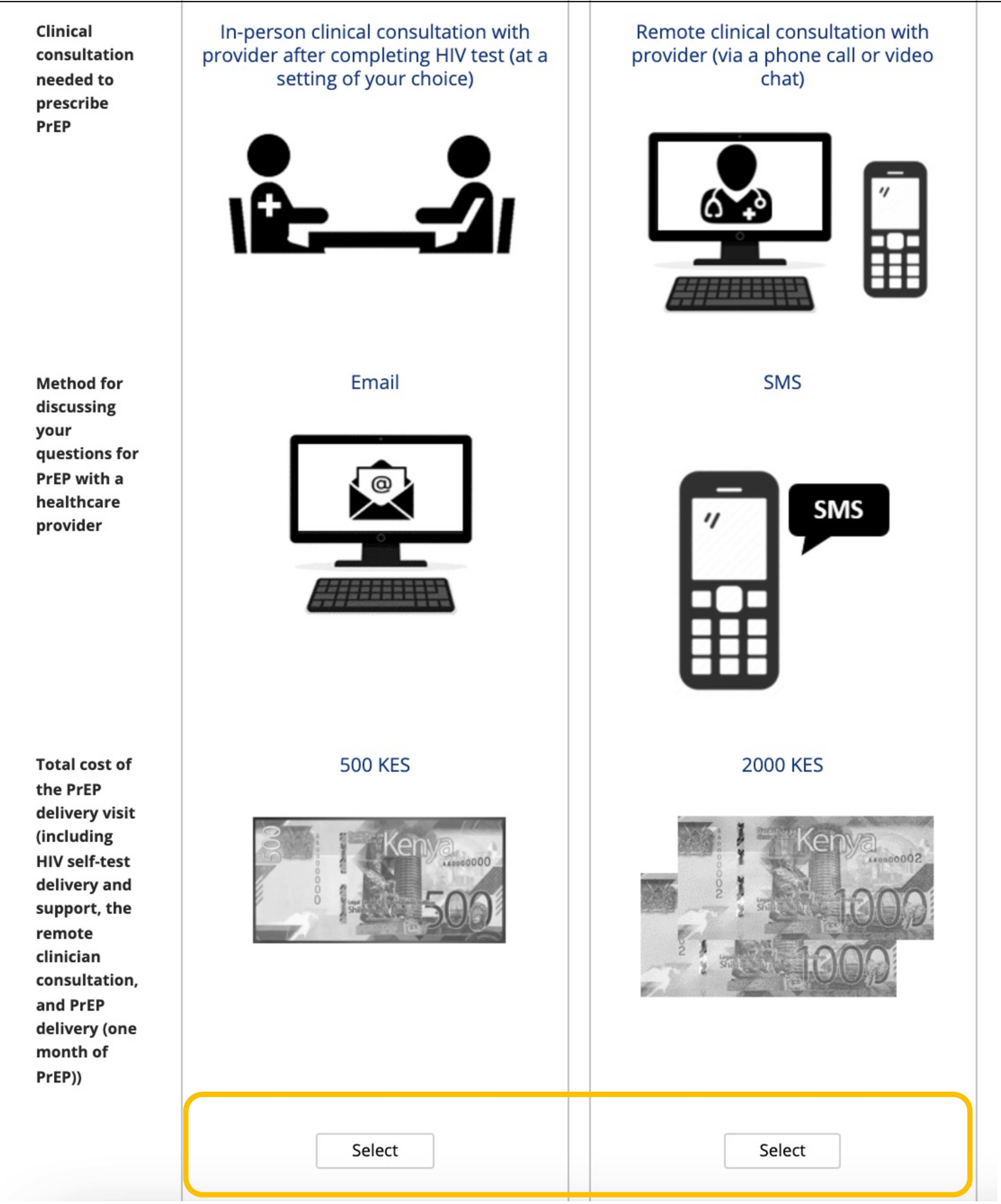

Part 5: Scenarios

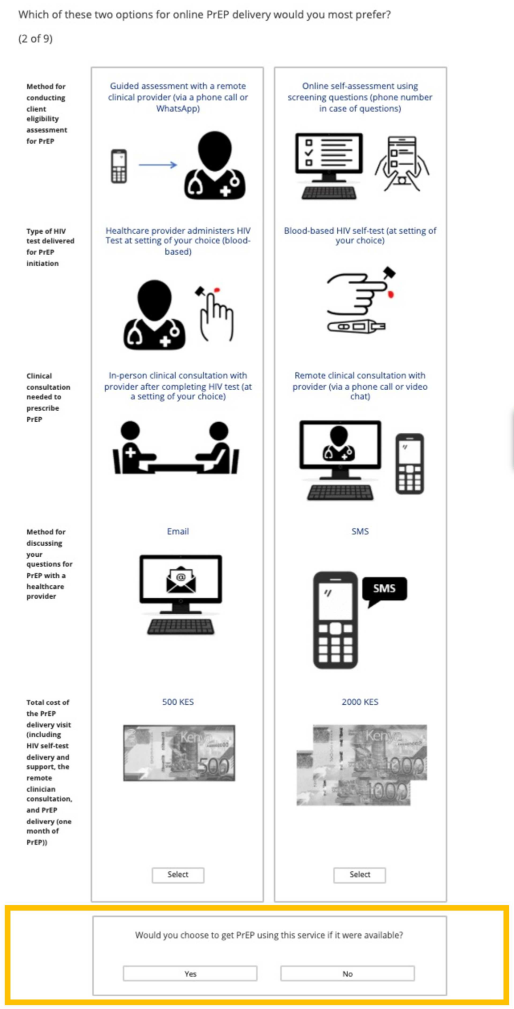

Dual-response question:

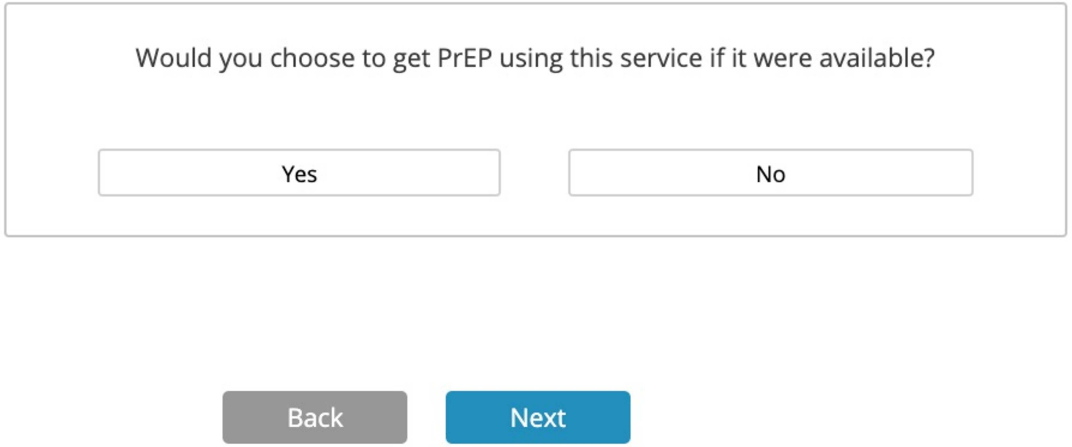

Part 6: E-Pharmacy Engagement and HIV Self-Testing

- Willingness-to-pay questions

The next set of questions will ask about how much you would like to pay for different parts of online PrEP delivery. For each part, please provide the lowest price, the maximum price and the ideal price you are willing to pay. For the lowest price, please tell us the lowest amount you think the service should cost because otherwise you would be concerned about its quality, and you would not purchase the service. In answering these questions, please consider your usual expenses. Remember that there are no right or wrong answers. We're interested in your preference. You can write zero if you feel that most accurately reflects your preference.

For a blood-based HIV self-test, delivered via a courier to a location of your choice (one-time cost):

What is the lowest price you are willing to pay? ______ KSH

What is the highest price you are willing to pay? ______ KSH

What price do you consider ideal? ______ KSH

Structure of the Questionnaire

- Introduction
- Part 1: Eligibility Assessment
- Part 2: PrEP Knowledge and Interest
- Part 3: Introduction to Discrete Choice Experiment
- Part 4: Definition of Characteristics
- Part 5: Scenarios
- Part 6: E-Pharmacy Engagement and HIV Self-Testing
- **Part 7: Participant Demographics**
- **Part 8: Sexual Behavior**

Part 7: Participant Demographics

Part 7: Participant Demographics

In what region do you live?

- North Eastern
- Nyanza
- Western
- Rift Valley
- Nairobi
- Central
- Coast
- Eastern

What is the highest level of education you have completed?

- Primary
- Secondary
- O Levels
- A Levels
- Technical or vocational school
- University or higher

Part 8: Sexual Behavior

- Prefer not to answer option

Part 8: Sexual Behavior

In the last 3 months, have you had a primary sex partner?

(A primary sex partner is a person you have sex with on a regular basis, or someone you consider to be your main partner.)

- ○ Yes
- ○ No
- ◉ Prefer not to answer

Submitting data

The questionnaire ends here. Thank you for your responses.

0% 100%

BMJ Publishing Group Limited (BMJ) disclaims all liability and responsibility arising from any reliance placed on this supplemental material which has been supplied by the author(s)

Questions?

ePharmacy PrEP: Discrete Choice Experiment Training

Day 1 Session 3
24th-25th January 2022

BMJ Publishing Group Limited (BMJ) disclaims all liability and responsibility arising from any reliance placed on this supplemental material which has been supplied by the author(s)

Cognitive Interviews, Pilot, and DCE Administration

Process

CI

Allow us confirm that the information used to describe the attributes is accurate and some of the questions in the survey are easy to understand and answer

PILOT

Help us confirm that the instrument (web-based survey) works well, 30 minutes is enough to answer all, and the participants are not (too) tired at the end

DCE

After adding the feedback from the CI and Pilot, the DCE will improve and allow us to collect the best data possible. Good-quality data will inform the creation of a good online-pharmacy service for PrEP delivery

BMJ Publishing Group Limited (BMJ) disclaims all liability and responsibility arising from any reliance placed on this supplemental material which has been supplied by the author(s)

Key characteristics

Cognitive Interviews

- Aim to obtain feedback in specific sections of the instrument
- Length: around 1 hour
- Uses a script
- RA very involved

Pilot

- Aim to verify usability and fatigue in the entire instrument
- Length: around 45 min
- No script
- RA involved but less

DCE

- Aims to obtain data of the participants' preferences as unbiased as possible
- Length: 30-45 min
- No script
- RA only involved if participant has questions

Structure – Cognitive Interviews

1. Approach and recruitment
   - In-person
   - Objective: to collect participants' feedback about the DCE attributes and specific sections of the instrument (see script)
   - Length: around 60 minutes
2. Presentation of the instrument
3. Active collection of feedback

Let's see the script

Structure – Pilot

1. Approach and recruitment
   - In-person
   - Objective: to verify if the instrument is friendly and easy to use, if the participants get stuck in any question, if any questions are not clear, how long does it take to complete the instrument, and how tired or bored is the participant at the end
   - Length: around 45 minutes
2. Presentation of the instrument
3. Passive collection of feedback:

   Observe closely while the participant goes through the survey, if at any point there's confusion ask what's happening and offer help. Take notes every time there's confusion or the participant needed clarification, how long it took to finish the survey, and if they were tired and to what extend.

Let's see the instrument

Structure – DCE

1. Approach and recruitment
   - In-person
   - Objective: to collect high-quality data about the participants' preferences.

   We assist but we don't advice
   - Length: around 30-45 minutes
2. Presentation of the instrument
3. Passive collection of feedback:

   After recruitment, present the survey to the participant and let them fill their answers.

Structure – DCE cont.

Clarifying vs. Advice questions

- Clarifying: Related to the meaning of a word or the sense of the question in case of ambiguity or vague meaning
  - Examples:
    - What does 'pay-by-piece' mean?
    - Is contraception the same as emergency pills?
    - Where do I click if I don't know the answer to this question (or don't want to tell)?
- Advice: asks for your opinion or advice
  - Examples:
    - How can I know if clinical consultation is more important than Cost?
    - Do you think oral HIV testing is easier than when they draw blood?

Summary

- Each step help us have the best possible instrument for the DCE
- Cognitive Interviews require the most involvement to obtain feedback, followed by Pilot, and in the DCE we collect no feedback but just administer the survey
- In the DCE we want to collect only the participants' preferences, so we avoid providing any opinion or advice, even if they ask
- Questions?
