# Supplementary material for: Assessing preferences for HIV pre-exposure prophylaxis (PrEP) delivery services via online pharmacies in Kenya: protocol for a discrete choice experiment
Source: BMJ Open. 2023 Apr 3;13(4):e069195. doi: 10.1136/bmjopen-2022-069195 (PMC10083853; doi:10.1136/bmjopen-2022-069195)
Supplement: Supplementary data [file bmjopen-2022-069195supp003.pdf]

Supplement 3: Study Questionnaire

DISCRETE CHOICE EXPERIMENT QUESTIONNAIRE

Introduction:

Thank you for taking time to talk with me. We would like to speak to you about pre-exposure prophylaxis or PrEP. PrEP is a medicine you can take to reduce your risk of getting HIV. We have asked you to participate in this survey because we are interested in learning how best to delivery PrEP using an online pharmacy. This means a pharmacy will deliver PrEP medication to clients using a courier, so clients do not need to travel to a pharmacy. We would like to understand your preferences for PrEP delivery through an online pharmacy. This survey should take about 45 minutes. Feel free to let me know if you need a break at any time. You can also stop the survey if you do not want to continue. Before we begin do you have any questions?

RA Initials
Research Assistant Initials
(The RA conducting this questionnaire should enter their initials.)

Participant ID
Please enter participant ID from enrollment log.
(This should match the PTID entered on the REDCap consent form.)

Please re-enter participant ID from enrollment log.
(This should match the PTID entered on the REDCap consent form.)

Part 1: Eligibility Assessment

1	What is your age in years?	|__|__| years <18, Stop screening. This individual is <u>NOT</u> eligible for study participation. Thank them for their time
2	Have you ever been tested for HIV?	☐ *Yes* ☐ *No* ☐ *Unsure*
3	When was your last HIV test?	_______ Month ______Year
4	What was the result of your last HIV test?	☐ *Negative*<br>☐ *Positive*<br>☐ *Unknown*<br><br>If positive, this individual is <u>NOT</u> eligible for study participation. Stop screening and thank them for their time.

Part 2: PrEP Knowledge and Interest

5	Have you ever heard of pre-exposure prophylaxis, or PrEP, for HIV prevention before today?	Yes<br>No
6	Have you ever taken PrEP to prevent HIV? *[If 5=Yes]*	Yes<br>No<br>I don't know<br>Prefer not to answer
6.1	Are you currently taking PrEP to prevent HIV? *[If Q6 = Yes]*	Yes<br>No

6.2	When did you first start taking PrEP? *[If Q6 = Yes]*	Month: Year:<br>MM/YYYY
7	Would you be interested in taking PrEP to prevent HIV? *[If Q6 != Yes]*	Yes<br>No<br>I don't know

Persons who answer NO to both of Q6, Q7 are not eligible for participation

If participant is not eligible: Based on your responses, you are not eligible to continue with the questionnaire. Thank you for taking the time to respond.

Part 3: Introduction to Discrete Choice Experiment

As we mentioned, we're designing a new way to deliver PrEP through an online pharmacy. We would like to understand your preferences to help us design this service. Online PrEP delivery includes different services, including an assessment to determine if you are at HIV risk, HIV testing to confirm you are HIV-negative, and talking to a medical provider to make sure PrEP is safe for you. To determine your preferences for these services, we will show you a series of **8** scenarios with 2 options for online PrEP service delivery. For each of these scenarios, we will ask you to choose the delivery strategy you most prefer.

Part 4: Definition of Characteristics

Part 5: Scenarios

We will now ask you about how you would like to get PrEP from an online pharmacy. Please remember there are no wrong answers, we want to know your preferences. When answering each scenario, imagine that you are planning to get PrEP using an online pharmacy. We will also ask you if you would choose to get PrEP using the option you selected. If you would not choose to get PrEP if only these two options were available, you can answer no to this question.

Scenarios

Which of these two options for online PrEP delivery would you most prefer?

If the PrEP service you just chose was available, do you think you would actually use it?

Part 6: E-Pharmacy Engagement and HIV Self-Testing

8	Have you ever purchased medication or other products from an online pharmacy (e-pharmacy)?	Yes<br>No
9	Have you ever purchased any of the following medications or products from an online pharmacy (e-pharmacy)? *[If Q8 = Yes]*<br>*Select all that apply*	Contraception<br>Condoms<br>HIV self-tests<br>Sex lubricants<br>Pregnancy test kits<br>Sexual performance enhancing drugs (e.g., Vega 50)<br>Other<br>None of these services
10	How would you most prefer to obtain your PrEP medication? *[If Q7 = Yes]*	Clinic<br>Community pharmacy or chemist<br>Online pharmacy (e-pharmacy)<br>Community health worker (e.g., home visit)<br>Other

11	If you were obtaining PrEP from an online pharmacy, you could have other products delivered to you with your PrEP medication. Please indicate if you would be interested in receiving any of the following with your PrEP delivery. *Select all that apply*	Pregnancy test kits<br>Contraception<br>Condoms<br>Sex lubricants<br>Sexual performance enhancing drugs (e.g., Vega 50)<br>HIV self-test<br>None of these products<br>Other _____________
12	If you were obtaining PrEP from an online pharmacy, how frequently would you prefer to refill your PrEP medication?	Every 1 month<br>Every 3 months<br>Every 6 months<br>Other _______
The next set of questions will ask about how much you would like to pay for different parts of online PrEP delivery. For each part, please provide the maximum price you are willing to pay. In answering these questions, please consider your usual expenses. Remember that there are no right or wrong answers. We're interested in your preference. You can write zero if you feel that most accurately reflects your preference.		
13	What is the maximum price you are willing to pay for a blood-based HIV self-test?	_________ KSH
14	What is the maximum price you are willing to pay for an oral HIV self-test?	_________ KSH
15	What is the maximum price you are willing to pay for remote/online clinical consultation to obtain a prescription for PrEP based on your HIV self-test results (one-time cost)?	_________ KSH
16	What is the maximum price you are willing to pay for a one-month supply of PrEP medicines?	_________ KSH
17	What is the maximum price you are willing to pay for delivery of PrEP medicines to a setting of your choice (one-time courier fee)?	_________ KSH
18	What is the maximum price you are willing to pay in total for the package of services associated online PrEP delivery (this includes courier delivery of an HIV self-test, a remote clinical consultation, and courier-delivered PrEP)?	_________ KSH
19	How would you most prefer to pay for the package of online PrEP services (e.g., HIV testing, remote consultation, PrEP medication delivery)?	Pay by piece (e.g., pay separately for HIV testing, the remote clinic visit, and PrEP drug delivery)<br>Pay per PrEP visit (including HIV testing, the remote visit, and PrEP medication delivery)<br>Pay a monthly subscription
The next set of questions ask about your preferences for online PrEP delivery		
20	What support, if any, would you be interested in from the online pharmacy provider to help you consistently take your PrEP medication? (select all that apply)	SMS reminders<br>Emails<br>Phone calls<br>Video calls<br>No support needed
21	At what frequency, would you like support for taking your PrEP medication?	Daily<br>Weekly<br>Bi-weekly (every other week)<br>Monthly
22	Have you heard about HIV self-testing as a method for testing for HIV before today?	Yes<br>No<br>Prefer not to answer

23	Have you ever used a self-test to test for HIV?	Yes<br>No<br>Prefer not to answer
24	How much would you like or dislike obtaining PrEP though an online platform?	Strongly dislike<br>Somewhat dislike<br>No opinion<br>Somewhat like<br>Strongly like
25	How much effort do you think it would take you to obtain PrEP online?	No effort<br>A little effort<br>A moderate effort<br>A huge amount of effort<br>No opinion
26	How confident do you feel about your ability to navigate an online website to obtain PrEP?	Very unconfident<br>Somewhat unconfident<br>No opinion<br>Somewhat confident<br>Very confident
27	How much do you agree or disagree with the following statement: Online PrEP delivery would help reduce HIV in my community.	Strongly disagree<br>Somewhat disagree<br>No opinion<br>Somewhat agree<br>Strongly agree
28	Which media sources do you most frequently use to get general information; such as daily news, important events, etc.	Newspapers<br>Radio<br>Television<br>WhatsApp groups<br>Social networks (e.g., Facebook, Instagram, Tik Tok)<br>Internet browsing (e.g., google, news' websites)
29	Which media sources do you most frequently use to get healthcare information; such as healthcare campaigns, interventions, new medicines, etc.	Newspapers<br>Radio<br>Television<br>WhatsApp groups<br>Social networks (e.g., Facebook, Instagram, Tik Tok)<br>Internet browsing (e.g., google, news' websites)

Part 7: Participant Demographics

30	In what region do you live?	North Eastern<br>Nyanza<br>Western<br>Rift Valley<br>Nairobi<br>Central<br>Coast<br>Eastern
31	What is your gender?	Male<br>Female<br>Other
32	Are you currently enrolled in school?	Yes<br>No
33	What is the highest level of education you have completed?	Primary<br>Secondary<br>O Levels

		A Levels<br>Technical or vocational school<br>University or higher<br>Prefer not to answer
34	Do you have regular employment?	Yes, I work full time<br>Yes, I work part time<br>Yes, I work multiple jobs<br>Yes, seasonal employment<br>No, I do not work<br>Prefer not to answer
35	How much money do you usually earn in a month?	|__|__|__|__|__|__| KES<br>Prefer not to answer
36	Have you ever been married?	Yes<br>No
37	What is your current relationship status?	Married<br>Steady boyfriend or girlfriend<br>Single<br>Widowed<br>Divorced or separated<br>Prefer not to answer
38	Have you ever been pregnant? *[If Q31 = female]*	Yes<br>No
39	Are you using any family planning method to delay or avoid pregnancy?<br>*[If Q31 = female]*	Yes<br>No<br>Prefer not to answer
40	What method(s) are you using? *[If Q39 = Yes]*	Oral pills<br>IUD<br>Implants<br>Injections<br>Condoms<br>Withdrawal<br>Rhythm or periodic abstinence<br>Emergency contraception<br>Tubal ligation/hysterectomy<br>Vasectomy<br>Traditional methods<br>Don't know<br>Prefer not to answer<br>Other ________________
41	Where do you prefer to access these family planning methods? *[If Q39 = Yes]*	Community pharmacy/chemist<br>Online pharmacy<br>Health facility<br>Friend or family member<br>Sexual partner(s)<br>Other ________________

Part 8: Sexual Behavior

42	<u>In the last 3 months</u>, have you had a primary sex partner? (A primary sex partner is a person you have sex with on a regular basis, or someone you consider to be your main partner.)	Yes<br>No<br>Prefer not to answer
43	How long have you been with your primary partner? *[If Q42 = Yes]*	|__|__| years |__|__| months

44	In the past 3 months, have you had sex?	Yes<br>No
45	In the past 3 months, what types of sexual partners have you had? *[If Q44 = Yes]*	One primary partner only (no other partners)<br>One primary partner and casual partner(s)<br>Casual partner(s) only<br>Other ___________<br>Prefer not to answer
46	In the past 3 months, how many different people have you had sex with? *[If Q44 = Yes]*	|__|__|__| people<br>888 = Don't know<br>999 = Prefer not to answer
47	In the past 3 months, how many of these people were new sexual partners (i.e., you had never had sex with them before)? *[If Q44 = Yes]*	|__|__|__| people<br>888 = Don't know<br>999 = Prefer not to answer
48	In the past month, how many times did you have sex?	|__|__|__| times
49	When you had sex in the past month, how many times did you use a condom? *[If Q47 > 0]*	|__|__|__| times
The next set of questions ask about your sexual behavior in the past 6 months		
50	In the past 6 months, have you had sex without a condom?	☐ *Yes* ☐ *No* ☐ *Unsure*
51	In the past 6 months, do you think you may have been exposed to HIV?<br>*For example, you might select "yes" if, during the past 6 months, you:*<br>• *had a condom break*<br>• *shared needles, syringes, or other equipment to inject drugs, or*<br>• *were sexually assaulted*	☐ *Yes* ☐ *No* ☐ *Unsure*
52	In the past 6 months, have you had sex with more than one person?	☐ *Yes* ☐ *No* ☐ *Unsure*
53	Do you have any sex partners who are HIV-positive and:<br>• are not currently taking antiretroviral therapy (ART),<br>• have been taking antiretroviral therapy (ART) for less than 6 months,<br>• have been missing doses of their antiretroviral therapy (ART),<br>• have a detectable HIV viral load, or<br>• the two of you are trying to get pregnant?<br>*Select "yes" if ANY of the above statements are true about any of your sex partners who are HIV-positive.*	☐ *Yes* ☐ *No* ☐ *Unsure*

54	Do you have any sex partner(s) who you think are at high risk for HIV and whose HIV status you do not know?<br>*Some example behaviors that may make your partner at high risk for HIV include:*<br>• *if he or she has other partners whose HIV status is unknown*<br>• *if he or she exchanges sex for money/gifts or money/gifts for sex*<br>• *if he or she engages in intravenous drug use*	☐ *Yes* ☐ *No* ☐ *Unsure*
55	In the past 6 months, have you used post-exposure prophylaxis (PEP) two times or more?<br>*Post-exposure prophylaxis is a medication that is taken very soon after a possible exposure to HIV to prevent becoming HIV-positive.*	☐ *Yes* ☐ *No* ☐ *Unsure*
56	In the past 6 months, have you been diagnosed with or treated for a sexually transmitted infection (STI)?<br>*Examples of sexually transmitted infections include chlamydia and gonorrhea.*	☐ *Yes* ☐ *No* ☐ *Unsure*
57	In the past 6 months, have you had sex while under the influence of drugs or alcohol?	☐ *Yes* ☐ *No* ☐ *Unsure*
58	In the past 6 months, have you been forced to have sex against your will or physically assaulted, including assault by your sex partner?	☐ *Yes* ☐ *No* ☐ *Unsure*
59	In the past 6 months, have you had sex with someone in exchange for money or a gift?	☐ *Yes* ☐ *No* ☐ *Unsure*
60	In the past 6 months, have you given someone money or a gift in exchange for sex?	☐ *Yes* ☐ *No* ☐ *Unsure*
61	In the past 6 months, have you shared needles with anyone while engaging in intravenous drug use?	☐ *Yes* ☐ *No* ☐ *Unsure*

The questionnaire ends here. Thank you for your responses.
