# Supplementary material for: Assessing preferences for HIV pre-exposure prophylaxis (PrEP) delivery services via online pharmacies in Kenya: protocol for a discrete choice experiment
Source: BMJ Open. 2023 Apr 3;13(4):e069195. doi: 10.1136/bmjopen-2022-069195 (PMC10083853; doi:10.1136/bmjopen-2022-069195)
Supplement: Supplementary data [file bmjopen-2022-069195supp004.pdf]

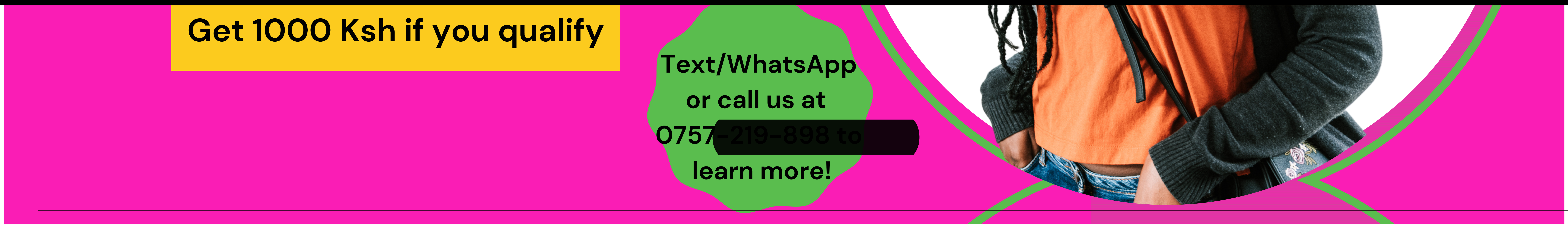
Get 1000 Ksh if you qualify
Text/WhatsApp
or call us at
0757[redacted]
learn more!

BMJ Publishing Group Limited (BMJ) disclaims all liability and responsibility arising from any reliance placed on this supplemental material which has been supplied by the author(s)

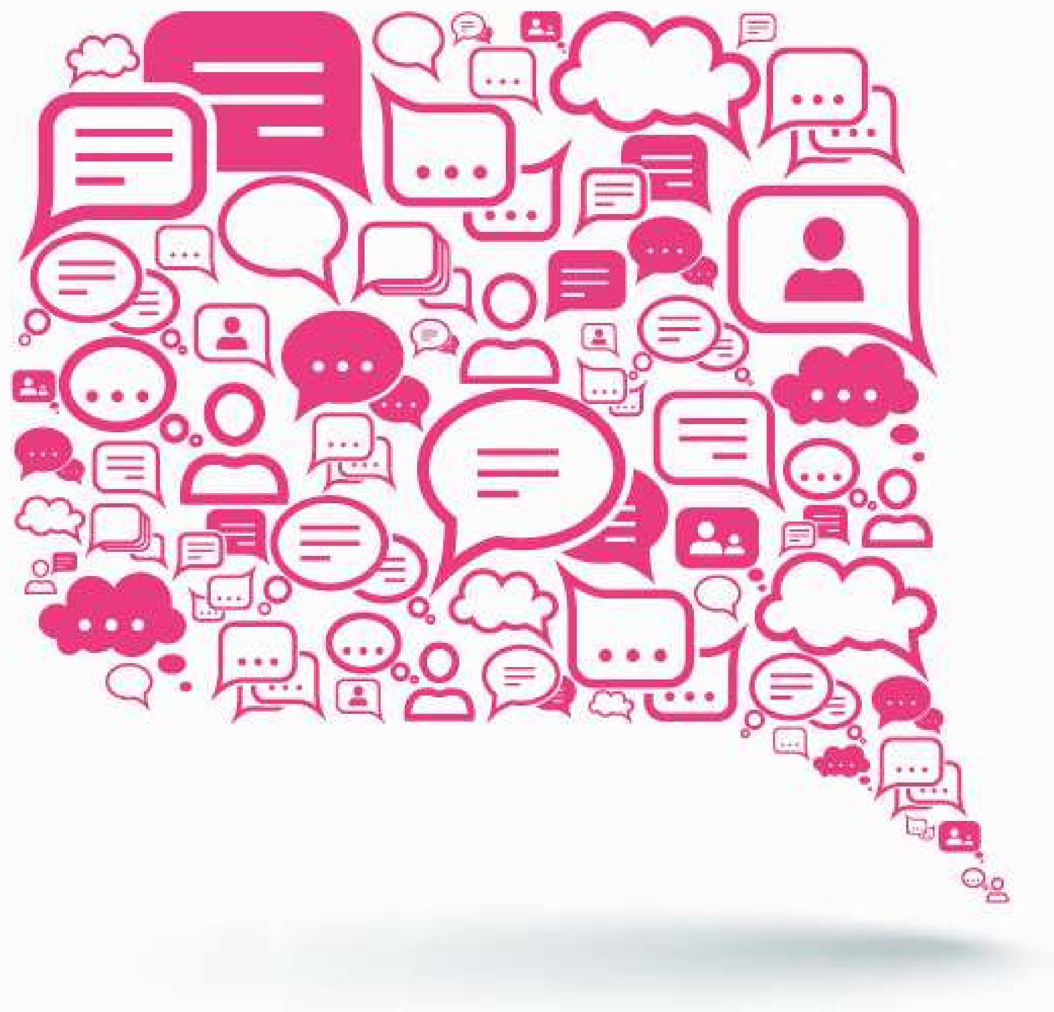

We are doing a survey about **PrEP service delivery.**
PrEP is a pill that you can take daily to reduce your risk of getting HIV.

We would like to understand what you think about getting PrEP delivered to you from an online pharmacy, like **MYDAWA.**

Hearing about your **preferences** will help us design new models of **PrEP delivery** for your community.

If you are interested, we will meet you in a **location of your choice** to complete the survey.
The survey is confidential and takes about **45 minutes.** You will **receive 1,000 Ksh** for your time.

If you are interested in taking part in the survey or have any questions, please send a message via **text/WhatsApp or call: 0757-219-898**
