# Supplementary material for: Assessing preferences for HIV pre-exposure prophylaxis (PrEP) delivery services via online pharmacies in Kenya: protocol for a discrete choice experiment
Source: BMJ Open. 2023 Apr 3;13(4):e069195. doi: 10.1136/bmjopen-2022-069195 (PMC10083853; doi:10.1136/bmjopen-2022-069195)
Supplement: Supplementary data [file bmjopen-2022-069195supp005.pdf]

Supplement 5: DCE Pre-screening Script

DCE RECRUITMENT SCRIPT

Hello, my name is…I'm a Research Assistant at KEMRI and we are doing a study to learn about what people in your community think about pre-exposure or PrEP. PrEP is a pill that you can take daily to reduce your risk of getting HIV.

We would like to learn about your preferences for getting them delivered to you from an online pharmacy. This means a pharmacy will deliver drugs to clients using a courier, so they do not need to travel to a pharmacy. Hearing about your preferences will help us design new models of PrEP delivery for your community.

If you are interested, we will meet you in a location of your choice to complete the survey. The survey is confidential and takes about 60 minutes. You will receive Ksh 1000 for your time and effort.

Are you interested in taking part in this survey?

[IF NO] Thank you very much and have a good [morning/afternoon evening]

[IF YES] That's great! I'm now going to ask you questions to make sure that you are eligible to be in this study. This will take about 10 minutes. If you are eligible, I'll schedule a time for someone from our research team to come to a place that is convenient for you, so you can complete the survey.

Before we begin, do you have any questions?

[Verifying Source of information] Thank you, tell me how you got to know about us?

[If they do not know, advise them on how they can access the study information on the MyDawa website]

[If the participant knows about PrEP study, go ahead and start the pre-screening process]

[Pre-screening process]

[What is your age?] If below the age of 18, inform the participant they are ineligible and explain the reasons why.

[What was the result of your last HIV test?] If positive, inform the participant they are ineligible for the survey.

Are you currently taking PrEP to prevent HIV?

[If eligible for the study participation. Please continue with scheduling their study visit] Thank you for responding to the questions. You are eligible to participate in the study. I would like to ask you for some information on how I can contact you and when and where we can schedule the study.

[Write down the first name and surname]

[Write done the phone number where we can reach them and how they would prefer to be contacted] Through a phone call, SMS, or WhatsApp.

[Which day of the week are you available for the interview] Write down the day given by the participant.

[Time of scheduled study visit] Someone from our research team will come to a location of your choice to administer the study survey. Where would you like to meet?

[Enter study visit] Inform the study participant of the need for privacy, security, and confidentiality.

Thank you so much for your time and for agreeing to participate. Someone from our research team will contact you on the day of the survey for final arrangements. If you have any questions before the day of the interview you can reach the study through our contact number.
