# Supplementary material for: Assessing preferences for HIV pre-exposure prophylaxis (PrEP) delivery services via online pharmacies in Kenya: protocol for a discrete choice experiment
Source: BMJ Open. 2023 Apr 3;13(4):e069195. doi: 10.1136/bmjopen-2022-069195 (PMC10083853; doi:10.1136/bmjopen-2022-069195)
Supplement: Supplementary data [file bmjopen-2022-069195supp006.pdf]

Supplement 6: DCE Data Management R Codes

```
---
title: "DCE Data Check - R Script"
date: "`r Sys.Date()`"
output:
  html_document:
    toc: true
    toc_float: true
---

# Load dataset
rm(list=ls())

# Set up R environment
## install.packages("stringr", "tidyverse", "dplyr", "pander")
library(stringr)
library(tidyverse)
library(dplyr)
library(pander)

# Set up functions
`%notin%` <- Negate(`%in%`)
table_na <- function(x){table(x, useNA = "always")}

# **NOTE**: Change my_path to your own directory
## my_path = "C:/Users/Yilin Chen/Desktop/2021 DCE ePharm/Data collection"
my_path = "~/Desktop/My projects/2021 DCE ePharm/Data collection" # Change this to the
path of your folder
setwd(my_path)

# Load data
## **NOTE**: Please change the dataset name accordingly
file_name = "GatesePharmDCESurvey_data_pre-launch_5.5.csv" # Change file_name to the
name of csv file
dat <- read.csv(file_name)
dat$x = seq(nrow(dat))

# Select relevant survey records in the data collection time window
dat$day <- str_split_fixed(dat$sys_StartTime, " ", 4)[,1]
dat$month <- str_split_fixed(dat$sys_StartTime, " ", 4)[,2]
dat$year <- str_split_fixed(dat$sys_StartTime, " ", 4)[,3]
dat$date <- as.Date(with(dat,paste(year,month,day,sep="-")),"%Y-%b-%d")
```

```
## **NOTE**: Please change the date_start and date_end below for specific data collection time window
date_start = "2022-03-09" # Change this to the start of time window
date_end = "2022-04-26"   # Change this to the end of time window
dat2 <- dat %>% filter(date >= date_start & date <= date_end) # example: from 2022-03-09 to 2022-04-26

# Select relevant survey records that contain "230" in the PTID
## **NOTE**: Please revise "230" to the starting number/word used in the actual survey
num_start = "230" # Change this to the actual starting number/word
dat2 <- dat2[str_detect(dat2$PTID1, num_start), ]
dat2 <- dat2[complete.cases(dat2[ , c('PTID1')]), ]
nrow(dat2)

# Data checks
## 1. Check for number of interviews

######################################
# 1. Check for number of interviews #
######################################

# Show PTIDs - first check if there are any missing PTIDs
unique(dat2$PTID1)

# Count the total number of interviews
length(unique(dat2$PTID1))

# Count the total number of interviews by RAs
dat2 %>% group_by(RAInitials) %>% tally() %>% pander()

## 2. Check for duplicate PTIDs and incomplete surveys

#########################################################
# 2. Check for duplicate PTIDs and incomplete surveys #
#########################################################

# Lists all duplicate records using the variable PTID1 and print out these PTIDs
count_pid <- as.data.frame(table(dat2$PTID1))
dup_pid <- as.character(count_pid$Var1[(count_pid$Freq > 1)])
dup_pid

# Create a subset with duplicate PTIDs
dat3a <- dat2 %>% filter(PTID1 %in% dup_pid) %>% select(sys_RespNum, sys_StartTime, sys_EndTime, sys_LastQuestion, RAInitials, PTID1, PTID2)
```

```
dat3a <- dat3a[order(dat3a$PTID1), ]

# Incomplete surveys: show PTIDs when the last answered question is not
"TeminateQuestionnaire" or "TerminateEligibility"
incomplete_id <- dat2 %>%
  filter(sys_LastQuestion %notin% c("TerminateQuestionnaire", "TerminateEligibility")) %>%
  select(x)

# List PTIDs of incomplete surveys
dat3 <- dat2 %>% filter(x %in% incomplete_id$x)
incomplete_pid <- unique(dat3$PTID1)
incomplete_pid

# Create a subset with incomplete surveys
dat3b <- dat3 %>% select(sys_RespNum, sys_StartTime, sys_EndTime, sys_LastQuestion,
RAInitials, PTID1, PTID2)
dat3b <- dat3b[order(dat3b$PTID1), ]

# Combine incomplete records and duplicate records
dat3c <- dat3a %>% full_join(dat3b, by = c("sys_RespNum", "sys_StartTime", "sys_EndTime",
"sys_LastQuestion", "RAInitials", "PTID1", "PTID2"))
dat3c$status = ifelse(dat3c$sys_LastQuestion %notin% c("TerminateQuestionnaire",
"TerminateEligibility"), "incomplete", "complete")
dat3c

# Save duplicate surveys to a csv file and add a date to the file name
## Most of duplicate surveys might be due to incompleteness
## **NOTE**: Change the date_now below to the current date
date_now = "05.12.2022" # Change to the current date
write.csv(dat3c, paste0("GatesePharmDCESurvey_duplicate_", date_now, ".csv"))

# You could also review these duplicate records on Sawtooth or original csv file, and figure out
reasons for duplicates
## Admin site link: https://ePharmacyPrEP.sawtoothsoftware.com/english/admin.html


## 3. Check for specific responses
### Part 1: Eligibility Assessment

####################################
# 3. Check for specific responses #
####################################

# Create a subset of eligible participants and interested variables for exploratory analysis
```

```
dat4 <- dat2 %>% filter(sys_LastQuestion == "TerminateQuestionnaire")
length(unique(dat4$PTID1)) # check number of unique participants
dat5 <- dat4[, 19:156] # check if this includes all variables of interest (starting from "RA initials"
variable)

# Part 1: Eligibility Assessment (Q1-4)
apply(dat5[, 4:8], 2, table_na)

## AgeYears: should be >= 18 years old
dat5$PTID1[dat5$AgeYears < 18]
## HIVTestDate_Month: should not be NA and should be numeric from 1-12
dat5$PTID1[is.na(dat5$HIVTestDate_Month)]
## HIVTestDate_Year: should not be NA and should be year
dat5$PTID1[is.na(dat5$HIVTestDate_Year)]
## HIVTestResult: should not include 2 - "Positive"
dat5$PTID1[dat5$HIVTestResult == 2]

### Part 2: PrEP Knowledge and Interest

# Part 2: PrEP Knowledge and Interest (Q5-7)
apply(dat5[, 9:14], 2, table_na)

### Part 3: Discrete Choice Experiment

# Part 3: Discrete Choice Experiment
apply(dat5[, 15:30], 2, table_na) # shouldn't have any NAs

### Part 6: E-Pharmacy Engagement and HIV Self-Testing

# Part 6: E-Pharmacy Engagement and HIV Self-Testing (Q8-29)
## e-Pharmacy engagement
apply(dat5[, 31:53], 2, table_na)

## ePharmEngDetail_7: if "Other" category = 1,  there should be text answers
table(dat5$ePharmEngDetail_7)
## PreferObtainPrEP_5_other: if PreferObtainPrEP == 5, there should be text answers
table(dat5$PreferObtainPrEP_5_other[dat5$PreferObtainPrEP == 5])
## AddlProducts_7: if "Other" category = 1,  there should be text answers
table(dat5$AddlProducts_7)
## PrEPRefillPref_4_other: if PrEPRefillPref == 4, there should be text answers
table(dat5$PrEPRefillPref_4_other[dat5$PrEPRefillPref == 4])

## WTP questions
apply(dat5[, 54:60], 2, table_na)
```

```
## Preferences for online PrEP delivery
apply(dat5[, 61:84], 2, table_na)

### Part 7: Participant Demographics

# Part 7: Participant Demographics (Q30-41)
apply(dat5[, 85:112], 2, table_na)

## FamPlanMethod_14_other: if FamPlanMethod_14 == 1, there should be text answers
table(dat5$FamPlanMethod_14_other[dat5$FamPlanMethod_14 == 1])
## AccessFamPlan_6_other: if AccessFamPlan == 6, there should be text answers
table(dat5$AccessFamPlan_6_other[dat5$AccessFamPlan == 6])

### Part 8: Sexual Behaviors

# Part 8: Sexual Behaviors (Q42-61)
apply(dat5[, 113:138], 2, table_na)

## SexPartType3Mo_4_other: if SexPartType3Mo == 4, there should be text answers
table(dat5$SexPartType3Mo_4_other[dat5$SexPartType3Mo == 4])
```
