# Supplementary material for: Assessing preferences for HIV pre-exposure prophylaxis (PrEP) delivery services via online pharmacies in Kenya: protocol for a discrete choice experiment
Source: BMJ Open. 2023 Apr 3;13(4):e069195. doi: 10.1136/bmjopen-2022-069195 (PMC10083853; doi:10.1136/bmjopen-2022-069195)
Supplement: Author's manuscript [file bmjopen-2022-069195.draft_revisions.pdf]

BMJ Open is committed to open peer review. As part of this commitment we make the peer review history of every article we publish publicly available.

When an article is published we post the peer reviewers' comments and the authors' responses online. We also post the versions of the paper that were used during peer review. These are the versions that the peer review comments apply to.

The versions of the paper that follow are the versions that were submitted during the peer review process. They are not the versions of record or the final published versions. They should not be cited or distributed as the published version of this manuscript.

BMJ Open is an open access journal and the full, final, typeset and author-corrected version of record of the manuscript is available on our site with no access controls, subscription charges or pay-per-view fees (http://bmjopen.bmj.com).

If you have any questions on BMJ Open's open peer review process please email info.bmjopen@bmj.com

BMJ Open

Assessing Preferences for Pre-exposure Prophylaxis (PrEP) Delivery Services via Online Pharmacies in Kenya: Protocol for a Discrete Choice Experiment

Journal:	*BMJ Open*
Manuscript ID	bmjopen-2022-069195
Article Type:	Protocol
Date Submitted by the Author:	13-Oct-2022

Complete List of Authors:	Chen, Yilin; University of Washington, The Comparative Health Outcomes, Policy, and Economics (CHOICE) Institute<br>Saldarriaga, Enrique; University of Washington, The Comparative Health Outcomes, Policy, and Economics (CHOICE) Institute<br>Montano, Michalina; Fred Hutchinson Cancer Research Center, Vaccine and Infectious Diseases Division (VIDD); University of Washington, Department of Global Health<br>Ngure, Kenneth; Jomo Kenyatta University of Agriculture and Technology, School of Public Health<br>Thuo, Nicholas; Partners in Health and Research Development<br>Kiptinness, Catherine; Partners in Health and Research Development<br>Rafferty, Maeve; MYDAWA<br>Terris-Prestholt, Fern; London School of Hygiene and Tropical Medicine Faculty of Public Health and Policy, Department of Global Health and Development<br>Stergachis, Andy; University of Washington, The Comparative Health Outcomes, Policy, and Economics (CHOICE) Institute; University of Washington, Department of Global Health<br>Mugambi, Melissa; University of Washington, Department of Global Health<br>Ortblad, Katrina; Fred Hutchinson Cancer Research Center, Public Health Science Division<br>Sharma , Monisha; University of Washington, Department of Global Health

Keywords:	HIV & AIDS < INFECTIOUS DISEASES, Health economics < HEALTH SERVICES ADMINISTRATION & MANAGEMENT, Public health < INFECTIOUS DISEASES, Telemedicine < BIOTECHNOLOGY & BIOINFORMATICS, International health services < HEALTH SERVICES ADMINISTRATION & MANAGEMENT, HEALTH SERVICES ADMINISTRATION & MANAGEMENT

SCHOLARONE™
Manuscripts

Assessing Preferences for Pre-exposure Prophylaxis (PrEP) Delivery Services via Online Pharmacies in Kenya: Protocol for a Discrete Choice Experiment

Yilin Chen[1], Enrique Saldarriaga[1], Michalina A. Montano[2,3], Kenneth Ngure[4], Nicholas Thuo[5], Catherine Kiptinness[5], Maeve Rafferty[6], Fern Terris-Prestholt[7], Andy Stergachis[1,3], Melissa Latigo Mugambi[3], Katrina F. Ortblad[8], Monisha Sharma[3]

[1]The Comparative Health Outcomes, Policy, and Economics (CHOICE) Institute, University of Washington, Seattle, USA

[2]Vaccine and Infectious Diseases Division (VIDD), Fred Hutchinson Cancer Center, Seattle, USA

[3]Department of Global Health, University of Washington, Seattle, USA

[4] School of Public Health, Jomo Kenyatta University of Agriculture and Technology

[5]Partners in Health and Research Development, Thika, Kenya

[6]MYDAWA, Nairobi, Kenya

[7]Department of Global Health and Development, Faculty of Public Health and Policy, London School of Hygiene and Tropical Medicine, London, UK

[8]Public Health Science Division, Fred Hutchinson Cancer Center, Seattle, USA

Corresponding author:
Yilin Chen, ylchen95@uw.edu
Phone: +1 206-618-8466
Health Sciences Building, 1956 NE Pacific St H362, Seattle, WA 98195

For peer review only - http://bmjopen.bmj.com/site/about/guidelines.xhtml

Keywords: PrEP, HIV prevention, mHealth, Discrete Choice Experiment, pharmacies, online delivery, preferences

Word count: 3704; **Abstract:** 300; **Tables:** 1; **Figures:** 2; **Supplement files:** 7.

For peer review only

For peer review only - http://bmjopen.bmj.com/site/about/guidelines.xhtml

ABSTRACT

Introduction: Pre-exposure prophylaxis (PrEP) is highly effective at preventing HIV acquisition, but coverage remains low in high prevalence settings. Initiating and continuing PrEP via online pharmacies is a promising strategy to expand PrEP uptake but little is known about user preferences for this strategy. We describe methods for a discrete choice experiment (DCE) to assess preferences for PrEP delivery from an online pharmacy.

Methods and analysis: This cross-sectional study is conducted in Nairobi, Kenya, in partnership with MYDAWA, a private online pharmacy retailer with a planned sample size of >400 participants. Eligibility criteria are: ≥18 years, not known HIV-positive, and are interested in PrEP. Initial DCE attributes and levels were developed via literature review and stakeholder meetings. We conducted cognitive interviews to assess participant understanding of the DCE survey and refined the design. The final DCE used a D-efficient design and contained four attributes: PrEP eligibility assessment, HIV test type, clinical consultation type, and user support options. Participants are presented with 8 scenarios consisting of two hypothetical PrEP delivery services. The survey was piloted among 20 participants before being advertised on the MYDAWA website on pages displaying products indicating HIV risk (e.g. emergency contraception). Interested participants call a study number and those screened eligible meet a research assistant in a convenient location to complete the survey. The DCE will be analyzed using a conditional logit model to assess average preferences and mixed logit and latent class models to evaluate preference heterogeneity among subgroups.

Ethics and dissemination: This study was approved by the University of the Washington Human Research Ethics Committee, the Kenya Medical Research Institute, Nairobi County, and the Scientific and Ethics Review Unit in Kenya. We will share findings through peer-reviewed

For peer review only - http://bmjopen.bmj.com/site/about/guidelines.xhtml

publications, international conferences, and study reports. We will hold meetings with local and international stakeholders to disseminate results.

Strengths and limitations of this study:

- The study uses a discrete choice experiment (DCE) to elicit the user preferences on initiating and continuing pre-exposure prophylaxis (PrEP) from an online pharmacy in Kenya.
- DCE results will provide a quantitative assessment of how users value different characteristic of an online PrEP delivery model; this can inform the design of online HIV PrEP delivery programs to optimize uptake.
- We are collecting information on the willingness-to-pay for each component of ePrEP services which can inform policy decisions and resource allocation.
- DCE scenarios are hypothetical and individuals' stated preferences may not represent their actual choices in real world settings. Additionally, participants are recruited from Nairobi, Kenya and their preferences may not generalize to other regions. Finally, online PrEP delivery requires access to internet and ability to pay for HIV self-testing and PrEP delivery services. This platform can reach the growing middle class in Kenya but is not accessible to those with low socioeconomic status.

For peer review only - http://bmjopen.bmj.com/site/about/guidelines.xhtml

INTRODUCTION

Kenya has one of the largest HIV epidemics globally, with a national prevalence of 4.2%.[1-3] Despite significant strides in HIV prevention, approximately 32,000 new HIV infections occurred in Kenya in 2021.[4] Pre-exposure prophylaxis (PrEP) is highly effective at preventing HIV acquisition and is recommended by the World Health Organization (WHO) and the Kenya Ministry of Health for persons at risk of HIV.[5] However, PrEP coverage remains low among those with risk indications.[6 7] Currently, PrEP is mainly delivered through health care facilities and barriers to widespread use include long wait times and travel distance, limited hours of operation, privacy concerns, stigma, and understaffing.[8 9] Additionally, PrEP demonstration projects in East Africa have found low PrEP retention among those who initiate PrEP at clinics, due in part to challenges returning to the clinic for frequent continuation visits.[10 11]

Initiating and continuing PrEP online is a novel approach that could expand PrEP coverage to individuals not accessing clinic-based PrEP and improve PrEP continuation.[12] Delivery of PrEP services closer to the client can reduce opportunity costs associated with clinic-based PrEP provision. A growing telehealth ecosystem in sub-Saharan Africa, recently expanded due to the COVID-19 pandemic, provides private and convenient options for remote consultation, purchasing, and delivery of medications to patients.[13-15] Coupled with Kenya's national commitment to PrEP roll-out, online PrEP delivery is a promising strategy to expand PrEP access. Our team is currently conducting a pilot study of the first online care model for PrEP delivery (NCT05377138) through collaboration with Kenya’s first e-pharmacy retailer MYDAWA.[16]

Successful PrEP scale up requires an understanding of user preferences to tailor services to optimize uptake, adherence, and retention. There are several potential options for structuring

For peer review only - http://bmjopen.bmj.com/site/about/guidelines.xhtml

PrEP delivery services *via* an online pharmacy including the use of different HIV testing modalities and user support options. As online PrEP delivery is not yet available in Kenya, user preferences for components of this service are unknown. Discrete Choice Experiments (DCEs) are an ideal method for assessing user preferences in cases when there is no observed behavior. With a foundation in economic theory, DCEs assume that individuals choose the service that maximizes their expected benefit.[17] DCEs evaluate how individuals value selected features of a service by repeatedly asking them to choose between different sets of hypothetical alternatives. Resulting data can provide a quantitative assessment of user preferences including the relative importance of each attribute and likelihood of service uptake, which can help inform policy decisions and resource allocation. DCEs have been applied to a range of health policy decisions and are increasingly used to assess preferences for PrEP services. Prior DCEs assessing PrEP have examined user preferences for dosing regimen, type of PrEP products, side effects, cost, dispensing venue, and support services. [18-23] However, this is the first study to assess user preferences for ePrEP delivery.

Here we describe the design and methods from our ongoing DCE to assess user preferences for PrEP delivery from an online pharmacy in Kenya. This study adds to the small but growing literature of DCE protocols for HIV prevention[24-28] and additionally provides detailed information on DCE attribute selection and refinement, materials used for staff training, and methods for participant recruitment. The methods described along with slide decks of training materials and survey guides can assist other researchers interested in designing DCEs for HIV prevention in Africa. The results of this DCE can inform the design of online PrEP delivery models to increase PrEP uptake among persons at risk of HIV.

For peer review only - http://bmjopen.bmj.com/site/about/guidelines.xhtml

METHODS

Setting and Participants

This study is being conducted in Nairobi, Kenya, in partnership with MYDAWA, Kenya's first licensed online pharmacy.[29 30] MYDAWA's online platform provides affordable access to wellness projects, prescription and over-the-counter medicines delivered directly to the consumer, including products related to sexual health (e.g. HIV self-test kits and emergency contraception). The majority of MYDAWA's customer base is located in Nairobi.[16 29] The target population for our study is selected to closely mirror persons who would likely be clients of ePrEP delivery. Inclusion criteria are: age ≥18 years, not known to be HIV-positive, interested in PrEP, and able and willing to provide informed consent.

Patient and Public Involvement

We developed the care pathway for online PrEP service delivery in collaboration with Kenyan stakeholders, including members from the Ministry of Health, implementing organizations, researchers, professional organizations. We refined list of attributes and levels via stakeholder discussions with individuals in Kenya with expertise in HIV prevention and PrEP delivery and representatives from MYDAWA. Finally, we plan to share study findings with both local and international audiences at conferences. We will disseminate results at engagement meetings with key informants including Kenya's National AIDS and STIs Control Programme (NASCOP), patients groups, service providers, and other stakeholders.

Care pathway for online PrEP service delivery

For peer review only - http://bmjopen.bmj.com/site/about/guidelines.xhtml

We collaboratively developed a care pathway for online PrEP service delivery, based on an ongoing model of pharmacy-based PrEP service delivery in Kenya.[31] The core components of the online PrEP service delivery care pathway include: 1) PrEP eligibility assessment: This assessment is based on Kenya's National AIDS and STI Control Programme (NASCOP) PrEP Rapid Assessment Screening Tool (RAST), routinely used at public clinics in Kenya to assess HIV risk and PrEP eligibility. 2) HIV testing: Individuals who are potentially eligible for PrEP will complete an HIV test to ensure they are HIV-negative prior to starting PrEP; 3) Clinical consultation and PrEP prescribing: Individuals confirmed to be HIV-negative will undergo a medical risk assessment with a clinical officer to ensure PrEP is safe for them and receive a PrEP prescription; 4) PrEP delivery: A one-month supply of PrEP will be delivered to a setting of the client's choice at initiation. For all future refills, clients will receive a 3-month PrEP supply, consistent with the guidelines for PrEP dispensing at public clinics in Kenya. Clients will be asked to complete the HIV testing process described above prior to each refill visit; and 5) PrEP support: Individuals can to opt-in to receive ongoing support to answer questions about PrEP use and to report concerns and side effects. Each step in the care pathway for online PrEP delivery can be optimized to align with user preferences and was evaluated for inclusion in the final DCE.

Overview of DCE approach

DCE participants are presented with a series of scenarios consisting of two or more hypothetical services with different characteristics "attributes" that have different variations or "levels" and are asked to choose their preferred services. Surveys generally contain 7-10 scenarios with varying levels for each attribute. The experimental design determines the scenario pairs and the series of questions presented to participants. Analysis of DCEs can quantify the

For peer review only - http://bmjopen.bmj.com/site/about/guidelines.xhtml

impact of each aspect of a service on participants' preference and assess the trade-offs they are willing to make to obtain their preferred service.

Design and administration of the DCE is divided into several stages (Figure 1): 1) formative stage, 2) refinement stage, 3) experimental design, 4) survey administration, and 5) data analysis and dissemination. First, in the formative stage, we selected attributes and levels for the DCE and developed survey questions to accompany the DCE. Second, in the refinement stage, we conducted cognitive interviews to assess participant understanding of the hypothetical scenarios and associated illustrations (See Supplement 1 cognitive interview guide and Supplement 2 for staff training materials). Then we refined our DCE attributes and levels based on participant feedback. In the third stage, we developed the experimental design using a D-optimal design with 8 choice tasks per participant. Our study design followed the ISPOR Good Research Practice for Conjoint Analysis.[32] We then piloted the DCE to estimate the time needed to complete the questionnaire and assess participant fatigue. The survey administration adopts a hybrid approach, including in-person administration by research assistants or self-administration by online participants to achieve sufficient sample size and reach our target population more efficiently. Finally, we will conduct data analysis to estimate average preferences of each attribute level and explore heterogeneity of responses.

Software

We are using Research Electronic Data Capture (REDCap) electronic data capture tools hosted at the University of Washington for screening and informed consent data.[33] The DCE questionnaire was programmed in Sawtooth Software's Lighthouse Studio modules for general questionnaires and choice-based conjoint scenario designs, and hosted on the Sawtooth servers.[34]

For peer review only - http://bmjopen.bmj.com/site/about/guidelines.xhtml

We contacted a Sawtooth representative and obtained the software at a discounted price. Screening and informed consent forms were constructed as separate, chained surveys in REDCap. Completion and submission of informed consent forms via REDCap directs online participants to the Sawtooth questionnaire via an external link for online participants. DCE questionnaires are administered via Sawtooth's offline data capture application for in-person participants.

For peer review only

DCE procedures

Stage I: Formative phase –selection of attributes and levels, development of data collection instruments, training

Collaborative attribute and level selection. We developed an initial list of attributes and levels based on the care pathways of the ePrEP delivery model and a review of the PrEP literature.[5 7 35-37] This list was refined during stakeholder discussions with individuals in Kenya with expertise in HIV prevention and PrEP delivery and representatives from MYDAWA. Five attributes were included in the formative list: PrEP eligibility assessment, HIV test type, clinical consultation, user support options, and cost of PrEP delivery. Each attribute consisted of 2-3 levels (Table 1).

Table 1. DCE attributes and levels

Attribute & Description	Levels	Illustrative image
**PrEP eligibility assessment**<br>Method for conducting client eligibility assessment for PrEP	Online self-assessment using screening questions (phone number in case of questions)	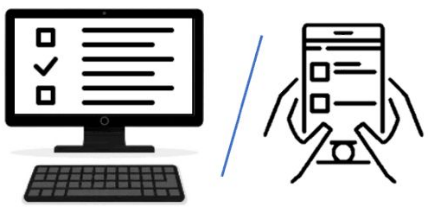

For peer review only - http://bmjopen.bmj.com/site/about/guidelines.xhtml

	Guided assessment with a remote clinical provider (via a phone call or WhatsApp)	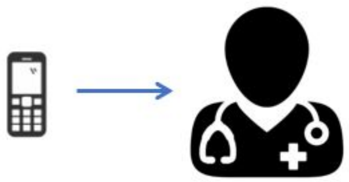
**HIV test type**<br><br>Type of HIV test delivered for PrEP initiation	Oral Fluid HIV self-test (at setting of your choice)	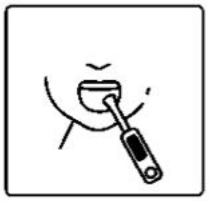
	Blood-based HIV self-test (at setting of your choice)	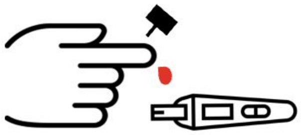
	Healthcare provider administers HIV Test at setting of your choice (blood-based)	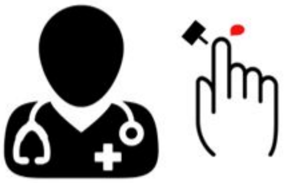
**Clinical consultation for prescribing PrEP**<br><br>Consultation needed to prescribe PrEP	Remote clinical consultation with provider (via a phone call or video chat)	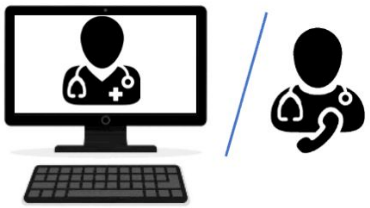
	In-person clinical consultation with provider after completing HIV test (at a setting of your choice)	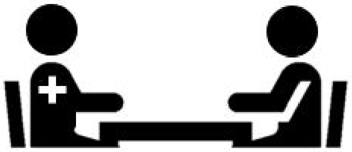
**User support options for PrEP**<br><br>Method for discussing your questions for PrEP with a healthcare provider	SMS	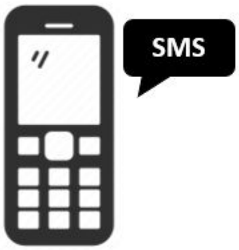

For peer review only - http://bmjopen.bmj.com/site/about/guidelines.xhtml

	Phone/video call	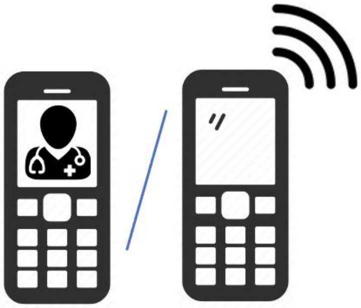
	Email	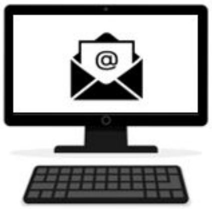

Abbreviations: PrEP, pre-exposure prophylaxis.

For peer review only

Questionnaire development. We developed a survey to accompany the DCE which included the following: PrEP eligibility assessment, PrEP interest and knowledge, online pharmacy and HIV testing interest, knowledge, and engagement, and participant demographics, and sexual behavior (Supplement 3 for survey questionnaire). We also assessed maximum willingness to pay (WTP) for different components of online PrEP delivery including oral vs blood-based HIV self-testing, remote clinical encounter, and delivery of PrEP drugs. We also asked about maximum WTP for the entire package of ePrEP delivery. The structure of the WTP questions was informed by a literature review.[38-40] The DCE itself consists of a brief overview of DCEs, definition of attributes and levels, and a series of choice tasks. For each DCE scenario, participants are first asked to choose their preferred service followed by an opt-out question: “Would you choose to get PrEP using this service if it were available?” (Figure 2).

Staff training. We conducted a training with study investigators, data mangers, and in-country research staff responsible for administering the survey; it was held over Zoom split over

For peer review only - http://bmjopen.bmj.com/site/about/guidelines.xhtml

two half day sessions to accommodate the time difference between Seattle and Kenya. The training consisted of presentations on the background of HIV in Kenya, the role of PrEP for HIV prevention, barriers to accessing PrEP via current delivery modalities and the potential ePrEP delivery to increase PrEP coverage. We explained the DCE administration procedures and instruments, including the REDCap pre-screening form and the process for scheduling in-person visits, REDCap e-consent forms, and the Sawtooth survey. On the second day, we discussed the process for conducting cognitive interviews and piloting and launching the DCE. Study staff practiced administering the cognitive interviews and DCE instruments in groups of two and provided feedback on study instruments. (See Supplement 2 for slide deck and training materials)

Stage II: Refinement of DCE attributes and levels, and cognitive interviews

DCE image design and cognitive interviews. We utilized images to pair with each level of DCE attributes to make the survey more engaging and understandable. Most of the images were adapted from the Noun Project, a free online image bank.[41] We chose simple images to minimize possibility of biasing participants. For example, images of healthcare providers were depicted with neutral expressions and their gender was not specified. In addition, images of fingerpicks for HIV testing scenarios were not portrayed with big needles or large amounts of blood (Supplement 4 for DCE images).

Cognitive interviews: We recruited individuals from the HIV research community in Kenya for cognitive interviews to assess the clarity of DCE images, DCE scenarios, and WTP questions (see Supplement 1 for cognitive interview guide). Participants were asked to interpret each attribute image and assess its accuracy in conveying the specified DCE level. They were

For peer review only - http://bmjopen.bmj.com/site/about/guidelines.xhtml

then presented with alternative images for each attribute and asked if any were a better match for the attribute description than the current image. The cognitive interviews also presented participants with two example DCE choice tasks to assess their understanding of the scenarios and ease completion. Additionally, we assessed participants' understanding of WTP questions for different aspects of the ePrEP delivery including HIV testing, clinical encounter, and delivery of PrEP drugs. We initially sought to audio-record the cognitive interviews but after consultation with the in-country staff, decided that it may hinder participants' ability to speak freely. Therefore, study staff took notes during the interviews.

Stage III: Experimental design and pilot testing

We used a D-efficient design for to construct the DCE.[32] The algorithm behind this design identifies the sub-set of the most meaningful comparisons across profiles from all possible choice tasks – 1,296 (i.e., $(2 \times 3 \times 2 \times 3)^2$ in our study.[42] The design enhances data efficiency and attempts to balance statistical power and sample size.[43 44] First, we considered the complete specification of the utility functions based on the chosen attributes and levels and multinomial logit as the analytical model.[42] We defined a main-effects utility function for each of the two alternatives in a choice task. The structure for the i-th utility function, U is defined as:

$$U_i = V(\beta, X_i) + \epsilon_i$$
$$V(\beta, X_i) = \beta_1 * X_1 + \ldots + \beta_k * X_k$$

Where $V(\beta, X_i)$ is the deterministic portion of the utility and a function of the k attribute-levels considered in the alternative; the β coefficients represent the average preference weight that the sample places on each attribute-level. The error term, ϵ_i, indicates the inability to perfectly measure the utility of each alternative, which is consistent with random utility theory. We did not use priors for the utility-function coefficients since none of the selected attributes had

For peer review only - http://bmjopen.bmj.com/site/about/guidelines.xhtml

a natural order (i.e., an ascending order example would be the attribute 'pain', because it is safe to assume that most people would prefer low pain rather that high pain).

Each scenario consists of a forced-choice task, followed by an opt-out question: Would you choose to get PrEP using this service if it were available? (Figure 2). [45] The goal of the opt-out option was to represent the status quo where participants can decide against uptake of a new product[46]. Participants who select "no" express their preference for the status quo. This format allows us to gathering data about user preferences while not drawing too much attention to the opt-out. Studies suggest potential for cognitive biases to preserve the status quo in 3-alternative choice tasks.[47 48]

Sample size: The DCE literature has not yet reached a consensus on the best method to estimate the sample size required in a DCE study to return meaningful, statistically robust parameter estimates.[49 50] Some experts suggest using a minimum sample size of 300.[49] Johnson and Orme provided another rule of thumb using an equation that considers the number of choice questions, alternative scenarios presented, and attributes and levels.[50 51] Additionally, the more tasks per participant, the smaller sample size required to achieve comparable levels of uncertainty in coefficients. During the formative phase, most participants stated that they could answer between 7 to 10 choice tasks before becoming fatigued.

A criticism to rules of thumb for DCE sample estimation is the lack of robustness to different product characteristics or consumers behavior.[52] Given this uncertainty, we implemented a scenario analysis considering all possible tasks per participant, from 6-10 under a sample size varying from 200-500 in 50 increments. For each scenario, we evaluated the standard error of coefficients and the D-efficiency measure for the overall design. We found that improvements were diminishing above a sample size of 350. Therefore, we chose a design with 8

For peer review only - http://bmjopen.bmj.com/site/about/guidelines.xhtml

choice tasks per participant and a sample size of 400, which had an expected standard deviation 0.049 per coefficient. This sample size was slightly higher than the improvement plateau to account for the possibility of preference heterogeneity in the sample while considering budgetary and logistic restrictions.[32 53]

Stage IV: Pilot testing, DCE recruitment, and administration

Pilot testing and survey refinement. After revising the questionnaire based on feedback from the cognitive interviews, we piloted the survey in two phases. First, we administered the questionnaire to individuals from the HIV research community in Kenya. Based on participants' feedback, we found the cost attribute seemed to be a strong driver of participant preferences, with many participants selecting the cheaper PrEP service regardless of other attributes. To avoid the possibility of having one attribute overwhelming participant decision making, we removed the cost attribute from the final version of the DCE and instead assessed cost preferences only through direct WTP questions included in the questionnaire (Table 1). Additionally, we moved the DCE scenario questions to the beginning of the questionnaire to minimize participant fatigue. Since several participants expressed hesitancy in answering sensitive sexual behavior questions at the beginning of the survey, we moved these questions to the end to provide increased opportunity for staff to develop rapport with participants; we also gave participants the option of self-administering these questions by taking the tablet and filling out this section on their own. Finally, participants found the DCE eligibility questions from the HIV Risk Assessment Tool to be invasive and too sensitive to answer over the phone, so we revised the pre-screening process to only ask if participants are be interested in obtaining PrEP. In the second phase, we piloted the

For peer review only - http://bmjopen.bmj.com/site/about/guidelines.xhtml

survey to 20 individuals recruited through the MYDAWA platform using study eligibility criteria. We assessed data quality, survey flow and time needed for survey administration.

Recruitment. Participants are recruited through banner ads on the MYDAWA online pharmacy website. To target persons at risk of HIV acquisition, only those who click on HIV self-test kits on the MYDAWA platform see the online recruitment banners. In addition, persons who purchase these items from MYDAWA are mailed a flyer describing the DCE with their purchases (See Supplement 5 for the recruitment banner). We are also planning to expand DCE recruitment by advertising on social media pages that provide information on HIV prevention as well as through MYDAWA call in center where persons can access support related to STI purchases.

Survey administration. Interested participants can voice call or send an SMS to the study phone number provided on recruitment materials. Study staff assess eligibility via a phone pre-screening and schedule time to meet participants at a convenient location to complete the survey (See Supplement 6 for the pre-screening script). Participants are reimbursed 1,000 KES (~$8.70 USD) for their time. The in-person data collection of this study was initiated on 1-June-2022.We plan to launch the online self-administered DCE after we finish data collection for the in-person component. For the online DCE, participants will follow the DCE link on the recruitment banner to an online screening tool, self-administered via REDCap survey. Once the screening survey is submitted, eligible participants will automatically be directed to complete online consent forms via REDCap, and subsequently the DCE questionnaire *via* Sawtooth.

Stage V: Data management and data analysis

For peer review only - http://bmjopen.bmj.com/site/about/guidelines.xhtml

Data are stored in a central location on the Sawtooth administration site, which allow users to directly examine survey responses or download a csv file for further analysis. We monitor data quality on a weekly basis, include checking total number of interviews, duplicate entries or incomplete surveys, and substantial missingness or outliers in responses (See Supplement 7 for R script used in data management).

To analyze the data, we will fit a conditional logit model with an alternative specific constant for the opt-out. The coefficients (i.e. preference weights) represent the average relative utility for each attribute-level, compared to the reference.[54 55] In addition, we will generate a ranking of importance across attributes calculated as the ratio of their preference weights over the preference weights of the attribute with the lowest impact in the decision-making process (i.e., the attribute with the lowest preference weights). This ranking will provide information about the relative importance of each attribute, and respondents' willingness to trade one attribute for another. We will conduct a two-part analysis for the main model: First, we will analyze only the forced-choice data (i.e., ignoring the opt-out question), and second, we will use a mixed logit regression model (i.e., random parameters logit) to account for preferences' heterogeneity in all coefficients. We will qualitatively assess if our conclusions are consistent in both analyses and compare the goodness of fit of the models using Akaike information criteria (AIC) and Bayesian information criteria (BIC).

Further, we will elicit preference heterogeneity by participant demographics and other variables of interest. Latent class analysis will be performed to examine variation in preference by using the data to identify groups of respondents with similar preferences.[56] We will fit to 2 to 10 classes and assess the goodness of fit of each one using McFaddens pseudo r-squared,[54] log likelihood test, and adjusted-AIC and -BIC. In the scenario where a covariate arises as an

For peer review only - http://bmjopen.bmj.com/site/about/guidelines.xhtml

important predictor of class membership, we will assess the need of treating it as an effect modifier and therefore presenting the results separately for each category of the covariate.

Descriptive analysis will be conducted to summarize participants' demographic characteristics, sexual behaviors, WTP for online PrEP services, and online pharmacy interest/engagement. Data analysis will be conducted using Sawtooth's built-in analysis capabilities and R version 4.1.2.

For peer review only

ETHICS CONSIDERATION AND DISSEMINATION

Ethics consideration

This study was approved by the University of the Washington Human Research Ethics Committee, the Kenya Medical Research Institute (KEMRI), Nairobi County and the Scientific and Ethics Review Unit (SERU) in Kenya. Participation in the DCE is voluntary and subject to completion of an electronic informed consent. Respondents' personal information is kept secure and confidential. Participants are not required to use their real names, and we do not collect any identifiable information. Participants are told that they can withdraw from the survey at any time.

Dissemination

Findings will be shared with both local and international audiences at international conferences and peer-reviewed publications. We will disseminate results at engagement meetings with key informants including Kenya's NASCOP and other stakeholders. Preliminary results will be used to inform the design of the ePrEP Kenya Pilot study (NCT05377138) which will evaluate uptake and continuation rates for online PrEP delivery via MYDAWA in Nairobi,

For peer review only - http://bmjopen.bmj.com/site/about/guidelines.xhtml

Kenya. These results can also be utilized to design online PrEP delivery models to optimize PrEP uptake in Kenya and similar settings.

Acknowledgement

We would like to thank Peter Mogere from KEMRI and Daniel Were from Jhpiego for participating in stakeholder discussions. We thank Tony Wood, Lisbet Charana, and other MYDAWA staff for their support of this project and assistance with promoting the study on their website. We acknowledge Nicole Young and her colleagues at the Gates Foundation for their ongoing support and funding for this work [Grant #: INV-037646]. We are grateful to study staff and survey participants.

Data availability statement

Not applicable, as this is a protocol paper and there is no associated data.

Author contributions

MS, KO, and MLM conceived the study. YC, ES, MM, KN, NT, CK, FT-P, AS, MLM, KO, MS contributed to its design. YC, ES, MM, and MS wrote the first draft of the protocol and designed the DCE tasks. All authors contributed to the drafting and editing of the manuscript, and approved the final version.

Conflicts of Interest: The authors report no conflicts of interest.

Funding: This study was funded by the Bill and Melinda Gates Foundation [Grant #: INV-037646]. The funders had no role in study design, data collection, analysis, writing of the report, nor the decision to submit for publication.

For peer review only - http://bmjopen.bmj.com/site/about/guidelines.xhtml

References

1. Cherutich P, Kaiser R, Galbraith J, et al. Lack of knowledge of HIV status a major barrier to HIV prevention, care and treatment efforts in Kenya: results from a nationally representative study. *PLoS One* 2012;7(5):e36797. doi: 10.1371/journal.pone.0036797 [published Online First: 2012/05/11]
2. CDC. Kenya Country Profile: Centers for Disease Control and Prevention; 2022 [Available from: https://www.cdc.gov/globalhivtb/where-we-work/kenya/kenya.html accessed August 4 2022.
3. Ortblad KF, Baeten JM, Cherutich P, et al. The arc of HIV epidemics in sub-Saharan Africa: new challenges with concentrating epidemics in the era of 90-90-90. *Curr Opin HIV AIDS* 2019;14(5):354-65. doi: 10.1097/COH.0000000000000569 [published Online First: 2019/07/26]
4. The National AIDS Control Council 2022 [Available from: https://nacc.or.ke/ accessed July 17 2022.
5. Kenya Ministry of Health. Guidelines on the use of antiretroviral drugs for treating and preventing HIV infection in Kenya - 2018 edition. Nairobi: Kenya Ministry of Health, 2018.
6. Koss CA, Charlebois ED, Ayieko J, et al. Uptake, engagement, and adherence to pre-exposure prophylaxis offered after population HIV testing in rural Kenya and Uganda: 72-week interim analysis of observational data from the SEARCH study. *Lancet HIV* 2020;7(4):e249-e61. doi: 10.1016/S2352-3018(19)30433-3 [published Online First: 2020/02/23]
7. Were D, Musau A, Mutegi J, et al. Using a HIV prevention cascade for identifying missed opportunities in PrEP delivery in Kenya: results from a programmatic surveillance study. *J Int AIDS Soc* 2020;23 Suppl 3:e25537. doi: 10.1002/jia2.25537 [published Online First: 2020/07/01]
8. Mack N, Odhiambo J, Wong CM, et al. Barriers and facilitators to pre-exposure prophylaxis (PrEP) eligibility screening and ongoing HIV testing among target populations in Bondo and Rarieda, Kenya: results of a consultation with community stakeholders. *BMC Health Serv Res* 2014;14:231. doi: 10.1186/1472-6963-14-231 [published Online First: 2014/06/03]
9. Patel RC, Stanford-Moore G, Odoyo J, et al. "Since both of us are using antiretrovirals, we have been supportive to each other": facilitators and barriers of pre-exposure prophylaxis use in heterosexual HIV serodiscordant couples in Kisumu, Kenya. *J Int AIDS Soc* 2016;19(1):21134. doi: 10.7448/IAS.19.1.21134 [published Online First: 2016/12/15]
10. How long will they take it? Oral pre-exposure prophylaxis (PrEP) retention for female sex workers, men who have sex with men and young women in a demonstration project in Kenya. Journal of the International AIDS Society; 2018. JOHN WILEY & SONS LTD THE ATRIUM, SOUTHERN GATE, CHICHESTER PO19 8SQ, W ….
11. Factors influencing initiation, continuation & discontinuation of oral PrEP at selected facilities in South Africa. JOURNAL OF THE INTERNATIONAL AIDS SOCIETY; 2018. JOHN WILEY & SONS LTD THE ATRIUM, SOUTHERN GATE, CHICHESTER PO19 8SQ, W ….
12. Touger R, Wood BR. A Review of Telehealth Innovations for HIV Pre-Exposure Prophylaxis (PrEP). *Curr HIV/AIDS Rep* 2019;16(1):113-19. doi: 10.1007/s11904-019-00430-z [published Online First: 2019/02/01]
13. Kamulegeya LH, Bwanika JM, Musinguzi D, et al. Continuity of health service delivery during the COVID-19 pandemic: the role of digital health technologies in Uganda. *Pan Afr Med J* 2020;35(Suppl 2):43. doi: 10.11604/pamj.supp.2020.35.2.23115 [published Online First: 2021/02/25]
14. Dodoo JE, Al-Samarraie H, Alzahrani AI. Telemedicine use in Sub-Saharan Africa: Barriers and policy recommendations for Covid-19 and beyond. *Int J Med Inform* 2021;151:104467. doi: 10.1016/j.ijmedinf.2021.104467 [published Online First: 2021/04/30]
15. Chen Y, Ronen K, Matemo D, et al. An Interactive Text Messaging Intervention to Improve Adherence to Option B+ Prevention of Mother-to-Child HIV Transmission in Kenya: Cost Analysis. *JMIR Mhealth Uhealth* 2020;8(10):e18351. doi: 10.2196/18351 [published Online First: 2020/10/03]

For peer review only - http://bmjopen.bmj.com/site/about/guidelines.xhtml

16. ZALO MA. The regulation of online pharmacies dispensing prescription-only medicines in Kenya: a consumer protection perspective. 2020
17. Bridges JF. Stated preference methods in health care evaluation: an emerging methodological paradigm in health economics. *Appl Health Econ Health Policy* 2003;2(4):213-24. [published Online First: 2004/05/04]
18. Lancsar E, Louviere J. Conducting discrete choice experiments to inform healthcare decision making: a user's guide. *Pharmacoeconomics* 2008;26(8):661-77. doi: 10.2165/00019053-200826080-00004 [published Online First: 2008/07/16]
19. Eshun-Wilson I, Kim HY, Schwartz S, et al. Exploring Relative Preferences for HIV Service Features Using Discrete Choice Experiments: a Synthetic Review. *Curr HIV/AIDS Rep* 2020;17(5):467-77. doi: 10.1007/s11904-020-00520-3 [published Online First: 2020/08/30]
20. Quaife M, Eakle R, Cabrera Escobar MA, et al. Divergent Preferences for HIV Prevention: A Discrete Choice Experiment for Multipurpose HIV Prevention Products in South Africa. *Med Decis Making* 2018;38(1):120-33. doi: 10.1177/0272989X17729376 [published Online First: 2017/09/03]
21. Zanolini A, Sikombe K, Sikazwe I, et al. Understanding preferences for HIV care and treatment in Zambia: Evidence from a discrete choice experiment among patients who have been lost to follow-up. *PLoS Med* 2018;15(8):e1002636. doi: 10.1371/journal.pmed.1002636 [published Online First: 2018/08/14]
22. Ostermann J, Njau B, Brown DS, et al. Heterogeneous HIV testing preferences in an urban setting in Tanzania: results from a discrete choice experiment. *PLoS One* 2014;9(3):e92100. doi: 10.1371/journal.pone.0092100 [published Online First: 2014/03/20]
23. Wulandari LPL, He SY, Fairley CK, et al. Preferences for pre-exposure prophylaxis for HIV: A systematic review of discrete choice experiments. *EClinicalMedicine* 2022;51:101507. doi: 10.1016/j.eclinm.2022.101507 [published Online First: 2022/07/19]
24. Pereira CCA, Torres TS, Luz PM, et al. Preferences for pre-exposure prophylaxis (PrEP) among men who have sex with men and transgender women at risk of HIV infection: a multicentre protocol for a discrete choice experiment in Brazil. *BMJ Open* 2021;11(9):e049011. doi: 10.1136/bmjopen-2021-049011 [published Online First: 2021/09/29]
25. Quaife M, Eakle R, Cabrera M, et al. Preferences for ARV-based HIV prevention methods among men and women, adolescent girls and female sex workers in Gauteng Province, South Africa: a protocol for a discrete choice experiment. *BMJ Open* 2016;6(6):e010682. doi: 10.1136/bmjopen-2015-010682 [published Online First: 2016/06/30]
26. Ostermann J, Njau B, Hobbie A, et al. Using discrete choice experiments to design interventions for heterogeneous preferences: protocol for a pragmatic randomised controlled trial of a preference-informed, heterogeneity-focused, HIV testing offer for high-risk populations. *BMJ Open* 2020;10(11):e039313. doi: 10.1136/bmjopen-2020-039313 [published Online First: 2020/11/08]
27. Terris-Prestholt F, Mulatu A, Quaife M, et al. Using choice experiments to improve equity in access to socially marketed HIV prevention products. *Journal of choice modelling* 2021;41:100319.
28. Beckham SW, Crossnohere NL, Gross M, et al. Eliciting Preferences for HIV Prevention Technologies: A Systematic Review. *Patient* 2021;14(2):151-74. doi: 10.1007/s40271-020-00486-9 [published Online First: 2020/12/16]
29. MYDAWA. MYDAWA - Most Trusted Online Pharmacy in Kenya [Available from: https://mydawa.com/ accessed July 17 2022.
30. Jhpiego. Fifty Years of Transforming Global Health 2022 [Available from: https://www.jhpiego.org accessed August 4 2022.
31. Mugambi M. Evaluate and Optimize an Online Care Model for PrEP Delivery: Pilot Study (ePrEP Kenya) 2022 [Available from: https://clinicaltrials.gov/ct2/show/NCT05377138.
32. Bridges JF, Hauber AB, Marshall D, et al. Conjoint analysis applications in health--a checklist: a report of the ISPOR Good Research Practices for Conjoint Analysis Task Force. *Value Health* 2011;14(4):403-13. doi: 10.1016/j.jval.2010.11.013 [published Online First: 2011/06/15]

For peer review only - http://bmjopen.bmj.com/site/about/guidelines.xhtml

33. Harris PA, Taylor R, Thielke R, et al. Research electronic data capture (REDCap)--a metadata-driven methodology and workflow process for providing translational research informatics support. *J Biomed Inform* 2009;42(2):377-81. doi: 10.1016/j.jbi.2008.08.010 [published Online First: 2008/10/22]
34. Sawtooth Software. The CBC System for Choice-Based Conjoint Analysis, Version 9, 2017.
35. Ortblad KF, Mogere P, Roche S, et al. Design of a care pathway for pharmacy-based PrEP delivery in Kenya: results from a collaborative stakeholder consultation. *BMC Health Serv Res* 2020;20(1):1034. doi: 10.1186/s12913-020-05898-9 [published Online First: 2020/11/13]
36. Mugo PM, Micheni M, Shangala J, et al. Uptake and Acceptability of Oral HIV Self-Testing among Community Pharmacy Clients in Kenya: A Feasibility Study. *PLoS One* 2017;12(1):e0170868. doi: 10.1371/journal.pone.0170868 [published Online First: 2017/01/27]
37. Kenya Ministry of Health. Framework for the implementation of pre-exposure prophylaxis of HIV in Kenya. Kenya: Ministry of Health, 2017.
38. Forsythe S, Arthur G, Ngatia G, et al. Assessing the cost and willingness to pay for voluntary HIV counselling and testing in Kenya. *Health Policy Plan* 2002;17(2):187-95. doi: 10.1093/heapol/17.2.187 [published Online First: 2002/05/10]
39. Bobinac A, Van Exel NJ, Rutten FF, et al. Willingness to pay for a quality-adjusted life-year: the individual perspective. *Value Health* 2010;13(8):1046-55. doi: 10.1111/j.1524-4733.2010.00781.x [published Online First: 2010/09/10]
40. Ashburn K, Antelman G, N'Goran MK, et al. Willingness to use HIV self-test kits and willingness to pay among urban antenatal clients in Cote d'Ivoire and Tanzania: a cross-sectional study. *Trop Med Int Health* 2020;25(9):1155-65. doi: 10.1111/tmi.13456 [published Online First: 2020/07/02]
41. Noun Project. Icons and Photos For Everything 2022 [Available from: https://thenounproject.com/.
42. Reed Johnson F, Lancsar E, Marshall D, et al. Constructing experimental designs for discrete-choice experiments: report of the ISPOR Conjoint Analysis Experimental Design Good Research Practices Task Force. *Value Health* 2013;16(1):3-13. doi: 10.1016/j.jval.2012.08.2223 [published Online First: 2013/01/23]
43. Kuhfeld WF, Tobias RD, Garratt M. Efficient Experimental Design with Marketing Research Applications. *Journal of Marketing Research* 1994;31(4):545-57. doi: 10.2307/3151882
44. Valuing environmental amenities using stated choice studies : a common sense approach to theory and practice; 2010.
45. Brazell JD, Diener CG, Karniouchina E, et al. The no-choice option and dual response choice designs. *Marketing Letters* 2006;17(4):255-68. doi: 10.1007/s11002-006-7943-8
46. Determann D, Gyrd-Hansen D, de Wit GA, et al. Designing Unforced Choice Experiments to Inform Health Care Decision Making: Implications of Using Opt-Out, Neither, or Status Quo Alternatives in Discrete Choice Experiments. *Med Decis Making* 2019;39(6):681-92. doi: 10.1177/0272989X19862275 [published Online First: 2019/07/30]
47. Wlömert N, Eggers F. Predicting new service adoption with conjoint analysis: external validity of BDM-based incentive-aligned and dual-response choice designs. *Marketing Letters* 2016;27(1):195-210. doi: 10.1007/s11002-014-9326-x
48. Bahamonde-Birke FJ, Navarro I, Ortúzar JdD. If you choose not to decide, you still have made a choice. *Journal of Choice Modelling* 2017;22:13-23. doi: https://doi.org/10.1016/j.jocm.2016.11.002
49. Orme B. Getting started with conjoint analysis: strategies for product design and pricing research: Research Publishers 2006.
50. Orme B. Sample size issues for conjoint analysis studies: Sawtooth Software 2019.
51. Johnson RO, B. Getting the Most from CBC, 2003.
52. de Bekker-Grob EW, Donkers B, Jonker MF, et al. Sample Size Requirements for Discrete-Choice Experiments in Healthcare: a Practical Guide. *Patient* 2015;8(5):373-84. doi: 10.1007/s40271-015-0118-z [published Online First: 2015/03/03]

For peer review only - http://bmjopen.bmj.com/site/about/guidelines.xhtml

53. Louviere JJ, Hensher DA, Swait JD. Stated Choice Methods: Analysis and Applications. Cambridge: Cambridge University Press 2000.
54. McFadden D. Conditional logit analysis of qualitative choice behavior. 1973
55. Hauber AB, Gonzalez JM, Groothuis-Oudshoorn CG, et al. Statistical Methods for the Analysis of Discrete Choice Experiments: A Report of the ISPOR Conjoint Analysis Good Research Practices Task Force. *Value Health* 2016;19(4):300-15. doi: 10.1016/j.jval.2016.04.004 [published Online First: 2016/06/22]
56. Collins LM, Lanza ST. Latent class and latent transition analysis: With applications in the social, behavioral, and health sciences: John Wiley & Sons 2009.

For peer review only

Figure legends

Figure 1. Study design flow chart
Figure 2. Example of DCE choice task

For peer review only - http://bmjopen.bmj.com/site/about/guidelines.xhtml

Figure 1. Study design flow chart

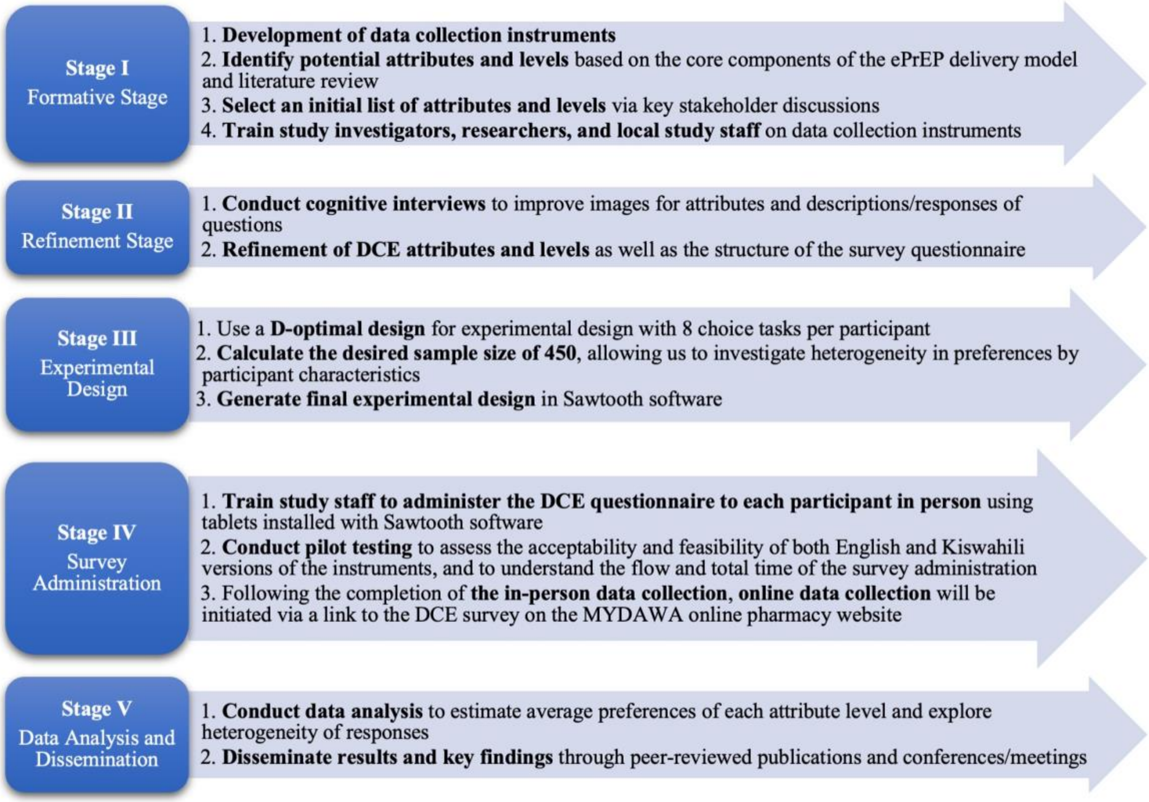

For peer review only - http://bmjopen.bmj.com/site/about/guidelines.xhtml

Figure 2. Example of DCE choice task

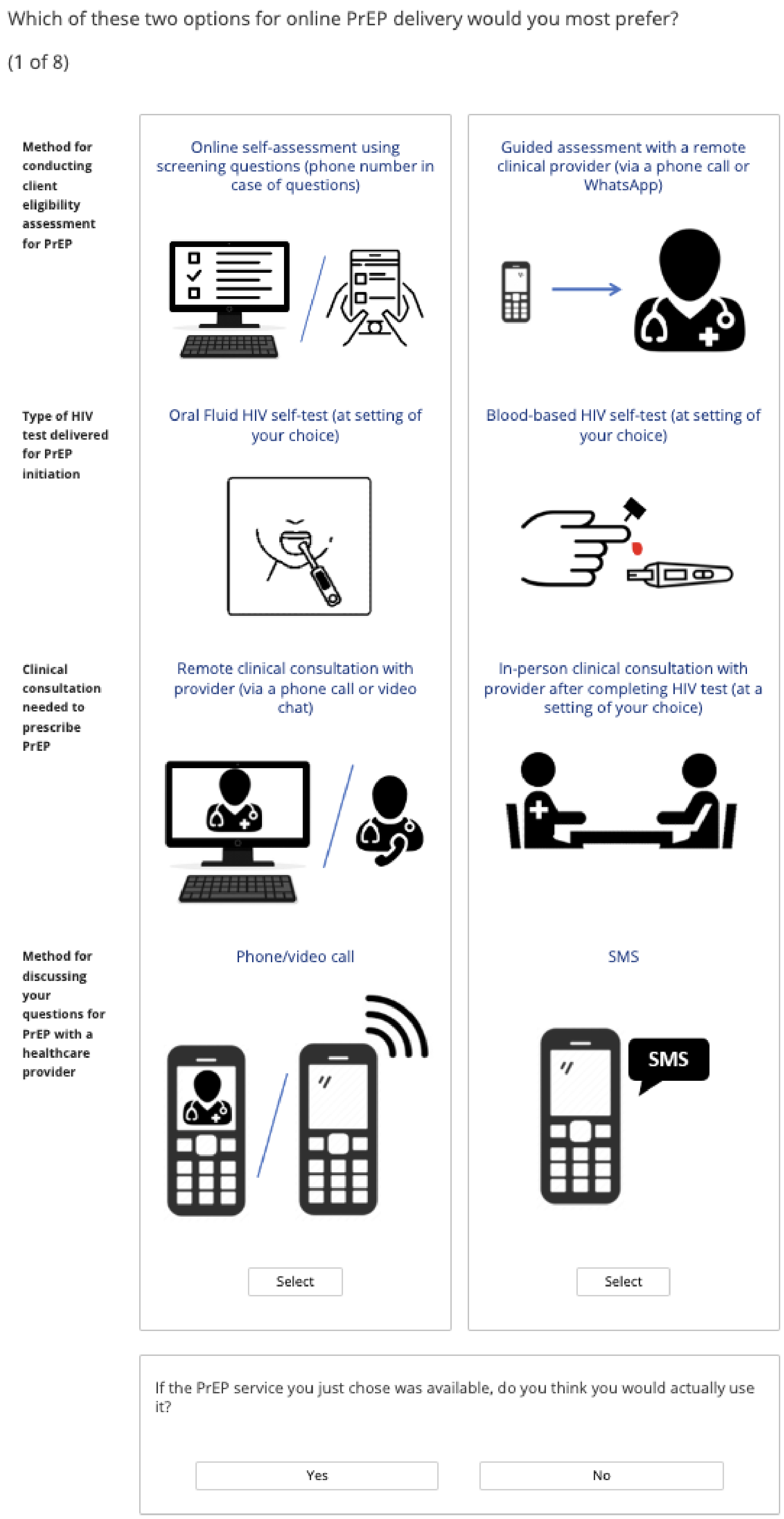

Supplement 1: Cognitive Interview Guide

Online-pharmacy for the delivery of pre-exposure prophylaxis (PrEP) medicines

Discrete Choice Experiment

Cognitive Interview

Introduction

Thank you for taking the time to speak with me. My name is ______. We would like to speak to you about pre-exposure prophylaxis or PrEP. PrEP is a medicine you can take to reduce your risk of getting HIV. We have asked you to participate in this exercise because we are interested in learning how best to delivery PrEP using an online pharmacy. This means a pharmacy will deliver PrEP medication to clients using a courier, so clients do not need to travel to a pharmacy. The aim of this session is to ask you about pictures that are related to PrEP delivery and how they can be improved for our survey instrument. We will also ask you for feedback about other section of our survey to make sure they are clear. This interview should take about 60 minutes. If it's OK with you, I would like to record the interview, so I don't miss any important details in your answers. Feel free to let me know if you need a break at any time. You can also stop the interview if you do not want to continue. Before we begin do you have any questions? Can I start recording?

[Prompt: Start recording]

For peer review only

Part 1. Presentation of Attributes and Levels

Online PrEP delivery includes different services, including an assessment to determine if you are at HIV risk, HIV testing to confirm you are HIV-negative, and talking to a medical provider to make sure PrEP is safe for you.

We will describe the characteristics of obtaining PrEP through online pharmacy in the order you would experience them.

For peer review only

Part 1, a.

PrEP Eligibility Assessment [No need to read the header out loud]

Please tell me how you would interpret these images. [Show the images. Alternative text: What do these images mean to you?]

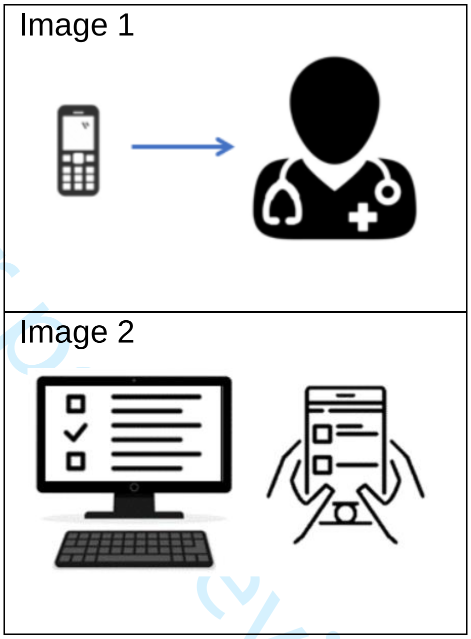

For peer review only

Now I'm going to read you the description and possible ways to have a PrEP Eligibility assessment

PrEP Eligibility Assessment

First, you would complete an **eligibility assessment** to see if PrEP is a good fit for you. This is done by answering questions about your sexual behavior and partners. You can do this in **two ways**: One way is with a provider asking you these questions over the phone or WhatsApp.

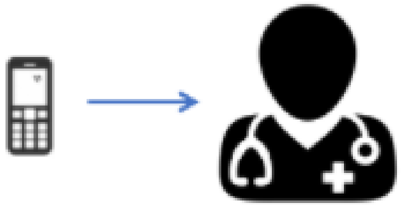

The second way is by answering these questions by yourself on your computer or phone. You will have a phone number to call in case of questions.

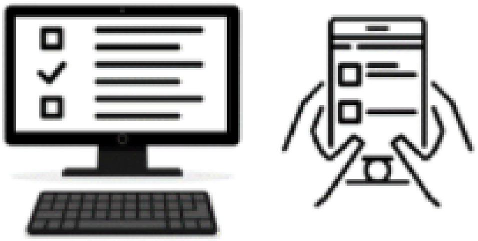

Questions:

- Is the description of "PrEP eligibility Assessment" clear to you? [If necessary, read the description again]
    - Is there any other information we should include to make the description clearer?
- Do the images represent the descriptions well? How could we modify the image to make it clearer?
    - Do you think another person would understand the meaning of this image?
- Are there other ways to assess PrEP eligibility that you think we should include?

Part 1, b.

HIV Test Type [No need to read the header out loud]

Please tell me what you think these images mean. [Show the images]

Image 1

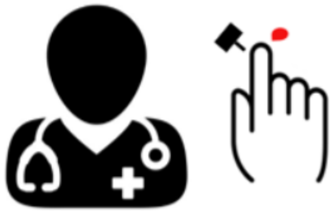

Image 2

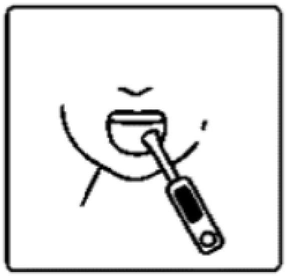

Image 3

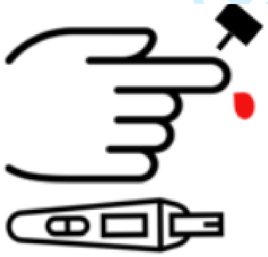

For peer review only

Now I'm going to read you the description and possible ways to have an HIV Test

HIV Test Type

Next you would take an HIV test to make sure you are HIV-negative before starting PrEP. You can test for HIV in one of 3 ways.

One way is to have a healthcare provider meet you at a setting of your choice to conduct HIV test by taking blood from your finger.

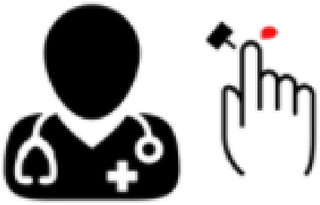

The second way is testing yourself using an HIV self-test kit that uses oral fluid that you collect by swabbing your cheek and gums.

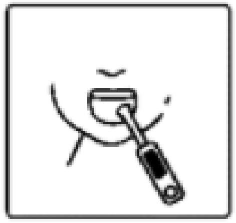

And the third way is testing yourself using a HIV self-test kit that uses blood that you collect from your finger (through a finger prick). Both self-testing kits would be delivered to a setting of your choice and would show a test result after about 20 minutes.

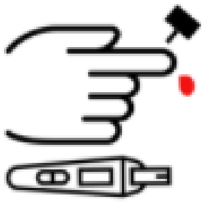

Questions:

- Is the description of "HIV Test Type" clear to you?
    - Is there any other information we should include to make the description clearer?
- Do the images represent the descriptions well? How could we modify the image to make it clearer?
    - Do you think another person would understand the meaning of this image?
- Are there other HIV Test Types that you think we should include?

Is any of the following images a better match for the descriptions I showed you before?

[Repeat the description of the ways to obtain HIV Test if necessary]

New	Original
Image 1 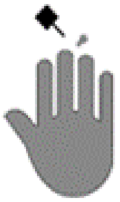 [Self-testing]	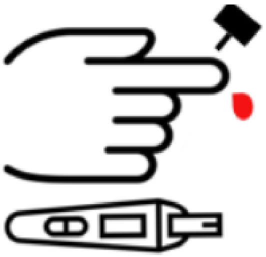
Image 2 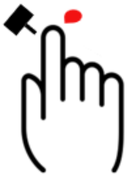 [Self-testing]	
Image 3 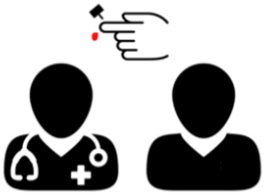 [Blood -based testing]	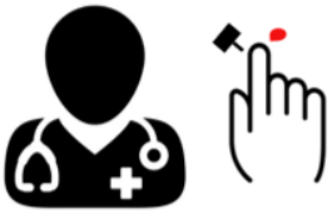
Image 3 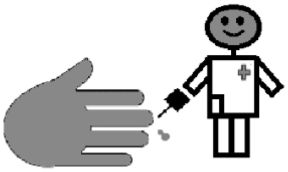 [Blood -based testing]	

Part 1, c.

Clinical Consultation [No need to read the header out loud]

Please tell me how you would interpret these images. [Show the images]

Image 1

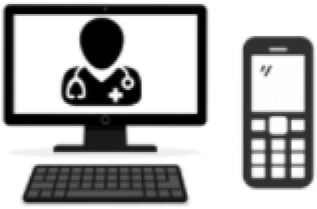

Image 2

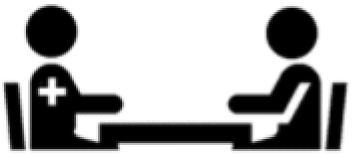

For peer review only

Now I'm going to read you the description and different possible ways to have a Clinical Consultation

Clinical Consultation

You would then have a clinical consultation with a medical provider to make sure PrEP is safe for you to use. This can be done over the phone or a video chat.

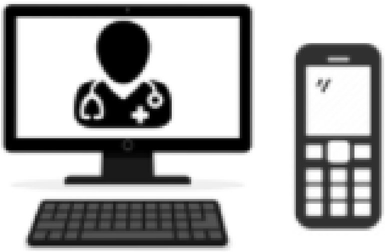

Another option is to see a provider in person at a setting of your choice.

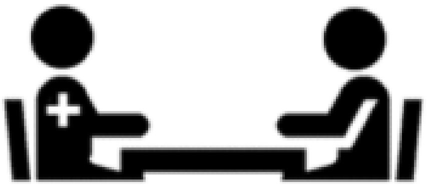

Questions:

- Is the description of "Clinical Consultation" clear to you?
    - Is there any other information we should include to make the description clearer?
- Do the images represent the descriptions well? How could we modify the image to make it clearer?
    - Do you think another person would understand the meaning of this image?
- Are there other ways to provide Clinical consultation that you think we should include?

Is any of the following images a better match for the descriptions I showed you before? [Repeat the description of the ways to provide Clinical Consultation if necessary]

New	Original
Image 1<br>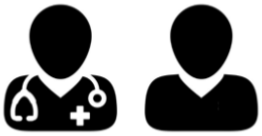<br>[In-person consultation]	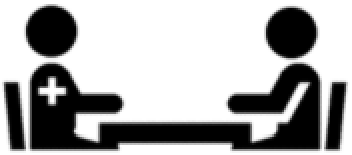
Image 2<br>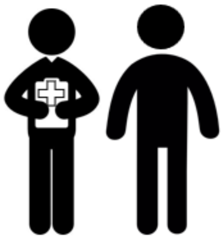<br>[In-person consultation]	

For peer review only

Part 1, d.

User Support Options [No need to read the header out loud]

Please tell me how you would interpret these images. [Show the images]

Image 1

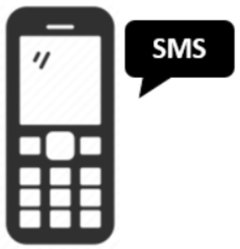

Image 2

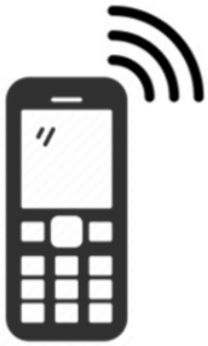

Image 3

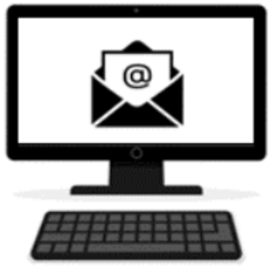

For peer review only

Now I’m going to read you the description and different ways to get User Support

User Support Options

In case you have any questions about PrEP, there are **three different options** for you to discuss your questions with a healthcare provider. The first is using SMS to talk to a provider.

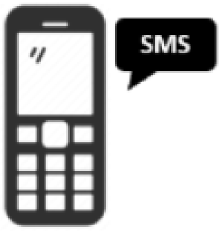

The second option is talking to a provider over the phone, using either voice or video call.

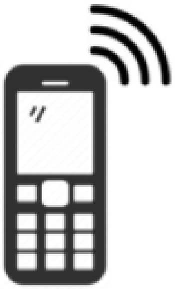

The third is using email to talk to a provider.

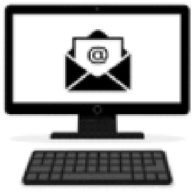

Questions:

- Is the description of “User Support Options” clear to you?
    - Is there any other information we should include to make the description clearer?
- Do the images represent the descriptions well? How could we modify the image to make it clearer?
    - Do you think another person would understand the meaning of this image?
- Are there other ways to provide user support that you think we should include?

For peer review only - http://bmjopen.bmj.com/site/about/guidelines.xhtml

Is any of the following images a better match for the descriptions I showed you before? [Repeat the description of the ways to provide User Support if necessary]

New	Original
Image 1 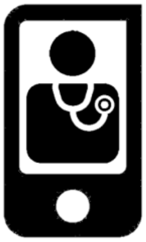 [Video call]	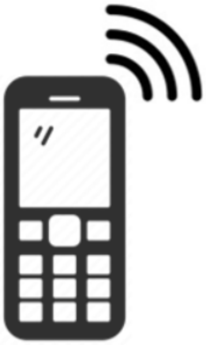
Image 2 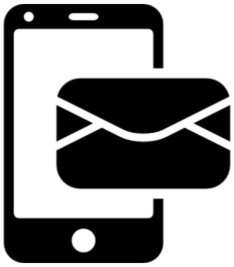 [Email]	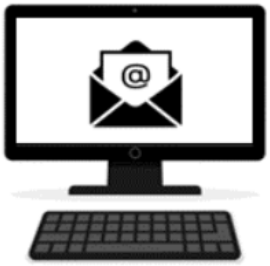

For peer review only - http://bmjopen.bmj.com/site/about/guidelines.xhtml

Part 1, e. Summary Table

**Characteristics**<br>*Description*	**Option 1**	**Option 2**	**Option 3**
**PrEP eligibility assessment**<br>*Method for conducting client eligibility assessment for PrEP*	*Online self-assessment* ***on your computer or phone*** *using screening questions (phone number in case of questions)*<br>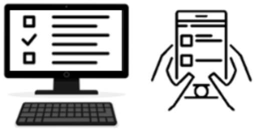	*Guided assessment with a remote clinical provider (via a phone call or WhatsApp)*<br>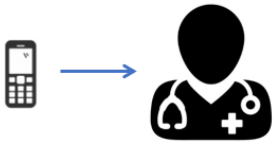	
**HIV test type**<br>*Type of HIV test delivered for PrEP initiation*	*Oral-fluid HIV self-test (at setting of your choice)*<br>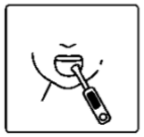	*Blood-based HIV self-test (at setting of your choice)*<br>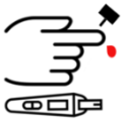	*Provider-administered blood-based rapid diagnostic test (at setting of your choice)*<br>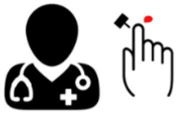
**Clinical consultation for prescribing PrEP**<br>*Clinical consultation needed to prescribe PrEP*	*Remote clinical consultation with provider (via a phone call or video chat)*<br>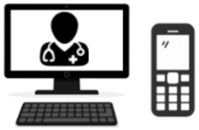	*In-person clinical consultation with provider after completing HIV testing (at a setting of your choice)*<br>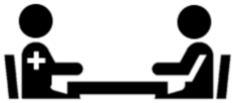	
**User support options for PrEP**<br>*Method for discussing your questions for PrEP with a healthcare provider*	*SMS*<br>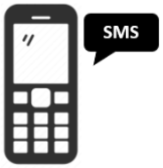	*Phone/video call*<br>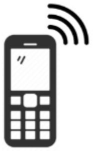	*Email*<br>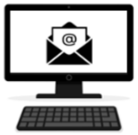

Questions:

We want to make sure we have included all the characteristics of online PrEP delivery that would be important to you

- Are any of the characteristics of PrEP delivery that we showed you today not important or don't add relevant information?
- Is there anything else about an online PrEP delivery service that would be important to you that we did not ask you about?

For peer review only - http://bmjopen.bmj.com/site/about/guidelines.xhtml

Part 2. DCE Choice Task Example

First Take:

I will now ask you about how you would like to get PrEP from an online pharmacy. Please remember there are no wrong answers, we want to know your preferences. When answering each scenario, imagine that you are planning to get PrEP using an online pharmacy.

When making your choice, please consider ONLY the characteristics shown.

For peer review only

Which of these two options for online PrEP delivery would you most prefer?

	A	B
**Method for conducting client eligibility assessment for PrEP**	Guided assessment with a remote clinical provider (via a phone call or WhatsApp)<br>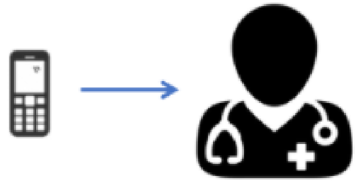	Online self-assessment using screening questions (phone number in case of questions)<br>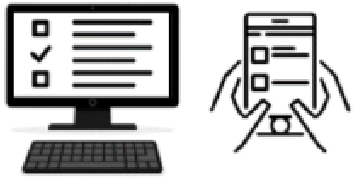
**Type of HIV test delivered for PrEP initiation**	Healthcare provider administers HIV Test at setting of your choice (blood-based)<br>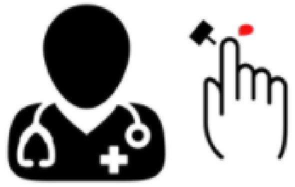	Oral Fluid HIV self-test (at setting of your choice)<br>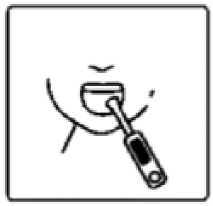
**Clinical consultation needed to prescribe PrEP**	In-person clinical consultation with provider after completing HIV test (at a setting of your choice)<br>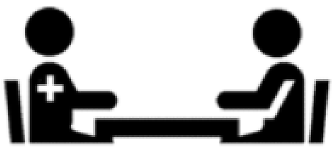	Remote clinical consultation with provider (via a phone call or video chat)<br>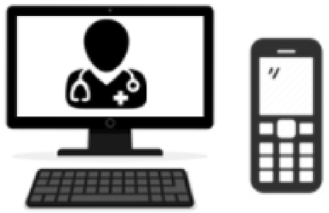
**Method for discussing your questions for PrEP with a healthcare provider**	Phone/video call<br>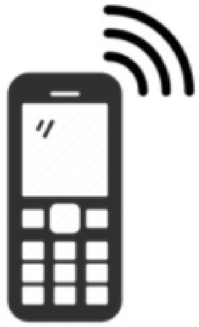	Email<br>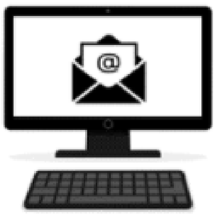

Questions:

- How hard did you find this exercise?
- How could we modify the exercise to make it easier?
- Do you think another person would understand this exercise?
- Is there anything in the question for the exercise that we should modify to make it clearer? [If necessary, read the question again "Which of these two options for online PrEP delivery would you most prefer?"]
- When I said, "When making your choice, please consider ONLY the characteristics I presented before.", what does that mean to you? [The objective of that sentence is to ignore any other characteristics of online pharmacy (e.g., the brand of the drug or the company providing the service) that are not included in the table. Check if the participant understood that.]
- Can you tell me how you made your decision? [Check if the participant is paying attention to all characteristics, if he doesn't mention one, ask about it. For example: I noticed you didn't mention "Clinical consultation", did you consider the option for that characteristic?"]

For peer review only

Second take:

I will now ask you about how you would like to get PrEP from an online pharmacy. Please remember there are no wrong answers, we want to know your preferences. When answering each scenario, imagine that you are planning to get PrEP using an online pharmacy. I will also ask you if you would choose to get PrEP using the option you selected. If you would not choose to get PrEP if only these two options were available, you can answer no to this question.

When making your choice, please consider ONLY the characteristics shown

For peer review only

Which of these two options for online PrEP delivery would you most prefer?

	A	B
**Method for conducting client eligibility assessment for PrEP**	Guided assessment with a remote clinical provider (via a phone call or WhatsApp) 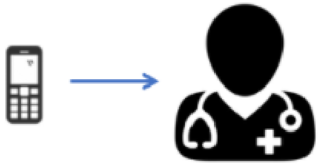	Online self-assessment using screening questions (phone number in case of questions) 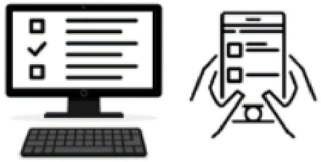
**Type of HIV test delivered for PrEP initiation**	Healthcare provider administers HIV Test at setting of your choice (blood-based) 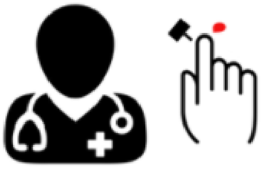	Oral Fluid HIV self-test (at setting of your choice) 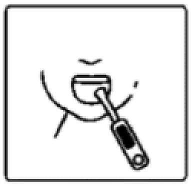
**Clinical consultation needed to prescribe PrEP**	In-person clinical consultation with provider after completing HIV test (at a setting of your choice) 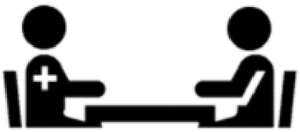	Remote clinical consultation with provider (via a phone call or video chat) 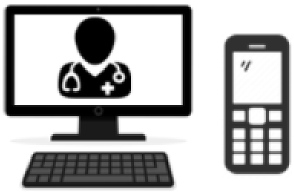
**Method for discussing your questions for PrEP with a healthcare provider**	Phone/video call 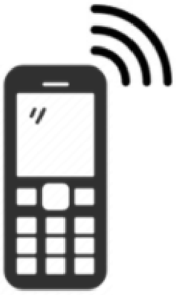	Email 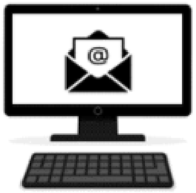

If the PrEP service you just chose was available, do you think you would actually use it?

Yes

No

Questions:

- How different was this time choosing an alternative compared to the first time? [Now the participant has the option to say if they would choose to get PrEP based on their previous selection or not]
- Was does the question “Would you choose to get PrEP using this service if it were available?” mean in your own words? What do you think is asking from you?
  - Our goal in asking this question is to understand if you would really choose to get this service if it were available to you. Do you think the question is clearly asking that?
  - If not, how can we make this question clearer? [If participant said they don’t know, that’s fine, move to the next section]
- How many of these tasks do you think you could answer before getting tired and/or bored?

For peer review only

Part c. Willingness to Pay Questions

The next set of questions will ask about how much you would like to pay for different parts of online PrEP delivery. For each part, please provide the lowest price, the maximum price and the ideal price you are willing to pay. For the lowest price, please tell us the lowest amount you think the service should cost because otherwise you would be concerned about its quality, and you would not purchase the service. In answering these questions, please consider your usual expenses. Remember that there are no right or wrong answers. We're interested in your preference. You can write zero if you feel that most accurately reflects your preference.

For a blood-based HIV self-test, delivered via a courier to a location of your choice (one-time cost):

What is the lowest price you are willing to pay? ______ KSH

What is the highest price you are willing to pay? ______ KSH

What price do you consider ideal? ______ KSH

For an oral HIV self-test, delivered via a courier to a location of your choice (one-time cost):

What is the lowest price you are willing to pay? ______ KSH

What is the highest price you are willing to pay? ______ KSH

What price do you consider ideal? ______ KSH

For remote/online clinical consultation to obtain a prescription for PrEP based on your HIV self-test results (one-time cost):

What is the lowest price you are willing to pay? ______ KSH

What is the highest price you are willing to pay? ______ KSH

What price do you consider ideal? ______ KSH

For delivery of PrEP medicines (delivery fee plus 1 month supply of medicines):

What is the lowest price you are willing to pay? ______ KSH

What is the highest price you are willing to pay? ______ KSH

What price do you consider ideal? ______ KSH

Questions:

- Overall, is it clear what the objective of the questions is? Would you mind explaining it to me in your own words?
- Please select one of the questions and give your answers. Would you explain me how did you select the amounts?
  [Pay attention to the order in which the participant finds the amounts. Which one comes first, the ideal, the lowest, or the highest?]
  - In this process, did you consider your usual expenses on medicines or healthcare?
  - What other expenses did you consider, if any?
  - Did you consider the cost options presented in the previous section?
- Is the instruction "For the lowest price, please tell us the lowest amount you think the service should cost because otherwise you would be concerned about its quality, and you would not purchase the service." clear enough? Would you tell me in your own words what does it mean?
  - Would you be concerned about the quality of a product that is free? Why or why not?
- Do you think that all questions are unique or some of them ask for the same information as others?

End of the interview

Do you have any questions or final thoughts you would like to share?

Thank you very much for your time and dedication in giving me your answers, I'm sure your information will help us create a better survey.

[Stop the recording]

For peer review only

Supplement 2: Training Materials

EPharmacy PrEP: Pilot Study Discrete Choice Experiment Training

24th-25th January 2022

Introductions

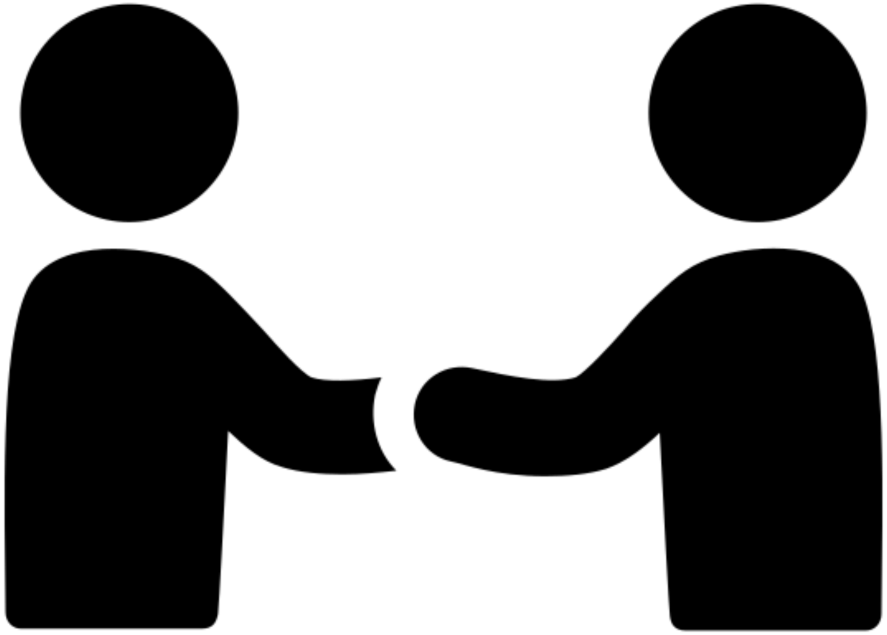

Overview of this session

- HIV in Kenya
- PrEP
- PrEP delivery strategies
- ePharmacy PrEP delivery
- Discrete Choice Experiments

Kenya has made significant progress in its fight against HIV.

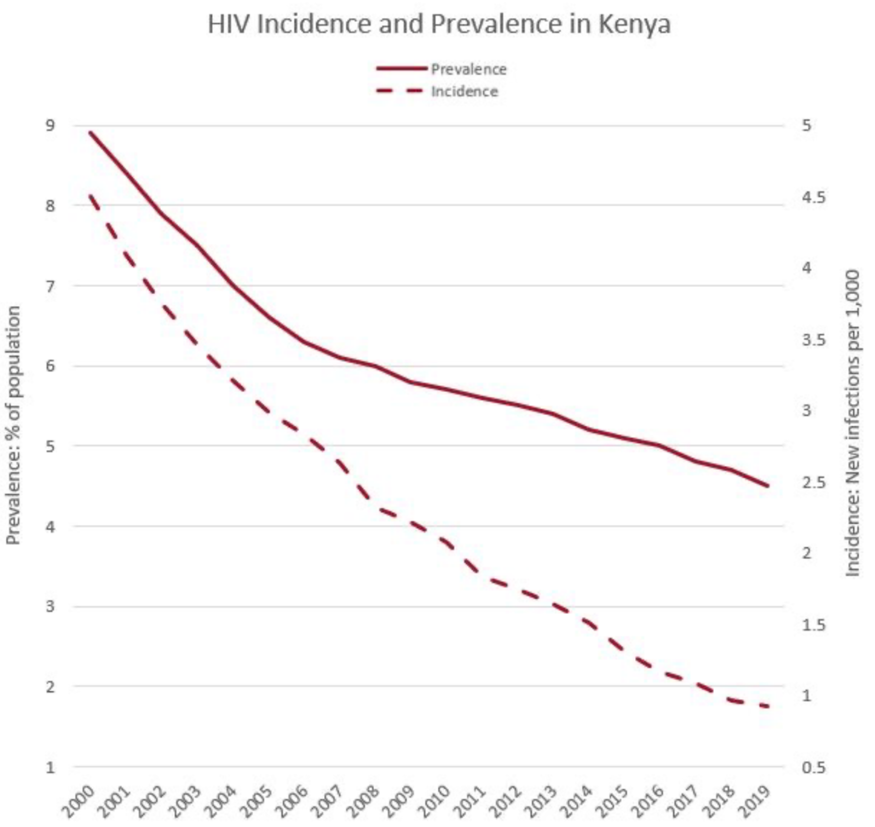

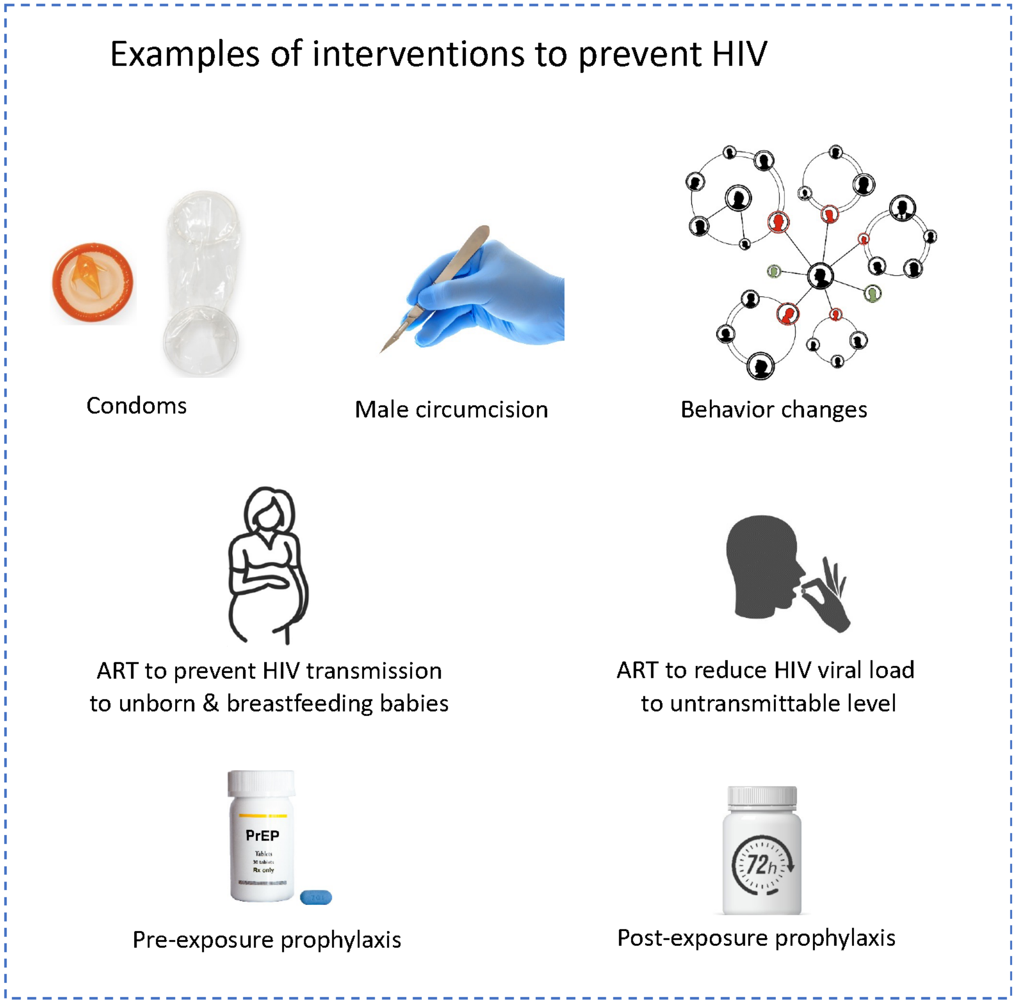

Source: World Development Indicators

But progress has been uneven across populations and counties.

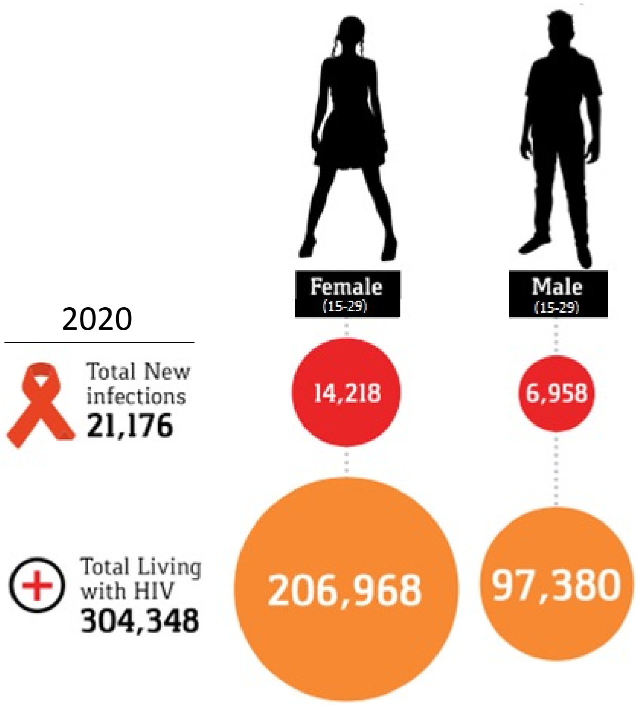

New HIV infections, 2020

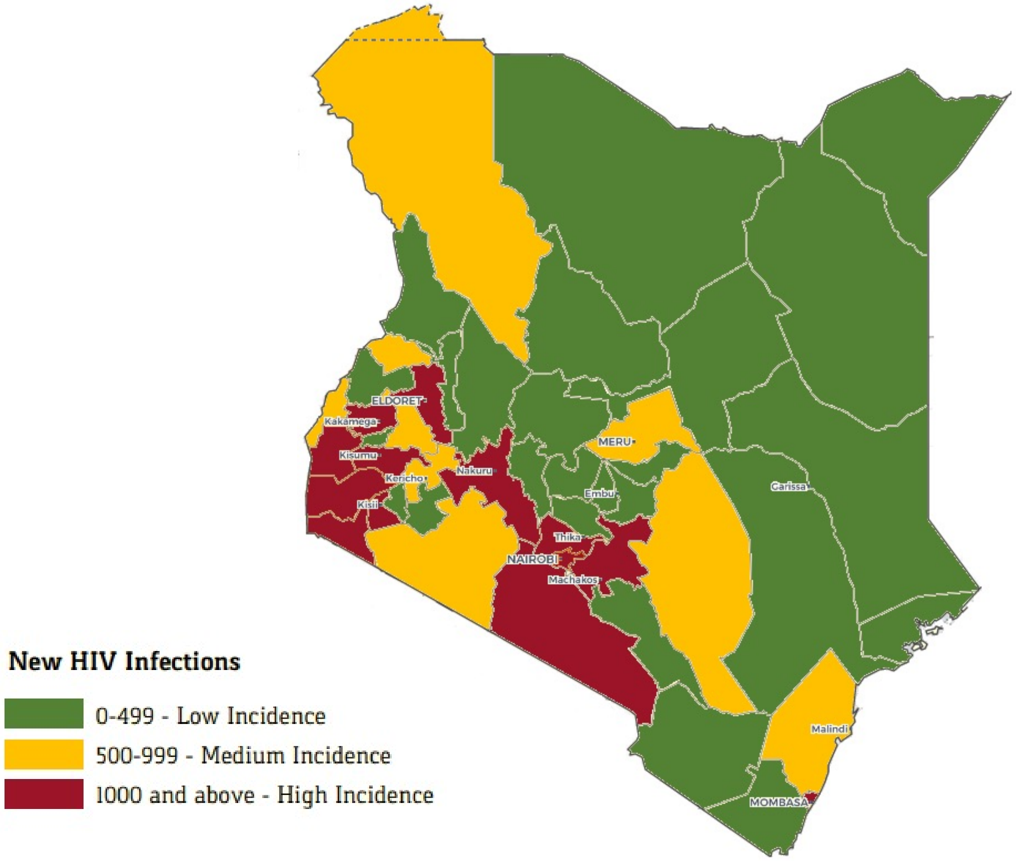

Source: KASF II 2021

PrEP is a core component of Kenya’s HIV prevention program

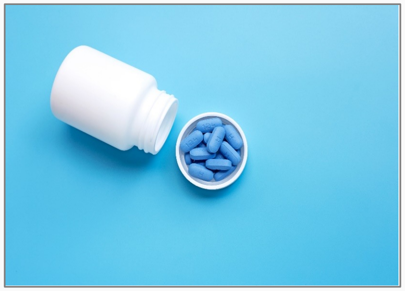

- Reduces risk of HIV from sex by at least 90% when taken daily.

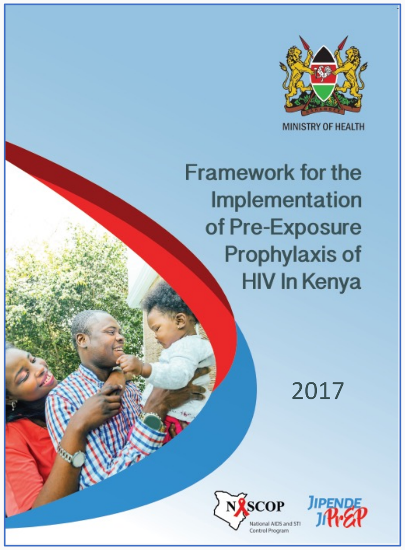

- Expanding PrEP to make it available nationwide
- 130,000 Kenyans have taken PrEP at least once
    - 4th most PrEP users in the world!

What is PrEP?

HIV pre-exposure prophylaxis (PrEP)

•It is a **HIV prevention** method in which a **HIV negative** person at **ongoing high risk** of HIV infection takes daily oral antiretroviral drug to prevent HIV infection

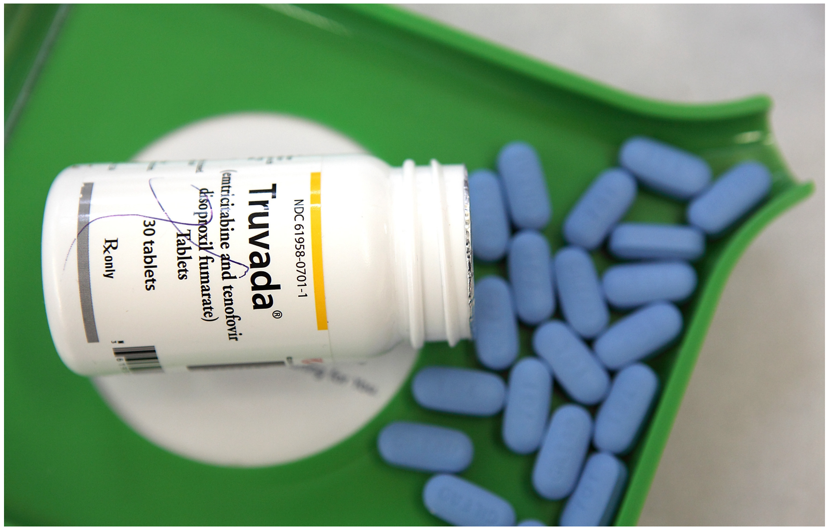

- PrEP is safe in pregnancy and for breastfeeding mothers
- It takes approximately **SEVEN** days after starting treatment for PrEP to be optimally effective.
- With poor adherence effectiveness becomes lower

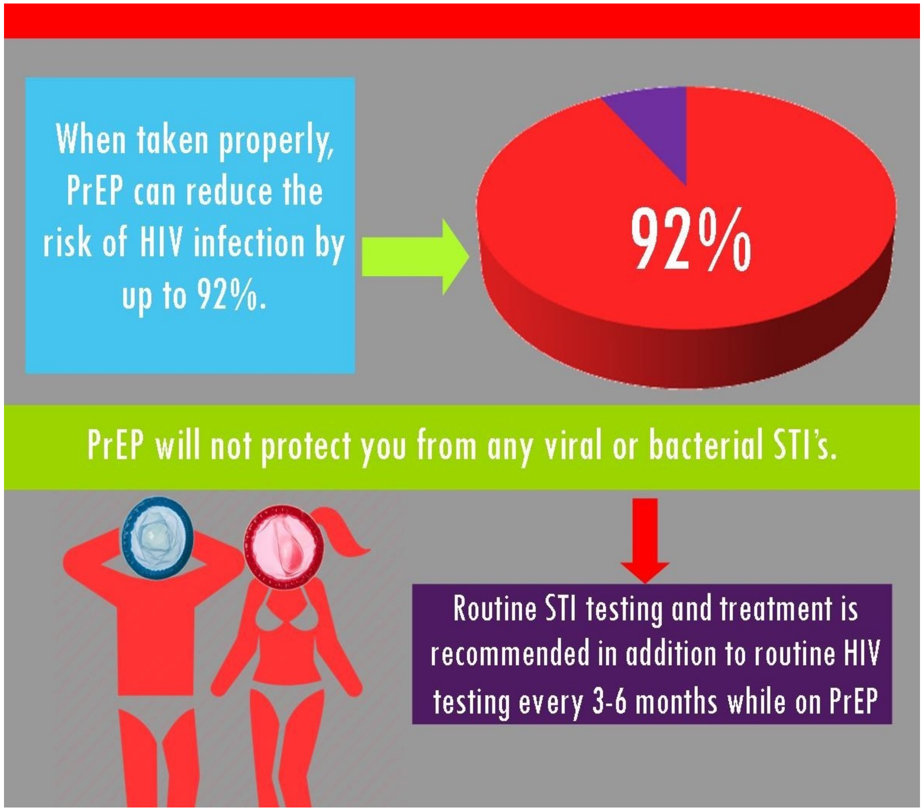

How is PrEP taken?

PrEP

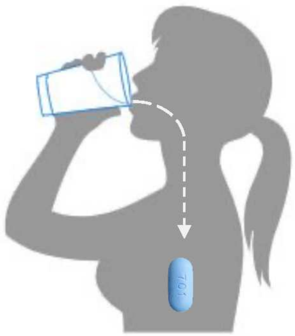

1 pill per day
No pre-specified duration of use

Recommended medication for PrEP use

PREFERRED	ALTERNATIVE
Tenofovir (TDF) + Emtricitabine (FTC) 300mg 200mg	Tenofovir (TDF) 300 mg
	Tenofovir (TDF) + Lamivudine (3TC) 300mg 300mg

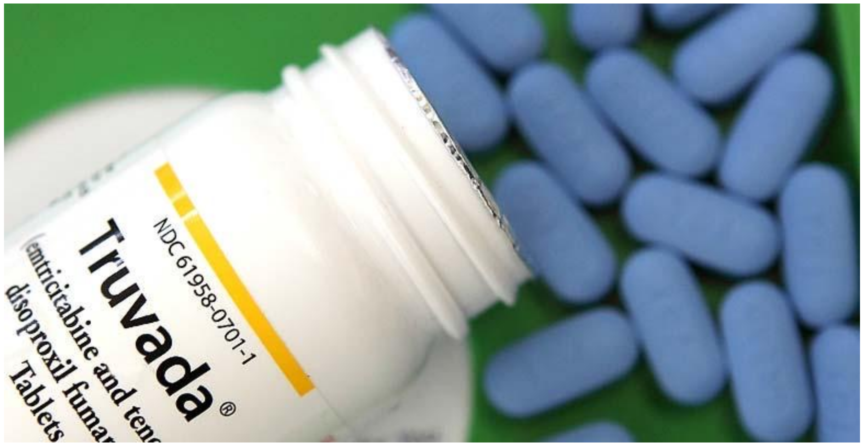

Does PrEP have side effects?

PrEP side effects

No major safety issues have been observed with use of PrEP.

About 1 in 10 people who use PrEP experience **minor**, **temporary** side effects, such as:

- Headache
- Weight loss
- Nausea
- Vomiting
- Abdominal discomfort

If side effects occur, they typically **start in the first few days or weeks** of PrEP use and **last a few days**—almost always less than 1 month.

References: AVAC (Nov. 2020); Tetteh et al. (2017)

What PrEP does and doesn’t prevent

PrEP reduces risk of getting HIV by at least 90%

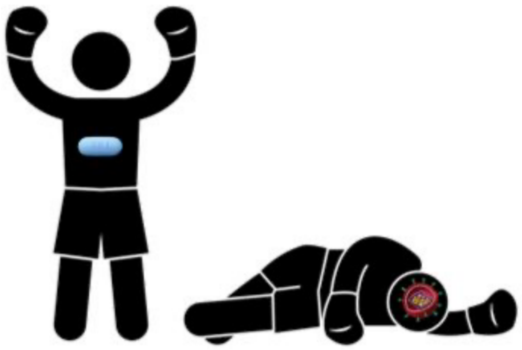

PrEP does **not** prevent sexually transmitted infections (STIs) or pregnancy.

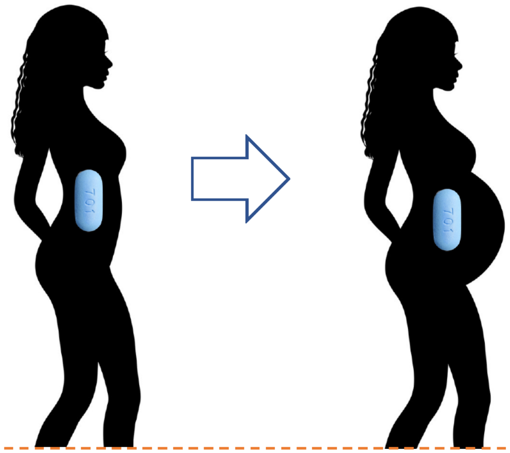

Prospective PrEP users should be advised that if they wish to protect themselves against STIs and/or pregnancy, they should **use PrEP in combination with other methods**, like condoms and contraception.

Aside from protecting against HIV, does PrEP have any other benefits?

Other PrEP benefits

Additional benefits of PrEP:

- Decreased anxiety or fear of contracting HIV
- Increased sense of empowerment and control over HIV
- Increased sense of pleasure and intimacy during sex
- Increased communication and trust with sex partners

Benefits specific to serodiscordant couples:

- Increased ability to cope with and accept serodiscordance
  (e.g., sense of solidarity taking daily medication)
- Ability to safely conceive a child

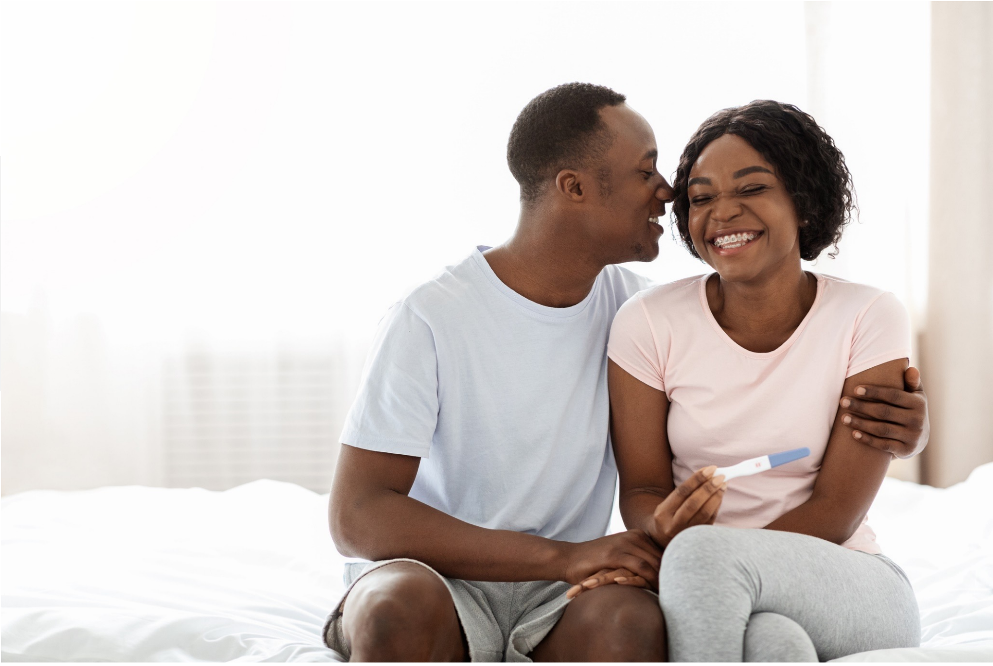

Sources: Gilmore et al. 2014; Ware et al. 2012; Ware et al. 2014

PrEP eligibility criteria

Per Kenya national guidelines

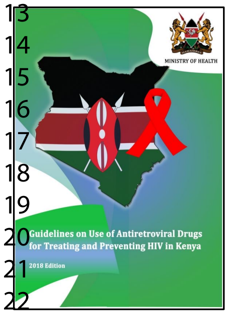

- Age 16+
- Confirmed HIV-negative
- Substantial, ongoing risk of HIV infection
- No signs of acute HIV infection in past month
- No contraindications to PrEP medications
- Willing to use PrEP as prescribed

*Specifically, HIV vaccine or HIV prevention studies

Currently, PrEP is primarily delivered in public HIV clinics (CCCs).

But this venue is not suitable for everyone.

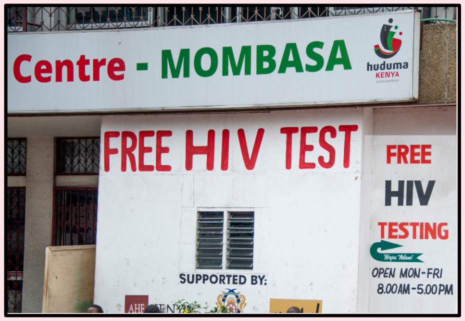

HIV stigma

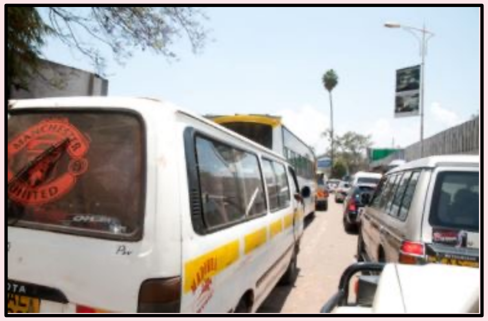

Travel distance, time & cost

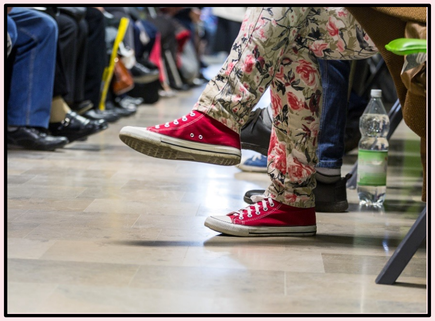

Long wait times

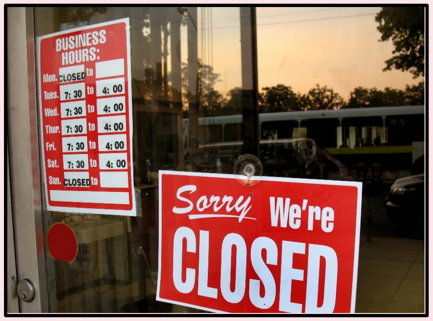

Limited opening hours

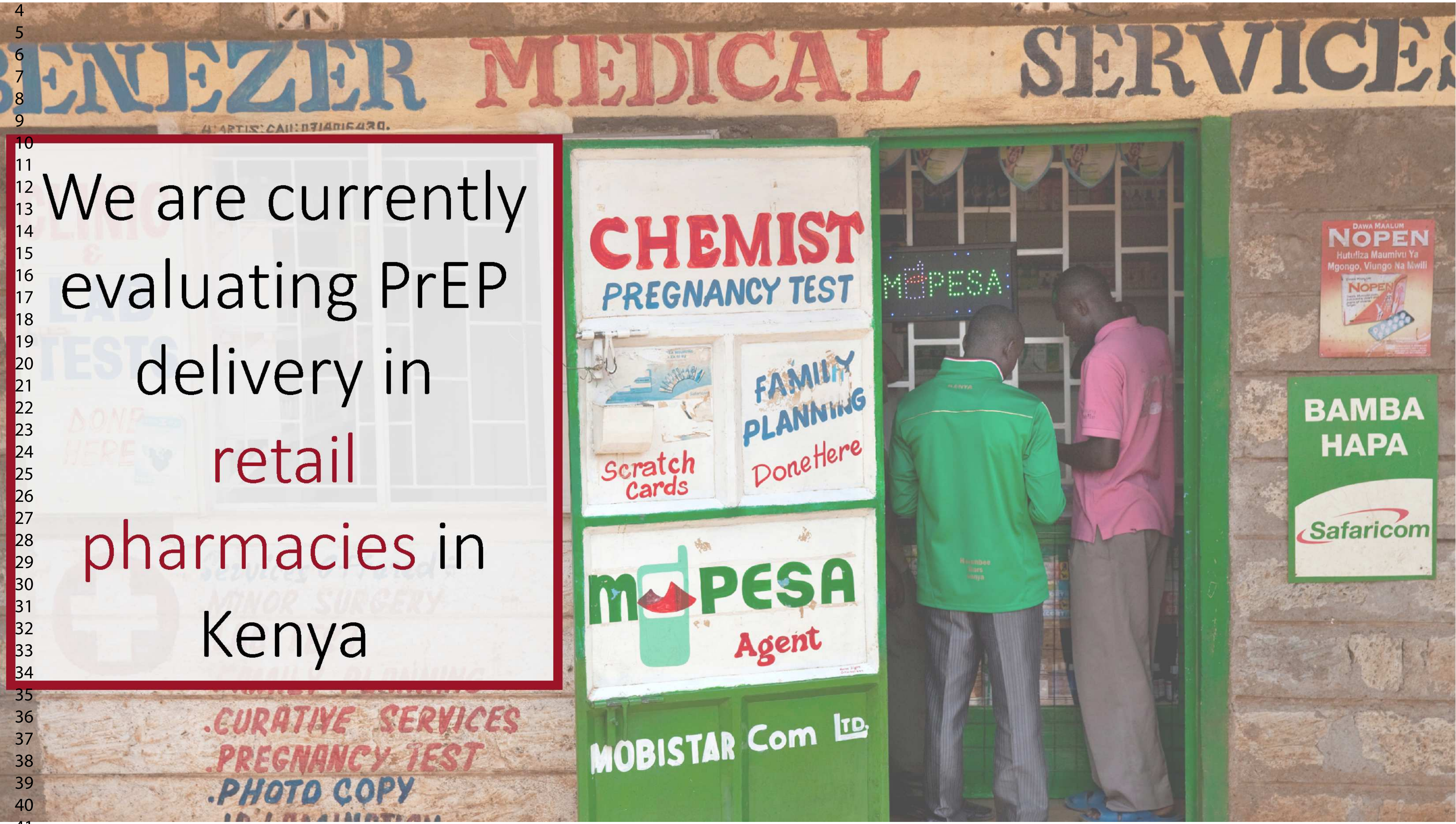
We are currently evaluating PrEP delivery in retail pharmacies in Kenya
MEDICAL SERVICES
CHEMIST
PREGNANCY TEST
Scratch Cards
FAMILY PLANNING
Done Here
M-PESA
Agent
MOBISTAR Com LTD.
NOPEN
BAMBA HAPA
Safaricom
.CURATIVE SERVICES
.PREGNANCY TEST
.PHOTO COPY

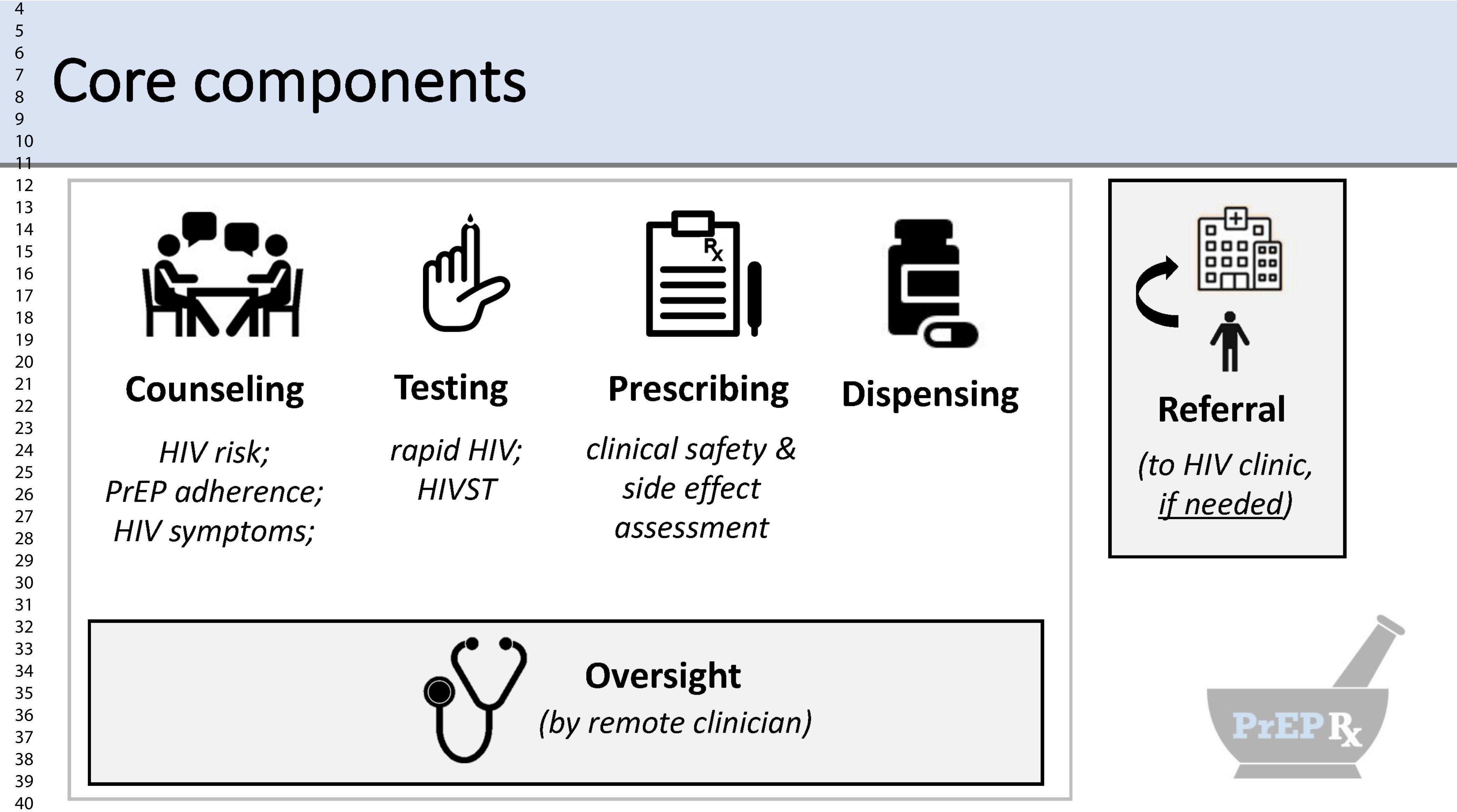
Core components
Counseling
HIV risk;
PrEP adherence;
HIV symptoms;
Testing
rapid HIV;
HIVST
Prescribing
clinical safety &
side effect
assessment
Dispensing
Referral
(to HIV clinic,
if needed)
Oversight
(by remote clinician)
PrEP Rx

PrEP use over time using retail pharmacies: Pilot study

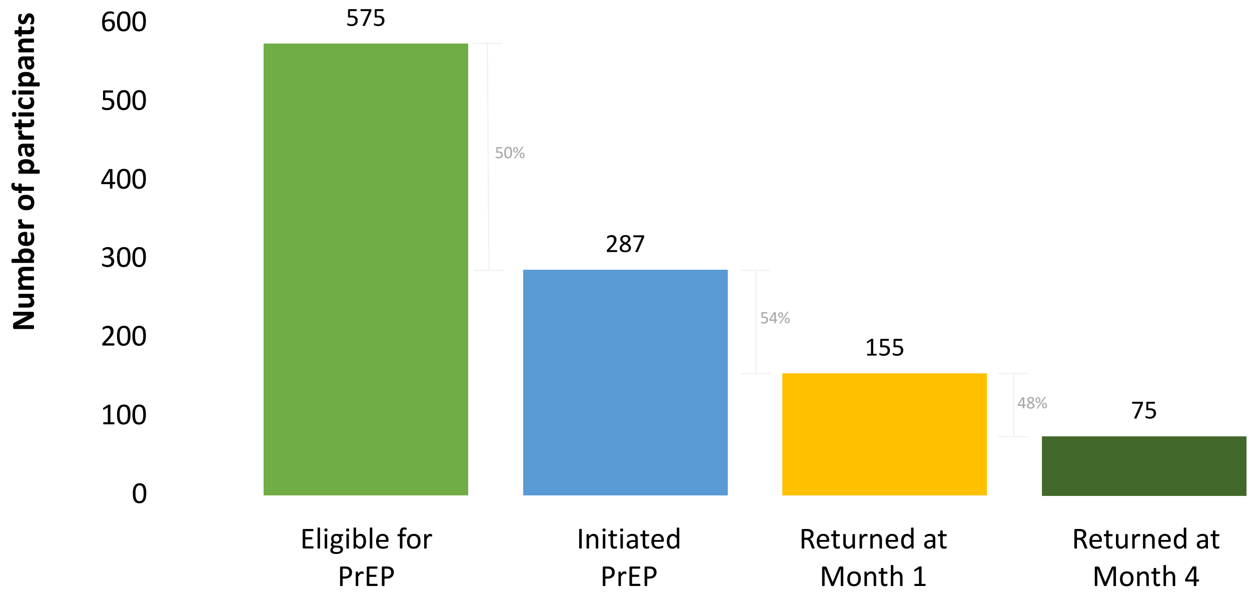

Challenges with delivering PrEP at retail pharmacies

1 PrEP services too **expensive** for some clients.

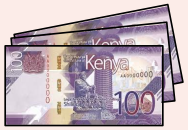

2 HIV risk assessment **time-consuming** & some clients **uncomfortable** doing it out loud.

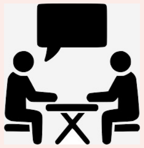

3 Some prospective clients **uncomfortable** testing for HIV at pharmacy, especially if unsure of status.

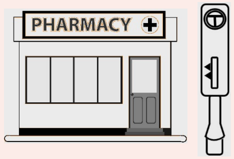

Can PrEP be delivered through E-pharmacies?

Possible benefits

- Convenient: Clients can get PrEP delivered to their home without having to go to a clinic or pharmacy
- Greater confidentiality (decreased stigma)

PrEP Rx

Steps for getting PrEP from E-pharmacy

1) HIV risk screening
2) HIV testing
3) Clinical consultation (medical assessment)
4) PrEP drugs delivered via courier
5) Ongoing user support (clients can talk to a provider to ask questions about their PrEP)

Step 1: HIV Risk assessment

Assessing HIV risk

Kenya Ministry of Health created the **HIV Risk Assessment Screening Tool (RAST)**

- Series of yes/no questions
- Sensitive topics (sex behaviors) – requires privacy & confidentiality

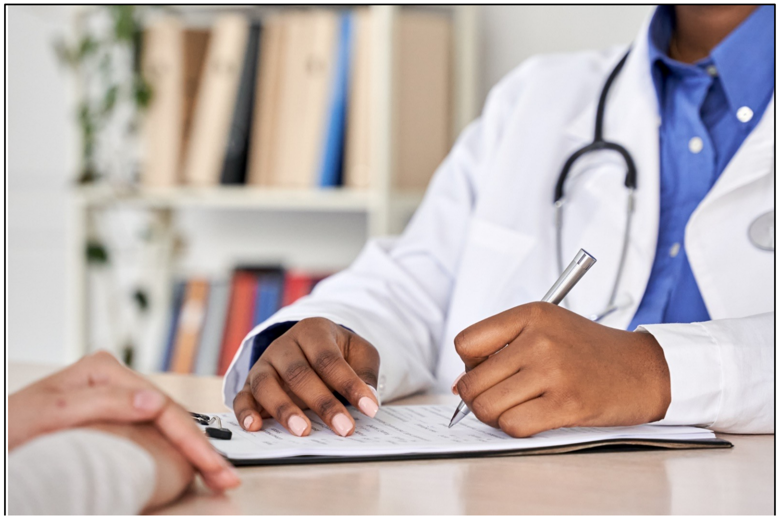

Assessing HIV risk

Example questions from the RAST

In the past 6 months, have you had sex without a condom with anyone?<br><br>*Select "yes" even if this only happened once in the past 6 months.*	☐ Yes	☐ No	☐ Unsure

Assessing HIV risk

Example questions from the RAST

In the past 6 months, have you had sex without a condom with anyone?<br><br>*Select "yes" even if this only happened once in the past 6 months.*	☐ Yes	☐ No	☐ Unsure
In the past 6 months, have you been diagnosed with or treated for a sexually transmitted infection (STI)?<br><br>*Examples of sexually transmitted infections include chlamydia and gonorrhea.*	☐ Yes	☐ No	☐ Unsure

Assessing HIV risk

Example questions from the RAST

In the past 6 months, have you had sex without a condom with anyone?<br><br>*Select "yes" even if this only happened once in the past 6 months.*	☐ Yes	☐ No	☐ Unsure
In the past 6 months, have you been diagnosed with or treated for a sexually transmitted infection (STI)?<br><br>*Examples of sexually transmitted infections include chlamydia and gonorrhea.*	☐ Yes	☐ No	☐ Unsure
Do you have any sex partners who are HIV-positive and:<br>• are not currently taking antiretroviral therapy (ART),<br>• have been taking antiretroviral therapy (ART) for less than 6 months,<br>• have been missing doses of their antiretroviral therapy (ART),<br>• have a detectable HIV viral load, or<br>• the two of you are trying to get pregnant?<br><br>*Select "yes" if any of the above statements are true about any of your sex partners who are HIV-positive.*	☐ Yes	☐ No	☐ Unsure

Assessing HIV risk

Example questions from the RAST

In the past 6 months, have you had sex without a condom with anyone?<br><br>*Select "yes" even if this only happened once in the past 6 months.*	☐ Yes	☐ No	☐ Unsure
In the past 6 months, have you been diagnosed with or treated for a sexually transmitted infection (STI)?<br><br>*Examples of sexually transmitted infections include chlamydia and gonorrhea.*	☐ Yes	☐ No	☐ Unsure
Do you have any sex partners who are HIV-positive and:<br>• are not currently taking antiretroviral therapy (ART),<br>• have been taking antiretroviral therapy (ART) for less than 6 months,<br>• have been missing doses of their antiretroviral therapy (ART),<br>• have a detectable HIV viral load, or<br>• the two of you are trying to get pregnant?<br><br>*Select "yes" if any of the above statements are true about any of your sex partners who are HIV-positive.*	☐ Yes	☐ No	☐ Unsure

If client answers "yes" to any RAST question, s/he is considered to have substantial ongoing risk of HIV.

Step 2: HIV testing

Importance of HIV testing

- PrEP is for HIV **prevention**, not treatment.
- Therefore, PrEP is only for **HIV-negative** individuals.
  - HIV-*positive* individuals should take ART, not PrEP.
  - Starting an HIV-*positive* individual on PrEP could make their HIV virus harder to treat.

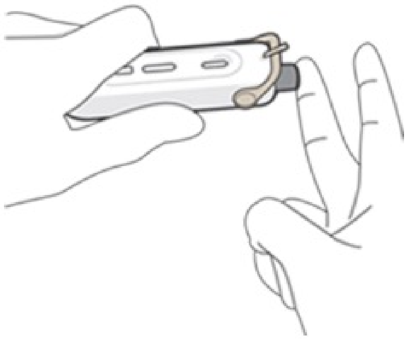

So before starting someone on PrEP, it is critical to confirm that they are HIV-negative.

PrEP users must also get ongoing periodic HIV testing to confirm that they are still HIV-negative (usually every 1-3 months.

HIV testing schedule

Kenya national PrEP guidelines

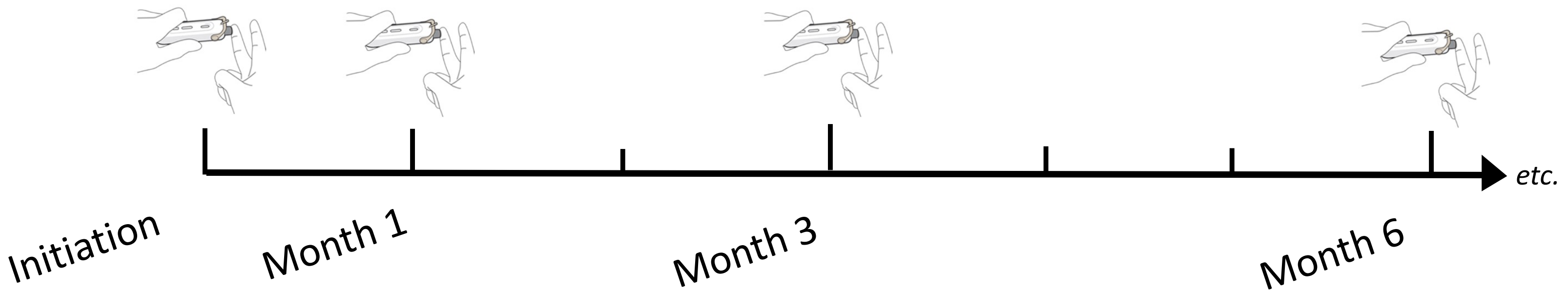

3 options for HIV testing

Oral-fluid HIVST

(OraQuick)

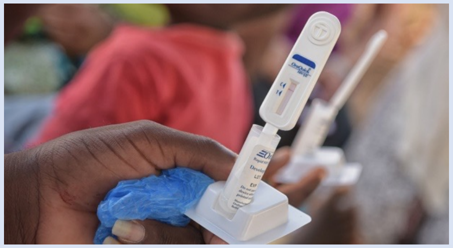

- **Sensitivity:** 92%
- **Specificity:** 99.9%

(i.e., one false negative out of every 12 test results in HIV infected individuals)

Blood-based HIVST

(Sure Check)

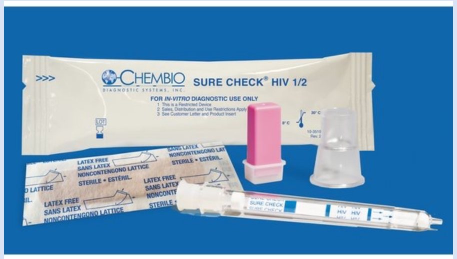

- **Sensitivity:** 99.7%
- **Specificity:** 99.9%

(i.e., one false negative out of every 333 test results in HIV infected individuals)

Rapid diagnostic testing

(Alere Determine)

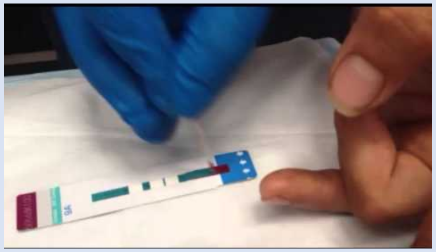

- **Sensitivity:** 99.9%
- **Specificity:** 99.6%

What is HIVST?

A process whereby an individual collects his or her own specimen (oral fluid or blood), performs an HIV rapid diagnostic test and interprets the result, often in a private setting, either alone or with someone she trusts.

Benefits:

- Promotes access to HIV testing services
- Increases autonomy
- Assures confidentiality/privacy
- Empowers
- Convenient

Approved HIVST kits for use in Kenya

Oraquick HIV Self-Test
(Oral fluid based)

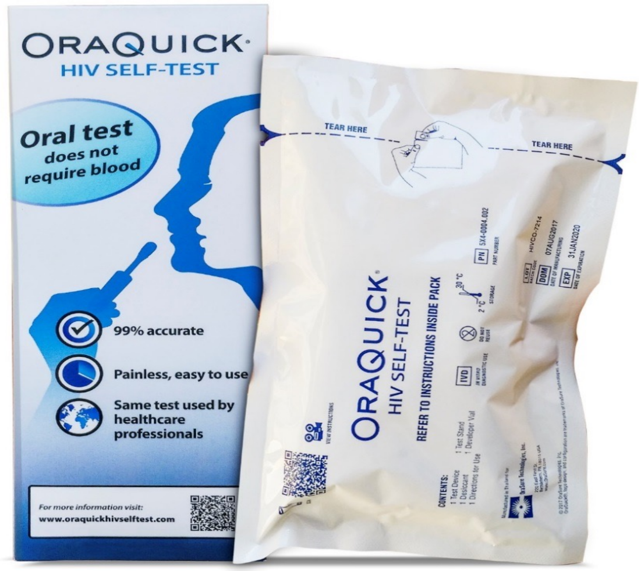

Insti HIV Self-Test
(Blood based)

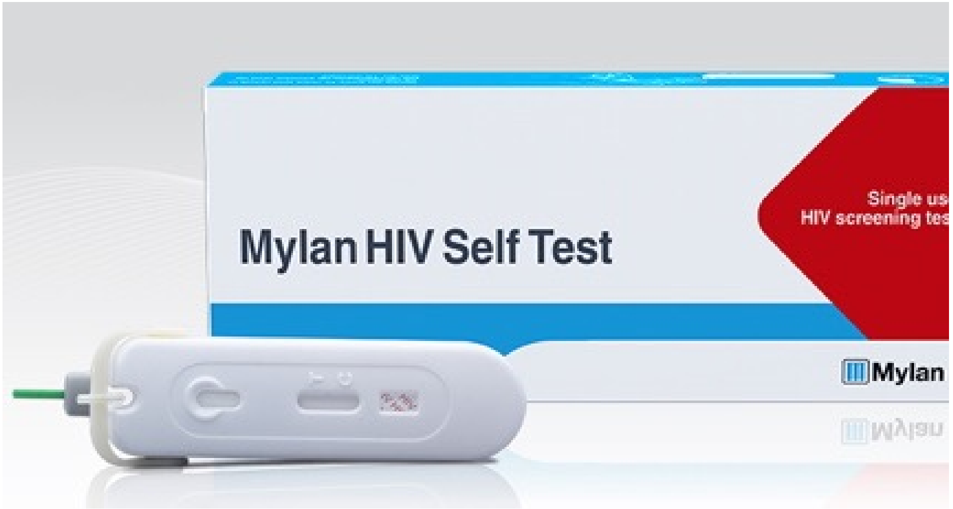

HIV self-testing for e-pharmacy PrEP initiation

Blood-based HIVST

(Sure Check)

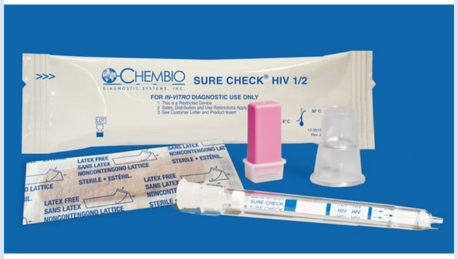

Client completes unassisted; provider interprets

Step 3: Clinical consultation

Medical safety assessment

Signs & symptoms of acute HIV infection?	☐ Yes	☐ No
Current or past liver disease?	☐ Yes	☐ No
Current or past kidney disease?	☐ Yes	☐ No
Diabetic?	☐ Yes	☐ No

Medical safety assessment

Signs & symptoms of acute HIV infection?	☐ Yes	☐ No

Provider asks the client if they have had any high-risk sexual contact **in past month** and subsequently experienced any of the following:

- Fever
- Sore throat
- Muscle or joint pains
- Swollen glands
- Diarrhoea
- Headache

If client answers "yes", provider does not prescribe PrEP. Refers client to clinic.

Medical safety assessment

Current or past liver disease?	☐ Yes	☐ No

Asks if client has ever had problems with their liver, such as hepatitis, cancer, or cirrhosis.

If client answers "yes", provider does not prescribe PrEP. Refers client to clinic.

Medical safety assessment

Current or past kidney disease?	☐ Yes	☐ No

Asks if client has ever had problems with their kidneys, such as acute or chronic kidney injury or kidney failure.

If client answers "yes", provider does not prescribe PrEP. Refers client to clinic.

Medical safety assessment

Diabetic?	☐ Yes	☐ No

If client is diabetic, provider does not start PrEP.

- Diabetes is a risk factor for kidney disease. So diabetic clients must be evaluated for kidney disease before starting PrEP.

Provider refers client to clinic

Step 4: PrEP delivery via courier

PrEP delivery

- Delivered by courier to location of client's choice (e.g. home or other location)
- Discreet packaging

Step 5: Ongoing user support

User support

- Client can ask a provider questions about PrEP use, side effects, etc
- Options for user support
    - Phone calls
    - WhatsApp/SMS
    - Email

For peer review only - http://bmjopen.bmj.com/site/about/guidelines.xhtml

Pilot study of E-pharmacy PrEP delivery

Online model: pilot study

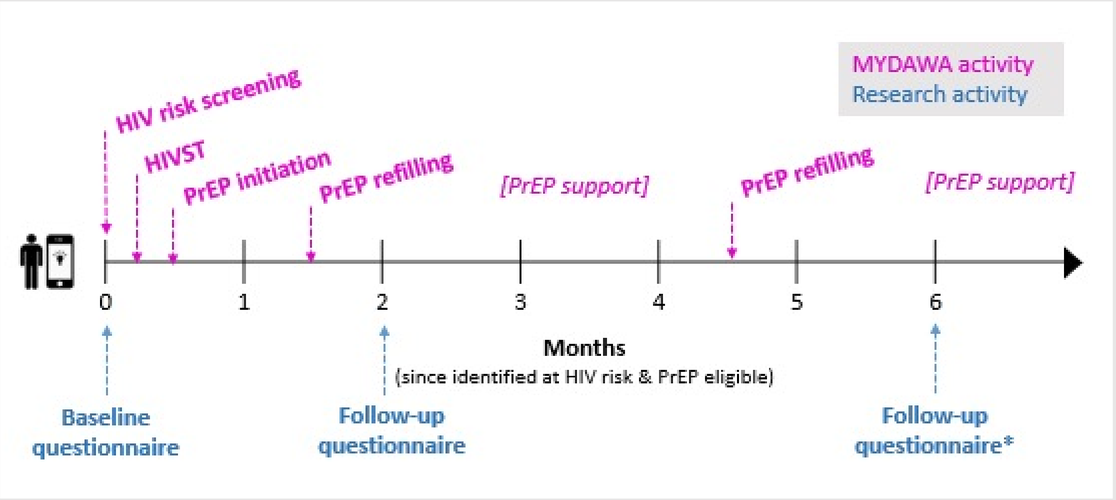

**Client characteristics**	**Demographics**	Month: 0
	**HIV risk profile**	Months: 0 & 2
	**History of PrEP use**	Month: 0
	**Contraceptive use**	Month: 0
**Effectiveness outcomes**	**PrEP initiation** *(primary)*	Month: 2
	**PrEP continuation**	Months: 2 & 6
**Process outcomes**	**Awareness of online PrEP**	Month: 0
	**Modality of HIV risk screening**	Month: 0
	**HIVST uptake**	Month: 2
	**Chatbot/clinical officer use**	N/A
	**HIVST/PrEP delivery setting**	N/A
	**Selection of support reminders**	N/A

What are client preferences for E-Pharmacy PrEP delivery?

- Risk assessment
- Clinical consultation
- Type of HIV test
- User support
- Cost

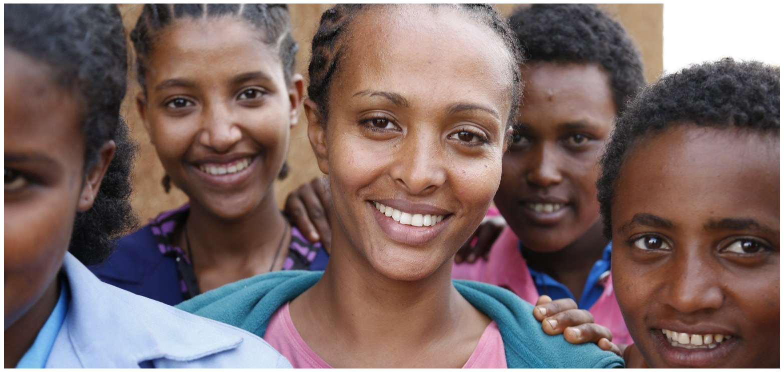

Understanding client preferences can help increase PrEP coverage among those at risk of HIV

Discrete Choice Experiments (DCEs)

- Quantitative method used to understand participants preferences for a service without directly asking them to state their options.
- Participants given a series of alternative hypothetical scenarios with variables or “attributes”, each with variations or “levels” and asked to choose their preferred scenario.
- Surveys usually contain 5-10 scenarios.
- Increasingly used in healthcare settings
- Especially useful for new interventions that are not currently available

Discrete-Choice Experiments

- Participants are asked to make choices between different hypothetical services
- Example: HIVST distribution
- Pictures are used to describe each option

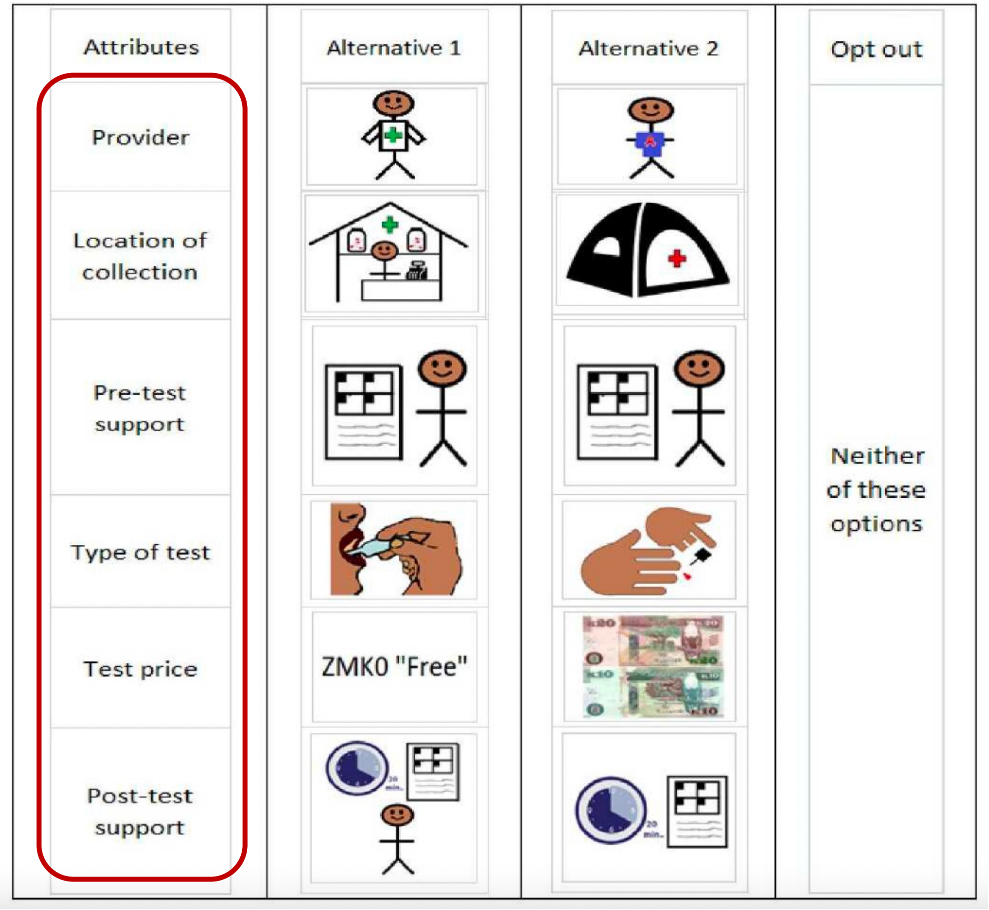

Discrete-Choice Experiments

- Participants are asked to make choices between different hypothetical services
- Each service is defined by attributes that can take on different levels
- Experimental design determines
  - service
  - service pairs
  - And series of choice questions

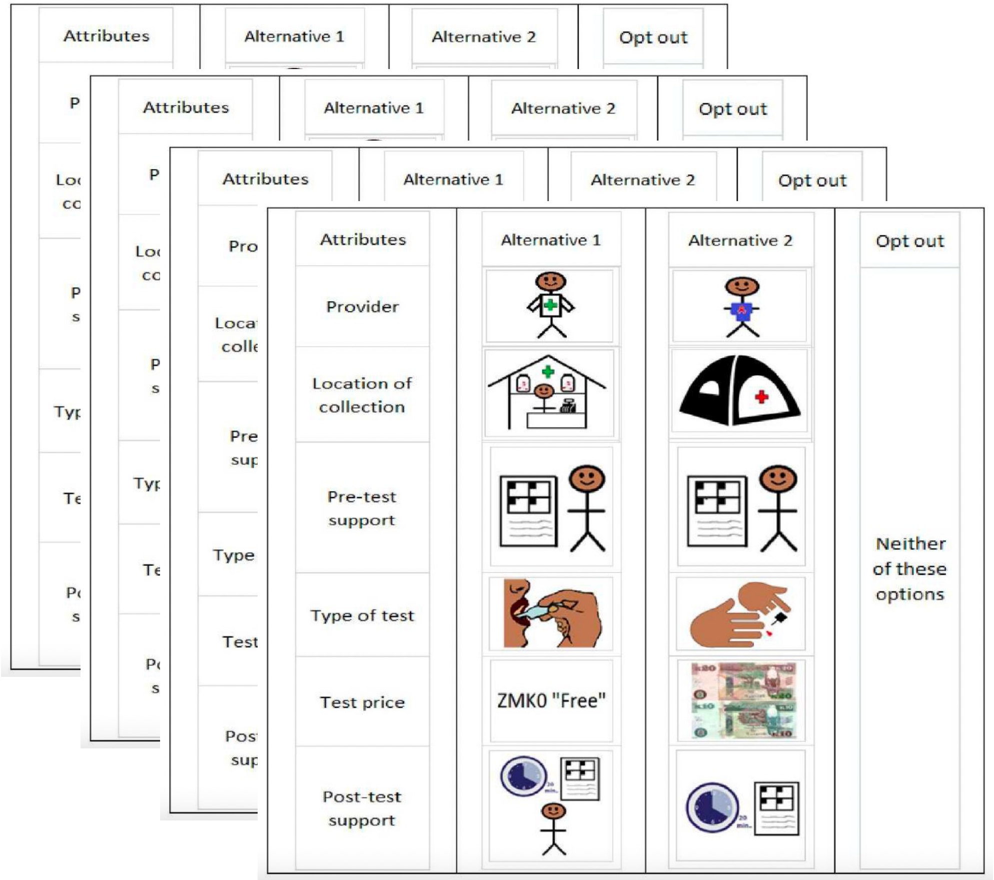

Why use a DCE instead of a qualitative interview?

- Qualitative interviews to understand preferences for straightforward decisions but DCEs are useful for more complex interventions. DCEs provide quantitative estimate of preferences, estimate trade-offs between preferences

For peer review only - http://bmjopen.bmj.com/site/about/guidelines.xhtml

DCE Example: Adolescent attribute preferences for long-acting PrEP

Minnis et al. Attribute preferences for long acting PrEP: Results of a Discrete Choice Experiment with South African Youth. R4P 2018

**Attribute**	**Levels**			
**Product form**	Implant 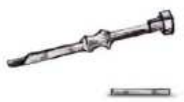	One Injection 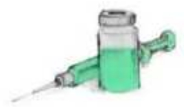	Two Injections 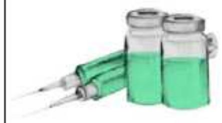	
**Duration** – how often you will need to a get a dose	One dose every 2 months 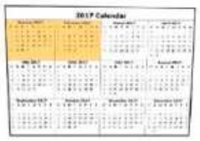	One dose every 6 months 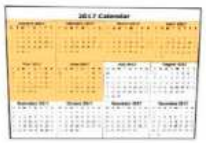	One dose every year 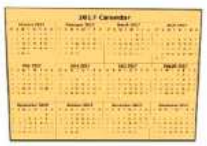	
**Where it is available**	Pharmacy 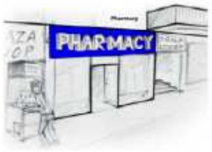	Health clinic 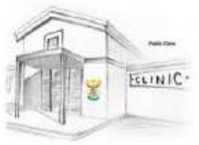	Mobile clinic (like Tutu Testers) 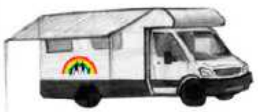	Community site (like a library or community hall) 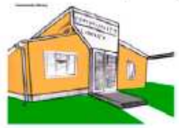
**Soreness** after injection /implant	Mild 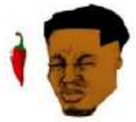	Moderate 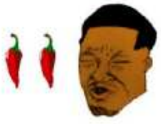		
**Location** of injection/ implant	Bum 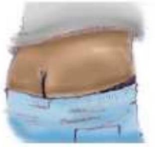	Thigh 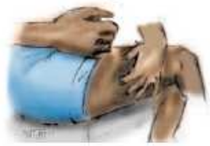	Upper Arm 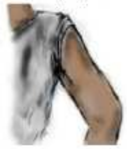	

DCE Survey

Example Question

Assume you are given two HIV prevention products, **A** and **B**.

Please compare their characteristics and choose the one you would prefer to use.

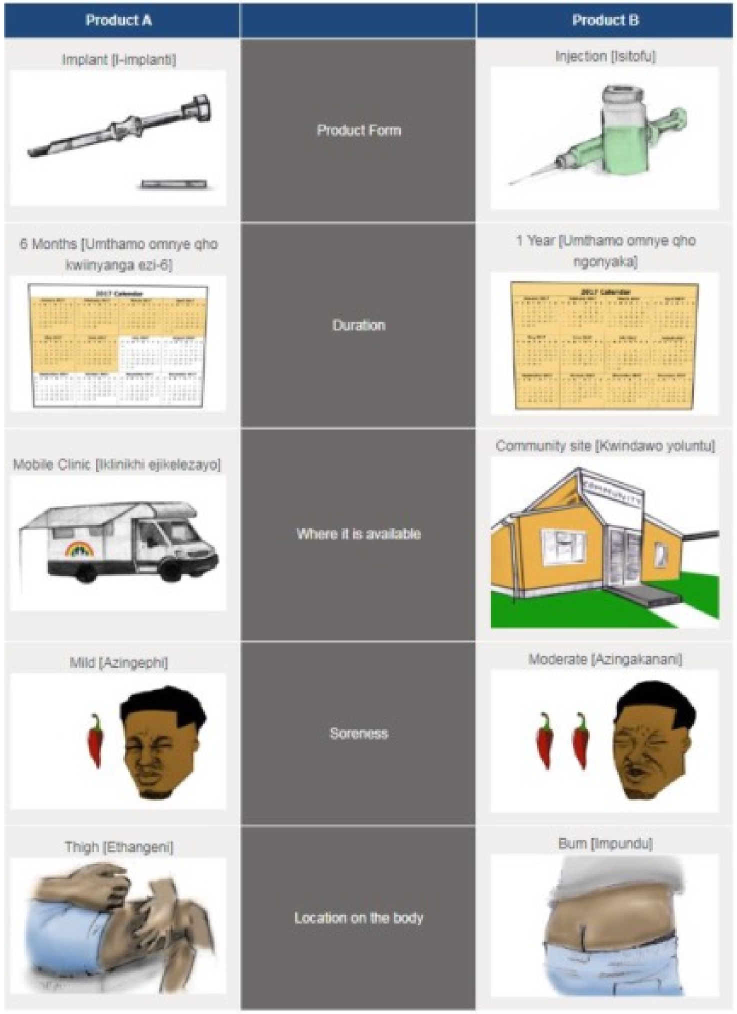

Analysis

Can quantify preferences to determine most important characteristics

Can stratify by participant type

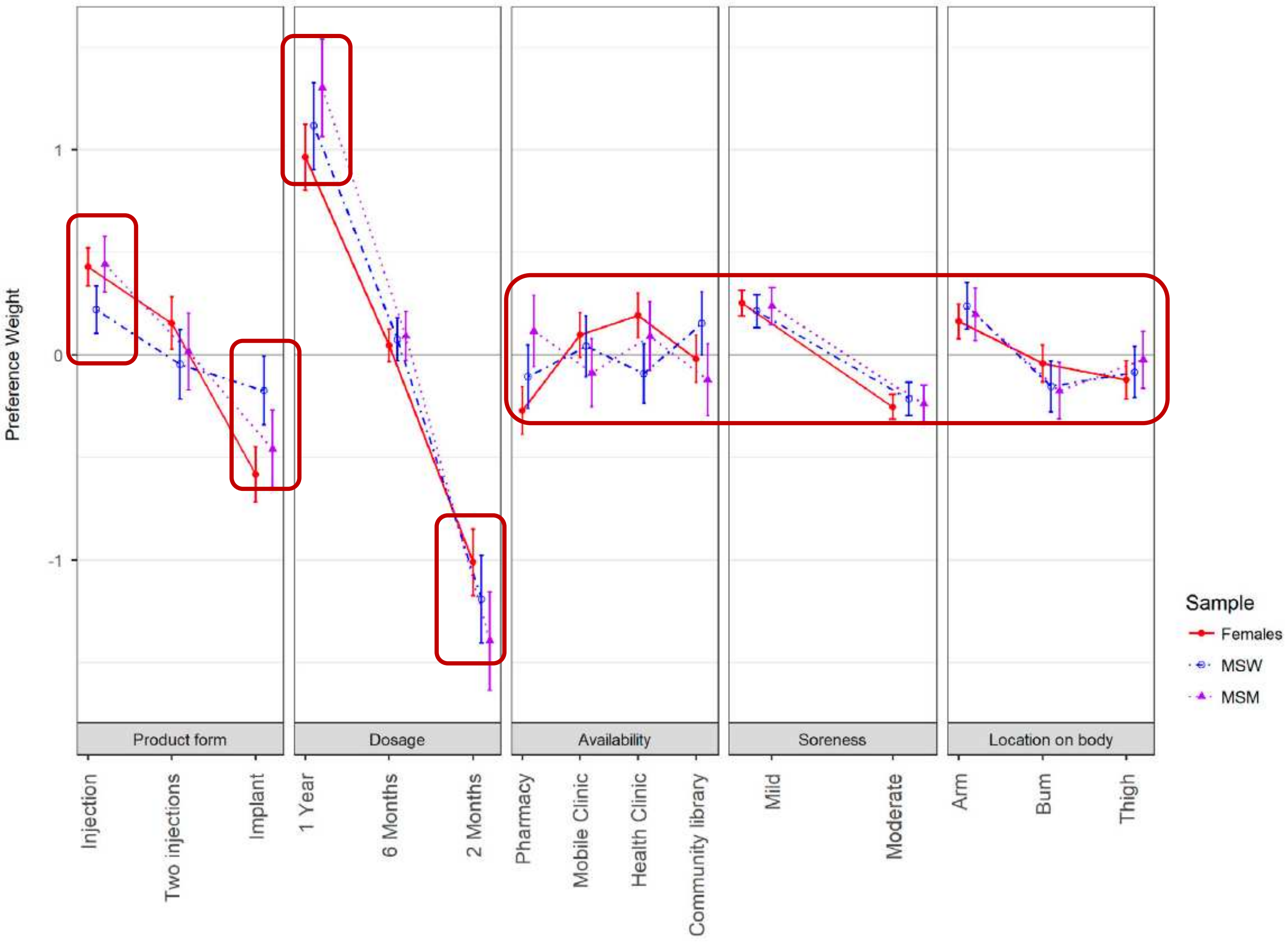

Using DCEs to quantify trade-offs

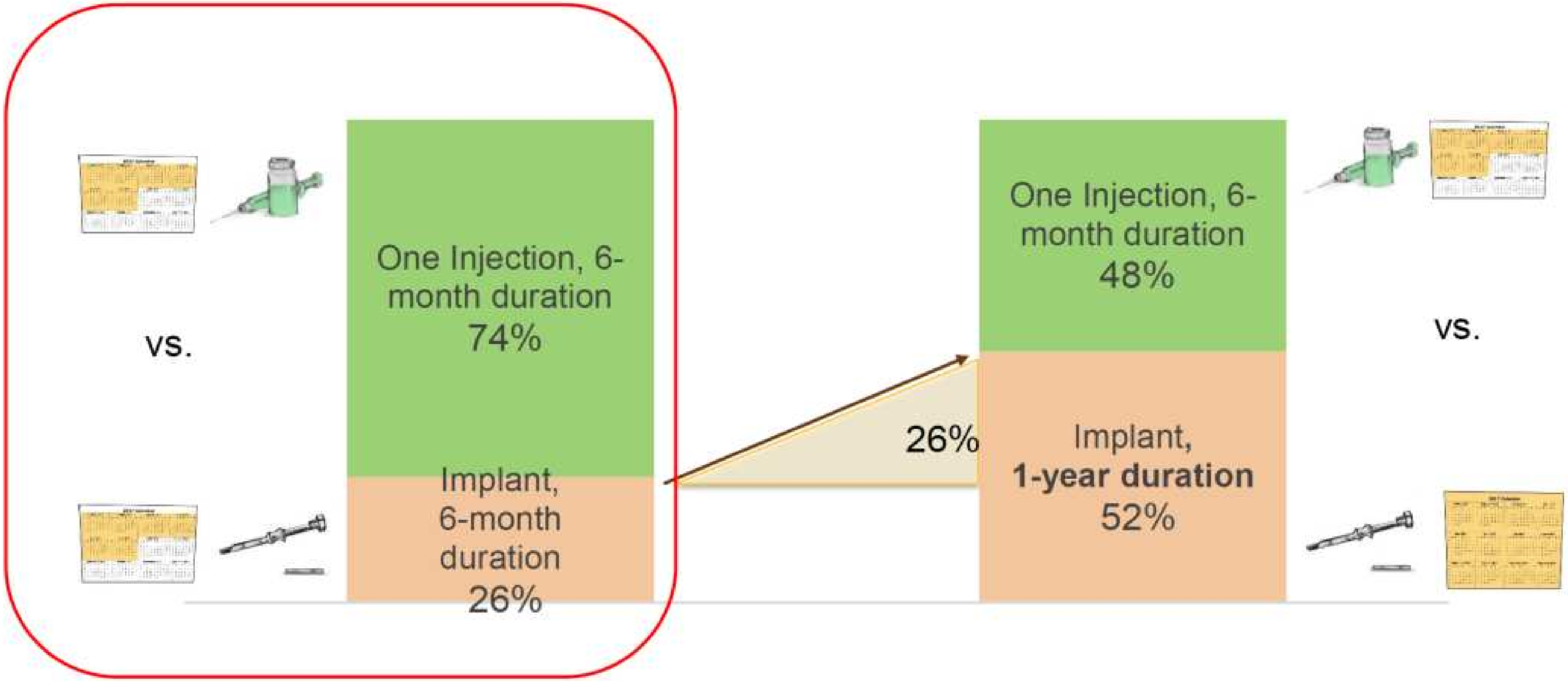

Steps for Conducting a DCE

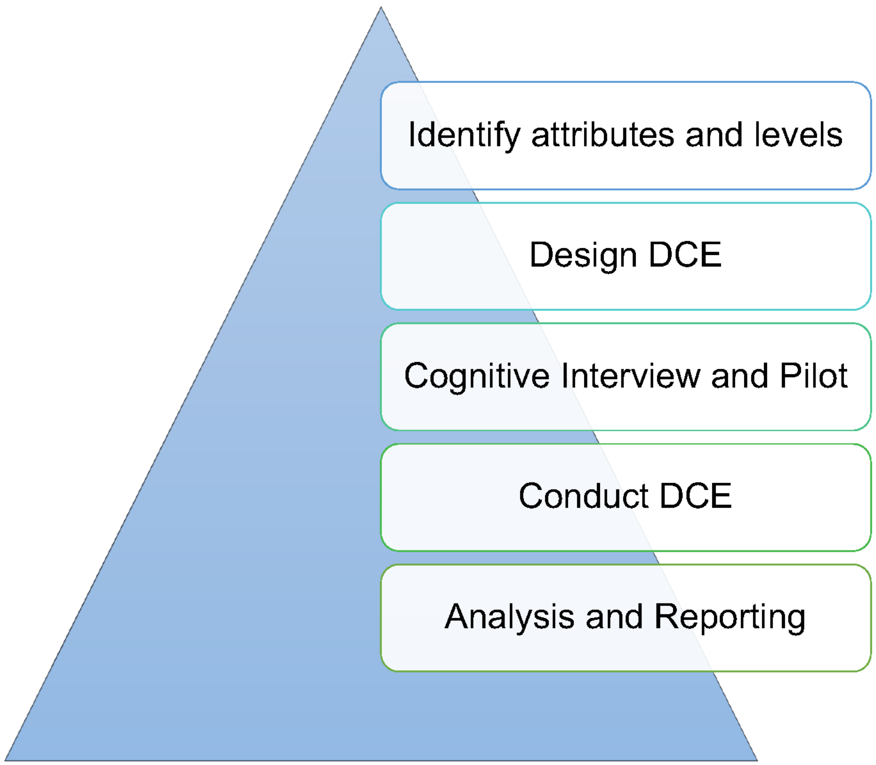

Attributes and Levels of ePharm DCE

Attribute	Description	Levels
**PrEP eligibility assessment**	Method for conducting client eligibility assessment for PrEP	• Online self-assessment using screening questions (phone number in case of questions)<br>• Guided assessment with a remote clinical provider (via a phone call or WhatsApp)
**HIV test type**	Type of HIV test delivered for PrEP initiation	• Oral Fluid HIV self-test (at setting of your choice)<br>• Blood-based HIV self-test (at setting of your choice)<br>• Healthcare provider administers HIV Test at setting of your choice (blood-based)
**Clinical consultation for prescribing PrEP**	Clinical consultation needed to prescribe PrEP	• Remote clinical consultation with provider (via a phone call or video chat)<br>• In-person clinical consultation with provider after completing HIV test (at a setting of your choice)
**User support options for PrEP**	Method for discussing your questions for PrEP with a healthcare provider	• SMS<br>• Phone/video call<br>• Email
**Cost of PrEP delivery**	Total cost of the PrEP delivery visit (including HIV self-test delivery and support, the remote clinician consultation, and PrEP delivery (one month of PrEP)	• 500 KES<br>• 2000 KES<br>• 4000 KES

Questions?

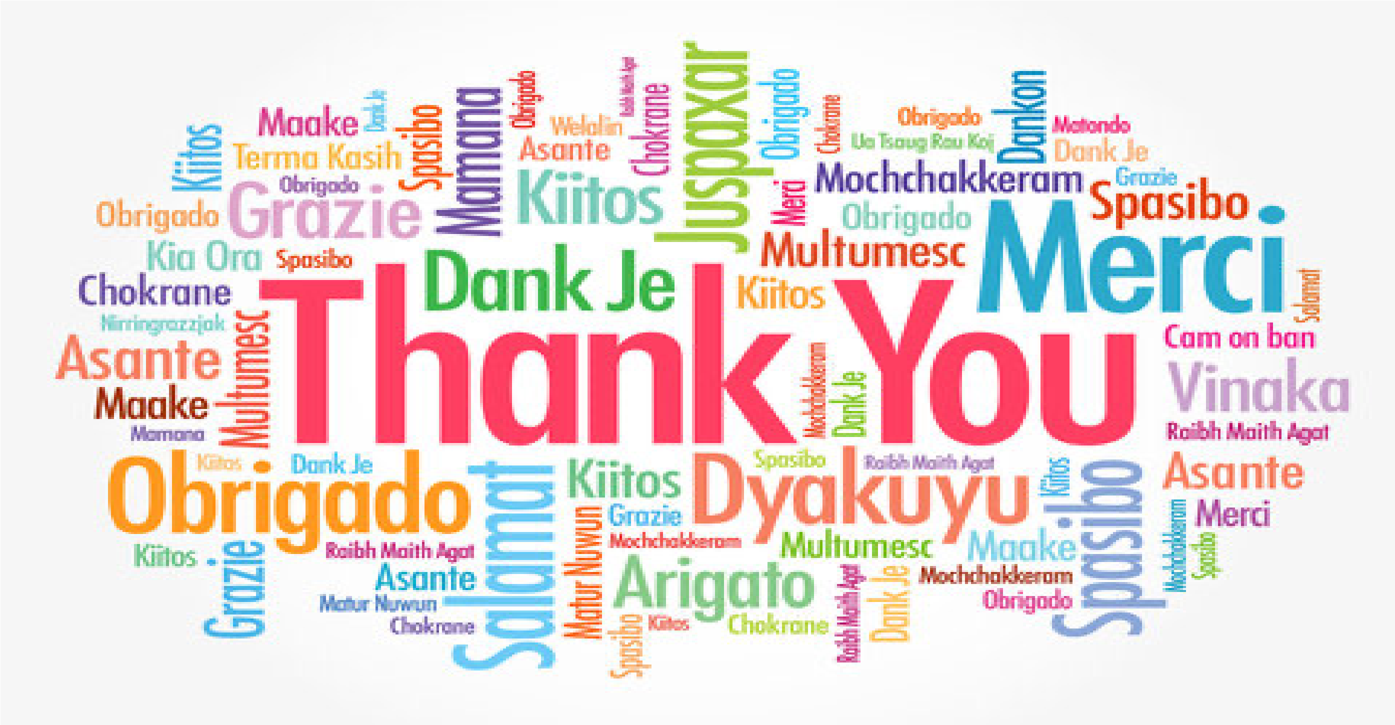

msharma1@uw.edu

Virtual PrEP delivery model

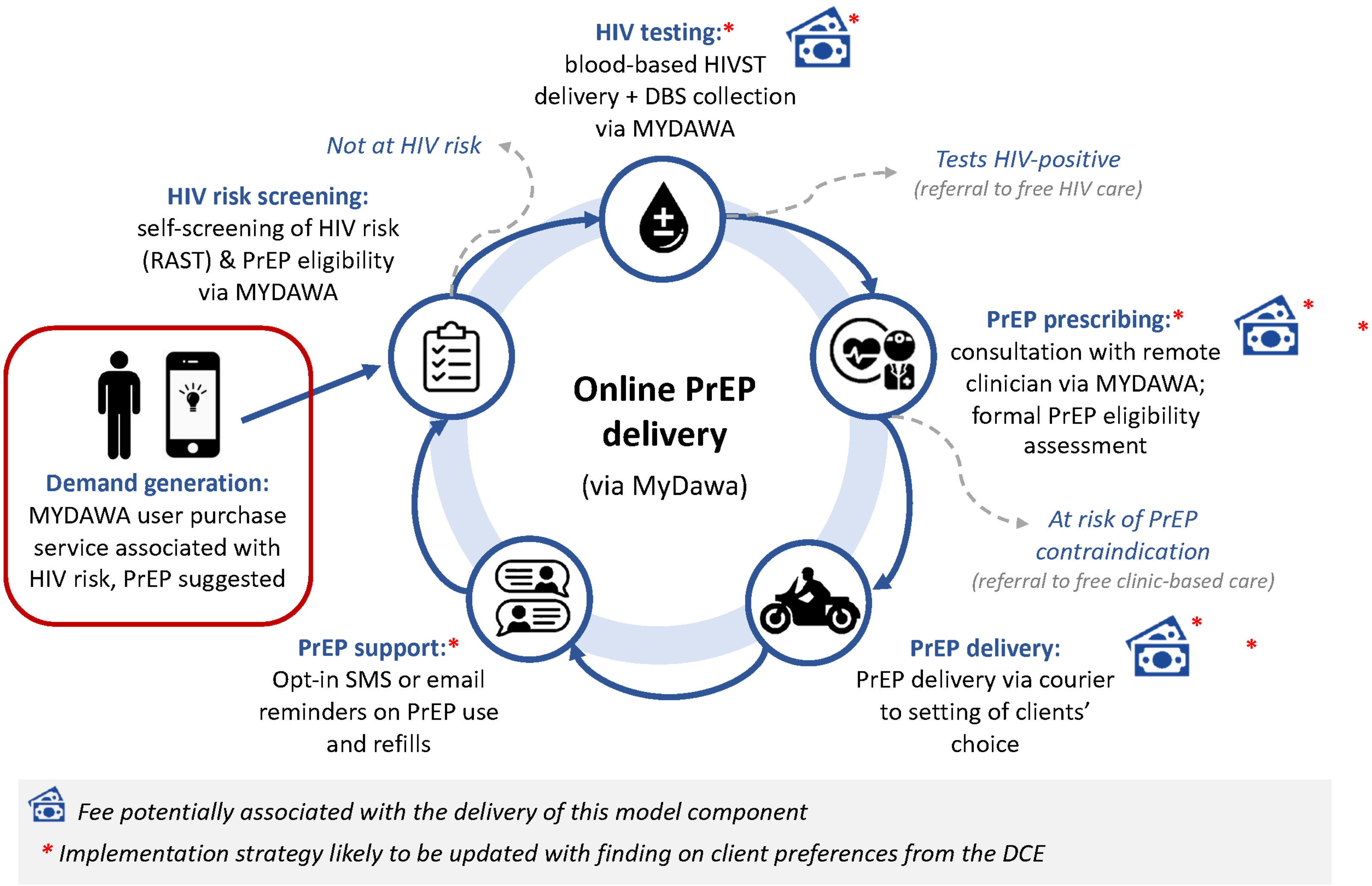

K. Ortblad 2021 ©

EPharmacy PrEP: Pilot Study Discrete Choice Experiment Training

Day 1 Session 2

24th-25th January 2022

For peer review only - http://bmjopen.bmj.com/site/about/guidelines.xhtml

Overview of study procedures

- Recruitment
- Pre-screening
- Consent
- Questionnaire administration (in-person)

Recruitment

- Target MYDAWA clients accessing products related to HIV/sexual health
- Banner ads on MYDAWA website, flyers included with product deliveries
- Potential participants will call study phone number or SMS/WhatsApp with the word #GenN if interested

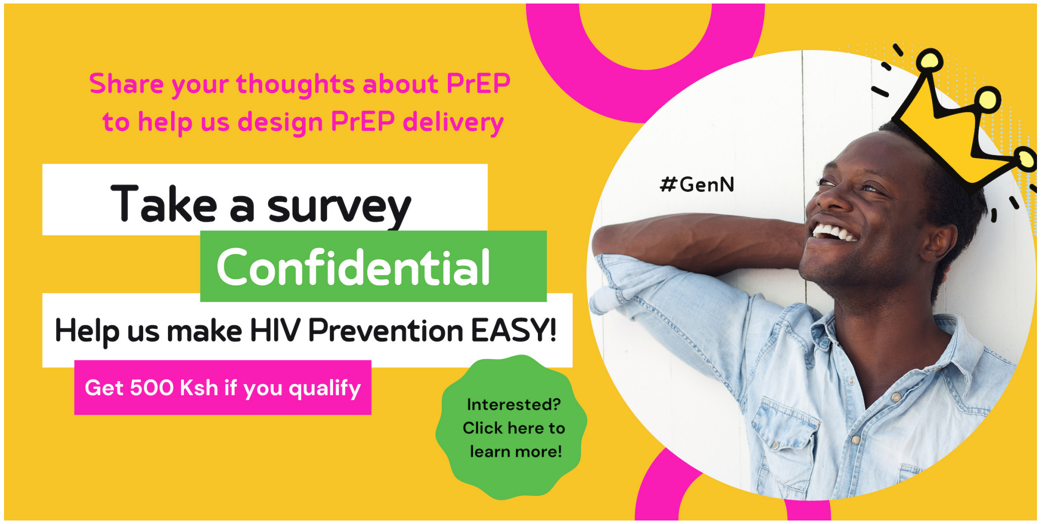

Pre-screening

- Pre-screening will take place by phone
- Study staff will complete REDCap pre-screening data entry form
    1. Eligibility assessment
    2. Schedule in-person visit

Eligibility criteria

- Participants are eligible to enroll in the study if they are:
  1. Age 18 or older
  2. HIV-negative or unknown HIV status
  3. Respond ‘yes’ to at least one of eleven screening questions about HIV risk
  4. Able and willing to provide informed consent

Eligibility Assessment

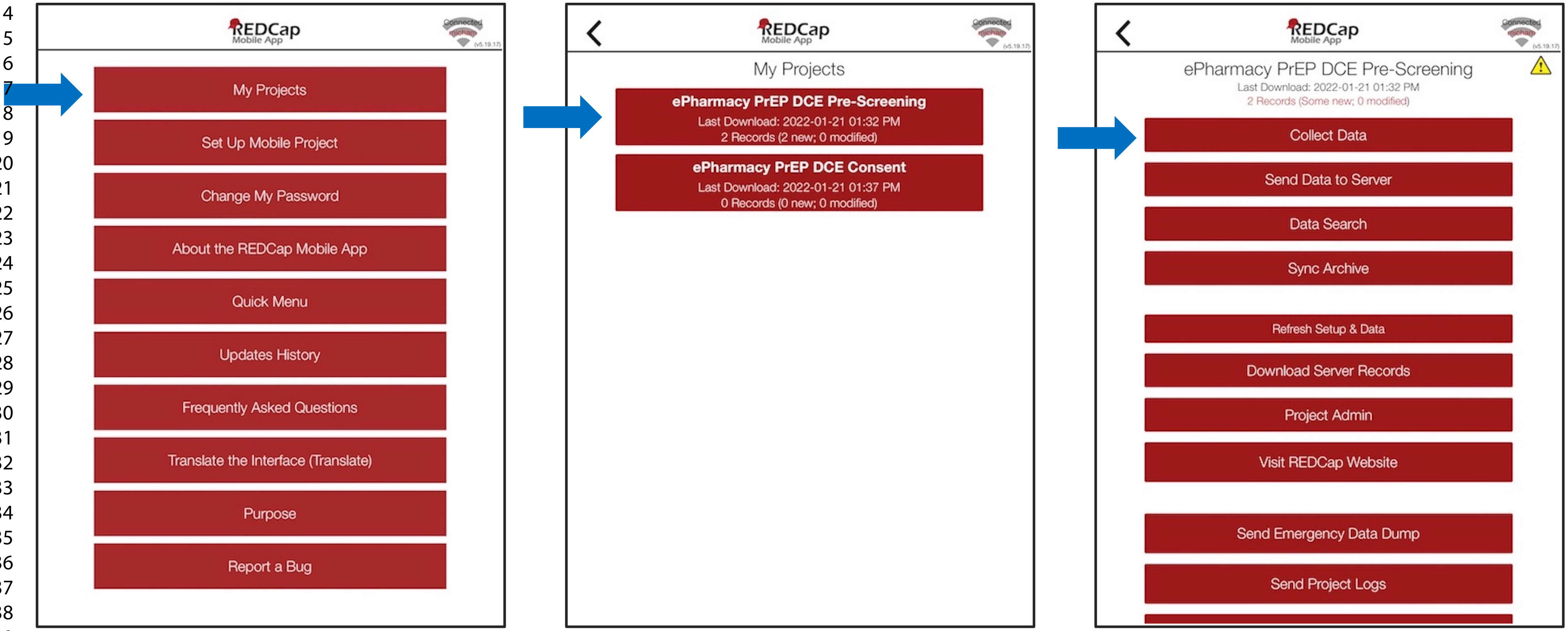

Eligibility Assessment

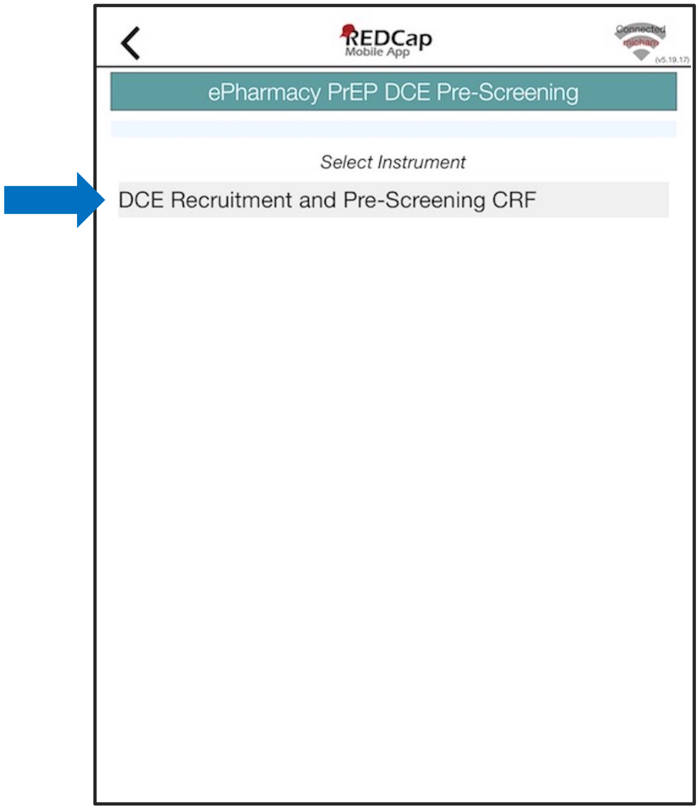

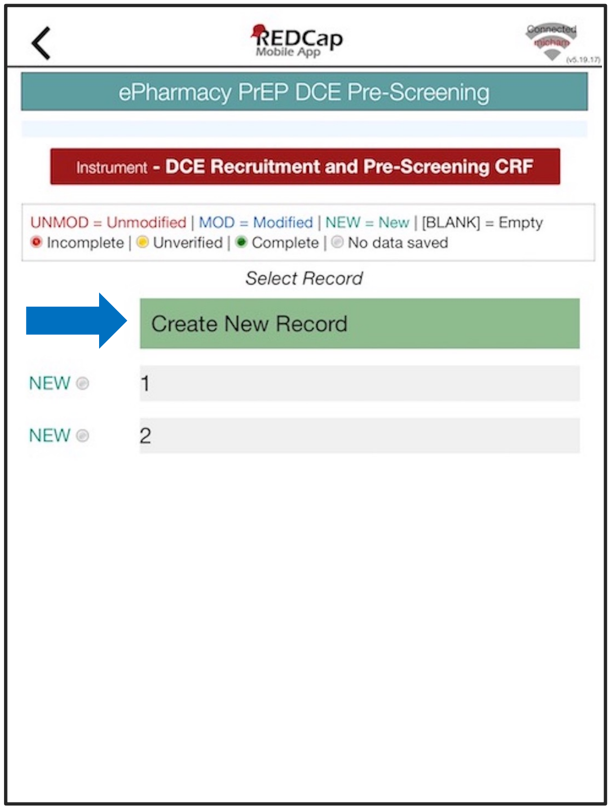

Eligibility Assessment

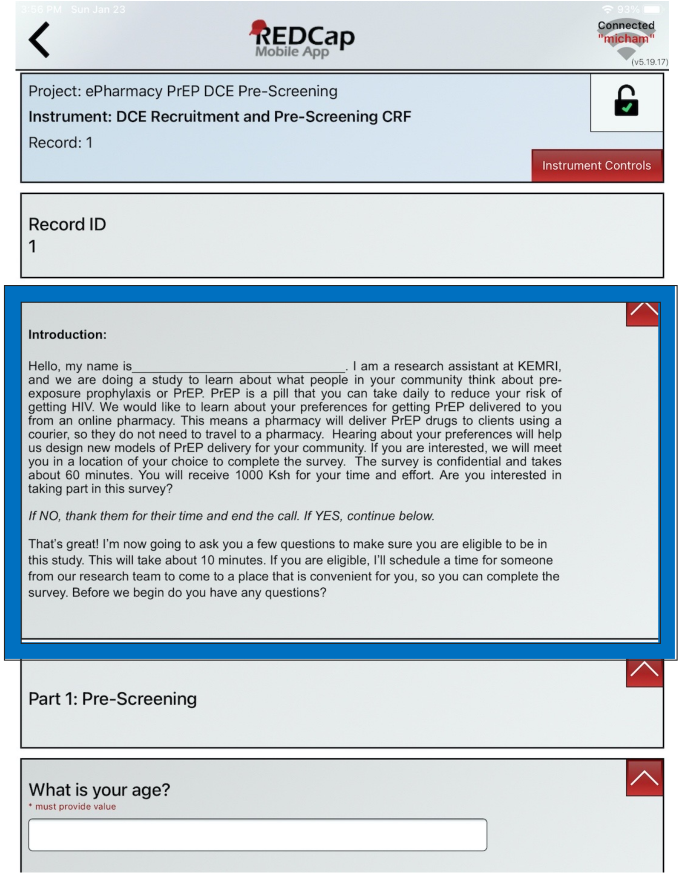
REDCap Mobile App

Connected "micham" (v5.19.17)

Project: ePharmacy PrEP DCE Pre-Screening

Instrument: DCE Recruitment and Pre-Screening CRF

Record: 1

Instrument Controls

Record ID
1

Introduction:

Hello, my name is______________________________. I am a research assistant at KEMRI, and we are doing a study to learn about what people in your community think about pre-exposure prophylaxis or PrEP. PrEP is a pill that you can take daily to reduce your risk of getting HIV. We would like to learn about your preferences for getting PrEP delivered to you from an online pharmacy. This means a pharmacy will deliver PrEP drugs to clients using a courier, so they do not need to travel to a pharmacy. Hearing about your preferences will help us design new models of PrEP delivery for your community. If you are interested, we will meet you in a location of your choice to complete the survey. The survey is confidential and takes about 60 minutes. You will receive 1000 Ksh for your time and effort. Are you interested in taking part in this survey?

If NO, thank them for their time and end the call. If YES, continue below.

That's great! I'm now going to ask you a few questions to make sure you are eligible to be in this study. This will take about 10 minutes. If you are eligible, I'll schedule a time for someone from our research team to come to a place that is convenient for you, so you can complete the survey. Before we begin do you have any questions?

Part 1: Pre-Screening

What is your age?
* must provide value

Eligibility Assessment

Introduction:

Hello, my name is_______________________________. I am a research assistant at KEMRI, and we are doing a study to learn about what people in your community think about pre-exposure prophylaxis or PrEP. PrEP is a pill that you can take daily to reduce your risk of getting HIV. We would like to learn about your preferences for getting PrEP delivered to you from an online pharmacy. This means a pharmacy will deliver PrEP drugs to clients using a courier, so they do not need to travel to a pharmacy. Hearing about your preferences will help us design new models of PrEP delivery for your community. If you are interested, we will meet you in a location of your choice to complete the survey. The survey is confidential and takes about 60 minutes. You will receive 1000 Ksh for your time and effort. Are you interested in taking part in this survey?

If NO, thank them for their time and end the call. If YES, continue below.

That's great! I'm now going to ask you a few questions to make sure you are eligible to be in this study. This will take about 10 minutes. If you are eligible, I'll schedule a time for someone from our research team to come to a place that is convenient for you, so you can complete the survey. Before we begin do you have any questions?

Eligibility Assessment – Not eligible

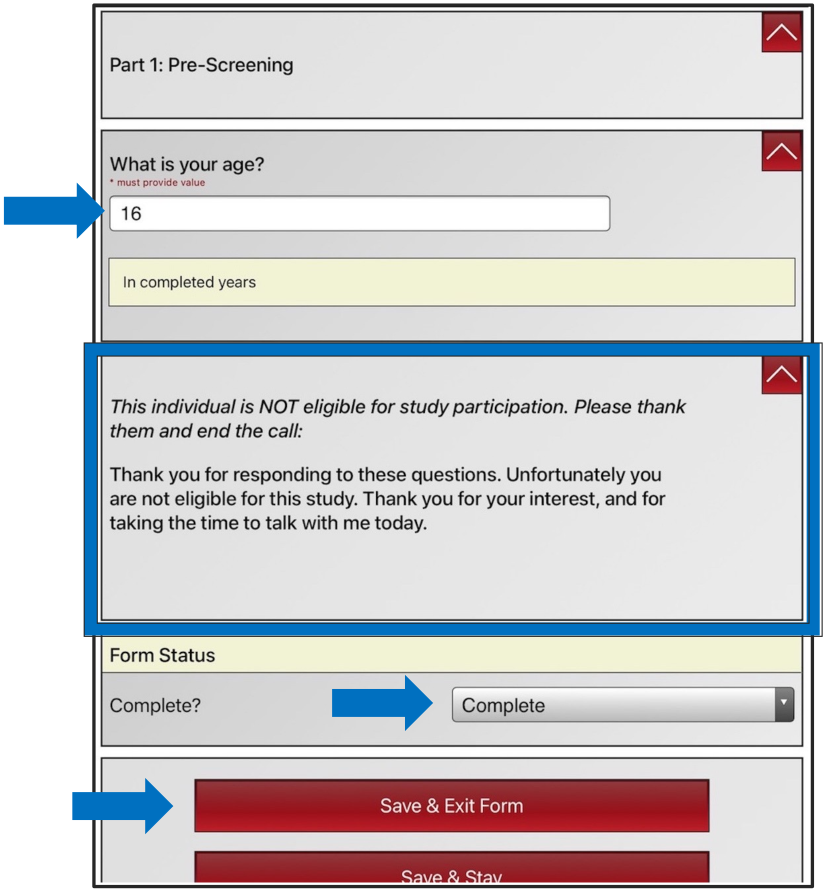

Eligibility Assessment – Not eligible

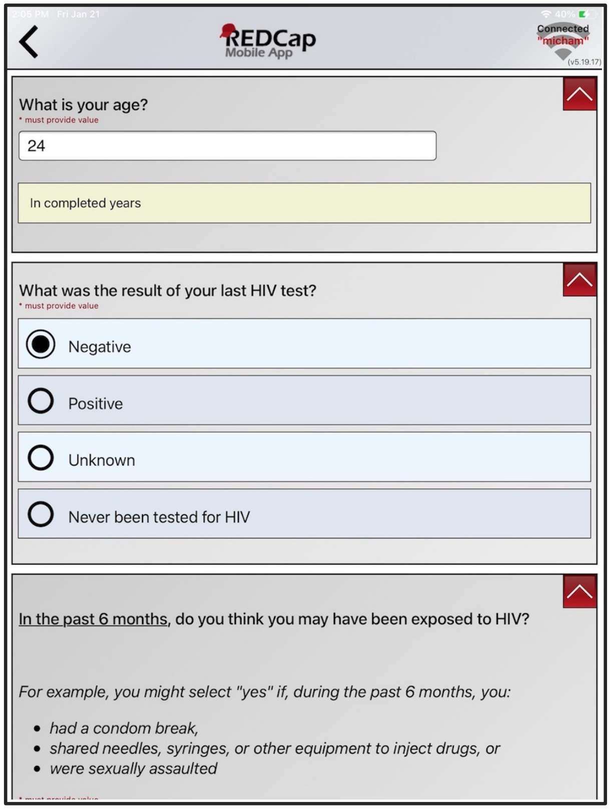

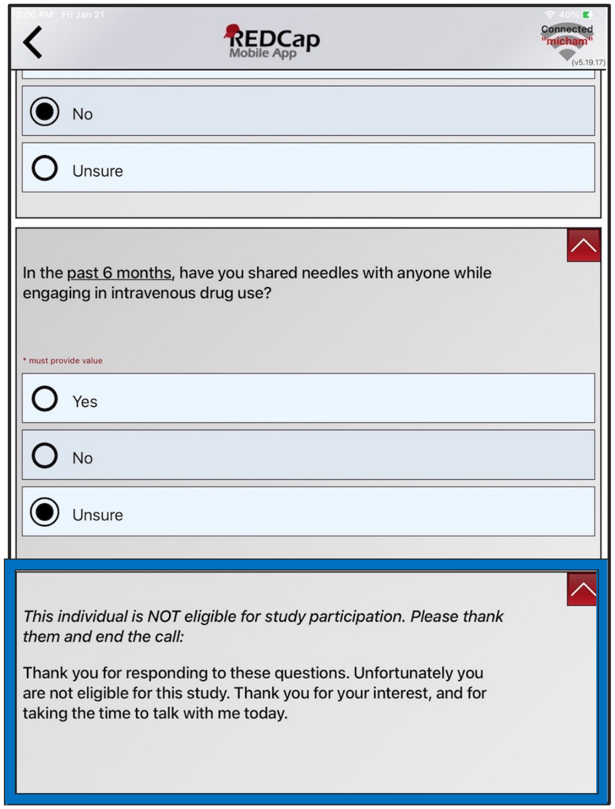

Eligibility Assessment – Eligible

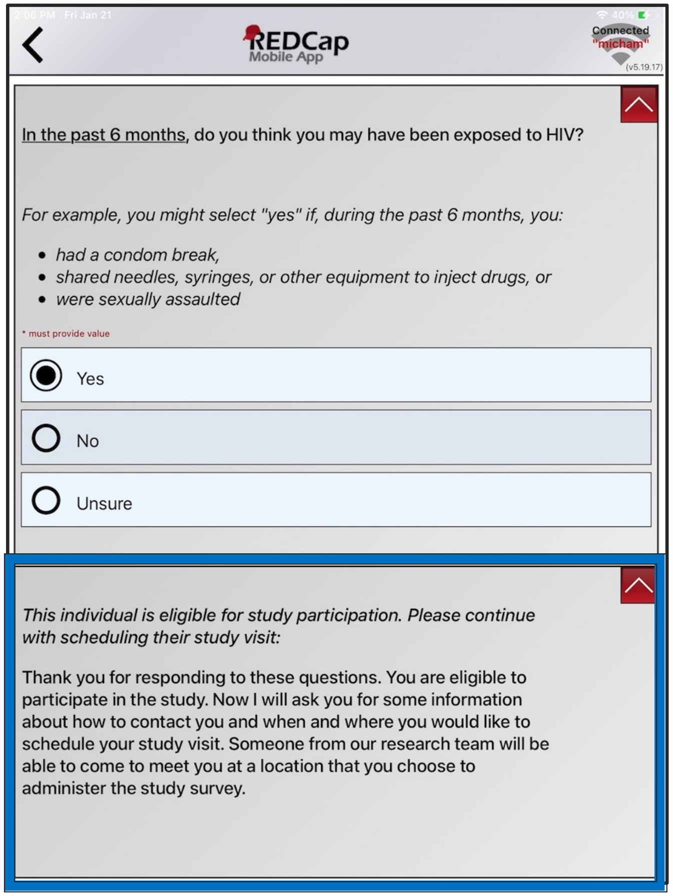

Eligibility Assessment – Scheduling and Contact Info

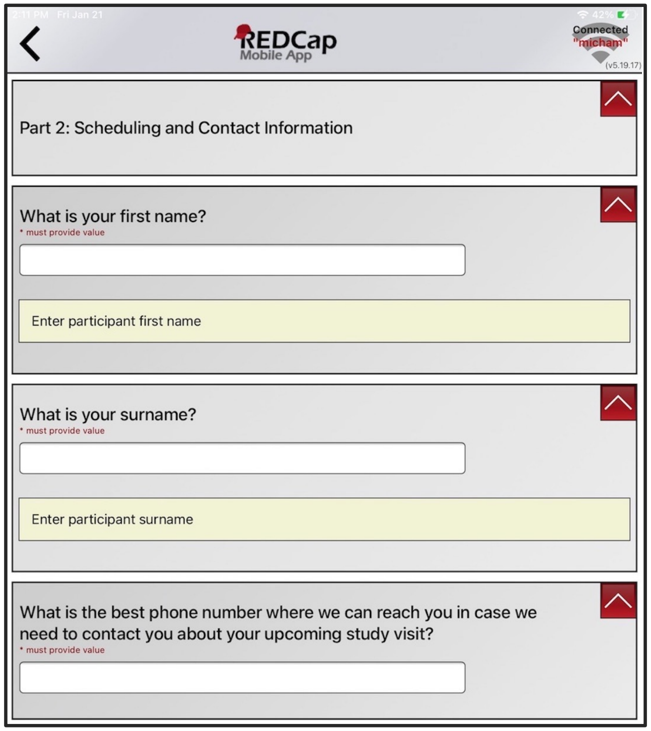

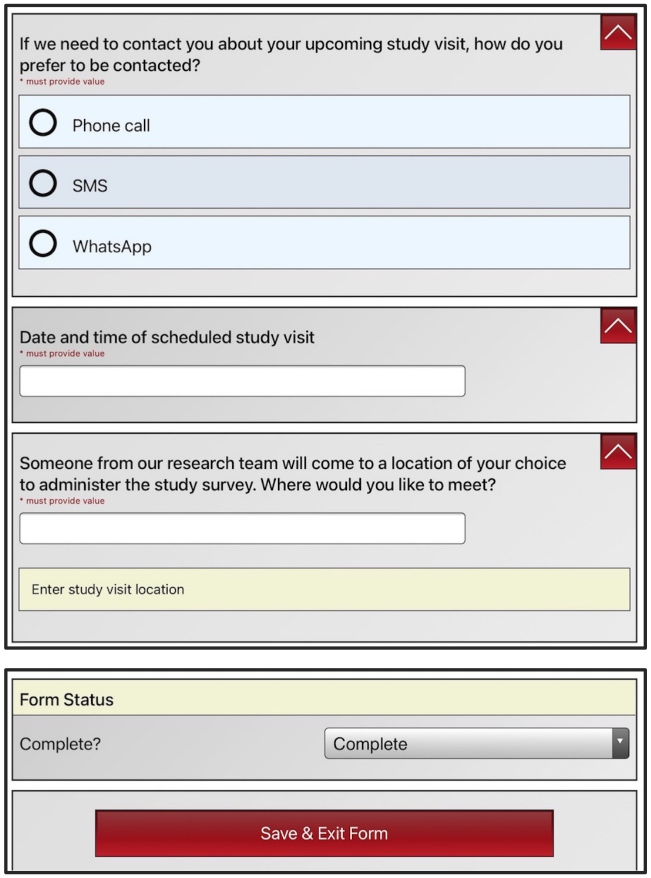

Consent procedures

- Research assistant will meet participant with tablet to administer ICF and questionnaire
- eConsent via REDCap app
- Review ICF with participant and answer questions
- Participant signs ICF on tablet

eConsent

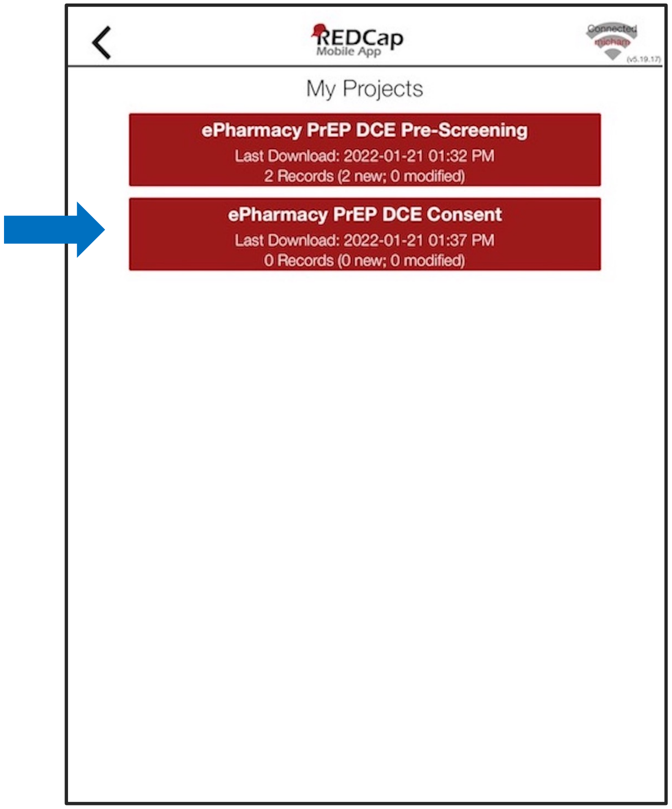
REDCap
Mobile App
My Projects
ePharmacy PrEP DCE Pre-Screening
Last Download: 2022-01-21 01:32 PM
2 Records (2 new; 0 modified)
ePharmacy PrEP DCE Consent
Last Download: 2022-01-21 01:37 PM
0 Records (0 new; 0 modified)

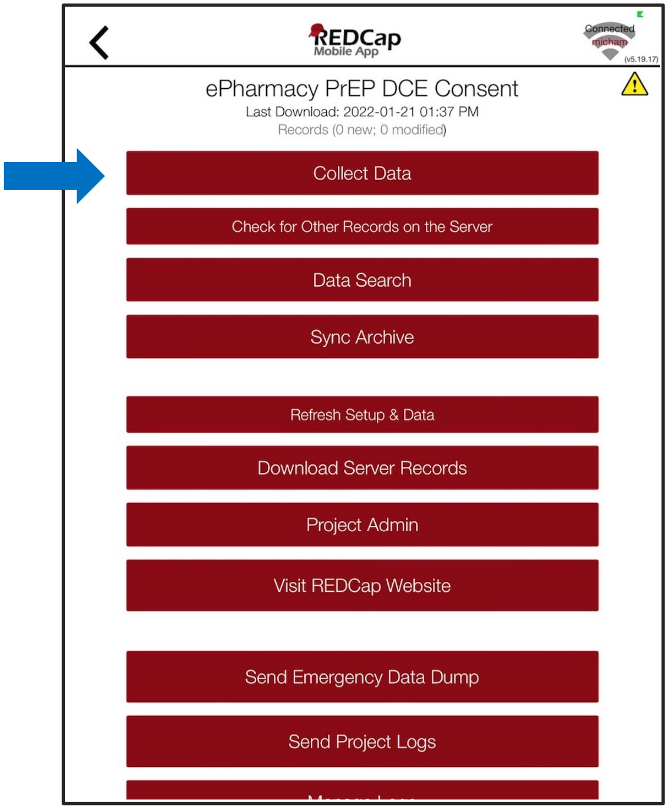
REDCap
Mobile App
ePharmacy PrEP DCE Consent
Last Download: 2022-01-21 01:37 PM
Records (0 new; 0 modified)
Collect Data
Check for Other Records on the Server
Data Search
Sync Archive
Refresh Setup & Data
Download Server Records
Project Admin
Visit REDCap Website
Send Emergency Data Dump
Send Project Logs

eConsent

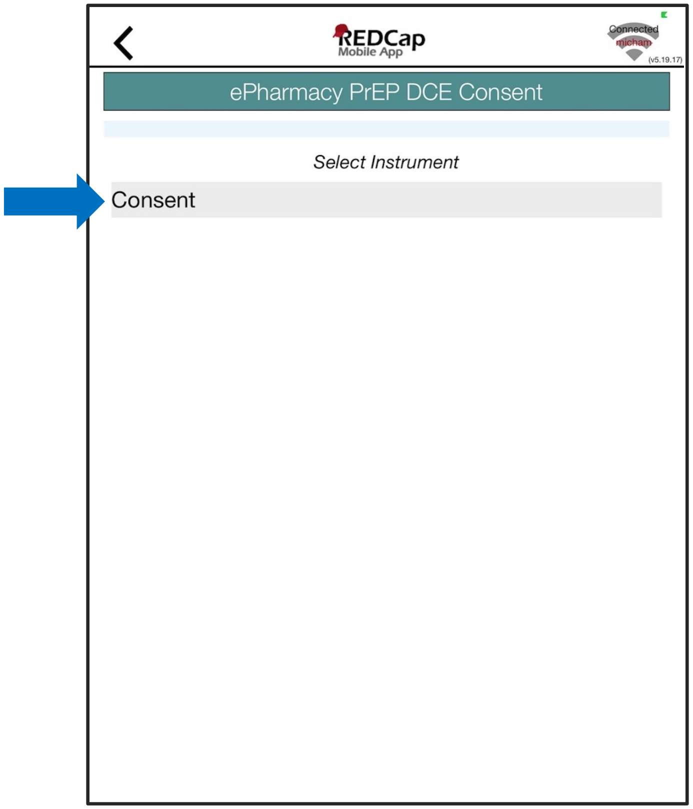
REDCap
Mobile App
Connected
(v5.19.17)
ePharmacy PrEP DCE Consent
Select Instrument
Consent

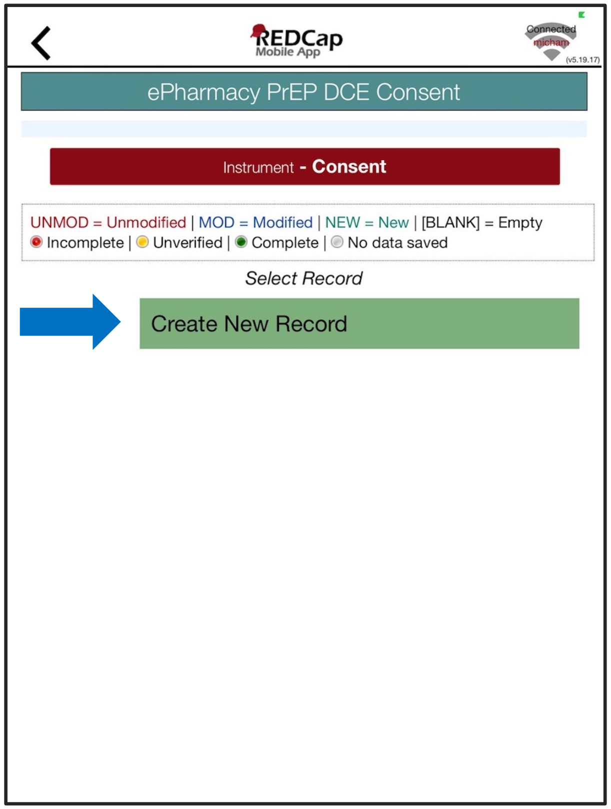
REDCap
Mobile App
Connected
(v5.19.17)
ePharmacy PrEP DCE Consent
Instrument - Consent
UNMOD = Unmodified | MOD = Modified | NEW = New | [BLANK] = Empty
Incomplete | Unverified | Complete | No data saved
Select Record
Create New Record

Sawtooth: types of questions

- Labels to be read
- Multiple-Choice
- Checklist
- Entering a Number or Text or Date
- DCE Scenarios

Sawtooth: labels to be read

- Labels do not require data entry
- Labels will have information for you and/or the participant
- In some cases, labels can be a script that can read aloud to participants
- For example, in **Questionnaire: Introduction;**

Introduction

Thank you for taking time to talk with me. We would like to speak to you about pre-exposure prophylaxis or PrEP. PrEP is a medicine you can take to reduce your risk of getting HIV. We have asked you to participate in this survey because we are interested in learning how best to delivery PrEP using an online pharmacy. This means a pharmacy will deliver PrEP medication to clients using a courier, so clients do not need to travel to a pharmacy. We would like to understand your preferences for PrEP delivery through an online pharmacy. This survey should take about 60 minutes. Feel free to let me know if you need a break at any time. You can also stop the survey if you do not want to continue. Before we begin do you have any questions?

Next

Sawtooth: multiple-choice questions

- You can only select one answer
- Example, **Questionnaire: Part 1: Eligibility Assessment;**

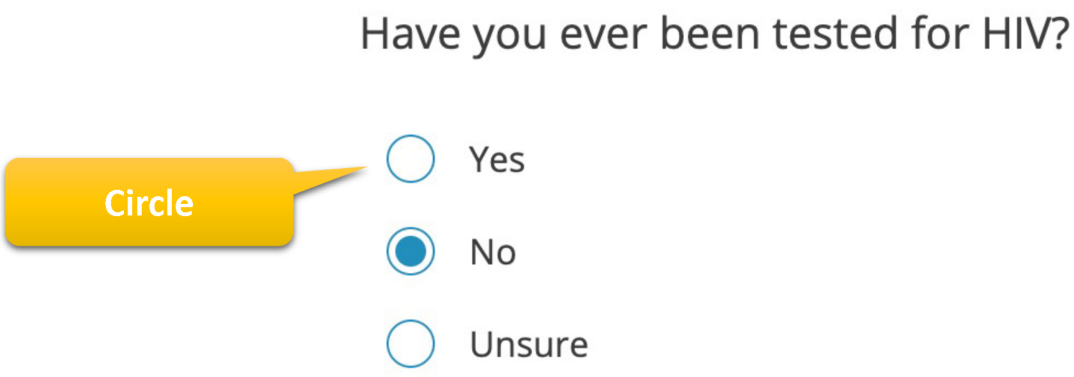

Sawtooth: checklist questions

- Here, you may select as many options as you like
- You should select all the answers that apply
- Example, **Questionnaire: Part 6: E-Pharmacy Engagement and HIV Self-Testing**

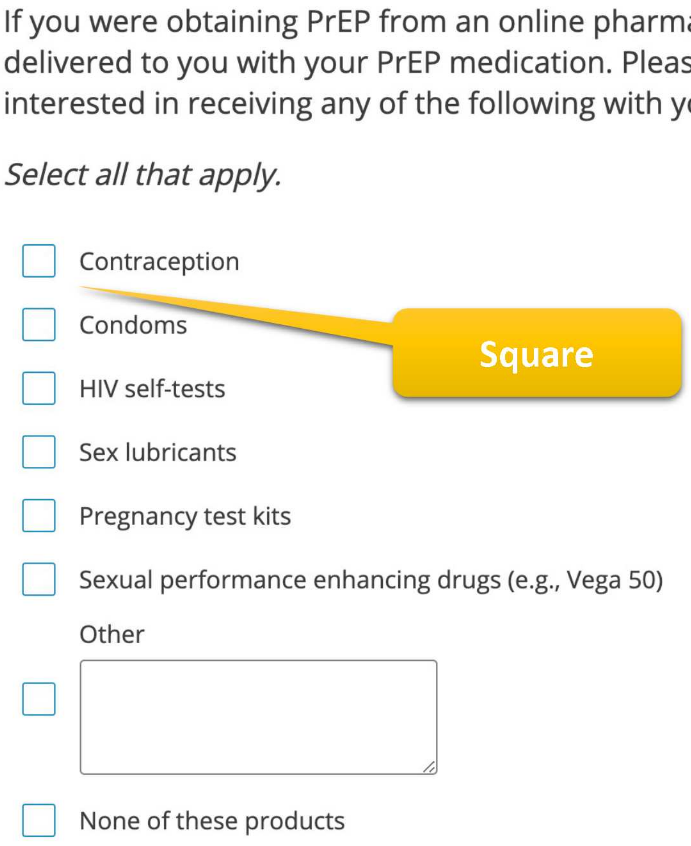
If you were obtaining PrEP from an online pharmacy, you could have other products delivered to you with your PrEP medication. Please indicate if you would be interested in receiving any of the following with your PrEP delivery.

Select all that apply.

- ☐ Contraception
- ☐ Condoms
- ☐ HIV self-tests
- ☐ Sex lubricants
- ☐ Pregnancy test kits
- ☐ Sexual performance enhancing drugs (e.g., Vega 50)
- ☐ Other
- ☐ None of these products

Sawtooth: entering numbers

- Entering number examples

Questionnaire: Part 1: Eligibility Assessment;

What is your age in years? [ ]

Questionnaire: Part 6: E-Pharmacy Engagement and HIV Self-Testing;

For a blood-based HIV self-test, delivered via a courier to a location of your choice (one-time cost):

What is the lowest price you are willing to pay? [ ] KSH

What is the highest price you are willing to pay? [ ] KSH

What price do you consider ideal? [ ] KSH

Sawtooth: entering text

- Entering text example
- Text questions will most often appear to clarify “Other”

If you were obtaining PrEP from an online pharmacy, you could have other products delivered to you with your PrEP medication. Please indicate if you would be interested in receiving any of the following with your PrEP delivery.

Select all that apply.

- ☐ Contraception
- ☐ Condoms
- ☐ HIV self-tests
- ☐ Sex lubricants
- ☐ Pregnancy test kits
- ☐ Sexual performance enhancing drugs (e.g., Vega 50)
- ☐ Other
- ☐ None of these products

Sawtooth: entering date

- Entering date examples
- Date questions will have drop-down menus

Questionnaire: Part 1: Eligibility Assessment

When was your last HIV test?

Month: June Year: 2017

Questionnaire: Part 2: PrEP Knowledge and Interest

When did you first start taking PrEP?

Month: Please select Year: Please select

Sawtooth: required questions

- Almost every question requires an answer
- If you try to move on without providing an answer, a red screen will appear with a reminder that the response is required.

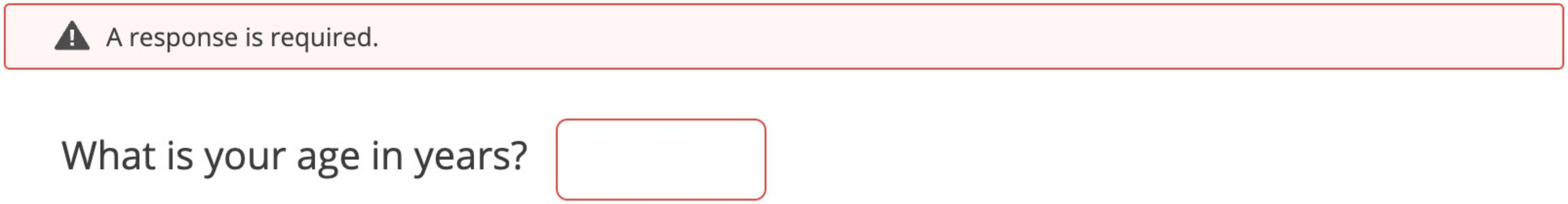

Sawtooth: DCE scenarios

- Explain in the next section

Structure of the Questionnaire

- Introduction
- Part 1: Eligibility Assessment
- Part 2: PrEP Knowledge and Interest
- Part 3: Introduction to Discrete Choice Experiment
- Part 4: Definition of Characteristics
- Part 5: Scenarios
- Part 6: E-Pharmacy Engagement and HIV Self-Testing
- Part 7: Participant Demographics
- Part 8: Sexual Behavior

Structure of the Questionnaire

- **Introduction**
- **Part 1: Eligibility Assessment**
- **Part 2: PrEP Knowledge and Interest**
- Part 3: Introduction to Discrete Choice Experiment
- Part 4: Definition of Characteristics
- Part 5: Scenarios
- Part 6: E-Pharmacy Engagement and HIV Self-Testing
- Part 7: Participant Demographics
- Part 8: Sexual Behavior

Introduction

Introduction

Thank you for taking time to talk with me. We would like to speak to you about pre-exposure prophylaxis or PrEP. PrEP is a medicine you can take to reduce your risk of getting HIV. We have asked you to participate in this survey because we are interested in learning how best to delivery PrEP using an online pharmacy. This means a pharmacy will deliver PrEP medication to clients using a courier, so clients do not need to travel to a pharmacy. We would like to understand your preferences for PrEP delivery through an online pharmacy. This survey should take about 60 minutes. Feel free to let me know if you need a break at any time. You can also stop the survey if you do not want to continue. Before we begin do you have any questions?

Next

Participant ID

- Participant ID should be entered twice
- Same PTID as ICF – links questionnaire responses to ICF

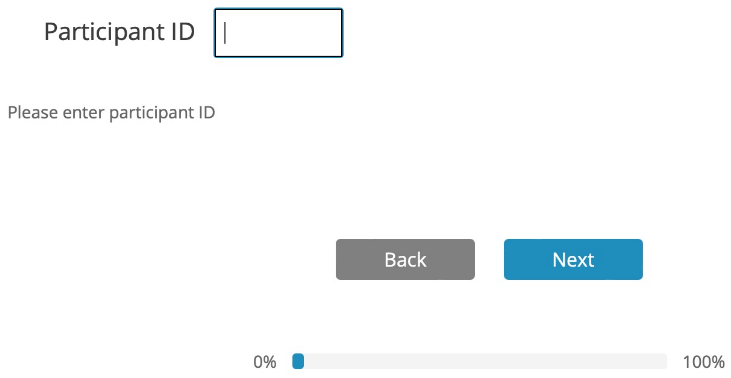

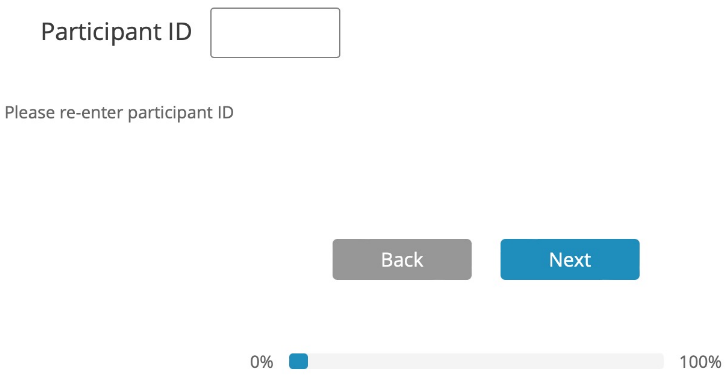

Part 1: Eligibility Assessment

- Eligibility assessment to be completed again at time of questionnaire administration

Part 1: Eligibility Assessment

What is your age in years? [ ]

Back | Next

In the past 6 months, do you think you may have been exposed to HIV?

For example, you might select "yes" if, during the past 6 months, you:

• had a condom break

• shared needles, syringes, or other equipment to inject drugs, or

• were sexually assaulted

○ Yes
○ No
○ Unsure

In the past 6 months, have you had sex with more than one person?

○ Yes
○ No
○ Unsure

Part 2: PrEP Knowledge and Interest

Part 2: PrEP Knowledge and Interest

Have you ever heard of pre-exposure prophylaxis, or PrEP, for HIV prevention before today?

- Yes
- No

Back   Next

Structure of the Questionnaire

- Introduction
- Part 1: Eligibility Assessment
- Part 2: PrEP Knowledge and Interest
- **Part 3: Introduction to Discrete Choice Experiment**
- **Part 4: Definition of Characteristics**
- **Part 5: Scenarios**
- **Part 6: E-Pharmacy Engagement and HIV Self-Testing**
- Part 7: Participant Demographics
- Part 8: Sexual Behavior

Part 3: Introduction to Discrete Choice Experiment

As we mentioned, we're designing a new way to deliver PrEP through an online pharmacy. We would like to understand your preferences to help us design this service. Online PrEP delivery includes different services, including an assessment to determine if you are at risk for HIV, HIV testing to confirm you are HIV-negative, and talking to a medical provider to make sure PrEP is safe for you. To determine your preferences for these services, we will show you a series of **9** scenarios with **2** options for online PrEP service delivery. For each of these scenarios, we will ask you to choose the delivery strategy you most prefer.

Back   Next

Part 4: Definition of Characteristics

Here we describe the characteristics of obtaining PrEP through online pharmacy in the order you would experience them. These are the characteristics we will ask you to consider when making a decision about which option you prefer. Please consider ONLY these characteristics when making your choices.

Clinical Consultation

You would then have a clinical consultation with a medical provider to make sure PrEP is safe for you to use. This can be done over the phone or a video chat.

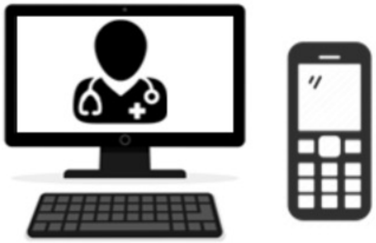

Another option is to see a provider in person at a setting of your choice.

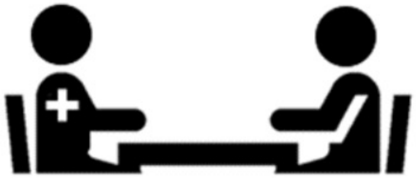

Part 5: Scenarios

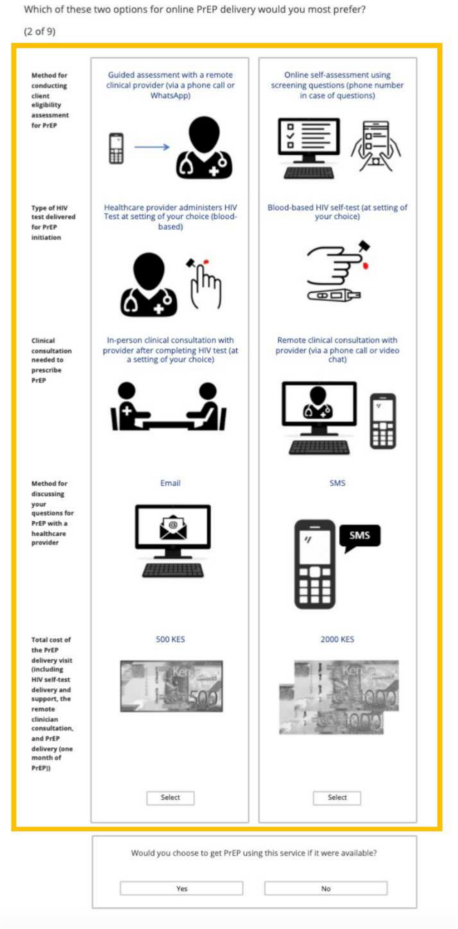

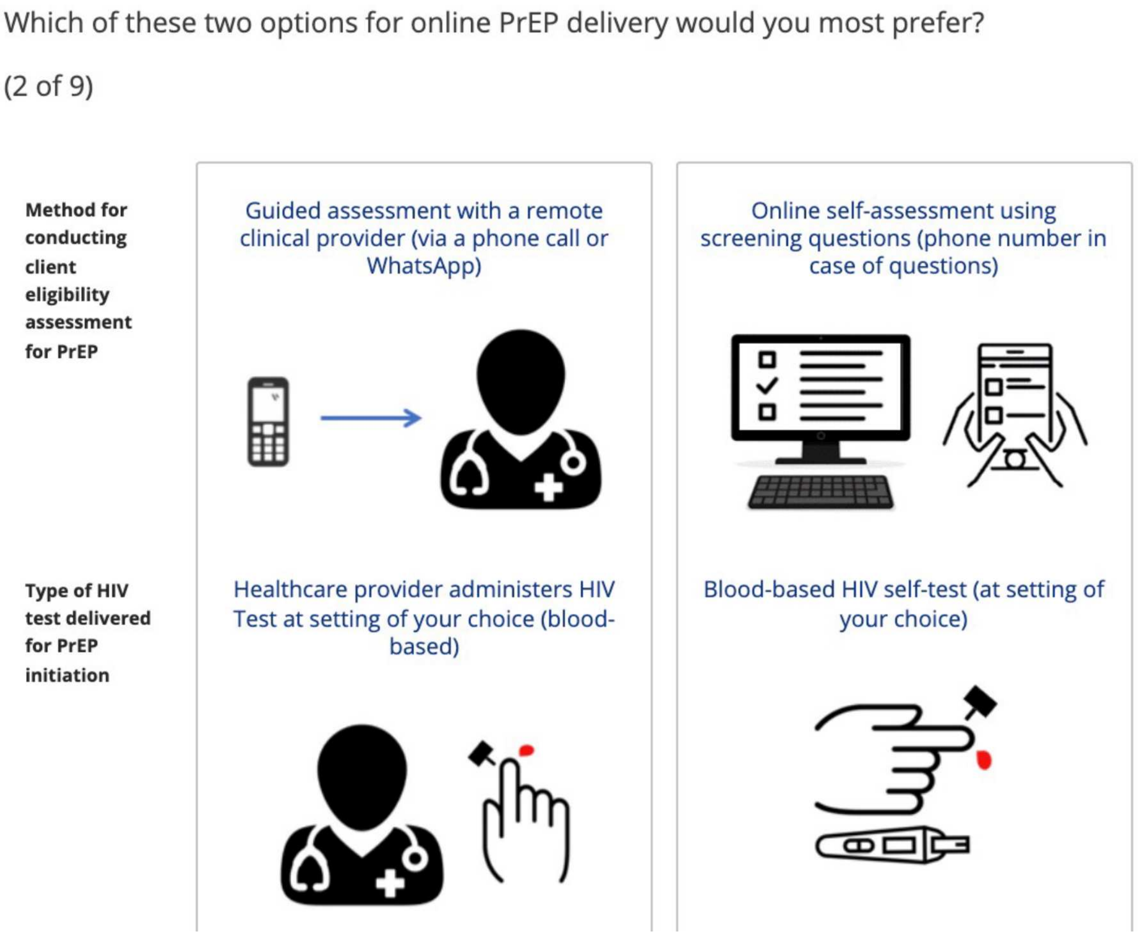

Part 5: Scenarios

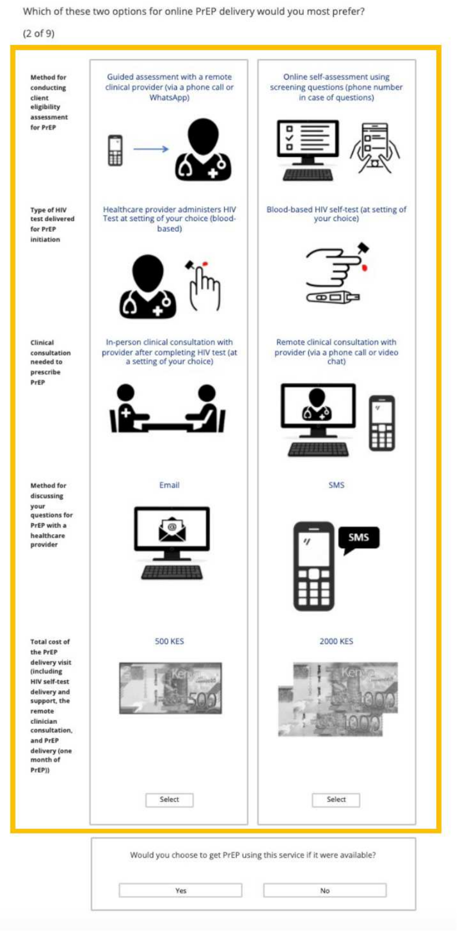

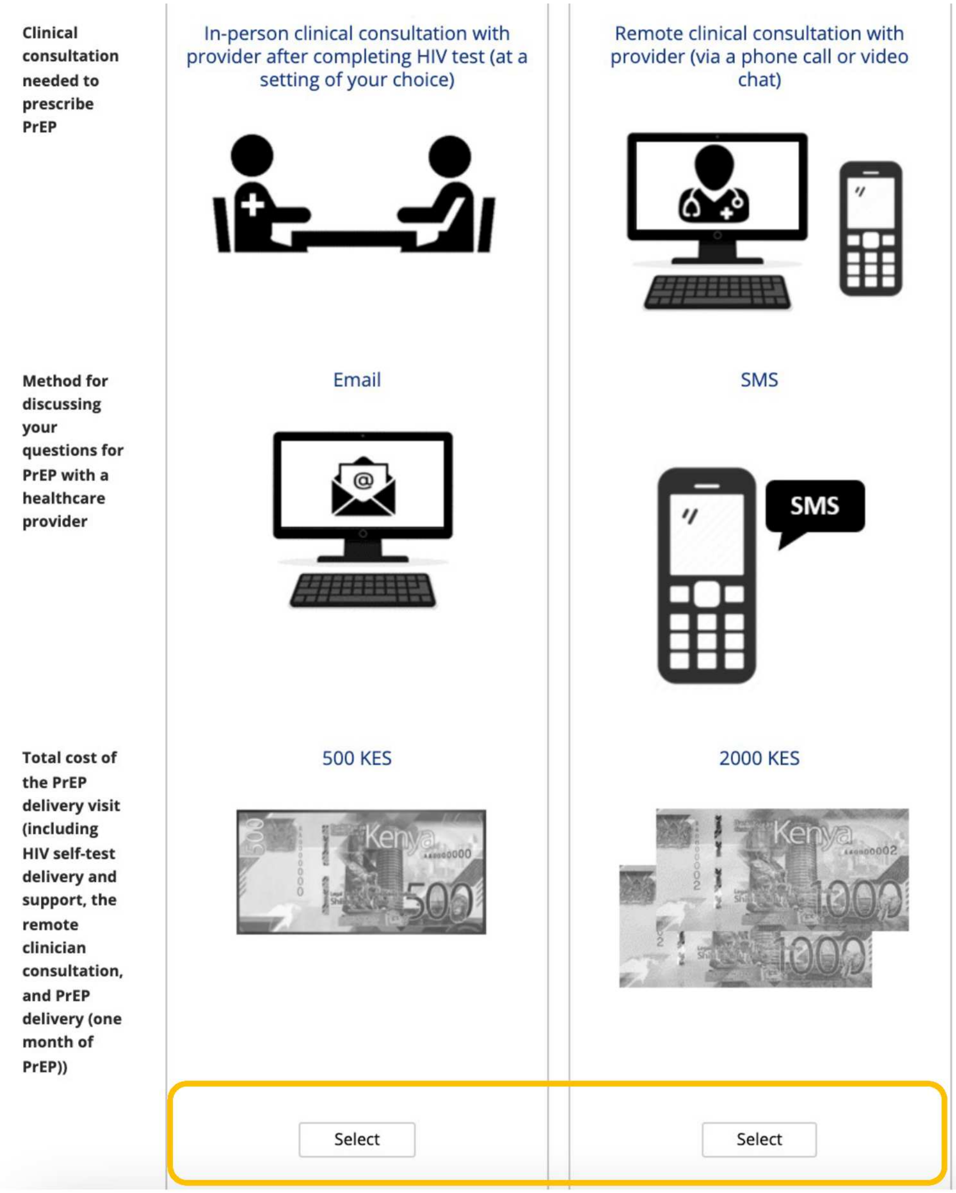

Part 5: Scenarios

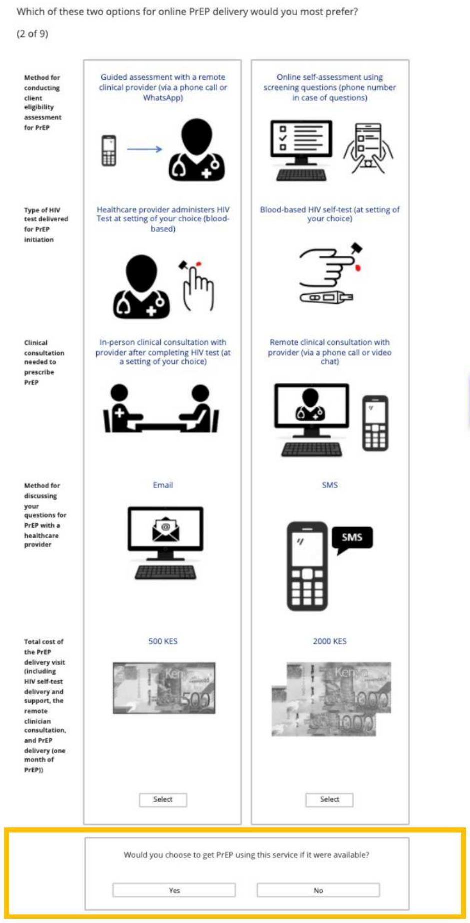

Dual-response question:

Would you choose to get PrEP using this service if it were available?

Yes | No

Back | Next

Part 6: E-Pharmacy Engagement and HIV Self-Testing

- Willingness-to-pay questions

The next set of questions will ask about how much you would like to pay for different parts of online PrEP delivery. For each part, please provide the lowest price, the maximum price and the ideal price you are willing to pay. For the lowest price, please tell us the lowest amount you think the service should cost because otherwise you would be concerned about its quality, and you would not purchase the service. In answering these questions, please consider your usual expenses. Remember that there are no right or wrong answers. We're interested in your preference. You can write zero if you feel that most accurately reflects your preference.

For a blood-based HIV self-test, delivered via a courier to a location of your choice (one-time cost):

What is the lowest price you are willing to pay? [ ] KSH

What is the highest price you are willing to pay? [ ] KSH

What price do you consider ideal? [ ] KSH

Structure of the Questionnaire

- Introduction
- Part 1: Eligibility Assessment
- Part 2: PrEP Knowledge and Interest
- Part 3: Introduction to Discrete Choice Experiment
- Part 4: Definition of Characteristics
- Part 5: Scenarios
- Part 6: E-Pharmacy Engagement and HIV Self-Testing
- **Part 7: Participant Demographics**
- **Part 8: Sexual Behavior**

Part 7: Participant Demographics

Part 7: Participant Demographics

In what region do you live?

- North Eastern
- Nyanza
- Western
- Rift Valley
- Nairobi
- Central
- Coast
- Eastern

What is the highest level of education you have completed?

- Primary
- Secondary
- O Levels
- A Levels
- Technical or vocational school
- University or higher

Part 8: Sexual Behavior

- Prefer not to answer option

Part 8: Sexual Behavior

In the last 3 months, have you had a primary sex partner?

(A primary sex partner is a person you have sex with on a regular basis, or someone you consider to be your main partner.)

- ○ Yes
- ○ No
- ● Prefer not to answer

Submitting data

The questionnaire ends here. Thank you for your responses.

0% 100%

Questions?

ePharmacy PrEP: Discrete Choice Experiment Training

Day 1 Session 3
24th-25th January 2022

Cognitive Interviews, Pilot, and DCE Administration

Process

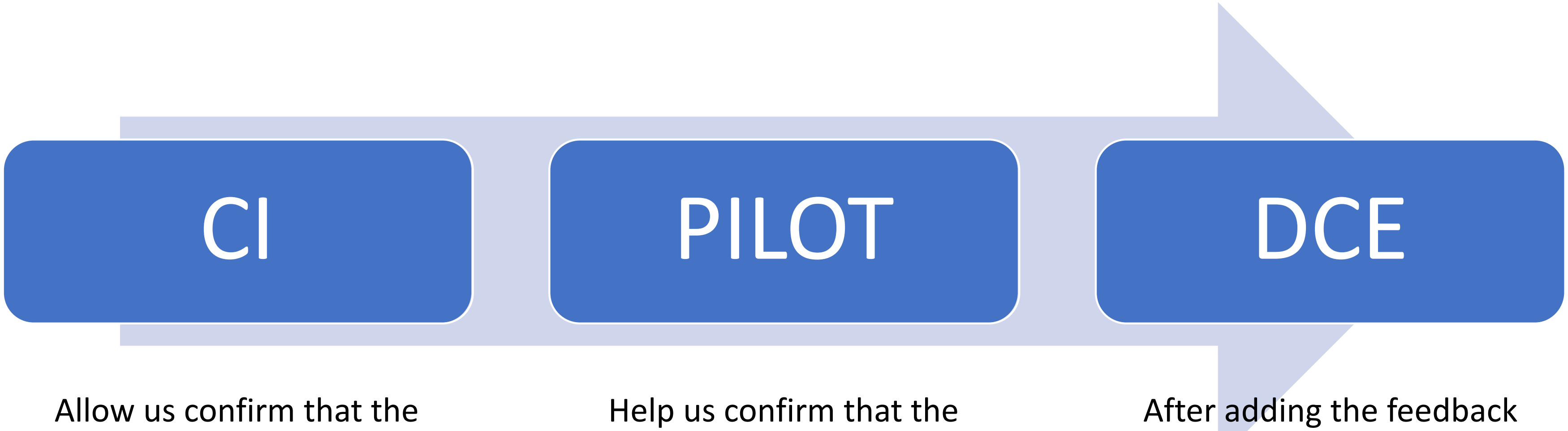

Allow us confirm that the information used to describe the attributes is accurate and some of the questions in the survey are easy to understand and answer

Help us confirm that the instrument (web-based survey) works well, 30 minutes is enough to answer all, and the participants are not (too) tired at the end

After adding the feedback from the CI and Pilot, the DCE will improve and allow us to collect the best data possible. Good-quality data will inform the creation of a good online-pharmacy service for PrEP delivery

Key characteristics

Cognitive Interviews

- Aim to obtain feedback in specific sections of the instrument
- Length: around 1 hour
- Uses a script
- RA very involved

Pilot

- Aim to verify usability and fatigue in the entire instrument
- Length: around 45 min
- No script
- RA involved but less

DCE

- Aims to obtain data of the participants' preferences as unbiased as possible
- Length: 30-45 min
- No script
- RA only involved if participant has questions

Structure – Cognitive Interviews

1. Approach and recruitment
    - In-person
    - Objective: to collect participants' feedback about the DCE attributes and specific sections of the instrument (see script)
    - Length: around 60 minutes
2. Presentation of the instrument
3. Active collection of feedback

Let's see the script

Structure – Pilot

1. Approach and recruitment
   - In-person
   - Objective: to verify if the instrument is friendly and easy to use, if the participants get stuck in any question, if any questions are not clear, how long does it take to complete the instrument, and how tired or bored is the participant at the end
   - Length: around 45 minutes
2. Presentation of the instrument
3. Passive collection of feedback:

   Observe closely while the participant goes through the survey, if at any point there's confusion ask what's happening and offer help. Take notes every time there's confusion or the participant needed clarification, how long it took to finish the survey, and if they were tired and to what extend.

Let's see the instrument

Structure – DCE

1. Approach and recruitment
    - In-person
    - Objective: to collect high-quality data about the participants’ preferences.

    We assist but we don’t advice
    - Length: around 30-45 minutes
2. Presentation of the instrument
3. Passive collection of feedback:

    After recruitment, present the survey to the participant and let them fill their answers.

For peer review only - http://bmjopen.bmj.com/site/about/guidelines.xhtml

Structure – DCE cont.

Clarifying vs. Advice questions

- Clarifying: Related to the meaning of a word or the sense of the question in case of ambiguity or vague meaning
  - Examples:
    - What does 'pay-by-piece' mean?
    - Is contraception the same as emergency pills?
    - Where do I click if I don't know the answer to this question (or don't want to tell)?
- Advice: asks for your opinion or advice
  - Examples:
    - How can I know if clinical consultation is more important than Cost?
    - Do you think oral HIV testing is easier than when they draw blood?

Summary

- Each step help us have the best possible instrument for the DCE
- Cognitive Interviews require the most involvement to obtain feedback, followed by Pilot, and in the DCE we collect no feedback but just administer the survey
- In the DCE we want to collect only the participants' preferences, so we avoid providing any opinion or advice, even if they ask
- Questions?

Supplement 3: Study Questionnaire

DISCRETE CHOICE EXPERIMENT QUESTIONNAIRE

Introduction:

Thank you for taking time to talk with me. We would like to speak to you about pre-exposure prophylaxis or PrEP. PrEP is a medicine you can take to reduce your risk of getting HIV. We have asked you to participate in this survey because we are interested in learning how best to delivery PrEP using an online pharmacy. This means a pharmacy will deliver PrEP medication to clients using a courier, so clients do not need to travel to a pharmacy. We would like to understand your preferences for PrEP delivery through an online pharmacy. This survey should take about 45 minutes. Feel free to let me know if you need a break at any time. You can also stop the survey if you do not want to continue. Before we begin do you have any questions?

RA Initials
Research Assistant Initials
(The RA conducting this questionnaire should enter their initials.)

Participant ID
Please enter participant ID from enrollment log.
(This should match the PTID entered on the REDCap consent form.)

Please re-enter participant ID from enrollment log.
(This should match the PTID entered on the REDCap consent form.)

Part 1: Eligibility Assessment

1	What is your age in years?	|__|__| years <18, Stop screening. This individual is NOT eligible for study participation. Thank them for their time
2	Have you ever been tested for HIV?	☐ *Yes* ☐ *No* ☐ *Unsure*
3	When was your last HIV test?	_______ Month ______Year
4	What was the result of your last HIV test?	☐ *Negative*<br>☐ *Positive*<br>☐ *Unknown*<br><br>If positive, this individual is NOT eligible for study participation. Stop screening and thank them for their time.

Part 2: PrEP Knowledge and Interest

5	Have you ever heard of pre-exposure prophylaxis, or PrEP, for HIV prevention before today?	Yes<br>No
6	Have you ever taken PrEP to prevent HIV? *[If 5=Yes]*	Yes<br>No<br>I don't know<br>Prefer not to answer
6.1	Are you currently taking PrEP to prevent HIV? *[If Q6 = Yes]*	Yes<br>No

For peer review only

6.2	When did you first start taking PrEP? *[If Q6 = Yes]*	Month: Year:<br>MM/YYYY
7	Would you be interested in taking PrEP to prevent HIV? *[If Q6 != Yes]*	Yes<br>No<br>I don't know

Persons who answer NO to both of Q6, Q7 are not eligible for participation

If participant is not eligible: Based on your responses, you are not eligible to continue with the questionnaire. Thank you for taking the time to respond.

Part 3: Introduction to Discrete Choice Experiment

As we mentioned, we're designing a new way to deliver PrEP through an online pharmacy. We would like to understand your preferences to help us design this service. Online PrEP delivery includes different services, including an assessment to determine if you are at HIV risk, HIV testing to confirm you are HIV-negative, and talking to a medical provider to make sure PrEP is safe for you. To determine your preferences for these services, we will show you a series of **8** scenarios with 2 options for online PrEP service delivery. For each of these scenarios, we will ask you to choose the delivery strategy you most prefer.

Part 4: Definition of Characteristics

Part 5: Scenarios

We will now ask you about how you would like to get PrEP from an online pharmacy. Please remember there are no wrong answers, we want to know your preferences. When answering each scenario, imagine that you are planning to get PrEP using an online pharmacy. We will also ask you if you would choose to get PrEP using the option you selected. If you would not choose to get PrEP if only these two options were available, you can answer no to this question.

Scenarios

Which of these two options for online PrEP delivery would you most prefer?

If the PrEP service you just chose was available, do you think you would actually use it?

Part 6: E-Pharmacy Engagement and HIV Self-Testing

8	Have you ever purchased medication or other products from an online pharmacy (e-pharmacy)?	Yes<br>No
9	Have you ever purchased any of the following medications or products from an online pharmacy (e-pharmacy)? *[If Q8 = Yes]*<br>*Select all that apply*	Contraception<br>Condoms<br>HIV self-tests<br>Sex lubricants<br>Pregnancy test kits<br>Sexual performance enhancing drugs (e.g., Vega 50)<br>Other<br>None of these services
10	How would you most prefer to obtain your PrEP medication? *[If Q7 = Yes]*	Clinic<br>Community pharmacy or chemist<br>Online pharmacy (e-pharmacy)<br>Community health worker (e.g., home visit)<br>Other

For peer review only

11	If you were obtaining PrEP from an online pharmacy, you could have other products delivered to you with your PrEP medication. Please indicate if you would be interested in receiving any of the following with your PrEP delivery. *Select all that apply*	Pregnancy test kits<br>Contraception<br>Condoms<br>Sex lubricants<br>Sexual performance enhancing drugs (e.g., Vega 50)<br>HIV self-test<br>None of these products<br>Other ______________
12	If you were obtaining PrEP from an online pharmacy, how frequently would you prefer to refill your PrEP medication?	Every 1 month<br>Every 3 months<br>Every 6 months<br>Other ________
The next set of questions will ask about how much you would like to pay for different parts of online PrEP delivery. For each part, please provide the maximum price you are willing to pay. In answering these questions, please consider your usual expenses. Remember that there are no right or wrong answers. We're interested in your preference. You can write zero if you feel that most accurately reflects your preference.		
13	What is the maximum price you are willing to pay for a blood-based HIV self-test?	__________ KSH
14	What is the maximum price you are willing to pay for an oral HIV self-test?	__________ KSH
15	What is the maximum price you are willing to pay for remote/online clinical consultation to obtain a prescription for PrEP based on your HIV self-test results (one-time cost)?	__________ KSH
16	What is the maximum price you are willing to pay for a one-month supply of PrEP medicines?	__________ KSH
17	What is the maximum price you are willing to pay for delivery of PrEP medicines to a setting of your choice (one-time courier fee)?	__________ KSH
18	What is the maximum price you are willing to pay in total for the package of services associated online PrEP delivery (this includes courier delivery of an HIV self-test, a remote clinical consultation, and courier-delivered PrEP)?	__________ KSH
19	How would you most prefer to pay for the package of online PrEP services (e.g., HIV testing, remote consultation, PrEP medication delivery)?	Pay by piece (e.g., pay separately for HIV testing, the remote clinic visit, and PrEP drug delivery)<br>Pay per PrEP visit (including HIV testing, the remote visit, and PrEP medication delivery)<br>Pay a monthly subscription
The next set of questions ask about your preferences for online PrEP delivery		
20	What support, if any, would you be interested in from the online pharmacy provider to help you consistently take your PrEP medication? (select all that apply)	SMS reminders<br>Emails<br>Phone calls<br>Video calls<br>No support needed
21	At what frequency, would you like support for taking your PrEP medication?	Daily<br>Weekly<br>Bi-weekly (every other week)<br>Monthly
22	Have you heard about HIV self-testing as a method for testing for HIV before today?	Yes<br>No<br>Prefer not to answer

For peer review only

23	Have you ever used a self-test to test for HIV?	Yes<br>No<br>Prefer not to answer
24	How much would you like or dislike obtaining PrEP though an online platform?	Strongly dislike<br>Somewhat dislike<br>No opinion<br>Somewhat like<br>Strongly like
25	How much effort do you think it would take you to obtain PrEP online?	No effort<br>A little effort<br>A moderate effort<br>A huge amount of effort<br>No opinion
26	How confident do you feel about your ability to navigate an online website to obtain PrEP?	Very unconfident<br>Somewhat unconfident<br>No opinion<br>Somewhat confident<br>Very confident
27	How much do you agree or disagree with the following statement: Online PrEP delivery would help reduce HIV in my community.	Strongly disagree<br>Somewhat disagree<br>No opinion<br>Somewhat agree<br>Strongly agree
28	Which media sources do you most frequently use to get general information; such as daily news, important events, etc.	Newspapers<br>Radio<br>Television<br>WhatsApp groups<br>Social networks (e.g., Facebook, Instagram, Tik Tok)<br>Internet browsing (e.g., google, news' websites)
29	Which media sources do you most frequently use to get healthcare information; such as healthcare campaigns, interventions, new medicines, etc.	Newspapers<br>Radio<br>Television<br>WhatsApp groups<br>Social networks (e.g., Facebook, Instagram, Tik Tok)<br>Internet browsing (e.g., google, news' websites)

For peer review only

Part 7: Participant Demographics

30	In what region do you live?	North Eastern<br>Nyanza<br>Western<br>Rift Valley<br>Nairobi<br>Central<br>Coast<br>Eastern
31	What is your gender?	Male<br>Female<br>Other
32	Are you currently enrolled in school?	Yes<br>No
33	What is the highest level of education you have completed?	Primary<br>Secondary<br>O Levels

		A Levels<br>Technical or vocational school<br>University or higher<br>Prefer not to answer
34	Do you have regular employment?	Yes, I work full time<br>Yes, I work part time<br>Yes, I work multiple jobs<br>Yes, seasonal employment<br>No, I do not work<br>Prefer not to answer
35	How much money do you usually earn in a month?	|__|__|__|__|__|__| KES<br>Prefer not to answer
36	Have you ever been married?	Yes<br>No
37	What is your current relationship status?	Married<br>Steady boyfriend or girlfriend<br>Single<br>Widowed<br>Divorced or separated<br>Prefer not to answer
38	Have you ever been pregnant? *[If Q31 = female]*	Yes<br>No
39	Are you using any family planning method to delay or avoid pregnancy?<br>*[If Q31 = female]*	Yes<br>No<br>Prefer not to answer
40	What method(s) are you using? *[If Q39 = Yes]*	Oral pills<br>IUD<br>Implants<br>Injections<br>Condoms<br>Withdrawal<br>Rhythm or periodic abstinence<br>Emergency contraception<br>Tubal ligation/hysterectomy<br>Vasectomy<br>Traditional methods<br>Don't know<br>Prefer not to answer<br>Other ________________
41	Where do you prefer to access these family planning methods? *[If Q39 = Yes]*	Community pharmacy/chemist<br>Online pharmacy<br>Health facility<br>Friend or family member<br>Sexual partner(s)<br>Other ________________

Part 8: Sexual Behavior

42	<u>In the last 3 months</u>, have you had a primary sex partner? (A primary sex partner is a person you have sex with on a regular basis, or someone you consider to be your main partner.)	Yes<br>No<br>Prefer not to answer
43	How long have you been with your primary partner? *[If Q42 = Yes]*	|__|__| years |__|__| months

For peer review only

44	<u>In the past 3 months</u>, have you had sex?	Yes<br>No
45	In the past 3 months<u>, what types of sexual partners have you had? *[If Q44 = Yes]*</u>	One primary partner only (no other partners)<br>One primary partner and casual partner(s)<br>Casual partner(s) only<br>Other ____________<br>Prefer not to answer
46	<u>In the past 3 months</u>, how many different people have you had sex with? *[If Q44 = Yes]*	|__|__|__| people<br>888 = Don't know<br>999 = Prefer not to answer
47	<u>In the past 3 months</u>, how many of these people were new sexual partners (i.e., you had never had sex with them before)? *[If Q44 = Yes]*	|__|__|__| people<br>888 = Don't know<br>999 = Prefer not to answer
48	<u>In the past month</u>, how many times did you have sex?	|__|__|__| times
49	When you had sex <u>in the past month</u>, how many times did you use a condom? *[If Q47 > 0]*	|__|__|__| times
The next set of questions ask about your sexual behavior in the past 6 months		
50	In the past 6 months, have you had sex without a condom?	☐ *Yes* ☐ *No* ☐ *Unsure*
51	<u>In the past 6 months</u>, do you think you may have been exposed to HIV?<br>*For example, you might select "yes" if, during the past 6 months, you:*<br>• *had a condom break*<br>• *shared needles, syringes, or other equipment to inject drugs, or*<br>• *were sexually assaulted*	☐ *Yes* ☐ *No* ☐ *Unsure*
52	<u>In the past 6 months</u>, have you had sex with more than one person?	☐ *Yes* ☐ *No* ☐ *Unsure*
53	Do you have any sex partners who are HIV-positive <u>and:</u><br>• are not currently taking antiretroviral therapy (ART),<br>• have been taking antiretroviral therapy (ART) for less than 6 months,<br>• have been missing doses of their antiretroviral therapy (ART),<br>• have a detectable HIV viral load, <u>or</u><br>• the two of you are trying to get pregnant?<br>*Select "yes" if ANY of the above statements are true about any of your sex partners who are HIV-positive.*	☐ *Yes* ☐ *No* ☐ *Unsure*

For peer review only

54	Do you have any sex partner(s) who you think are at high risk for HIV <u>and</u> whose HIV status you do not know?<br>*Some example behaviors that may make your partner at high risk for HIV include:*<br>• *if he or she has other partners whose HIV status is unknown*<br>• *if he or she exchanges sex for money/gifts <u>or</u> money/gifts for sex*<br>• *if he or she engages in intravenous drug use*	☐ *Yes* ☐ *No* ☐ *Unsure*
55	In the <u>past 6 months</u>, have you used post-exposure prophylaxis (PEP) two times or more?<br>*Post-exposure prophylaxis is a medication that is taken very soon after a possible exposure to HIV to prevent becoming HIV-positive.*	☐ *Yes* ☐ *No* ☐ *Unsure*
56	In the <u>past 6 months</u>, have you been diagnosed with or treated for a sexually transmitted infection (STI)?<br>*Examples of sexually transmitted infections include chlamydia and gonorrhea.*	☐ *Yes* ☐ *No* ☐ *Unsure*
57	In the <u>past 6 months</u>, have you had sex while under the influence of drugs or alcohol?	☐ *Yes* ☐ *No* ☐ *Unsure*
58	In the <u>past 6 months</u>, have you been forced to have sex against your will or physically assaulted, including assault by your sex partner?	☐ *Yes* ☐ *No* ☐ *Unsure*
59	In the <u>past 6 months</u>, have you had sex with someone in exchange for money or a gift?	☐ *Yes* ☐ *No* ☐ *Unsure*
60	In the <u>past 6 months</u>, have you given someone money or a gift in exchange for sex?	☐ *Yes* ☐ *No* ☐ *Unsure*
61	In the <u>past 6 months</u>, have you shared needles with anyone while engaging in intravenous drug use?	☐ *Yes* ☐ *No* ☐ *Unsure*

The questionnaire ends here. Thank you for your responses.

For peer review only

Supplement 4: DCE Images

Online PrEP: DCE Art

3/9/2022

PrEP eligibility assessment

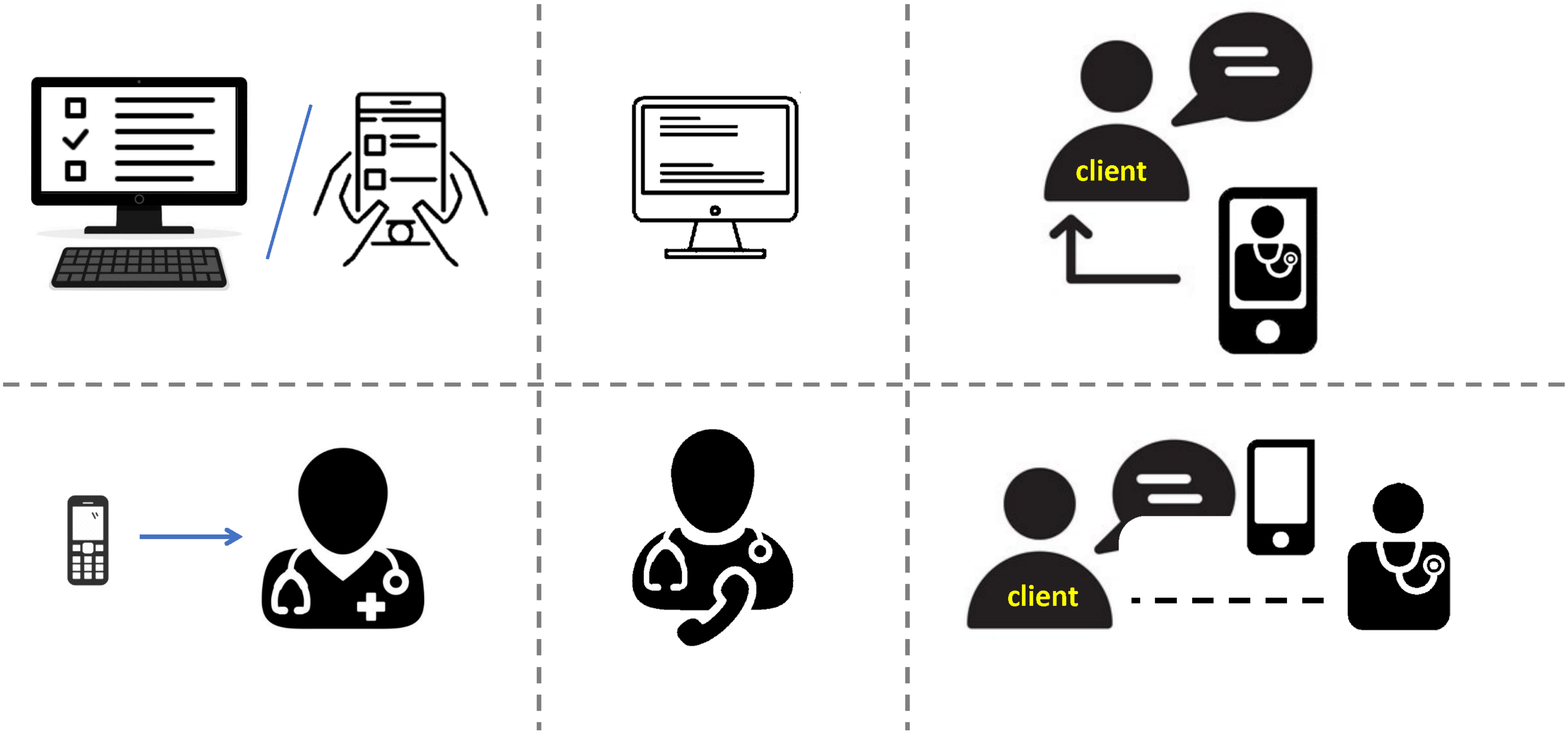

HIV test type

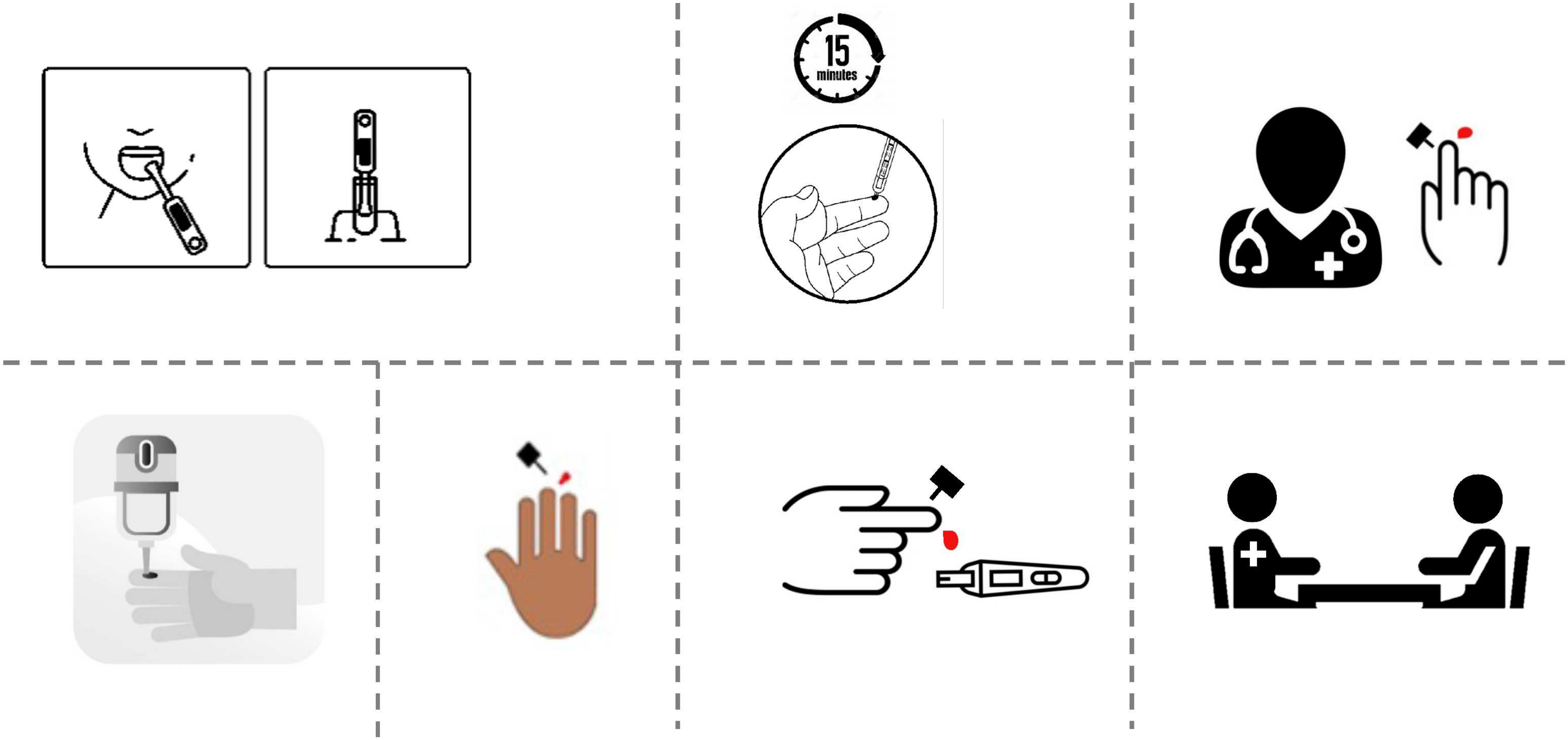

Clinical consultation for prescribing PrEP

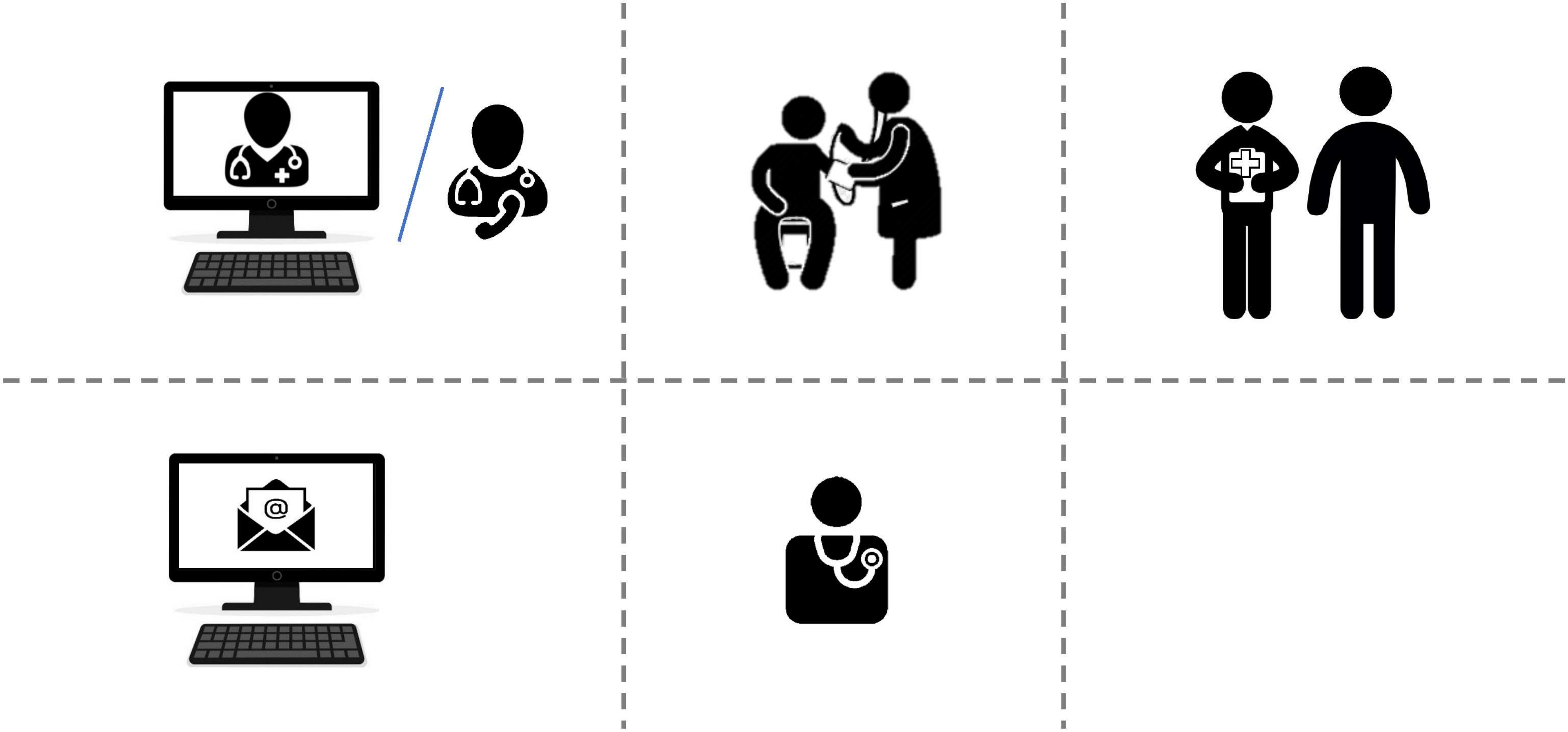

User support options for PrEP

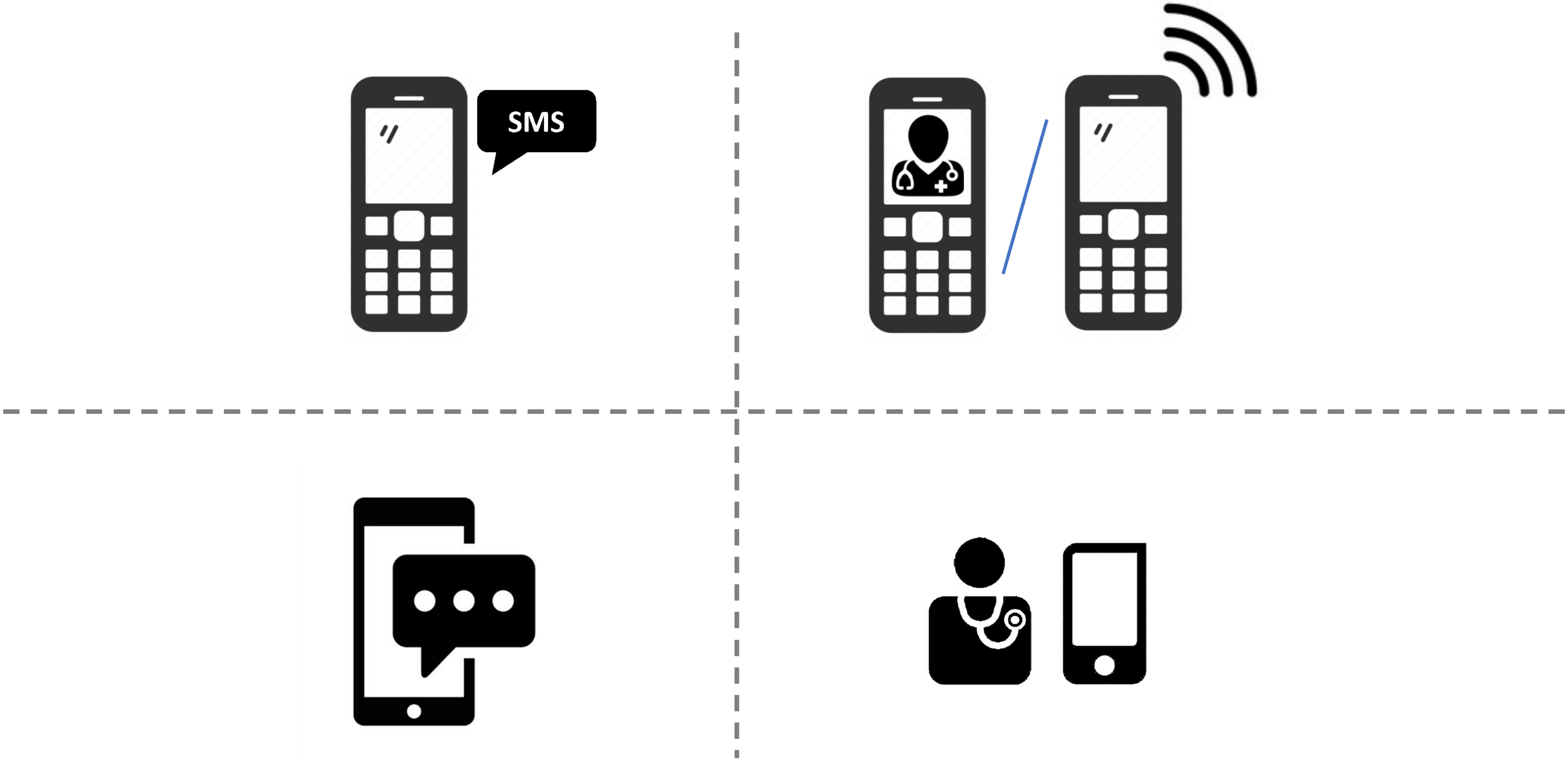

Cost of PrEP delivery

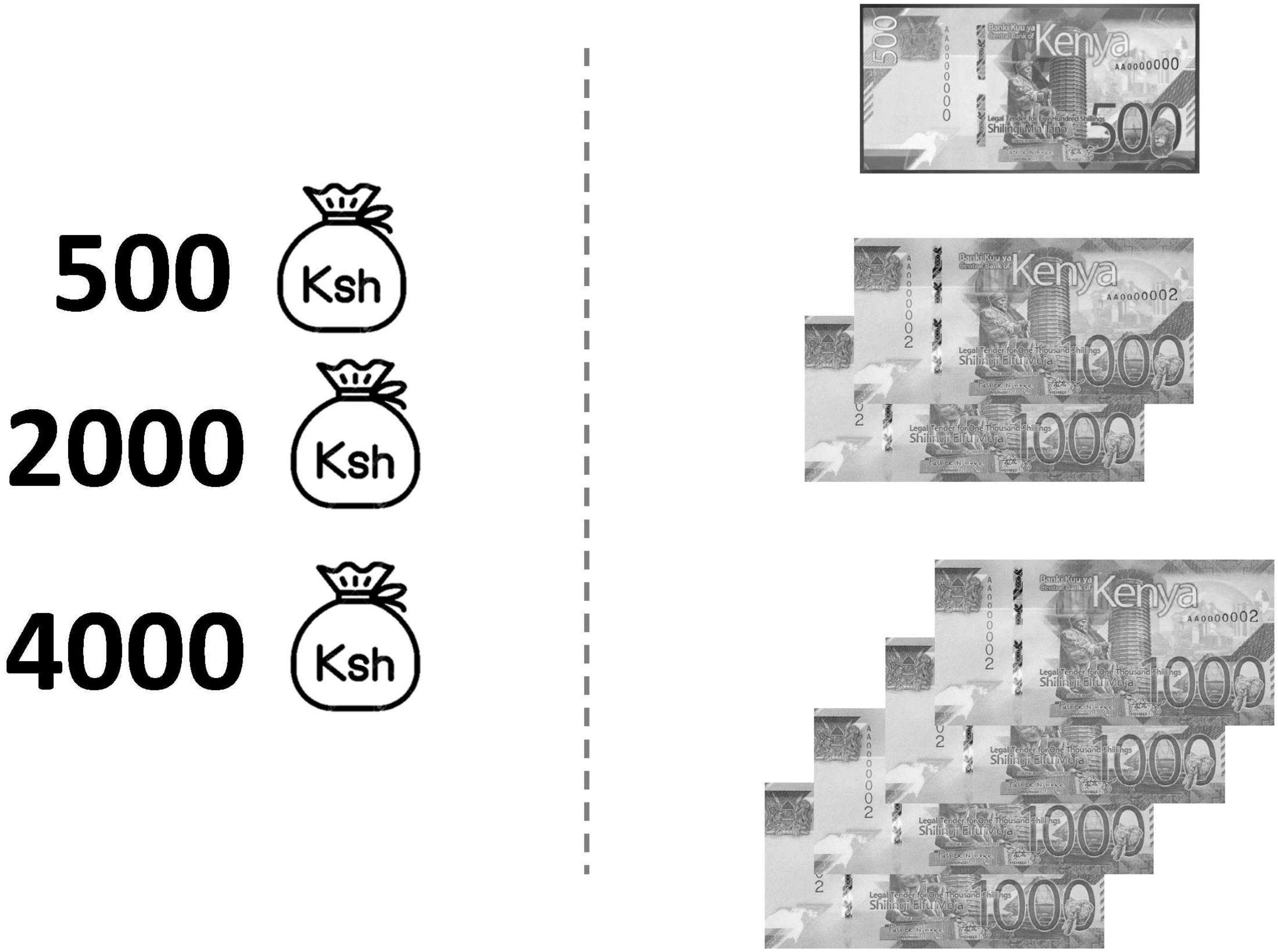

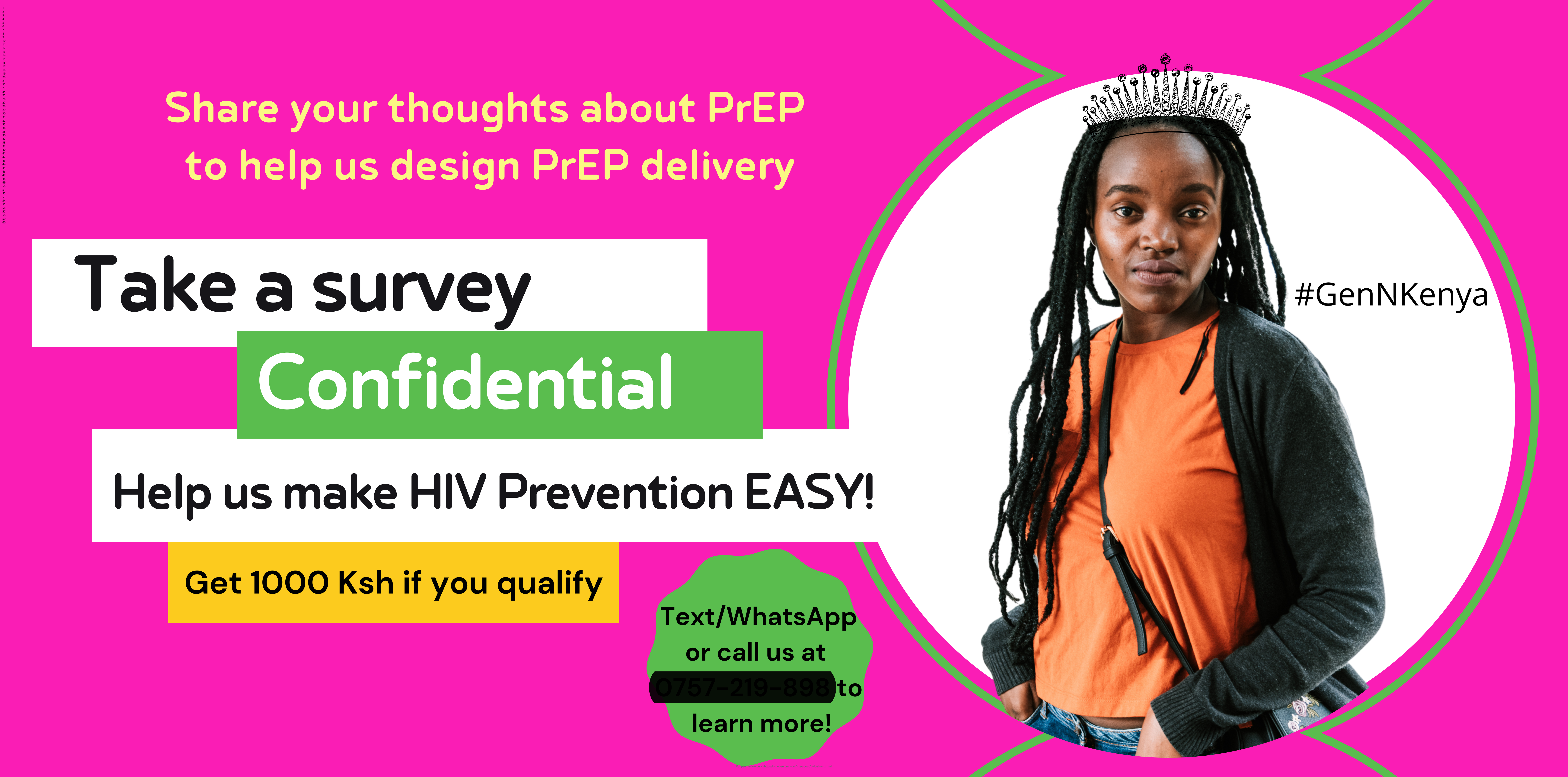
Share your thoughts about PrEP
to help us design PrEP delivery
Take a survey
Confidential
Help us make HIV Prevention EASY!
Get 1000 Ksh if you qualify
Text/WhatsApp
or call us at
[redacted] to
learn more!
#GenNKenya

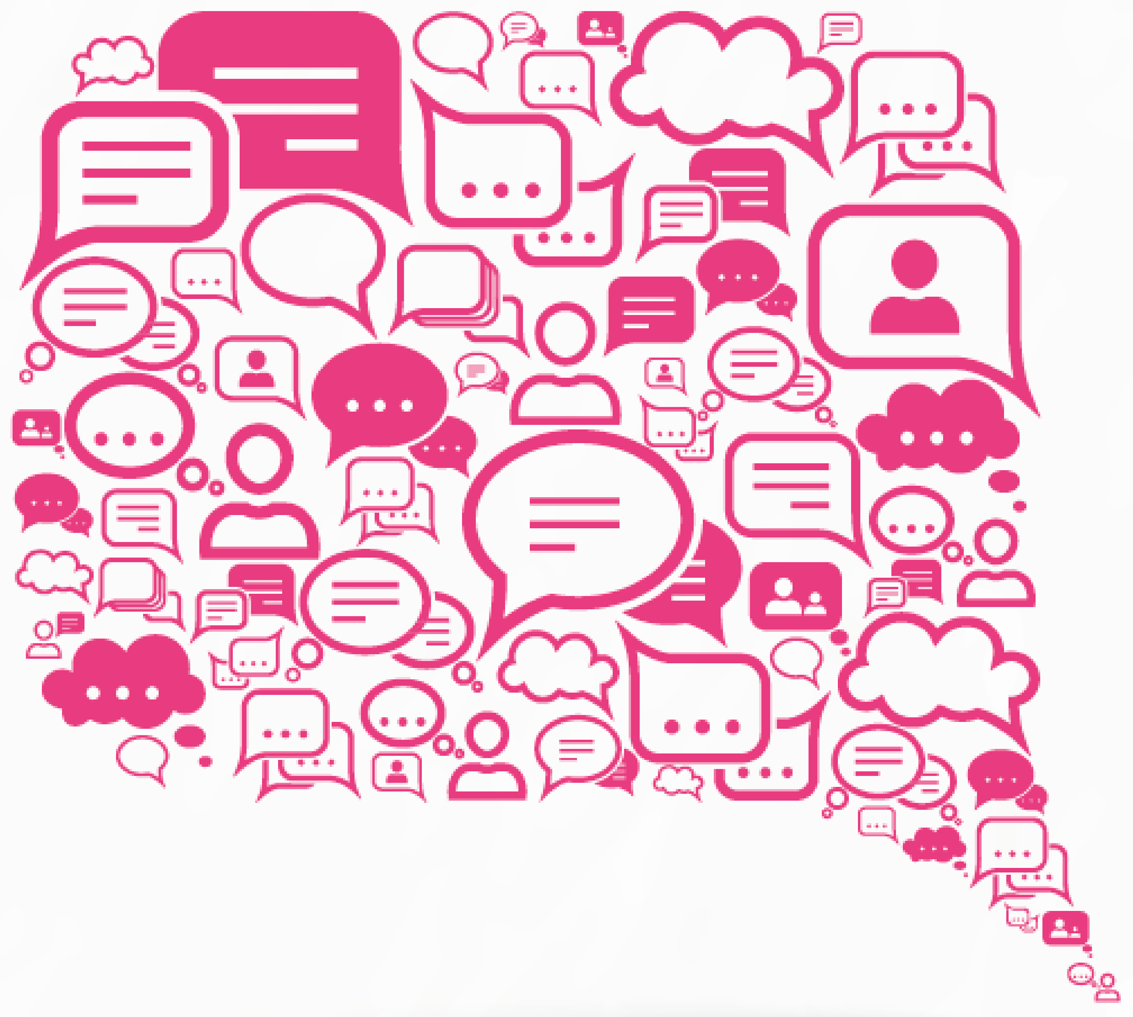

We are doing a survey about **PrEP service delivery.**
PrEP is a pill that you can take daily to reduce your risk of getting HIV.

We would like to understand what you think about getting PrEP delivered to you from an online pharmacy, like **MYDAWA.**

Hearing about your **preferences** will help us design new models of **PrEP delivery** for your community.

If you are interested, we will meet you in a **location of your choice** to complete the survey.
The survey is confidential and takes about **45 minutes.** You will **receive 1,000 Ksh** for your time.

If you are interested in taking part in the survey or have any questions, please send a message via **text/WhatsApp or call: 0757-219-898**

Supplement 6: DCE Pre-screening Script

DCE RECRUITMENT SCRIPT

Hello, my name is…I'm a Research Assistant at KEMRI and we are doing a study to learn about what people in your community think about pre-exposure or PrEP. PrEP is a pill that you can take daily to reduce your risk of getting HIV.

We would like to learn about your preferences for getting them delivered to you from an online pharmacy. This means a pharmacy will deliver drugs to clients using a courier, so they do not need to travel to a pharmacy. Hearing about your preferences will help us design new models of PrEP delivery for your community.

If you are interested, we will meet you in a location of your choice to complete the survey. The survey is confidential and takes about 60 minutes. You will receive Ksh 1000 for your time and effort.

Are you interested in taking part in this survey?

[IF NO] Thank you very much and have a good [morning/afternoon evening]

[IF YES] That's great! I'm now going to ask you questions to make sure that you are eligible to be in this study. This will take about 10 minutes. If you are eligible, I'll schedule a time for someone from our research team to come to a place that is convenient for you, so you can complete the survey.

Before we begin, do you have any questions?

[Verifying Source of information] Thank you, tell me how you got to know about us?

[If they do not know, advise them on how they can access the study information on the MyDawa website]

[If the participant knows about PrEP study, go ahead and start the pre-screening process]

[Pre-screening process]

[What is your age?] If below the age of 18, inform the participant they are ineligible and explain the reasons why.

[What was the result of your last HIV test?] If positive, inform the participant they are ineligible for the survey.

Are you currently taking PrEP to prevent HIV?

[If eligible for the study participation. Please continue with scheduling their study visit] Thank you for responding to the questions. You are eligible to participate in the study. I would like to ask you for some information on how I can contact you and when and where we can schedule the study.

For peer review only

[Write down the first name and surname]

[Write done the phone number where we can reach them and how they would prefer to be contacted] Through a phone call, SMS, or WhatsApp.

[Which day of the week are you available for the interview] Write down the day given by the participant.

[Time of scheduled study visit] Someone from our research team will come to a location of your choice to administer the study survey. Where would you like to meet?

[Enter study visit] Inform the study participant of the need for privacy, security, and confidentiality.

Thank you so much for your time and for agreeing to participate. Someone from our research team will contact you on the day of the survey for final arrangements. If you have any questions before the day of the interview you can reach the study through our contact number.

For peer review only

Supplement 7: DCE Data Management R Codes

```
---
title: "DCE Data Check - R Script"
date: "`r Sys.Date()`"
output:
  html_document:
    toc: true
    toc_float: true
---

# Load dataset
rm(list=ls())

# Set up R environment
## install.packages("stringr", "tidyverse", "dplyr", "pander")
library(stringr)
library(tidyverse)
library(dplyr)
library(pander)

# Set up functions
`%notin%` <- Negate(`%in%`)
table_na <- function(x){table(x, useNA = "always")}

# **NOTE**: Change my_path to your own directory
## my_path = "C:/Users/Yilin Chen/Desktop/2021 DCE ePharm/Data collection"
my_path = "~/Desktop/My projects/2021 DCE ePharm/Data collection" # Change this to the
path of your folder
setwd(my_path)

# Load data
## **NOTE**: Please change the dataset name accordingly
file_name = "GatesePharmDCESurvey_data_pre-launch_5.5.csv" # Change file_name to the
name of csv file
dat <- read.csv(file_name)
dat$x = seq(nrow(dat))

# Select relevant survey records in the data collection time window
dat$day <- str_split_fixed(dat$sys_StartTime, " ", 4)[,1]
dat$month <- str_split_fixed(dat$sys_StartTime, " ", 4)[,2]
dat$year <- str_split_fixed(dat$sys_StartTime, " ", 4)[,3]
dat$date <- as.Date(with(dat,paste(year,month,day,sep="-")),"%Y-%b-%d")
```

For peer review only

```
## **NOTE**: Please change the date_start and date_end below for specific data collection time window
date_start = "2022-03-09" # Change this to the start of time window
date_end = "2022-04-26"   # Change this to the end of time window
dat2 <- dat %>% filter(date >= date_start & date <= date_end) # example: from 2022-03-09 to 2022-04-26

# Select relevant survey records that contain "230" in the PTID
## **NOTE**: Please revise "230" to the starting number/word used in the actual survey
num_start = "230" # Change this to the actual starting number/word
dat2 <- dat2[str_detect(dat2$PTID1, num_start), ]
dat2 <- dat2[complete.cases(dat2[ , c('PTID1')]), ]
nrow(dat2)

# Data checks
## 1. Check for number of interviews

######################################
# 1. Check for number of interviews #
######################################

# Show PTIDs - first check if there are any missing PTIDs
unique(dat2$PTID1)

# Count the total number of interviews
length(unique(dat2$PTID1))

# Count the total number of interviews by RAs
dat2 %>% group_by(RAInitials) %>% tally() %>% pander()

## 2. Check for duplicate PTIDs and incomplete surveys

########################################################
# 2. Check for duplicate PTIDs and incomplete surveys #
########################################################

# Lists all duplicate records using the variable PTID1 and print out these PTIDs
count_pid <- as.data.frame(table(dat2$PTID1))
dup_pid <- as.character(count_pid$Var1[(count_pid$Freq > 1)])
dup_pid

# Create a subset with duplicate PTIDs
dat3a <- dat2 %>% filter(PTID1 %in% dup_pid) %>% select(sys_RespNum, sys_StartTime, sys_EndTime, sys_LastQuestion, RAInitials, PTID1, PTID2)
```

For peer review only

```
dat3a <- dat3a[order(dat3a$PTID1), ]

# Incomplete surveys: show PTIDs when the last answered question is not
"TeminateQuestionnaire" or "TerminateEligibility"
incomplete_id <- dat2 %>%
  filter(sys_LastQuestion %notin% c("TerminateQuestionnaire", "TerminateEligibility")) %>%
  select(x)

# List PTIDs of incomplete surveys
dat3 <- dat2 %>% filter(x %in% incomplete_id$x)
incomplete_pid <- unique(dat3$PTID1)
incomplete_pid

# Create a subset with incomplete surveys
dat3b <- dat3 %>% select(sys_RespNum, sys_StartTime, sys_EndTime, sys_LastQuestion,
RAInitials, PTID1, PTID2)
dat3b <- dat3b[order(dat3b$PTID1), ]

# Combine incomplete records and duplicate records
dat3c <- dat3a %>% full_join(dat3b, by = c("sys_RespNum", "sys_StartTime", "sys_EndTime",
"sys_LastQuestion", "RAInitials", "PTID1", "PTID2"))
dat3c$status = ifelse(dat3c$sys_LastQuestion %notin% c("TerminateQuestionnaire",
"TerminateEligibility"), "incomplete", "complete")
dat3c

# Save duplicate surveys to a csv file and add a date to the file name
## Most of duplicate surveys might be due to incompleteness
## **NOTE**: Change the date_now below to the current date
date_now = "05.12.2022" # Change to the current date
write.csv(dat3c, paste0("GatesePharmDCESurvey_duplicate_", date_now, ".csv"))

# You could also review these duplicate records on Sawtooth or original csv file, and figure out
reasons for duplicates
## Admin site link: https://ePharmacyPrEP.sawtoothsoftware.com/english/admin.html


## 3. Check for specific responses
### Part 1: Eligibility Assessment

####################################
# 3. Check for specific responses #
####################################

# Create a subset of eligible participants and interested variables for exploratory analysis
```

For peer review only

```
dat4 <- dat2 %>% filter(sys_LastQuestion == "TerminateQuestionnaire")
length(unique(dat4$PTID1)) # check number of unique participants
dat5 <- dat4[, 19:156] # check if this includes all variables of interest (starting from "RA initials" variable)

# Part 1: Eligibility Assessment (Q1-4)
apply(dat5[, 4:8], 2, table_na)

## AgeYears: should be >= 18 years old
dat5$PTID1[dat5$AgeYears < 18]
## HIVTestDate_Month: should not be NA and should be numeric from 1-12
dat5$PTID1[is.na(dat5$HIVTestDate_Month)]
## HIVTestDate_Year: should not be NA and should be year
dat5$PTID1[is.na(dat5$HIVTestDate_Year)]
## HIVTestResult: should not include 2 - "Positive"
dat5$PTID1[dat5$HIVTestResult == 2]

### Part 2: PrEP Knowledge and Interest

# Part 2: PrEP Knowledge and Interest (Q5-7)
apply(dat5[, 9:14], 2, table_na)

### Part 3: Discrete Choice Experiment

# Part 3: Discrete Choice Experiment
apply(dat5[, 15:30], 2, table_na) # shouldn't have any NAs

### Part 6: E-Pharmacy Engagement and HIV Self-Testing

# Part 6: E-Pharmacy Engagement and HIV Self-Testing (Q8-29)
## e-Pharmacy engagement
apply(dat5[, 31:53], 2, table_na)

## ePharmEngDetail_7: if "Other" category = 1,  there should be text answers
table(dat5$ePharmEngDetail_7)
## PreferObtainPrEP_5_other: if PreferObtainPrEP == 5, there should be text answers
table(dat5$PreferObtainPrEP_5_other[dat5$PreferObtainPrEP == 5])
## AddlProducts_7: if "Other" category = 1,  there should be text answers
table(dat5$AddlProducts_7)
## PrEPRefillPref_4_other: if PrEPRefillPref == 4, there should be text answers
table(dat5$PrEPRefillPref_4_other[dat5$PrEPRefillPref == 4])

## WTP questions
apply(dat5[, 54:60], 2, table_na)
```

For peer review only

```
## Preferences for online PrEP delivery
apply(dat5[, 61:84], 2, table_na)

### Part 7: Participant Demographics

# Part 7: Participant Demographics (Q30-41)
apply(dat5[, 85:112], 2, table_na)

## FamPlanMethod_14_other: if FamPlanMethod_14 == 1, there should be text answers
table(dat5$FamPlanMethod_14_other[dat5$FamPlanMethod_14 == 1])
## AccessFamPlan_6_other: if AccessFamPlan == 6, there should be text answers
table(dat5$AccessFamPlan_6_other[dat5$AccessFamPlan == 6])

### Part 8: Sexual Behaviors

# Part 8: Sexual Behaviors (Q42-61)
apply(dat5[, 113:138], 2, table_na)

## SexPartType3Mo_4_other: if SexPartType3Mo == 4, there should be text answers
table(dat5$SexPartType3Mo_4_other[dat5$SexPartType3Mo == 4])
```

For peer review only

BMJ Open

Assessing preferences for HIV pre-exposure prophylaxis (PrEP) delivery services via online pharmacies in Kenya: protocol for a discrete choice experiment

Journal:	*BMJ Open*
Manuscript ID	bmjopen-2022-069195.R1
Article Type:	Protocol
Date Submitted by the Author:	15-Feb-2023
Complete List of Authors:	Chen, Yilin; University of Washington, The Comparative Health Outcomes, Policy, and Economics (CHOICE) Institute<br>Saldarriaga, Enrique; University of Washington, The Comparative Health Outcomes, Policy, and Economics (CHOICE) Institute<br>Montano, Michalina; Fred Hutchinson Cancer Research Center, Vaccine and Infectious Diseases Division (VIDD); University of Washington, Department of Global Health<br>Ngure, Kenneth; Jomo Kenyatta University of Agriculture and Technology, School of Public Health<br>Thuo, Nicholas; Partners in Health and Research Development<br>Kiptinness, Catherine; Partners in Health and Research Development<br>Rafferty, Maeve; MYDAWA<br>Terris-Prestholt, Fern; London School of Hygiene and Tropical Medicine Faculty of Public Health and Policy, Department of Global Health and Development<br>Stergachis, Andy; University of Washington, The Comparative Health Outcomes, Policy, and Economics (CHOICE) Institute; University of Washington, Department of Global Health<br>Mugambi, Melissa; University of Washington, Department of Global Health<br>Ortblad, Katrina; Fred Hutchinson Cancer Research Center, Public Health Science Division<br>Sharma , Monisha; University of Washington, Department of Global Health
**Primary Subject Heading**:	HIV/AIDS
Secondary Subject Heading:	Global health, Health economics, Health services research, Patient-centred medicine
Keywords:	HIV & AIDS < INFECTIOUS DISEASES, Health economics < HEALTH SERVICES ADMINISTRATION & MANAGEMENT, Public health < INFECTIOUS DISEASES, Telemedicine < BIOTECHNOLOGY & BIOINFORMATICS, International health services < HEALTH SERVICES ADMINISTRATION & MANAGEMENT, HEALTH SERVICES ADMINISTRATION & MANAGEMENT

For peer review only - http://bmjopen.bmj.com/site/about/guidelines.xhtml

Assessing preferences for HIV pre-exposure prophylaxis (PrEP) delivery services via online pharmacies in Kenya: protocol for a discrete choice experiment

Yilin Chen[1], Enrique Saldarriaga[1], Michalina A. Montano[2,3], Kenneth Ngure[4], Nicholas Thuo[5], Catherine Kiptinness[5], Maeve Rafferty[6], Fern Terris-Prestholt[7], Andy Stergachis[1,3], Melissa Latigo Mugambi[3], Katrina F. Ortblad[8], Monisha Sharma[3]

[1]The Comparative Health Outcomes, Policy, and Economics (CHOICE) Institute, University of Washington, Seattle, USA

[2]Vaccine and Infectious Diseases Division (VIDD), Fred Hutchinson Cancer Center, Seattle, USA

[3]Department of Global Health, University of Washington, Seattle, USA

[4] School of Public Health, Jomo Kenyatta University of Agriculture and Technology

[5]Partners in Health and Research Development, Thika, Kenya

[6]MYDAWA, Nairobi, Kenya

[7]Department of Global Health and Development, Faculty of Public Health and Policy, London School of Hygiene and Tropical Medicine, London, UK

[8]Public Health Science Division, Fred Hutchinson Cancer Center, Seattle, USA

Correspondence to:
Yilin Chen
Health Sciences Building, 1956 NE Pacific St H362, Seattle, WA 98195, USA
ylchen95@uw.edu

For peer review only

For peer review only - http://bmjopen.bmj.com/site/about/guidelines.xhtml

Keywords: PrEP, HIV prevention, mHealth, Discrete Choice Experiment, pharmacies, online delivery, preferences

Word count: 3696; **Abstract:** 293; **Tables:** 1; **Figures:** 2; **Supplement files:** 6.

For peer review only

ABSTRACT

Introduction: Pre-exposure prophylaxis (PrEP) is highly effective at preventing HIV acquisition, but coverage remains low in high prevalence settings. Initiating and continuing PrEP via online pharmacies is a promising strategy to expand PrEP uptake but little is known about user preferences for this strategy. We describe methods for a discrete choice experiment (DCE) to assess preferences for PrEP delivery from an online pharmacy.

Methods and analysis: This cross-sectional study is conducted in Nairobi, Kenya, in partnership with MYDAWA, a private online pharmacy retailer with a planned sample size of >400 participants. Eligibility criteria are: ≥18 years, not known HIV-positive, and are interested in PrEP. Initial DCE attributes and levels were developed via literature review and stakeholder meetings. We conducted cognitive interviews to assess participant understanding of the DCE survey and refined the design. The final DCE used a D-efficient design and contained four attributes: PrEP eligibility assessment, HIV test type, clinical consultation type, and user support options. Participants are presented with 8 scenarios consisting of two hypothetical PrEP delivery services. The survey was piloted among 20 participants before being advertised on the MYDAWA website on pages displaying products indicating HIV risk (e.g. emergency contraception). Interested participants call a study number and those screened eligible meet a research assistant in a convenient location to complete the survey. The DCE will be analyzed using a conditional logit model to assess average preferences and mixed logit and latent class models to evaluate preference heterogeneity among subgroups.

Ethics and dissemination: This study was approved by the University of Washington Human Research Ethics Committee (STUDY00014011), the Kenya Medical Research Institute, Nairobi County (EOP/NMS/HS/128), and the Scientific and Ethics Review Unit in Kenya

For peer review only - http://bmjopen.bmj.com/site/about/guidelines.xhtml

For peer review only

(KEMRI/RES/7/3/1). Participation in the DCE is voluntary and subject to completion of an electronic informed consent. Findings will be shared at international conferences and peer-reviewed publications, and via engagement meetings with stakeholders.

Strengths and limitations of this study

- The study uses a discrete choice experiment (DCE) to elicit the user preferences on initiating and continuing pre-exposure prophylaxis (PrEP) from an online pharmacy in Kenya.
- DCE results will provide a quantitative assessment of how users value different characteristic of an online PrEP delivery model; this can inform the design of online HIV PrEP delivery programs to optimize uptake.
- We are collecting information on the willingness-to-pay for each component of ePrEP services, which can inform policy decisions and resource allocation.
- DCE scenarios are hypothetical, and individuals' stated preferences may not represent their actual choices in real-world settings.
- Online PrEP delivery requires access to internet and ability to pay for HIV self-testing and PrEP delivery services; therefore, this platform is designed to growing middle class in Kenya, but other PrEP service delivery strategies are needed to reach those with low-income and/or lack of internet access.

For peer review only - http://bmjopen.bmj.com/site/about/guidelines.xhtml

INTRODUCTION

Kenya has one of the largest HIV epidemics globally, with a national prevalence of 4.2% [1-3]. Despite significant strides in HIV prevention, approximately 32,000 new HIV infections occurred in Kenya in 2021[4]. Pre-exposure prophylaxis (PrEP) is highly effective at preventing HIV acquisition and is recommended by the World Health Organization (WHO) and the Kenya Ministry of Health for persons at risk of HIV [5]. However, PrEP coverage remains low among those with risk indications [6-7]. Currently, PrEP is mainly delivered through health care facilities and barriers to widespread use include long wait times and travel distance, limited hours of operation, privacy concerns, stigma, and understaffing [8-9]. Additionally, PrEP demonstration projects in East Africa have found low PrEP retention among those who initiate PrEP at clinics, due in part to challenges returning to the clinic for frequent continuation visits [10-11].

Initiating and continuing PrEP online is a novel approach that could expand PrEP coverage to individuals not accessing clinic-based PrEP and improve PrEP continuation [12]. Delivery of PrEP services closer to the client can reduce opportunity costs associated with clinic-based PrEP provision. A growing telehealth ecosystem in sub-Saharan Africa, recently expanded due to the COVID-19 pandemic, provides private and convenient options for remote consultation, purchasing, and delivery of medications to patients [13-15]. Coupled with Kenya's national commitment to PrEP roll-out, online PrEP delivery is a promising strategy to expand PrEP access. Our team is currently conducting a pilot study of the first online care model for PrEP delivery (NCT05377138) through collaboration with Kenya’s first e-pharmacy retailer MYDAWA [16].

For peer review only - http://bmjopen.bmj.com/site/about/guidelines.xhtml

Successful PrEP scale up requires an understanding of user preferences to tailor services to optimize uptake, adherence, and retention. There are several potential options for structuring PrEP delivery services *via* an online pharmacy including the use of different HIV testing modalities and user support options. As online PrEP delivery is not yet available in Kenya, user preferences for components of this service are unknown. Discrete Choice Experiments (DCEs) are an ideal method for assessing user preferences in cases when there is no observed behavior. With a foundation in economic theory, DCEs assume that individuals choose the service that maximizes their expected benefit [17]. DCEs evaluate how individuals value selected features of a service by repeatedly asking them to choose between different sets of hypothetical alternatives. Resulting data can provide a quantitative assessment of user preferences including the relative importance of each attribute and likelihood of service uptake, which can help inform policy decisions and resource allocation. DCEs have been applied to a range of health policy decisions and are increasingly used to assess preferences for PrEP services. Prior DCEs assessing PrEP have examined user preferences for dosing regimen, type of PrEP products, side effects, cost, dispensing venue, and support services [18-23]. However, this is the first study to assess user preferences for ePrEP delivery.

Here we describe the design and methods from our ongoing DCE to assess user preferences for PrEP delivery from an online pharmacy in Kenya. This study adds to the small but growing literature of DCE protocols for HIV prevention [24-28] and additionally provides detailed information on DCE attribute selection and refinement, materials used for staff training, and methods for participant recruitment. The methods described along with slide decks of training materials and survey guides can assist other researchers interested in designing DCEs for

For peer review only - http://bmjopen.bmj.com/site/about/guidelines.xhtml

HIV prevention in Africa. The results of this DCE can inform the design of online PrEP delivery models to increase PrEP uptake among persons at risk of HIV.

METHODS AND ANALYSIS

Setting and participants

This study is conducted in Nairobi, Kenya, in partnership with MYDAWA, Kenya's first licensed online pharmacy [29-30]. MYDAWA's online platform provides affordable access to wellness projects, prescription and over-the-counter medicines delivered directly to the consumer, including products related to sexual health (e.g. HIV self-test kits and emergency contraception). The majority of MYDAWA's customer base is located in Nairobi [16, 29]. The target population is selected to closely mirror persons who would likely be clients of ePrEP delivery. Inclusion criteria are: age ≥18 years, not known to be HIV-positive, interested in PrEP, and able and willing to provide informed consent.

Patient and public involvement

We developed the care pathway for online PrEP service delivery in collaboration with Kenyan stakeholders, including members from the Ministry of Health, implementing organizations, researchers, professional organizations. We refined list of attributes and levels via stakeholder discussions with individuals in Kenya with expertise in HIV prevention and PrEP delivery and representatives from MYDAWA. Finally, we plan to share study findings with both local and international audiences at conferences. We will disseminate results at engagement meetings with key informants including Kenya's National AIDS and STIs Control Programme (NASCOP), patients groups, service providers, and other stakeholders.

For peer review only - http://bmjopen.bmj.com/site/about/guidelines.xhtml

Care pathway for online PrEP service delivery

We collaboratively developed a care pathway for online PrEP service delivery, based on an ongoing model of pharmacy-based PrEP service delivery in Kenya [31]. The core components of the online PrEP service delivery care pathway include: 1) PrEP eligibility assessment: Based on Kenya's National AIDS and STI Control Programme (NASCOP) PrEP Rapid Assessment Screening Tool (RAST), routinely used at public clinics in Kenya to assess HIV risk and PrEP eligibility. 2) HIV testing: Individuals who are potentially eligible for PrEP complete an HIV test to ensure they are HIV-negative prior to starting PrEP; 3) Clinical consultation and PrEP prescribing: Individuals testing HIV-negative undergo a medical risk assessment with a clinical officer to ensure PrEP is safe for them and receive a PrEP prescription; 4) PrEP delivery: A one-month supply of PrEP is delivered to a setting of the client's choice at initiation. For all future refills, clients receive a 3-month PrEP supply, consistent with the guidelines for PrEP dispensing at public clinics in Kenya. Clients complete the HIV testing process described above prior to each refill visit; and 5) PrEP support: Individuals can to opt-in to receive ongoing support to answer questions about PrEP use and to report concerns and side effects. Each step in the care pathway for online PrEP delivery can be optimized to align with user preferences and was evaluated for inclusion in the final DCE.

Overview of DCE approach

DCE participants are presented with a series of scenarios consisting of two or more hypothetical services with different characteristics "attributes" that have different variations or "levels" and are asked to choose their preferred services. Surveys generally contain 7-10 scenarios with varying levels for each attribute. The experimental design determines the scenario pairs and the

For peer review only - http://bmjopen.bmj.com/site/about/guidelines.xhtml

series of questions presented to participants. Analysis of DCEs can quantify the impact of each aspect of a service on participants' preference and assess the trade-offs they are willing to make to obtain their preferred service.

Design and administration of the DCE is divided into several stages (Figure 1): 1) formative stage, 2) refinement stage, 3) experimental design, 4) survey administration, and 5) data analysis and dissemination. First, in the formative stage, we selected attributes and levels for the DCE and developed survey questions to accompany the DCE. Second, in the refinement stage, we conducted cognitive interviews to assess participant understanding of the hypothetical scenarios and associated illustrations (see Supplement 1 [cognitive interview guide] and Supplement 2 [staff training materials]). We refined our DCE attributes and levels based on participant feedback. In the third stage, we developed the experimental design using a D-optimal design with 8 choice tasks per participant. Our study design followed the ISPOR Good Research Practice for Conjoint Analysis [32]. We piloted the DCE to estimate the time needed to complete the questionnaire and assess participant fatigue. The survey administration adopts a hybrid approach, including in-person administration by research assistants or self-administration by online participants to achieve sufficient sample size and reach our target population more efficiently. Finally, we conduct data analysis to estimate average preferences of each attribute level and explore heterogeneity of responses.

Software

We use Research Electronic Data Capture (REDCap) electronic data capture tools hosted at the University of Washington for screening and informed consent data [33]. The DCE questionnaire is programmed in Sawtooth Software's Lighthouse Studio modules for general questionnaires

For peer review only - http://bmjopen.bmj.com/site/about/guidelines.xhtml

and choice-based conjoint scenario designs, and hosted on the Sawtooth servers [34]. We contacted a Sawtooth representative and obtained the software at a discounted price. Screening and informed consent forms are constructed as separate, chained surveys in REDCap. Completion and submission of informed consent forms via REDCap directs online participants to the Sawtooth questionnaire via an external link for online participants. DCE questionnaires are administered via Sawtooth's offline data capture application for in-person participants.

DCE procedures

Stage I: Formative phase –selection of attributes and levels, development of data collection instruments, training

Collaborative attribute and level selection: We developed an initial list of attributes and levels based on the care pathways of the ePrEP delivery model and a review of the PrEP literature [5, 7, 35-37]. This list was refined during stakeholder discussions with individuals in Kenya with expertise in HIV prevention and PrEP delivery and representatives from MYDAWA. Five attributes were included in the formative list: PrEP eligibility assessment, HIV test type, clinical consultation, user support options, and cost of PrEP delivery. Each attribute consists of 2-3 levels (Table 1).

Table 1. DCE attributes and levels

**Attribute & Description**	**Levels**
**PrEP eligibility assessment**<br>Method for conducting client eligibility assessment for PrEP	Online self-assessment using screening questions (phone number in case of questions)
	Guided assessment with a remote clinical provider (via a phone call or WhatsApp)
**HIV test type**<br>Type of HIV test delivered for PrEP	Oral Fluid HIV self-test (at setting of your choice)

For peer review only - http://bmjopen.bmj.com/site/about/guidelines.xhtml

initiation	Blood-based HIV self-test (at setting of your choice)
	Healthcare provider administers HIV Test at setting of your choice (blood-based)
**Clinical consultation for prescribing PrEP**<br>Consultation needed to prescribe PrEP	Remote clinical consultation with provider (via a phone call or video chat)
	In-person clinical consultation with provider after completing HIV test (at a setting of your choice)
**User support options for PrEP**<br>Method for discussing your questions for PrEP with a healthcare provider	SMS
	Phone/video call
	Email

Abbreviations: PrEP, pre-exposure prophylaxis.

Questionnaire development: We developed a survey to accompany the DCE which includes the following: PrEP eligibility assessment, PrEP interest and knowledge, online pharmacy and HIV testing interest, knowledge, and engagement, and participant demographics, and sexual behavior (see Supplement 3 for survey questionnaire). We also assessed maximum willingness to pay (WTP) for different components of online PrEP delivery including oral vs blood-based HIV self-testing, remote clinical encounter, and delivery of PrEP drugs. We also ask about maximum WTP for the entire package of ePrEP delivery. The structure of the WTP questions was informed by a literature review [38-40]. The DCE itself consists of a brief overview of DCEs, definition of attributes and levels, and a series of choice tasks. For each DCE scenario, participants are first asked to choose their preferred service followed by an opt-out question: “Would you choose to get PrEP using this service if it were available?” (Figure 2).

Staff training. We conducted a training with study investigators, data mangers, and in-country research staff responsible for administering the survey; it was held over Zoom split over two half

For peer review only - http://bmjopen.bmj.com/site/about/guidelines.xhtml

day sessions to accommodate the time difference between Seattle and Kenya. The training consisted of presentations on the background of HIV in Kenya, the role of PrEP for HIV prevention, barriers to accessing PrEP via current delivery modalities and the potential ePrEP delivery to increase PrEP coverage. We explained the DCE administration procedures and instruments, including the REDCap pre-screening form and the process for scheduling in-person visits, REDCap e-consent forms, and the Sawtooth survey. On the second day, we discussed the process for conducting cognitive interviews and piloting and launching the DCE. Study staff practiced administering the cognitive interviews and DCE instruments in groups of two and provided feedback on study instruments (see Supplement 2 for slide deck and training materials).

Stage II: Refinement of DCE attributes and levels, and cognitive interviews

<u>DCE image design and cognitive interviews:</u> We utilized images to pair with each level of DCE attributes to make the survey more engaging and understandable. Most of the images were adapted from the Noun Project, a free online image bank [41]. We chose simple images to minimize possibility of biasing participants. For example, images of healthcare providers are depicted with neutral expressions and their gender is not specified. In addition, images of fingerpicks for HIV testing scenarios are not portrayed with big needles or large amounts of blood.

<u>Cognitive interviews</u>: We recruited individuals from the HIV research community in Kenya for cognitive interviews to assess the clarity of DCE images, DCE scenarios, and WTP questions (see Supplement 1 for cognitive interview guide). Participants were asked to interpret each attribute image and assess its accuracy in conveying the specified DCE level. They were then presented with alternative images for each attribute and asked if any were a better match for the

For peer review only - http://bmjopen.bmj.com/site/about/guidelines.xhtml

attribute description than the current image. The cognitive interviews also presented participants with two example DCE choice tasks to assess their understanding of the scenarios and ease completion. Additionally, we assessed participants' understanding of WTP questions for different aspects of the ePrEP delivery including HIV testing, clinical encounter, and delivery of PrEP drugs. We initially sought to audio-record the cognitive interviews but after consultation with the in-country staff, decided that it may hinder participants' ability to speak freely; therefore, study staff took notes during the interviews.

Stage III: Experimental design and pilot testing

We used a D-efficient design to construct the DCE [32]. The algorithm behind this design identifies the sub-set of the most meaningful comparisons across profiles from all possible choice tasks – 1,296 (i.e., $(2 \times 3 \times 2 \times 3)^2$ in our study [42]. The design enhances data efficiency and attempts to balance statistical power and sample size [43-44]. First, we considered the complete specification of the utility functions based on the chosen attributes and levels and multinomial logit as the analytical model [42]. We defined a main-effects utility function for each of the two alternatives in a choice task. The structure for the i-th utility function, U is defined as:

$$U_i = V(\beta, X_i) + \epsilon_i$$
$$V(\beta, X_i) = \beta_1 * X_1 + \ldots + \beta_k * X_k$$

Where $V(\beta, X_i)$ is the deterministic portion of the utility and a function of the k attribute-levels considered in the alternative; the β coefficients represent the average preference weight that the sample places on each attribute-level. The error term, ϵ_i, indicates the inability to perfectly measure the utility of each alternative, which is consistent with random utility theory. We did not use priors for the utility-function coefficients since none of the selected attributes had

For peer review only - http://bmjopen.bmj.com/site/about/guidelines.xhtml

a natural order (i.e., an ascending order example is 'pain' as we assume that most people prefer low to high pain).

Each scenario consists of a forced-choice task, followed by an opt-out question: "Would you choose to get PrEP using this service if it were available?" (Figure 2) [45]. The goal of the opt-out option was to represent the status quo where participants can decide against uptake of a new product [46]. Participants who select "no" express their preference for the status quo. This format allows us to gathering data about user preferences while not drawing too much attention to the opt-out. Studies suggest potential for cognitive biases to preserve the status quo in 3-alternative choice tasks [47-48].

Sample size: The DCE literature has not yet reached a consensus on the best method to estimate the sample size required to return meaningful, statistically robust parameter estimates [49-50]. Some experts suggest using a minimum sample size of 300 [49]. Johnson and Orme provide another rule of thumb using an equation that considers the number of choice questions, alternative scenarios presented, and attributes and levels [50-51]. Additionally, the more tasks per participant, the smaller sample size required to achieve comparable levels of uncertainty in coefficients. During the formative phase of our study, most participants stated that they could answer between 7 to 10 choice tasks before becoming fatigued.

A criticism to rules of thumb for DCE sample estimation is the lack of robustness to different product characteristics or consumers behavior [52]. Given this uncertainty, we implemented a scenario analysis considering all possible tasks per participant, from 6-10 under a sample size varying from 200-500 in 50 increments. For each scenario, we evaluated the standard error of coefficients and the D-efficiency measure for the overall design. We found that improvements were diminishing above a sample size of 350. Therefore, we chose a design with 8

For peer review only - http://bmjopen.bmj.com/site/about/guidelines.xhtml

choice tasks per participant and a sample size of 400, which had an expected standard deviation 0.049 per coefficient. This sample size was slightly higher than 350 to account for the possibility of preference heterogeneity and incomplete DCE surveys [32, 53].

Stage IV: Pilot testing, DCE recruitment, and administration

<u>Pilot testing and survey refinement:</u> After revising the DCE survey based on feedback from the cognitive interviews, we piloted it in two phases. First, we administered the survey to individuals from the HIV research community in Kenya. Based on participants' feedback, we found the cost attribute seemed to be a strong driver of participant preferences, with many participants selecting the cheaper PrEP service regardless of other attributes. To avoid the possibility of having one attribute overwhelming participant decision making, we removed the cost attribute from the final version of the DCE and instead assessed cost preferences only through direct WTP questions included in the survey (Table 1). Additionally, we moved the DCE scenario questions to the beginning of the survey to minimize participant fatigue. Since several participants expressed hesitancy in answering sensitive sexual behavior questions at the beginning of the survey, we moved these questions to the end to provide increased opportunity for staff to develop rapport with participants; we also gave participants the option of self-administering these questions by taking the tablet and filling out this section on their own. Finally, participants found the DCE eligibility questions from the HIV Risk Assessment Tool to be invasive and too sensitive to answer over the phone, so we revised the pre-screening process to only ask if participants are interested in obtaining PrEP. In the second phase, we piloted the survey to 20 individuals recruited through the MYDAWA platform using study eligibility criteria. We assessed data quality, survey flow and time needed for survey administration.

For peer review only - http://bmjopen.bmj.com/site/about/guidelines.xhtml

Recruitment: Similar to other DCEs in the literature, we utilize online recruitment strategies [23-24]. Participants are recruited through banner ads on the MYDAWA online pharmacy website. To target persons at risk of HIV acquisition, only those who click on HIV self-test kits on the MYDAWA platform see the online recruitment banners. In addition, persons who purchase HIVSTs from MYDAWA are mailed a flyer with their purchases that advertises the DCE (see Supplement 4 for the recruitment banner). We are also planning to expand DCE recruitment by advertising on social media pages that provide information on HIV prevention as well as through MYDAWA call in center where persons can access support related to STI purchases.

Survey administration: Interested participants can voice call or send an SMS to the study phone number provided on recruitment materials. Study staff assess eligibility via a phone pre-screening and schedule time to meet participants at a convenient location to complete the survey (see Supplement 5 for the pre-screening script). Participants are reimbursed 1,000 KES (~$8.70 USD) for their time. The in-person data collection of this study was initiated on 1-June-2022 and expected to be completed by 28-Feb-2023. We plan to launch the online self-administered DCE after we finish data collection for the in-person component. For the online DCE, participants will follow the DCE link on the recruitment banner to an online screening tool, self-administered via REDCap survey. Once the screening survey is submitted, eligible participants will automatically be directed to complete online consent forms via REDCap, and subsequently the DCE questionnaire *via* Sawtooth.

Stage V: Data management and data analysis

Data are stored in a central location on the Sawtooth administration site, which allow users to directly examine survey responses or download a csv file for further analysis. We monitor data

For peer review only - http://bmjopen.bmj.com/site/about/guidelines.xhtml

quality on a weekly basis, include checking total number of interviews, duplicate entries or incomplete surveys, and substantial missingness or outliers in responses (see Supplement 6 for R script used for data management).

To analyze the data, we will fit a conditional logit model with an alternative specific constant for the opt-out. The coefficients (i.e. preference weights) represent the average relative utility for each attribute-level, compared to the reference [54-55]. In addition, we will generate a ranking of importance across attributes calculated as the ratio of their preference weights over the preference weights of the attribute with the lowest impact in the decision-making process (i.e., the attribute with the lowest preference weights). This ranking will provide information about the relative importance of each attribute, and respondents' willingness to trade one attribute for another. We will conduct a two-part analysis for the main model: First, we will analyze only the forced-choice data (i.e., ignoring the opt-out question), and second, we will use a mixed logit regression model (i.e., random parameters logit) to account for preferences' heterogeneity in all coefficients. We will qualitatively assess if our conclusions are consistent in both analyses and compare the goodness of fit of the models using Akaike information criteria (AIC) and Bayesian information criteria (BIC).

Further, we will elicit preference heterogeneity by participant demographics and other characteristics. Latent class analysis will be performed to examine variation in preference by using the data to identify groups of respondents with similar preferences [56]. We will fit to 2 to 10 classes and assess the goodness of fit of each one using McFaddens pseudo r-squared [54], log likelihood test, and adjusted-AIC and -BIC. In a scenario in which a covariate is an important predictor of class membership, we will assess its potential impact as an effect modifier and assess the results separately for each category of the covariate.

For peer review only - http://bmjopen.bmj.com/site/about/guidelines.xhtml

Descriptive analysis will be conducted to summarize participants' demographic characteristics, sexual behaviors, WTP for online PrEP services, and online pharmacy interest/engagement. Data analysis will be conducted using Sawtooth's built-in analysis capabilities and R version 4.1.2.

ETHICS AND DISSEMINATION

Ethics considerations

This study was approved by the University of the Washington Human Research Ethics Committee (STUDY00014011), the Kenya Medical Research Institute (KEMRI), Nairobi County (EOP/NMS/HS/128) and the Scientific and Ethics Review Unit (SERU) in Kenya (KEMRI/RES/7/3/1). Participation in the DCE is voluntary and subject to completion of an electronic informed consent. Respondents' personal information is kept secure and confidential. Participants are not required to use their real names and we do not collect any identifiable information. Participants are told that they can withdraw from the survey at any time.

Dissemination

Findings will be shared at international conferences and peer-reviewed publications. We will disseminate results at engagement meetings with key informants including Kenya's NASCOP and other stakeholders. Preliminary results will be used to inform the design of the ePrEP Kenya Pilot study (NCT05377138) which will evaluate uptake and continuation rates for online PrEP delivery via MYDAWA in Nairobi, Kenya. Our results can be utilized to design online PrEP delivery models to optimize PrEP uptake in Kenya and similar settings.

For peer review only - http://bmjopen.bmj.com/site/about/guidelines.xhtml
For peer review only

Acknowledgements

We would like to thank Peter Mogere from KEMRI and Daniel Were from Jhpiego for participating in stakeholder discussions. We thank Tony Wood, Lisbet Charana, and other MYDAWA staff for their support of this project and assistance with promoting the study on their website. We would like to acknowledge Dr. Nicole Young (Program Officer, New Product Planning & Introduction, HIV/TB) and her colleagues at the Bill & Melinda Gates Foundation for their support in developing and funding this work [Grant #: INV-037646]. We are grateful to study staff and survey participants.

Contributors: MS, KO, and MLM conceived the study. YC, ES, MM, KN, NT, CK, MR, FT-P, AS, MLM, KO, MS contributed to its design. YC, ES, MM, and MS wrote the first draft of the protocol and designed the DCE tasks. All authors contributed to the drafting and editing of the manuscript and approved the final version.

Competing interests: The authors report no conflicts of interest.

Funding: This study was funded by the Bill and Melinda Gates Foundation [Grant #: INV-037646]. The funders had no role in study design, data collection, analysis, writing of the report, nor the decision to submit for publication.

For peer review only

References

1. Cherutich P, Kaiser R, Galbraith J, et al. Lack of knowledge of HIV status a major barrier to HIV prevention, care and treatment efforts in Kenya: results from a nationally representative study. *PLoS One* 2012;7(5):e36797. doi: 10.1371/journal.pone.0036797 [published Online First: 2012/05/11]
2. CDC. Kenya Country Profile: Centers for Disease Control and Prevention; 2022 [Available from: https://www.cdc.gov/globalhivtb/where-we-work/kenya/kenya.html accessed August 4 2022.
3. Ortblad KF, Baeten JM, Cherutich P, et al. The arc of HIV epidemics in sub-Saharan Africa: new challenges with concentrating epidemics in the era of 90-90-90. *Curr Opin HIV AIDS* 2019;14(5):354-65. doi: 10.1097/COH.0000000000000569 [published Online First: 2019/07/26]
4. The National AIDS Control Council 2022 [Available from: https://nacc.or.ke/ accessed July 17 2022.
5. Kenya Ministry of Health. Guidelines on the use of antiretroviral drugs for treating and preventing HIV infection in Kenya - 2018 edition. Nairobi: Kenya Ministry of Health, 2018.
6. Koss CA, Charlebois ED, Ayieko J, et al. Uptake, engagement, and adherence to pre-exposure prophylaxis offered after population HIV testing in rural Kenya and Uganda: 72-week interim analysis of observational data from the SEARCH study. *Lancet HIV* 2020;7(4):e249-e61. doi: 10.1016/S2352-3018(19)30433-3 [published Online First: 2020/02/23]
7. Were D, Musau A, Mutegi J, et al. Using a HIV prevention cascade for identifying missed opportunities in PrEP delivery in Kenya: results from a programmatic surveillance study. *J Int AIDS Soc* 2020;23 Suppl 3:e25537. doi: 10.1002/jia2.25537 [published Online First: 2020/07/01]
8. Mack N, Odhiambo J, Wong CM, et al. Barriers and facilitators to pre-exposure prophylaxis (PrEP) eligibility screening and ongoing HIV testing among target populations in Bondo and Rarieda, Kenya: results of a consultation with community stakeholders. *BMC Health Serv Res* 2014;14:231. doi: 10.1186/1472-6963-14-231 [published Online First: 2014/06/03]
9. Patel RC, Stanford-Moore G, Odoyo J, et al. "Since both of us are using antiretrovirals, we have been supportive to each other": facilitators and barriers of pre-exposure prophylaxis use in heterosexual HIV serodiscordant couples in Kisumu, Kenya. *J Int AIDS Soc* 2016;19(1):21134. doi: 10.7448/IAS.19.1.21134 [published Online First: 2016/12/15]
10. How long will they take it? Oral pre-exposure prophylaxis (PrEP) retention for female sex workers, men who have sex with men and young women in a demonstration project in Kenya. Journal of the International AIDS Society; 2018. JOHN WILEY & SONS LTD THE ATRIUM, SOUTHERN GATE, CHICHESTER PO19 8SQ, W ….
11. Factors influencing initiation, continuation & discontinuation of oral PrEP at selected facilities in South Africa. JOURNAL OF THE INTERNATIONAL AIDS SOCIETY; 2018. JOHN WILEY & SONS LTD THE ATRIUM, SOUTHERN GATE, CHICHESTER PO19 8SQ, W ….
12. Touger R, Wood BR. A Review of Telehealth Innovations for HIV Pre-Exposure Prophylaxis (PrEP). *Curr HIV/AIDS Rep* 2019;16(1):113-19. doi: 10.1007/s11904-019-00430-z [published Online First: 2019/02/01]
13. Kamulegeya LH, Bwanika JM, Musinguzi D, et al. Continuity of health service delivery during the COVID-19 pandemic: the role of digital health technologies in Uganda. *Pan Afr Med J* 2020;35(Suppl 2):43. doi: 10.11604/pamj.supp.2020.35.2.23115 [published Online First: 2021/02/25]
14. Dodoo JE, Al-Samarraie H, Alzahrani AI. Telemedicine use in Sub-Saharan Africa: Barriers and policy recommendations for Covid-19 and beyond. *Int J Med Inform* 2021;151:104467. doi: 10.1016/j.ijmedinf.2021.104467 [published Online First: 2021/04/30]
15. Chen Y, Ronen K, Matemo D, et al. An Interactive Text Messaging Intervention to Improve Adherence to Option B+ Prevention of Mother-to-Child HIV Transmission in Kenya: Cost Analysis. *JMIR Mhealth Uhealth* 2020;8(10):e18351. doi: 10.2196/18351 [published Online First: 2020/10/03]
16. ZALO MA. The regulation of online pharmacies dispensing prescription-only medicines in Kenya: a consumer protection perspective. 2020

For peer review only - http://bmjopen.bmj.com/site/about/guidelines.xhtml

17. Bridges JF. Stated preference methods in health care evaluation: an emerging methodological paradigm in health economics. *Appl Health Econ Health Policy* 2003;2(4):213-24. [published Online First: 2004/05/04]
18. Lancsar E, Louviere J. Conducting discrete choice experiments to inform healthcare decision making: a user's guide. *Pharmacoeconomics* 2008;26(8):661-77. doi: 10.2165/00019053-200826080-00004 [published Online First: 2008/07/16]
19. Eshun-Wilson I, Kim HY, Schwartz S, et al. Exploring Relative Preferences for HIV Service Features Using Discrete Choice Experiments: a Synthetic Review. *Curr HIV/AIDS Rep* 2020;17(5):467-77. doi: 10.1007/s11904-020-00520-3 [published Online First: 2020/08/30]
20. Quaife M, Eakle R, Cabrera Escobar MA, et al. Divergent Preferences for HIV Prevention: A Discrete Choice Experiment for Multipurpose HIV Prevention Products in South Africa. *Med Decis Making* 2018;38(1):120-33. doi: 10.1177/0272989X17729376 [published Online First: 2017/09/03]
21. Zanolini A, Sikombe K, Sikazwe I, et al. Understanding preferences for HIV care and treatment in Zambia: Evidence from a discrete choice experiment among patients who have been lost to follow-up. *PLoS Med* 2018;15(8):e1002636. doi: 10.1371/journal.pmed.1002636 [published Online First: 2018/08/14]
22. Ostermann J, Njau B, Brown DS, et al. Heterogeneous HIV testing preferences in an urban setting in Tanzania: results from a discrete choice experiment. *PLoS One* 2014;9(3):e92100. doi: 10.1371/journal.pone.0092100 [published Online First: 2014/03/20]
23. Wulandari LPL, He SY, Fairley CK, et al. Preferences for pre-exposure prophylaxis for HIV: A systematic review of discrete choice experiments. *EClinicalMedicine* 2022;51:101507. doi: 10.1016/j.eclinm.2022.101507 [published Online First: 2022/07/19]
24. Pereira CCA, Torres TS, Luz PM, et al. Preferences for pre-exposure prophylaxis (PrEP) among men who have sex with men and transgender women at risk of HIV infection: a multicentre protocol for a discrete choice experiment in Brazil. *BMJ Open* 2021;11(9):e049011. doi: 10.1136/bmjopen-2021-049011 [published Online First: 2021/09/29]
25. Quaife M, Eakle R, Cabrera M, et al. Preferences for ARV-based HIV prevention methods among men and women, adolescent girls and female sex workers in Gauteng Province, South Africa: a protocol for a discrete choice experiment. *BMJ Open* 2016;6(6):e010682. doi: 10.1136/bmjopen-2015-010682 [published Online First: 2016/06/30]
26. Ostermann J, Njau B, Hobbie A, et al. Using discrete choice experiments to design interventions for heterogeneous preferences: protocol for a pragmatic randomised controlled trial of a preference-informed, heterogeneity-focused, HIV testing offer for high-risk populations. *BMJ Open* 2020;10(11):e039313. doi: 10.1136/bmjopen-2020-039313 [published Online First: 2020/11/08]
27. Terris-Prestholt F, Mulatu A, Quaife M, et al. Using choice experiments to improve equity in access to socially marketed HIV prevention products. *Journal of choice modelling* 2021;41:100319.
28. Beckham SW, Crossnohere NL, Gross M, et al. Eliciting Preferences for HIV Prevention Technologies: A Systematic Review. *Patient* 2021;14(2):151-74. doi: 10.1007/s40271-020-00486-9 [published Online First: 2020/12/16]
29. MYDAWA. MYDAWA - Most Trusted Online Pharmacy in Kenya [Available from: https://mydawa.com/ accessed July 17 2022.
30. Jhpiego. Fifty Years of Transforming Global Health 2022 [Available from: https://www.jhpiego.org accessed August 4 2022.
31. Mugambi M. Evaluate and Optimize an Online Care Model for PrEP Delivery: Pilot Study (ePrEP Kenya) 2022 [Available from: https://clinicaltrials.gov/ct2/show/NCT05377138.
32. Bridges JF, Hauber AB, Marshall D, et al. Conjoint analysis applications in health--a checklist: a report of the ISPOR Good Research Practices for Conjoint Analysis Task Force. *Value Health* 2011;14(4):403-13. doi: 10.1016/j.jval.2010.11.013 [published Online First: 2011/06/15]
33. Harris PA, Taylor R, Thielke R, et al. Research electronic data capture (REDCap)--a metadata-driven methodology and workflow process for providing translational research informatics support. *J*

For peer review only - http://bmjopen.bmj.com/site/about/guidelines.xhtml

Biomed Inform 2009;42(2):377-81. doi: 10.1016/j.jbi.2008.08.010 [published Online First: 2008/10/22]

34. Sawtooth Software. The CBC System for Choice-Based Conjoint Analysis, Version 9, 2017.
35. Ortblad KF, Mogere P, Roche S, et al. Design of a care pathway for pharmacy-based PrEP delivery in Kenya: results from a collaborative stakeholder consultation. *BMC Health Serv Res* 2020;20(1):1034. doi: 10.1186/s12913-020-05898-9 [published Online First: 2020/11/13]
36. Mugo PM, Micheni M, Shangala J, et al. Uptake and Acceptability of Oral HIV Self-Testing among Community Pharmacy Clients in Kenya: A Feasibility Study. *PLoS One* 2017;12(1):e0170868. doi: 10.1371/journal.pone.0170868 [published Online First: 2017/01/27]
37. Kenya Ministry of Health. Framework for the implementation of pre-exposure prophylaxis of HIV in Kenya. Kenya: Ministry of Health, 2017.
38. Forsythe S, Arthur G, Ngatia G, et al. Assessing the cost and willingness to pay for voluntary HIV counselling and testing in Kenya. *Health Policy Plan* 2002;17(2):187-95. doi: 10.1093/heapol/17.2.187 [published Online First: 2002/05/10]
39. Bobinac A, Van Exel NJ, Rutten FF, et al. Willingness to pay for a quality-adjusted life-year: the individual perspective. *Value Health* 2010;13(8):1046-55. doi: 10.1111/j.1524-4733.2010.00781.x [published Online First: 2010/09/10]
40. Ashburn K, Antelman G, N'Goran MK, et al. Willingness to use HIV self-test kits and willingness to pay among urban antenatal clients in Cote d'Ivoire and Tanzania: a cross-sectional study. *Trop Med Int Health* 2020;25(9):1155-65. doi: 10.1111/tmi.13456 [published Online First: 2020/07/02]
41. Noun Project. Icons and Photos For Everything 2022 [Available from: https://thenounproject.com/.
42. Reed Johnson F, Lancsar E, Marshall D, et al. Constructing experimental designs for discrete-choice experiments: report of the ISPOR Conjoint Analysis Experimental Design Good Research Practices Task Force. *Value Health* 2013;16(1):3-13. doi: 10.1016/j.jval.2012.08.2223 [published Online First: 2013/01/23]
43. Kuhfeld WF, Tobias RD, Garratt M. Efficient Experimental Design with Marketing Research Applications. *Journal of Marketing Research* 1994;31(4):545-57. doi: 10.2307/3151882
44. Valuing environmental amenities using stated choice studies : a common sense approach to theory and practice; 2010.
45. Brazell JD, Diener CG, Karniouchina E, et al. The no-choice option and dual response choice designs. *Marketing Letters* 2006;17(4):255-68. doi: 10.1007/s11002-006-7943-8
46. Determann D, Gyrd-Hansen D, de Wit GA, et al. Designing Unforced Choice Experiments to Inform Health Care Decision Making: Implications of Using Opt-Out, Neither, or Status Quo Alternatives in Discrete Choice Experiments. *Med Decis Making* 2019;39(6):681-92. doi: 10.1177/0272989X19862275 [published Online First: 2019/07/30]
47. Wlömert N, Eggers F. Predicting new service adoption with conjoint analysis: external validity of BDM-based incentive-aligned and dual-response choice designs. *Marketing Letters* 2016;27(1):195-210. doi: 10.1007/s11002-014-9326-x
48. Bahamonde-Birke FJ, Navarro I, Ortúzar JdD. If you choose not to decide, you still have made a choice. *Journal of Choice Modelling* 2017;22:13-23. doi: https://doi.org/10.1016/j.jocm.2016.11.002
49. Orme B. Getting started with conjoint analysis: strategies for product design and pricing research: Research Publishers 2006.
50. Orme B. Sample size issues for conjoint analysis studies: Sawtooth Software 2019.
51. Johnson RO, B. Getting the Most from CBC, 2003.
52. de Bekker-Grob EW, Donkers B, Jonker MF, et al. Sample Size Requirements for Discrete-Choice Experiments in Healthcare: a Practical Guide. *Patient* 2015;8(5):373-84. doi: 10.1007/s40271-015-0118-z [published Online First: 2015/03/03]
53. Louviere JJ, Hensher DA, Swait JD. Stated Choice Methods: Analysis and Applications. Cambridge: Cambridge University Press 2000.

For peer review only - http://bmjopen.bmj.com/site/about/guidelines.xhtml

54. McFadden D. Conditional logit analysis of qualitative choice behavior. 1973
55. Hauber AB, Gonzalez JM, Groothuis-Oudshoorn CG, et al. Statistical Methods for the Analysis of Discrete Choice Experiments: A Report of the ISPOR Conjoint Analysis Good Research Practices Task Force. *Value Health* 2016;19(4):300-15. doi: 10.1016/j.jval.2016.04.004 [published Online First: 2016/06/22]
56. Collins LM, Lanza ST. Latent class and latent transition analysis: With applications in the social, behavioral, and health sciences: John Wiley & Sons 2009.

Figure titles

Figure 1. Study design flowchart
Figure 2. Example of DCE choice task

For peer review only

Figure 1. Study design flow chart

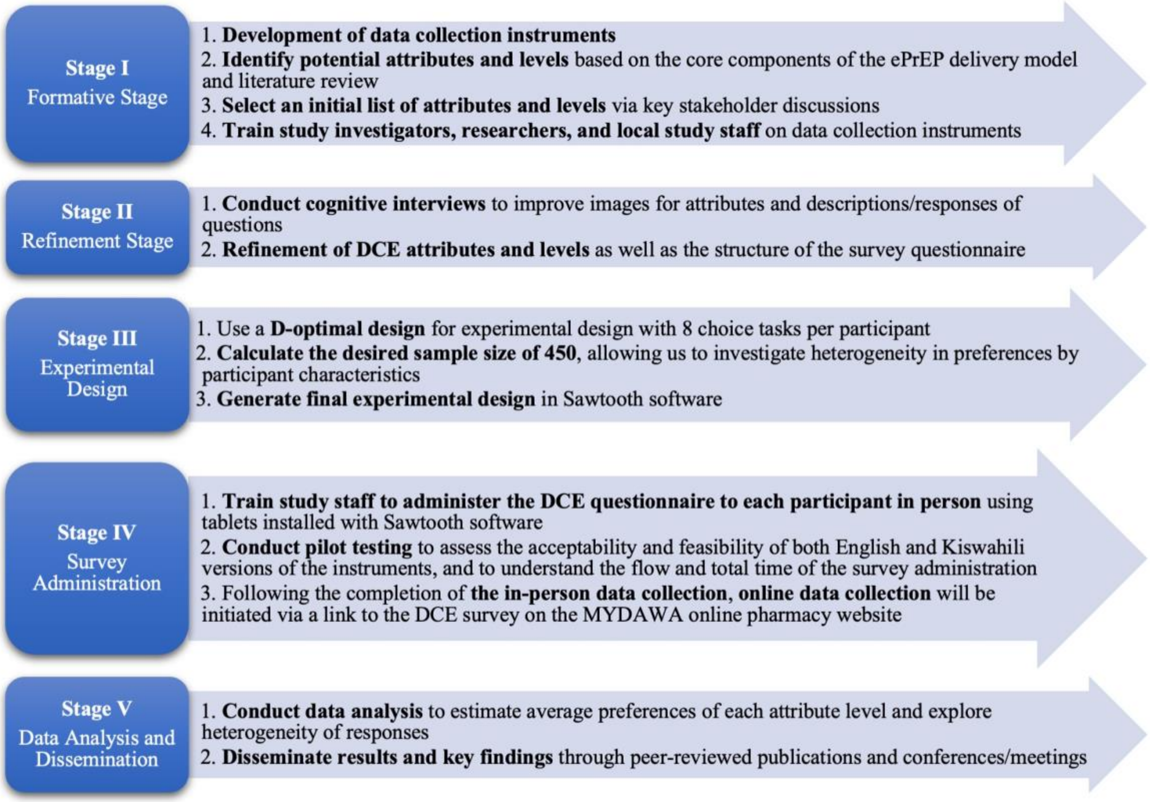

Figure 2. Example of DCE choice task

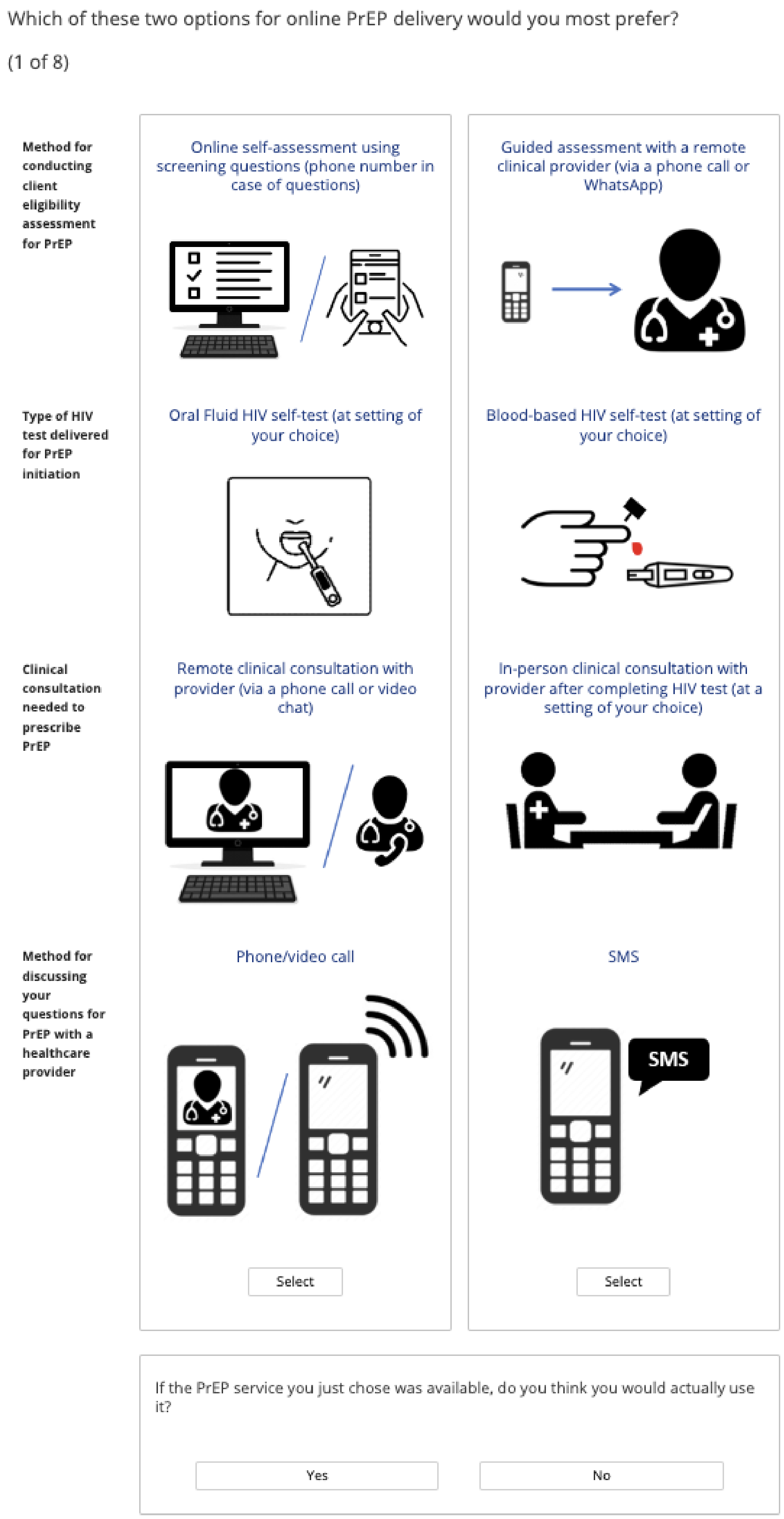

Supplement 1: Cognitive Interview Guide

Online-pharmacy for the delivery of pre-exposure prophylaxis (PrEP) medicines

Discrete Choice Experiment

Cognitive Interview

Introduction

Thank you for taking the time to speak with me. My name is ______. We would like to speak to you about pre-exposure prophylaxis or PrEP. PrEP is a medicine you can take to reduce your risk of getting HIV. We have asked you to participate in this exercise because we are interested in learning how best to delivery PrEP using an online pharmacy. This means a pharmacy will deliver PrEP medication to clients using a courier, so clients do not need to travel to a pharmacy. The aim of this session is to ask you about pictures that are related to PrEP delivery and how they can be improved for our survey instrument. We will also ask you for feedback about other section of our survey to make sure they are clear. This interview should take about 60 minutes. If it's OK with you, I would like to record the interview, so I don't miss any important details in your answers. Feel free to let me know if you need a break at any time. You can also stop the interview if you do not want to continue. Before we begin do you have any questions? Can I start recording?

[Prompt: Start recording]

For peer review only

Part 1. Presentation of Attributes and Levels

Online PrEP delivery includes different services, including an assessment to determine if you are at HIV risk, HIV testing to confirm you are HIV-negative, and talking to a medical provider to make sure PrEP is safe for you.

We will describe the characteristics of obtaining PrEP through online pharmacy in the order you would experience them.

For peer review only

Part 1, a.

PrEP Eligibility Assessment [No need to read the header out loud]

Please tell me how you would interpret these images. [Show the images. Alternative text: What do these images mean to you?]

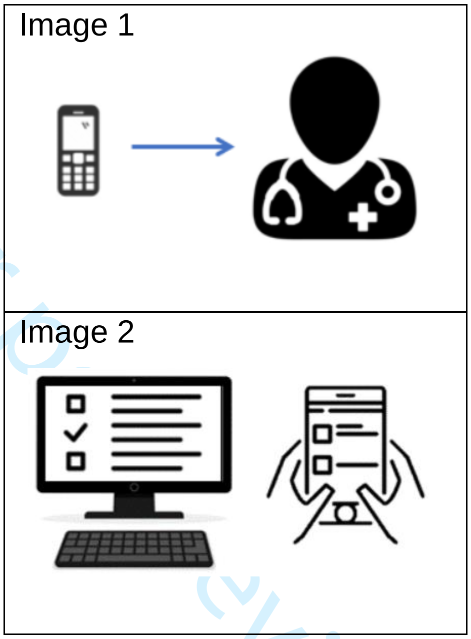

For peer review only

Now I'm going to read you the description and possible ways to have a PrEP Eligibility assessment

PrEP Eligibility Assessment

First, you would complete an **eligibility assessment** to see if PrEP is a good fit for you. This is done by answering questions about your sexual behavior and partners. You can do this in **two ways**: One way is with a provider asking you these questions over the phone or WhatsApp.

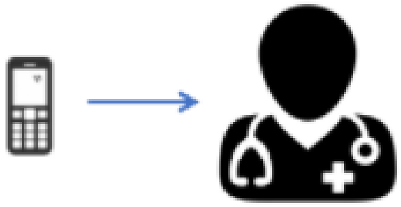

The second way is by answering these questions by yourself on your computer or phone. You will have a phone number to call in case of questions.

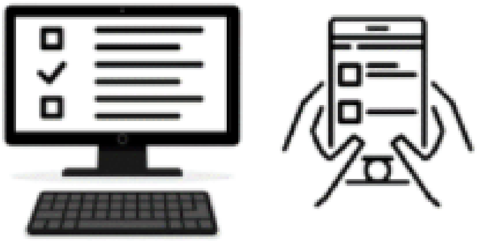

Questions:

- Is the description of "PrEP eligibility Assessment" clear to you? [If necessary, read the description again]
    - Is there any other information we should include to make the description clearer?
- Do the images represent the descriptions well? How could we modify the image to make it clearer?
    - Do you think another person would understand the meaning of this image?
- Are there other ways to assess PrEP eligibility that you think we should include?

Part 1, b.

HIV Test Type [No need to read the header out loud]

Please tell me what you think these images mean. [Show the images]

Image 1

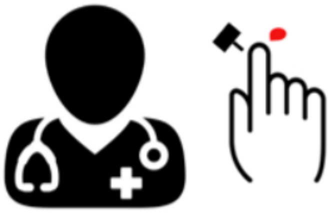

Image 2

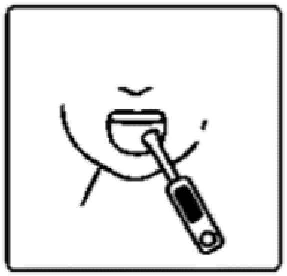

Image 3

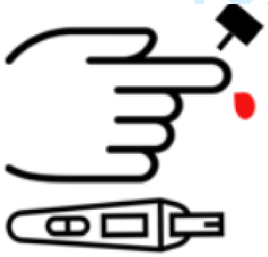

For peer review only

Now I'm going to read you the description and possible ways to have an HIV Test

HIV Test Type

Next you would take an HIV test to make sure you are HIV-negative before starting PrEP. You can test for HIV in one of 3 ways.

One way is to have a healthcare provider meet you at a setting of your choice to conduct HIV test by taking blood from your finger.

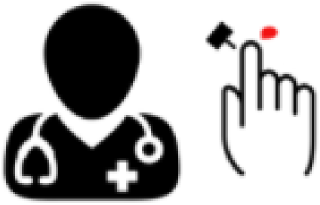

The second way is testing yourself using an HIV self-test kit that uses oral fluid that you collect by swabbing your cheek and gums.

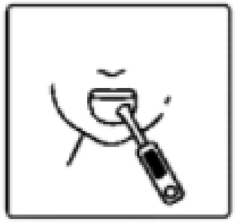

And the third way is testing yourself using a HIV self-test kit that uses blood that you collect from your finger (through a finger prick). Both self-testing kits would be delivered to a setting of your choice and would show a test result after about 20 minutes.

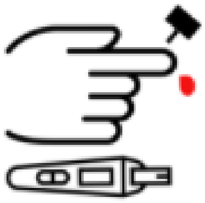

Questions:

- Is the description of "HIV Test Type" clear to you?
    - Is there any other information we should include to make the description clearer?
- Do the images represent the descriptions well? How could we modify the image to make it clearer?
    - Do you think another person would understand the meaning of this image?
- Are there other HIV Test Types that you think we should include?

Is any of the following images a better match for the descriptions I showed you before?

[Repeat the description of the ways to obtain HIV Test if necessary]

New	Original
Image 1<br>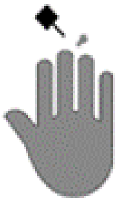<br>[Self-testing]	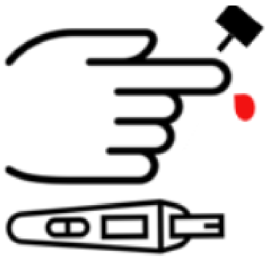
Image 2<br>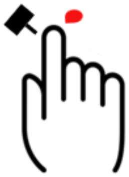<br>[Self-testing]	
Image 3<br>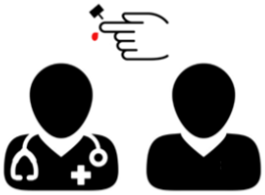<br>[Blood -based testing]	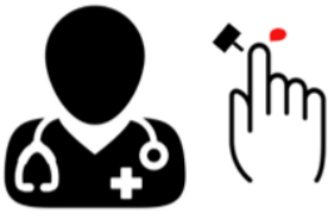
Image 3<br>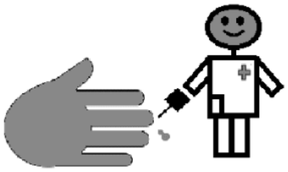<br>[Blood -based testing]	

Part 1, c.

Clinical Consultation [No need to read the header out loud]

Please tell me how you would interpret these images. [Show the images]

Image 1

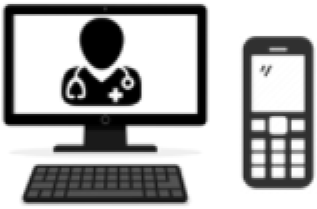

Image 2

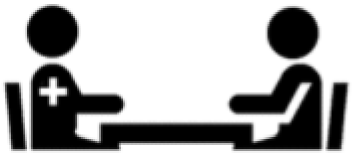

For peer review only

Now I'm going to read you the description and different possible ways to have a Clinical Consultation

Clinical Consultation

You would then have a clinical consultation with a medical provider to make sure PrEP is safe for you to use. This can be done over the phone or a video chat.

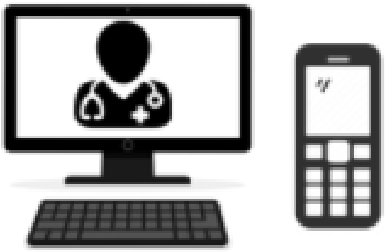

Another option is to see a provider in person at a setting of your choice.

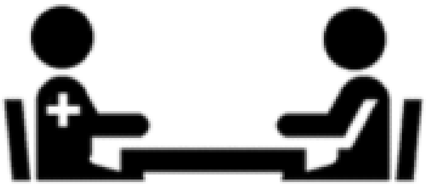

Questions:

- Is the description of "Clinical Consultation" clear to you?
    - Is there any other information we should include to make the description clearer?
- Do the images represent the descriptions well? How could we modify the image to make it clearer?
    - Do you think another person would understand the meaning of this image?
- Are there other ways to provide Clinical consultation that you think we should include?

Is any of the following images a better match for the descriptions I showed you before? [Repeat the description of the ways to provide Clinical Consultation if necessary]

New	Original
Image 1 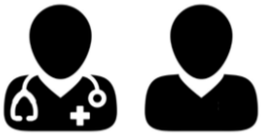 [In-person consultation]	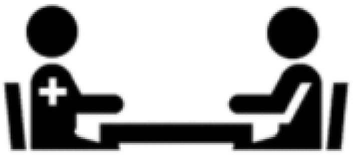
Image 2 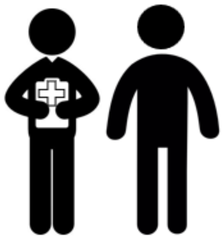 [In-person consultation]	

For peer review only

Part 1, d.

User Support Options [No need to read the header out loud]

Please tell me how you would interpret these images. [Show the images]

Image 1

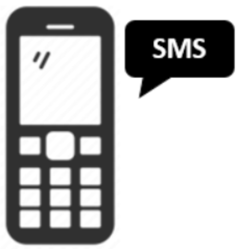

Image 2

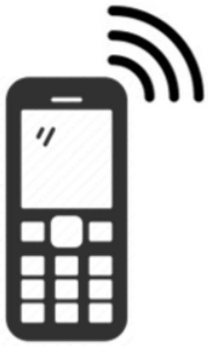

Image 3

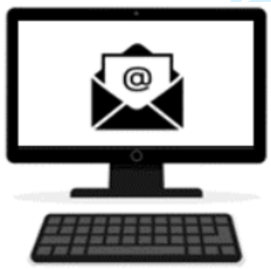

For peer review only

Now I’m going to read you the description and different ways to get User Support

User Support Options

In case you have any questions about PrEP, there are **three different options** for you to discuss your questions with a healthcare provider. The first is using SMS to talk to a provider.

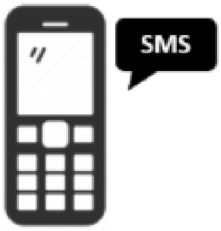

The second option is talking to a provider over the phone, using either voice or video call.

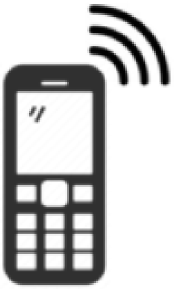

The third is using email to talk to a provider.

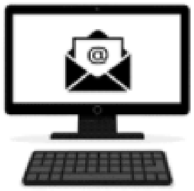

Questions:

- Is the description of “User Support Options” clear to you?
    - Is there any other information we should include to make the description clearer?
- Do the images represent the descriptions well? How could we modify the image to make it clearer?
    - Do you think another person would understand the meaning of this image?
- Are there other ways to provide user support that you think we should include?

only

Is any of the following images a better match for the descriptions I showed you before? [Repeat the description of the ways to provide User Support if necessary]

New	Original
Image 1 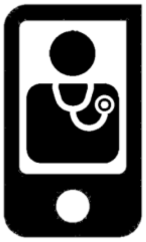 [Video call]	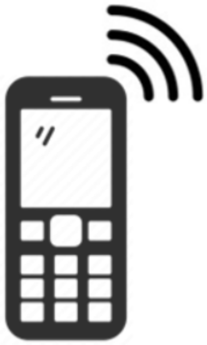
Image 2 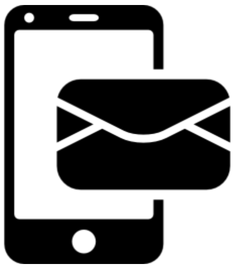 [Email]	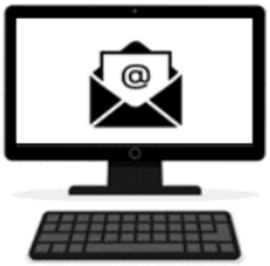

For peer review only

Part 1, e. Summary Table

**Characteristics** *Description*	**Option 1**	**Option 2**	**Option 3**
**PrEP eligibility assessment** *Method for conducting client eligibility assessment for PrEP*	*Online self-assessment* ***on your computer or phone*** *using screening questions (phone number in case of questions)* 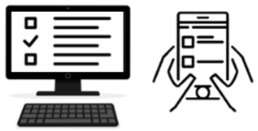	*Guided assessment with a remote clinical provider (via a phone call or WhatsApp)* 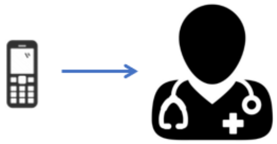	
**HIV test type** *Type of HIV test delivered for PrEP initiation*	*Oral-fluid HIV self-test (at setting of your choice)* 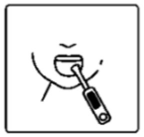	*Blood-based HIV self-test (at setting of your choice)* 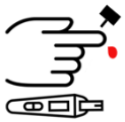	*Provider-administered blood-based rapid diagnostic test (at setting of your choice)* 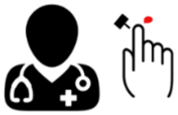
**Clinical consultation for prescribing PrEP** *Clinical consultation needed to prescribe PrEP*	*Remote clinical consultation with provider (via a phone call or video chat)* 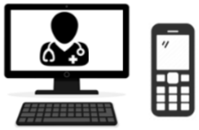	*In-person clinical consultation with provider after completing HIV testing (at a setting of your choice)* 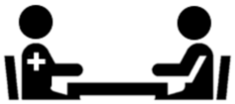	
**User support options for PrEP** *Method for discussing your questions for PrEP with a healthcare provider*	*SMS* 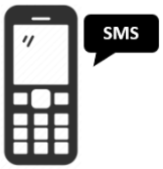	*Phone/video call* 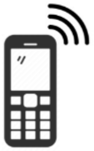	*Email* 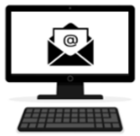

Questions:

We want to make sure we have included all the characteristics of online PrEP delivery that would be important to you

- Are any of the characteristics of PrEP delivery that we showed you today not important or don't add relevant information?
- Is there anything else about an online PrEP delivery service that would be important to you that we did not ask you about?

For peer review only - http://bmjopen.bmj.com/site/about/guidelines.xhtml

Part 2. DCE Choice Task Example

First Take:

I will now ask you about how you would like to get PrEP from an online pharmacy. Please remember there are no wrong answers, we want to know your preferences. When answering each scenario, imagine that you are planning to get PrEP using an online pharmacy.

When making your choice, please consider ONLY the characteristics shown.

For peer review only

Which of these two options for online PrEP delivery would you most prefer?

	A	B
**Method for conducting client eligibility assessment for PrEP**	Guided assessment with a remote clinical provider (via a phone call or WhatsApp) 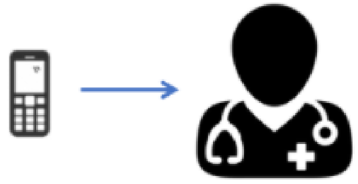	Online self-assessment using screening questions (phone number in case of questions) 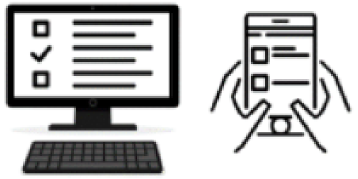
**Type of HIV test delivered for PrEP initiation**	Healthcare provider administers HIV Test at setting of your choice (blood-based) 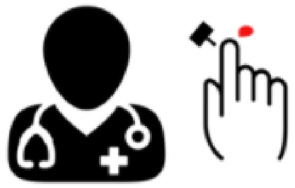	Oral Fluid HIV self-test (at setting of your choice) 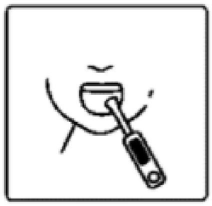
**Clinical consultation needed to prescribe PrEP**	In-person clinical consultation with provider after completing HIV test (at a setting of your choice) 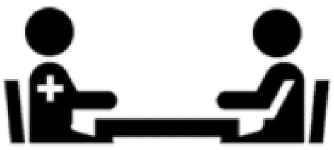	Remote clinical consultation with provider (via a phone call or video chat) 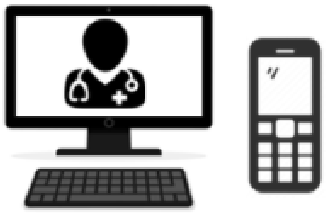
**Method for discussing your questions for PrEP with a healthcare provider**	Phone/video call 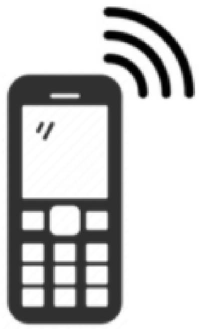	Email 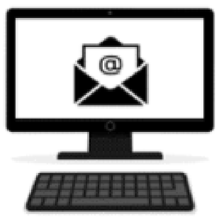

Questions:

- How hard did you find this exercise?
- How could we modify the exercise to make it easier?
- Do you think another person would understand this exercise?
- Is there anything in the question for the exercise that we should modify to make it clearer? [If necessary, read the question again "Which of these two options for online PrEP delivery would you most prefer?"]
- When I said, "When making your choice, please consider ONLY the characteristics I presented before.", what does that mean to you? [The objective of that sentence is to ignore any other characteristics of online pharmacy (e.g., the brand of the drug or the company providing the service) that are not included in the table. Check if the participant understood that.]
- Can you tell me how you made your decision? [Check if the participant is paying attention to all characteristics, if he doesn't mention one, ask about it. For example: I noticed you didn't mention "Clinical consultation", did you consider the option for that characteristic?"]

For peer review only

Second take:

I will now ask you about how you would like to get PrEP from an online pharmacy. Please remember there are no wrong answers, we want to know your preferences. When answering each scenario, imagine that you are planning to get PrEP using an online pharmacy. I will also ask you if you would choose to get PrEP using the option you selected. If you would not choose to get PrEP if only these two options were available, you can answer no to this question.

When making your choice, please consider ONLY the characteristics shown

For peer review only

Which of these two options for online PrEP delivery would you most prefer?

	A	B
**Method for conducting client eligibility assessment for PrEP**	Guided assessment with a remote clinical provider (via a phone call or WhatsApp) 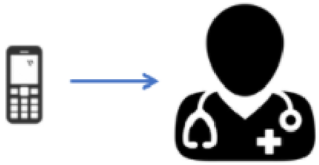	Online self-assessment using screening questions (phone number in case of questions) 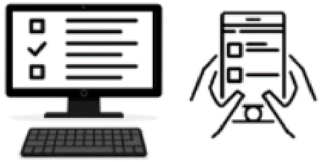
**Type of HIV test delivered for PrEP initiation**	Healthcare provider administers HIV Test at setting of your choice (blood-based) 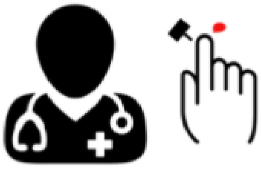	Oral Fluid HIV self-test (at setting of your choice) 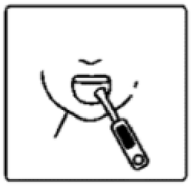
**Clinical consultation needed to prescribe PrEP**	In-person clinical consultation with provider after completing HIV test (at a setting of your choice) 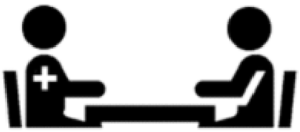	Remote clinical consultation with provider (via a phone call or video chat) 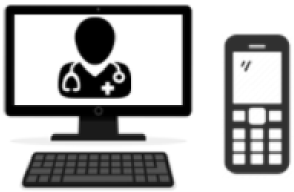
**Method for discussing your questions for PrEP with a healthcare provider**	Phone/video call 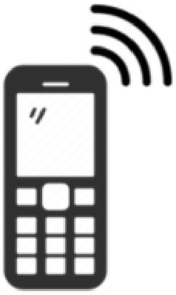	Email 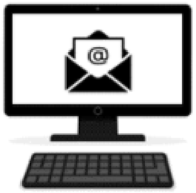

If the PrEP service you just chose was available, do you think you would actually use it?

Yes | No

Questions:

- How different was this time choosing an alternative compared to the first time? [Now the participant has the option to say if they would choose to get PrEP based on their previous selection or not]
- Was does the question "Would you choose to get PrEP using this service if it were available?" mean in your own words? What do you think is asking from you?
  - Our goal in asking this question is to understand if you would really choose to get this service if it were available to you. Do you think the question is clearly asking that?
  - If not, how can we make this question clearer? [If participant said they don't know, that's fine, move to the next section]
- How many of these tasks do you think you could answer before getting tired and/or bored?

For peer review only

Part c. Willingness to Pay Questions

The next set of questions will ask about how much you would like to pay for different parts of online PrEP delivery. For each part, please provide the lowest price, the maximum price and the ideal price you are willing to pay. For the lowest price, please tell us the lowest amount you think the service should cost because otherwise you would be concerned about its quality, and you would not purchase the service. In answering these questions, please consider your usual expenses. Remember that there are no right or wrong answers. We're interested in your preference. You can write zero if you feel that most accurately reflects your preference.

For a blood-based HIV self-test, delivered via a courier to a location of your choice (one-time cost):

What is the lowest price you are willing to pay? ______ KSH

What is the highest price you are willing to pay? ______ KSH

What price do you consider ideal? ______ KSH

For an oral HIV self-test, delivered via a courier to a location of your choice (one-time cost):

What is the lowest price you are willing to pay? ______ KSH

What is the highest price you are willing to pay? ______ KSH

What price do you consider ideal? ______ KSH

For remote/online clinical consultation to obtain a prescription for PrEP based on your HIV self-test results (one-time cost):

What is the lowest price you are willing to pay? ______ KSH

What is the highest price you are willing to pay? ______ KSH

What price do you consider ideal? ______ KSH

For delivery of PrEP medicines (delivery fee plus 1 month supply of medicines):

What is the lowest price you are willing to pay? ______ KSH

What is the highest price you are willing to pay? ______ KSH

What price do you consider ideal? ______ KSH

Questions:

- Overall, is it clear what the objective of the questions is? Would you mind explaining it to me in your own words?
- Please select one of the questions and give your answers. Would you explain me how did you select the amounts?
  [Pay attention to the order in which the participant finds the amounts. Which one comes first, the ideal, the lowest, or the highest?]
  - In this process, did you consider your usual expenses on medicines or healthcare?
  - What other expenses did you consider, if any?
  - Did you consider the cost options presented in the previous section?
- Is the instruction “For the lowest price, please tell us the lowest amount you think the service should cost because otherwise you would be concerned about its quality, and you would not purchase the service.” clear enough? Would you tell me in your own words what does it mean?
  - Would you be concerned about the quality of a product that is free? Why or why not?
- Do you think that all questions are unique or some of them ask for the same information as others?

End of the interview

Do you have any questions or final thoughts you would like to share?

Thank you very much for your time and dedication in giving me your answers, I’m sure your information will help us create a better survey.

[Stop the recording]

For peer review only

Supplement 2: Training Materials

EPharmacy PrEP: Pilot Study Discrete Choice Experiment Training

24th-25th January 2022

Introductions

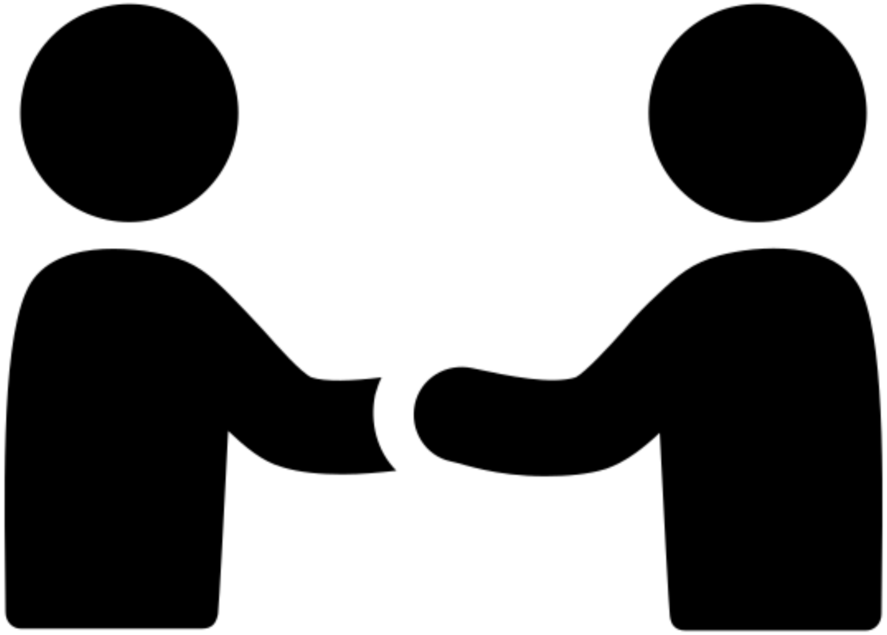

PrEP Rx

Overview of this session

- PrEP
- PrEP delivery strategies
- ePharmacy PrEP delivery
- Discrete Choice Experiments

What is PrEP?

- PrEP is safe in pregnancy and for breastfeeding mothers
- It takes approximately **SEVEN** days after starting treatment for PrEP to be optimally effective.
- With poor adherence effectiveness becomes lower

For peer review only - http://bmjopen.bmj.com/site/about/guidelines.xhtml

Recommended medication for PrEP use

PREFERRED	ALTERNATIVE
Tenofovir (TDF) + Emtricitabine (FTC)<br>300mg 200mg	Tenofovir (TDF) 300 mg
	Tenofovir (TDF) + Lamivudine (3TC)<br>300mg 300mg

Does PrEP have side effects?

PrEP side effects

No major safety issues have been observed with use of PrEP.

About 1 in 10 people who use PrEP experience **minor**, **temporary** side effects, such as:

- Headache
- Weight loss
- Nausea
- Vomiting
- Abdominal discomfort

If side effects occur, they typically **start in the first few days or weeks** of PrEP use and **last a few days**—almost always less than 1 month.

References: AVAC (Nov. 2020); Tetteh et al. (2017)

What PrEP does and doesn’t prevent

PrEP reduces risk of getting HIV by at least 90%

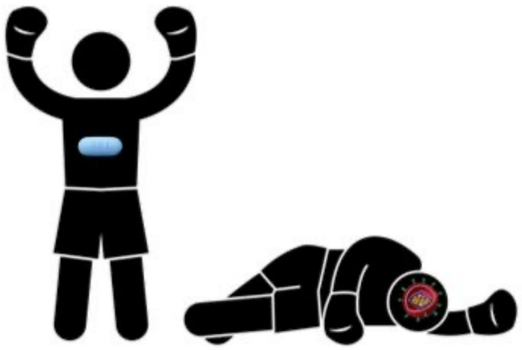

PrEP does **not** prevent sexually transmitted infections (STIs) or pregnancy.

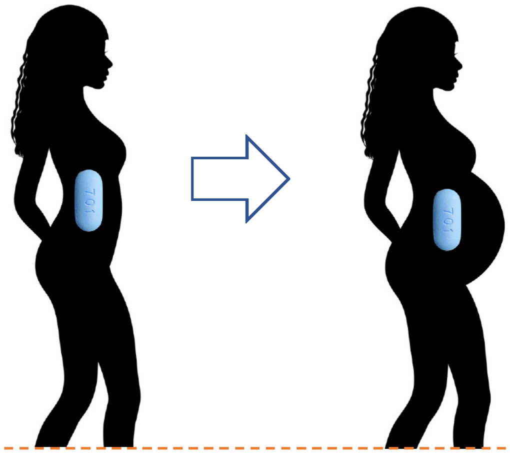

Prospective PrEP users should be advised that if they wish to protect themselves against STIs and/or pregnancy, they should **use PrEP in combination with other methods**, like condoms and contraception.

Aside from protecting against HIV, does PrEP have any other benefits?

Other PrEP benefits

Additional benefits of PrEP:

- Decreased anxiety or fear of contracting HIV
- Increased sense of empowerment and control over HIV
- Increased sense of pleasure and intimacy during sex
- Increased communication and trust with sex partners

Benefits specific to serodiscordant couples:

- Increased ability to cope with and accept serodiscordance
  (e.g., sense of solidarity taking daily medication)
- Ability to safely conceive a child

Sources: Gilmore et al. 2014; Ware et al. 2012; Ware et al. 2014

PrEP eligibility criteria

Per Kenya national guidelines

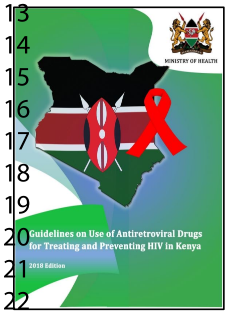

- Age 16+
- Confirmed HIV-negative
- Substantial, ongoing risk of HIV infection
- No signs of acute HIV infection in past month
- No contraindications to PrEP medications
- Willing to use PrEP as prescribed

*Specifically, HIV vaccine or HIV prevention studies

Core components

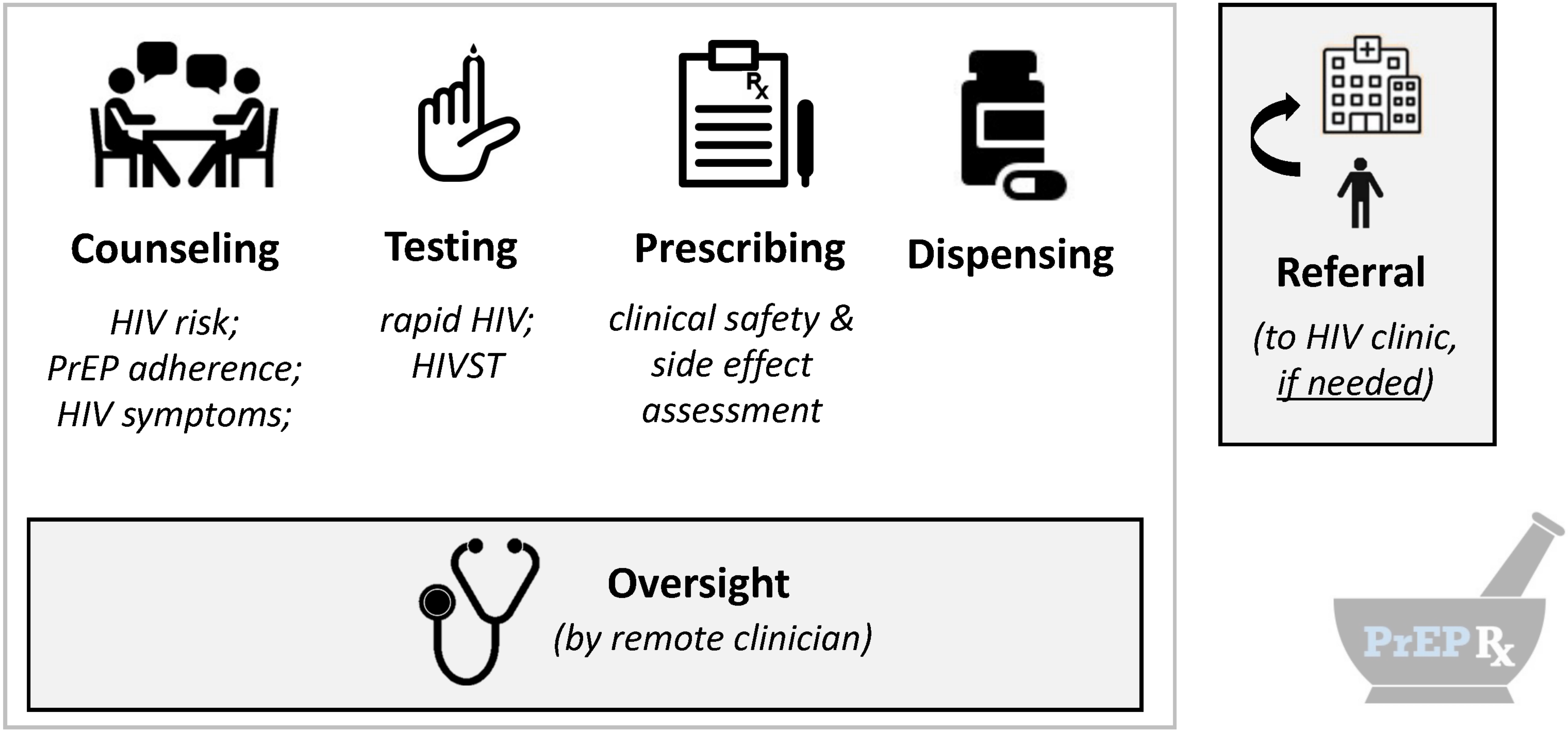

PrEP use over time using retail pharmacies: Pilot study

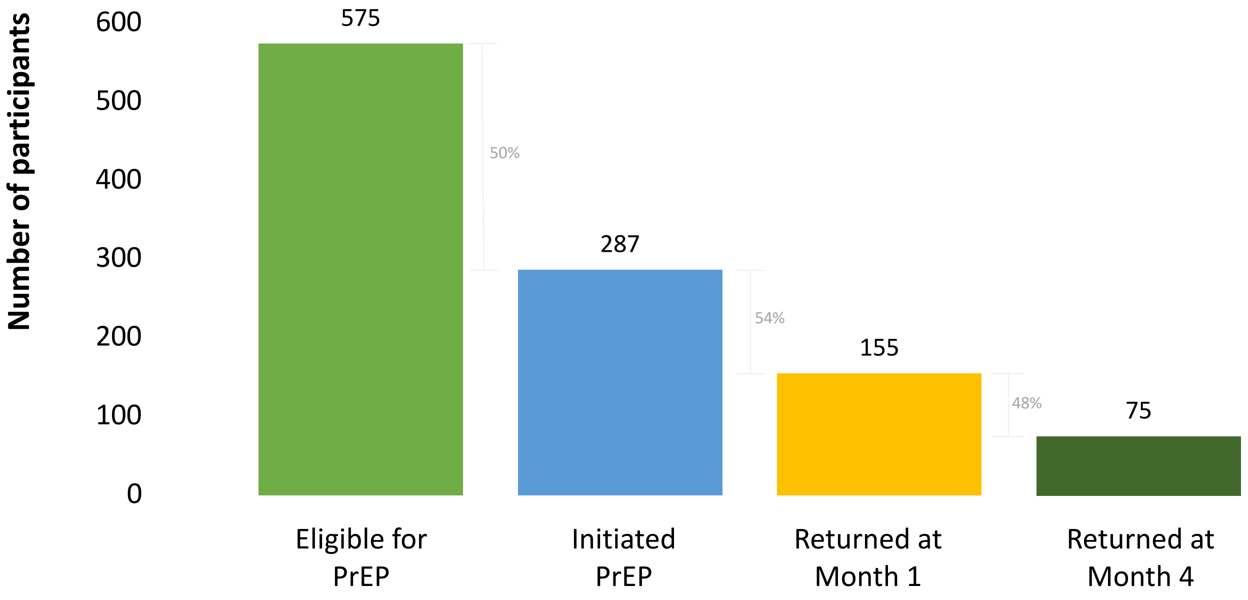

Challenges with delivering PrEP at retail pharmacies

1 PrEP services too **expensive** for some clients.

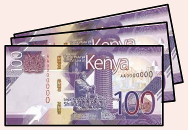

2 HIV risk assessment **time-consuming** & some clients **uncomfortable** doing it out loud.

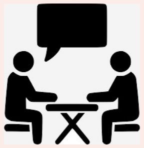

3 Some prospective clients **uncomfortable** testing for HIV at pharmacy, especially if unsure of status.

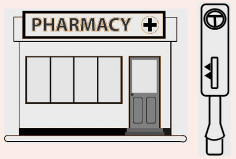

Can PrEP be delivered through E-pharmacies?

Possible benefits

- Convenient: Clients can get PrEP delivered to their home without having to go to a clinic or pharmacy
- Greater confidentiality (decreased stigma)

For peer review only - http://bmjopen.bmj.com/site/about/guidelines.xhtml

Steps for getting PrEP from E-pharmacy

1) HIV risk screening
2) HIV testing
3) Clinical consultation (medical assessment)
4) PrEP drugs delivered via courier
5) Ongoing user support (clients can talk to a provider to ask questions about their PrEP)

For peer review only - http://bmjopen.bmj.com/site/about/guidelines.xhtml

Step 1: HIV Risk assessment

Assessing HIV risk

Example questions from the RAST

In the past 6 months, have you had sex without a condom with anyone?<br><br>*Select "yes" even if this only happened once in the past 6 months.*	☐ Yes	☐ No	☐ Unsure

Assessing HIV risk

Example questions from the RAST

In the past 6 months, have you had sex without a condom with anyone?<br><br>*Select "yes" even if this only happened once in the past 6 months.*	☐ Yes	☐ No	☐ Unsure
In the past 6 months, have you been diagnosed with or treated for a sexually transmitted infection (STI)?<br><br>*Examples of sexually transmitted infections include chlamydia and gonorrhea.*	☐ Yes	☐ No	☐ Unsure

Assessing HIV risk

Example questions from the RAST

In the past 6 months, have you had sex without a condom with anyone?<br><br>*Select "yes" even if this only happened once in the past 6 months.*	☐ Yes	☐ No	☐ Unsure
In the past 6 months, have you been diagnosed with or treated for a sexually transmitted infection (STI)?<br><br>*Examples of sexually transmitted infections include chlamydia and gonorrhea.*	☐ Yes	☐ No	☐ Unsure
Do you have any sex partners who are HIV-positive and:<br>• are not currently taking antiretroviral therapy (ART),<br>• have been taking antiretroviral therapy (ART) for less than 6 months,<br>• have been missing doses of their antiretroviral therapy (ART),<br>• have a detectable HIV viral load, or<br>• the two of you are trying to get pregnant?<br><br>*Select "yes" if any of the above statements are true about any of your sex partners who are HIV-positive.*	☐ Yes	☐ No	☐ Unsure

Assessing HIV risk

Example questions from the RAST

In the past 6 months, have you had sex without a condom with anyone?<br><br>*Select "yes" even if this only happened once in the past 6 months.*	☐ Yes	☐ No	☐ Unsure
In the past 6 months, have you been diagnosed with or treated for a sexually transmitted infection (STI)?<br><br>*Examples of sexually transmitted infections include chlamydia and gonorrhea.*	☐ Yes	☐ No	☐ Unsure
Do you have any sex partners who are HIV-positive and:<br>• are not currently taking antiretroviral therapy (ART),<br>• have been taking antiretroviral therapy (ART) for less than 6 months,<br>• have been missing doses of their antiretroviral therapy (ART),<br>• have a detectable HIV viral load, or<br>• the two of you are trying to get pregnant?<br><br>*Select "yes" if any of the above statements are true about any of your sex partners who are HIV-positive.*	☐ Yes	☐ No	☐ Unsure

If client answers "yes" to any RAST question, s/he is considered to have substantial ongoing risk of HIV.

Step 2: HIV testing

Importance of HIV testing

- PrEP is for HIV **prevention**, not treatment.
- Therefore, PrEP is only for **HIV-negative** individuals.
    - HIV-*positive* individuals should take ART, not PrEP.
    - Starting an HIV-*positive* individual on PrEP could make their HIV virus harder to treat.

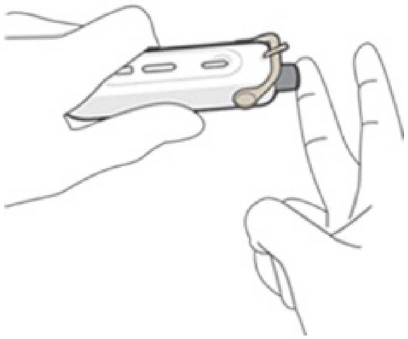

**So before starting someone on PrEP,
it is critical to confirm that they are HIV-negative.**

**PrEP users must also get ongoing
periodic HIV testing to confirm that they are still HIV-negative (usually every 1-3 months.**

HIV testing schedule

Kenya national PrEP guidelines

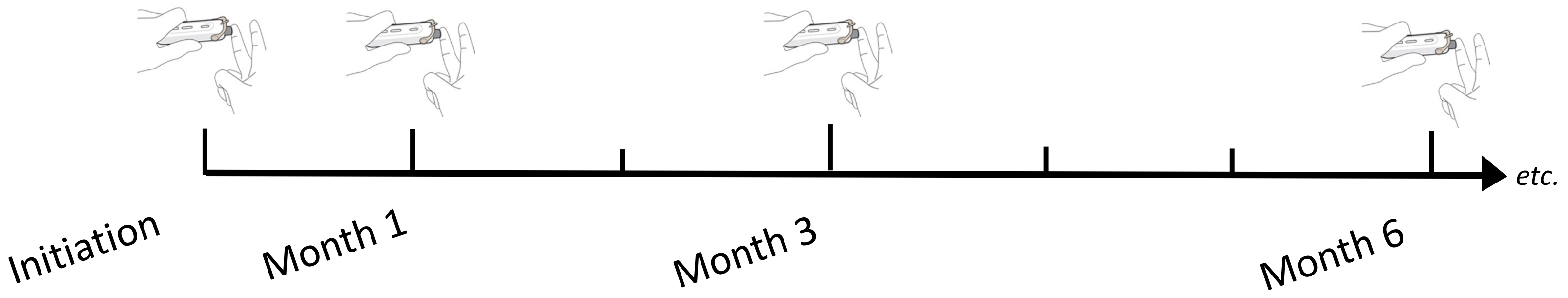

What is HIVST?

A process whereby an individual collects his or her own specimen (oral fluid or blood), performs an HIV rapid diagnostic test and interprets the result, often in a private setting, either alone or with someone she trusts.

Benefits:

- Promotes access to HIV testing services
- Increases autonomy
- Assures confidentiality/privacy
- Empowers
- Convenient

For peer review only - http://bmjopen.bmj.com/site/about/guidelines.xhtml

Step 3: Clinical consultation

Medical safety assessment

Signs & symptoms of acute HIV infection?	☐ Yes	☐ No
Current or past liver disease?	☐ Yes	☐ No
Current or past kidney disease?	☐ Yes	☐ No
Diabetic?	☐ Yes	☐ No

Medical safety assessment

Signs & symptoms of acute HIV infection?	☐ Yes	☐ No

Provider asks the client if they have had any high-risk sexual contact **in past month** and subsequently experienced any of the following:

- Fever
- Sore throat
- Muscle or joint pains
- Swollen glands
- Diarrhoea
- Headache

If client answers "yes", provider does not prescribe PrEP. Refers client to clinic.

Medical safety assessment

Current or past liver disease?	☐ Yes	☐ No

Asks if client has ever had problems with their liver, such as hepatitis, cancer, or cirrhosis.

If client answers "yes", provider does not prescribe PrEP. Refers client to clinic.

Medical safety assessment

Current or past kidney disease?	☐ Yes	☐ No

Asks if client has ever had problems with their kidneys, such as acute or chronic kidney injury or kidney failure.

If client answers "yes", provider does not prescribe PrEP. Refers client to clinic.

Medical safety assessment

Diabetic?	☐ Yes	☐ No

If client is diabetic, provider does not start PrEP.

- Diabetes is a risk factor for kidney disease. So diabetic clients must be evaluated for kidney disease before starting PrEP.

Provider refers client to clinic

Step 4: PrEP delivery via courier

PrEP delivery

- Delivered by courier to location of client’s choice (e.g. home or other location)
- Discreet packaging

Step 5: Ongoing user support

User support

- Client can ask a provider questions about PrEP use, side effects, etc
- Options for user support
  - Phone calls
  - WhatsApp/SMS
  - Email

Pilot study of E-pharmacy PrEP delivery

Online model: pilot study

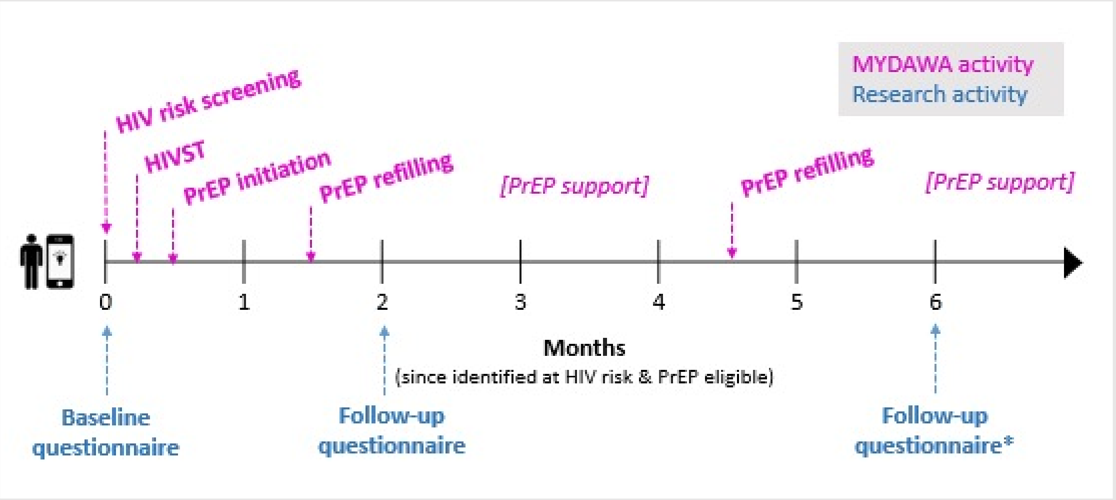

**Client characteristics**	**Demographics**	Month: 0
	**HIV risk profile**	Months: 0 & 2
	**History of PrEP use**	Month: 0
	**Contraceptive use**	Month: 0
**Effectiveness outcomes**	**PrEP initiation** *(primary)*	Month: 2
	**PrEP continuation**	Months: 2 & 6
**Process outcomes**	**Awareness of online PrEP**	Month: 0
	**Modality of HIV risk screening**	Month: 0
	**HIVST uptake**	Month: 2
	**Chatbot/clinical officer use**	N/A
	**HIVST/PrEP delivery setting**	N/A
	**Selection of support reminders**	N/A

What are client preferences for E-Pharmacy PrEP delivery?

- Risk assessment
- Clinical consultation
- Type of HIV test
- User support
- Cost

Understanding client preferences can help increase PrEP coverage among those at risk of HIV

For peer review only - http://bmjopen.bmj.com/site/about/guidelines.xhtml

Discrete Choice Experiments (DCEs)

- Quantitative method used to understand participants preferences for a service without directly asking them to state their options.
- Participants given a series of alternative hypothetical scenarios with variables or “attributes”, each with variations or “levels” and asked to choose their preferred scenario.
- Surveys usually contain 5-10 scenarios.
- Increasingly used in healthcare settings
- Especially useful for new interventions that are not currently available

Why use a DCE instead of a qualitative interview?

- Qualitative interviews to understand preferences for straightforward decisions but DCEs are useful for more complex interventions. DCEs provide quantitative estimate of preferences, estimate trade-offs between preferences

For peer review only - http://bmjopen.bmj.com/site/about/guidelines.xhtml

Analysis

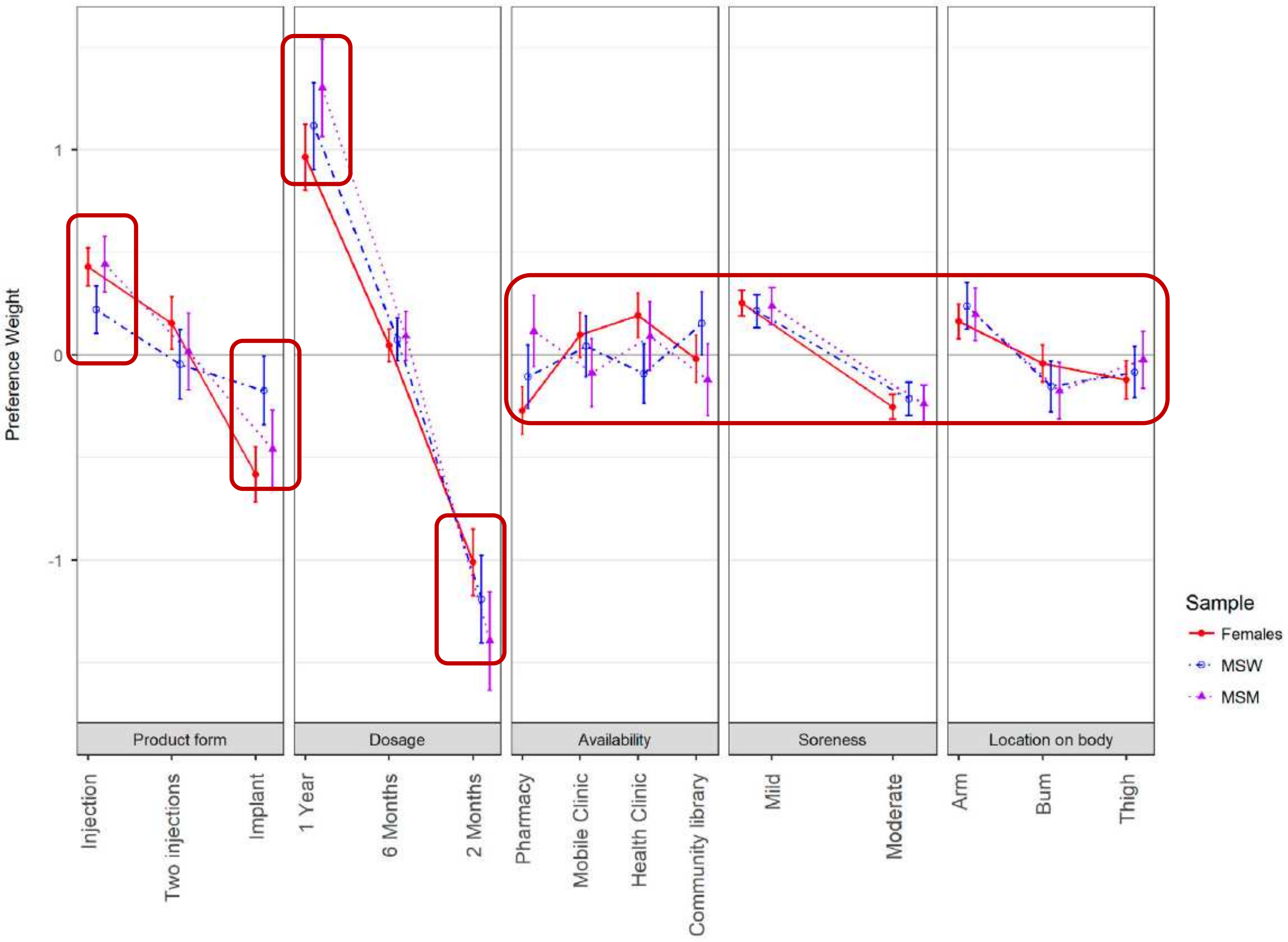

- Can quantify preferences to determine most important characteristics
- Can stratify by participant type

Using DCEs to quantify trade-offs

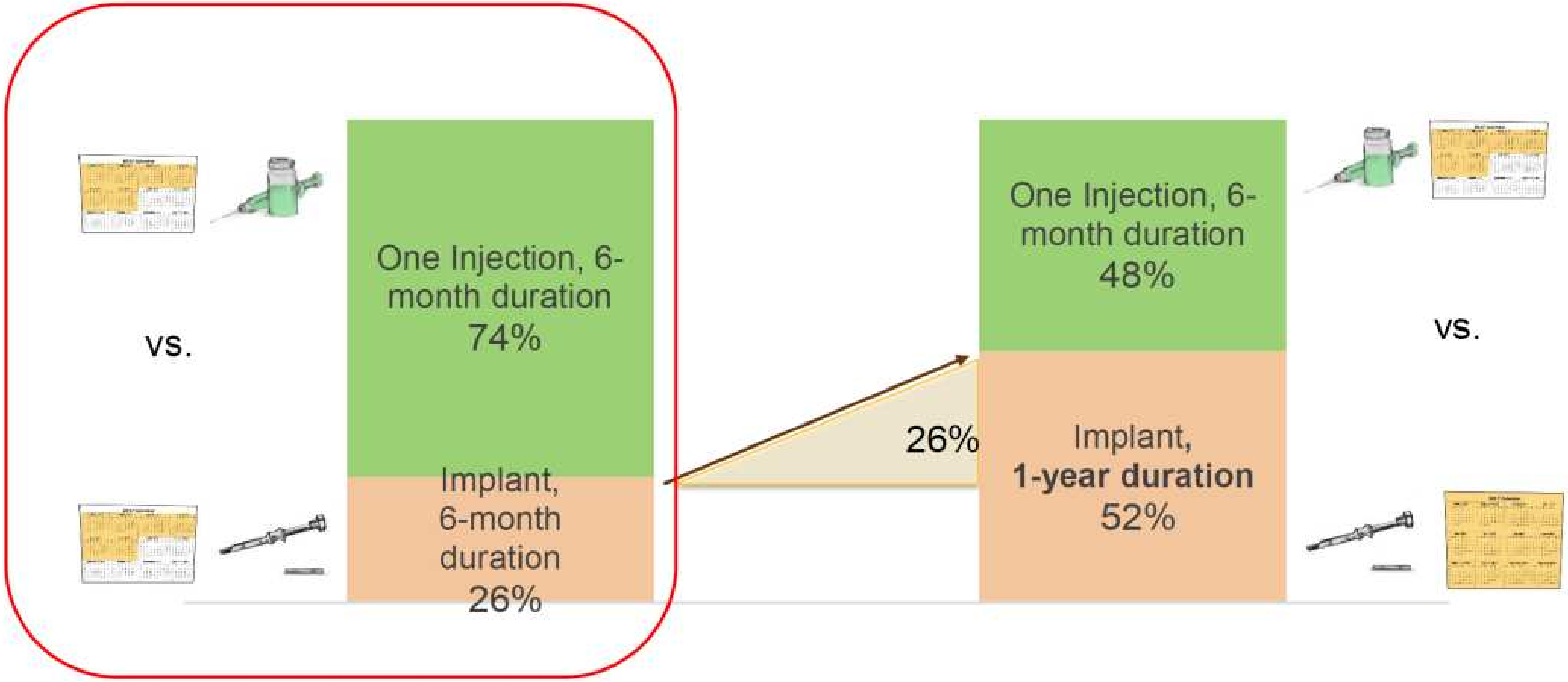

Steps for Conducting a DCE

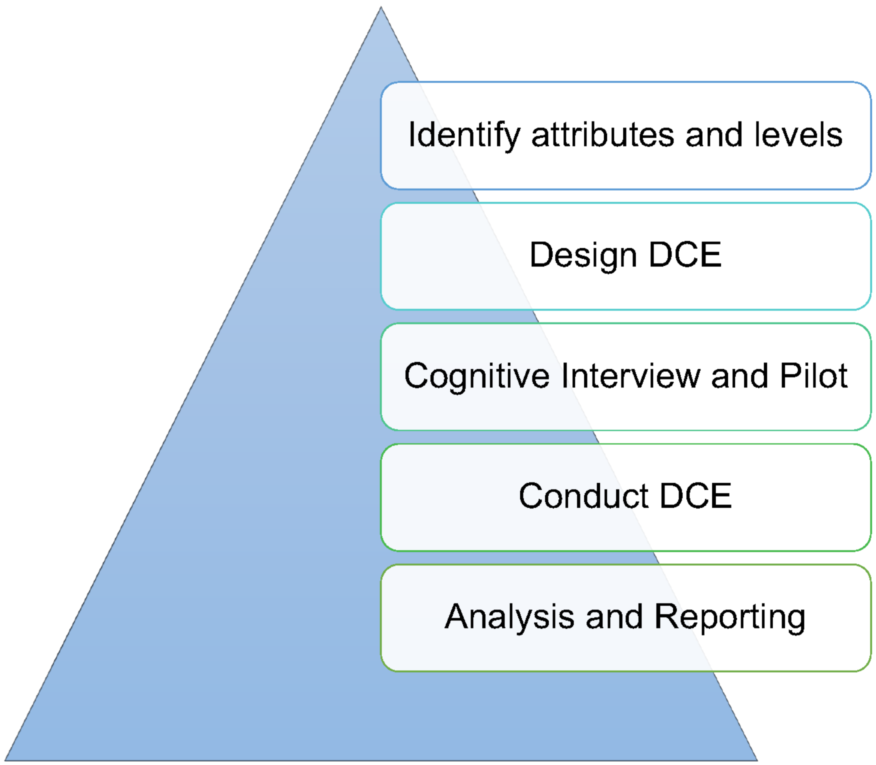

Attributes and Levels of ePharm DCE

Attribute	Description	Levels
**PrEP eligibility assessment**	Method for conducting client eligibility assessment for PrEP	• Online self-assessment using screening questions (phone number in case of questions)<br>• Guided assessment with a remote clinical provider (via a phone call or WhatsApp)
**HIV test type**	Type of HIV test delivered for PrEP initiation	• Oral Fluid HIV self-test (at setting of your choice)<br>• Blood-based HIV self-test (at setting of your choice)<br>• Healthcare provider administers HIV Test at setting of your choice (blood-based)
**Clinical consultation for prescribing PrEP**	Clinical consultation needed to prescribe PrEP	• Remote clinical consultation with provider (via a phone call or video chat)<br>• In-person clinical consultation with provider after completing HIV test (at a setting of your choice)
**User support options for PrEP**	Method for discussing your questions for PrEP with a healthcare provider	• SMS<br>• Phone/video call<br>• Email
**Cost of PrEP delivery**	Total cost of the PrEP delivery visit (including HIV self-test delivery and support, the remote clinician consultation, and PrEP delivery (one month of PrEP)	• 500 KES<br>• 2000 KES<br>• 4000 KES

Virtual PrEP delivery model

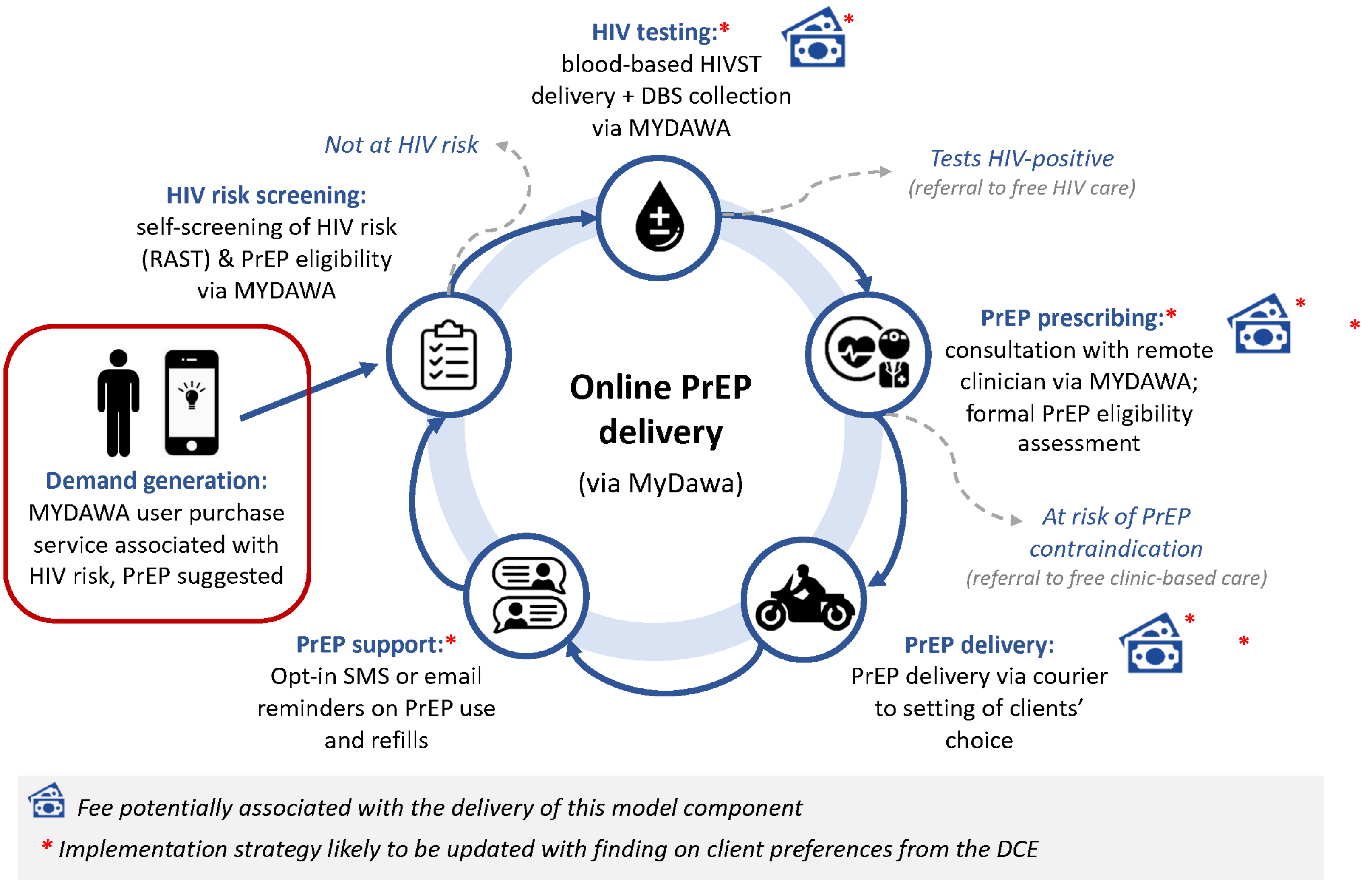

K. Ortblad 2021 ©

EPharmacy PrEP: Pilot Study Discrete Choice Experiment Training

Day 1 Session 2

24th-25th January 2022

Overview of study procedures

- Recruitment
- Pre-screening
- Consent
- Questionnaire administration (in-person)

For peer review only - http://bmjopen.bmj.com/site/about/guidelines.xhtml

Recruitment

- Target MYDAWA clients accessing products related to HIV/sexual health
- Banner ads on MYDAWA website, flyers included with product deliveries
- Potential participants will call study phone number or SMS/WhatsApp with the word #GenN if interested

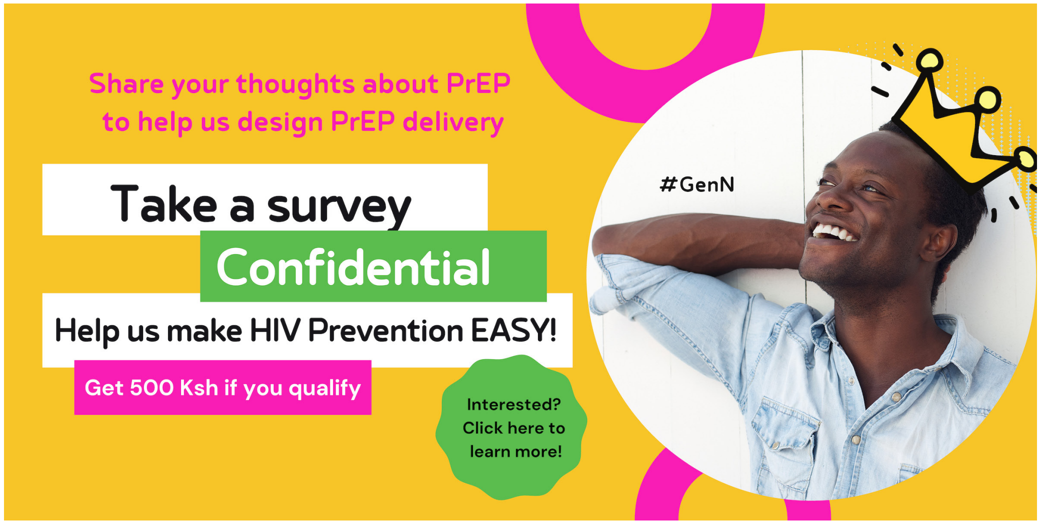

Pre-screening

- Pre-screening will take place by phone
- Study staff will complete REDCap pre-screening data entry form
    1. Eligibility assessment
    2. Schedule in-person visit

For peer review only - http://bmjopen.bmj.com/site/about/guidelines.xhtml

Eligibility criteria

- Participants are eligible to enroll in the study if they are:
  1. Age 18 or older
  2. HIV-negative or unknown HIV status
  3. Respond ‘yes’ to at least one of eleven screening questions about HIV risk
  4. Able and willing to provide informed consent

Eligibility Assessment

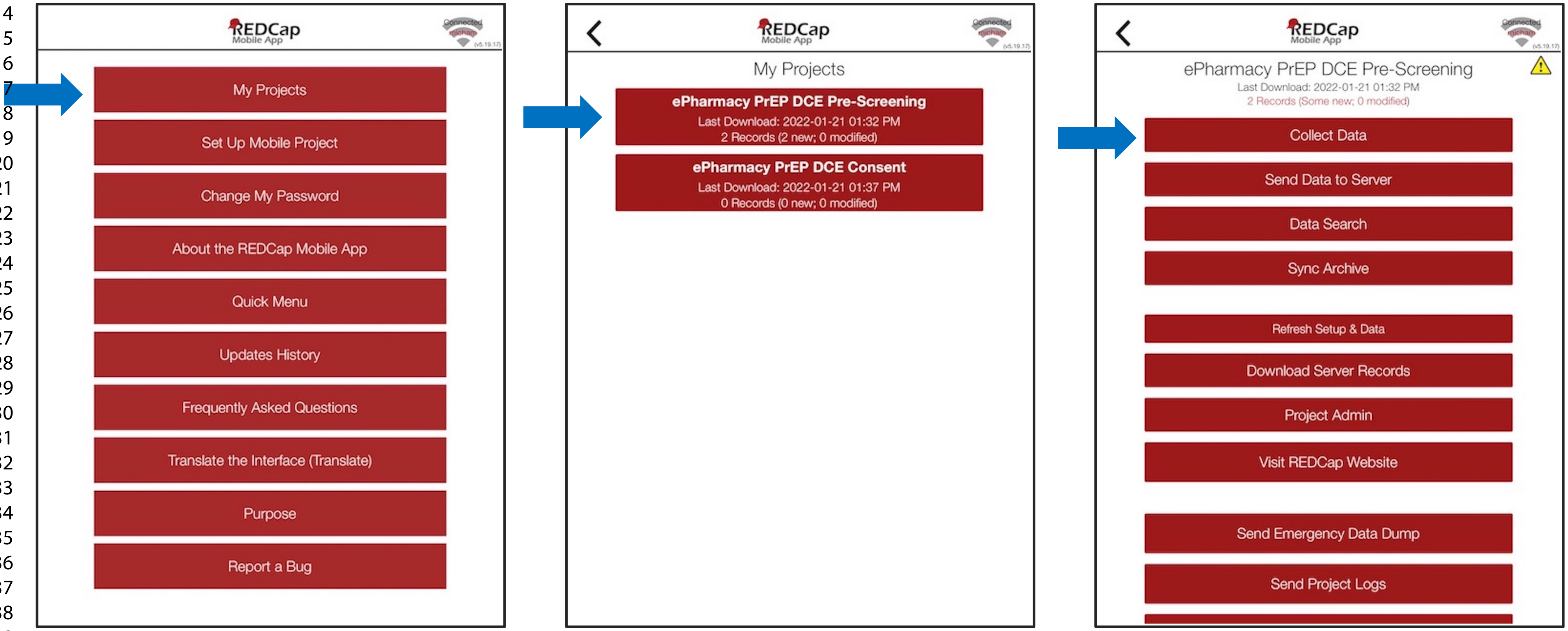

Eligibility Assessment

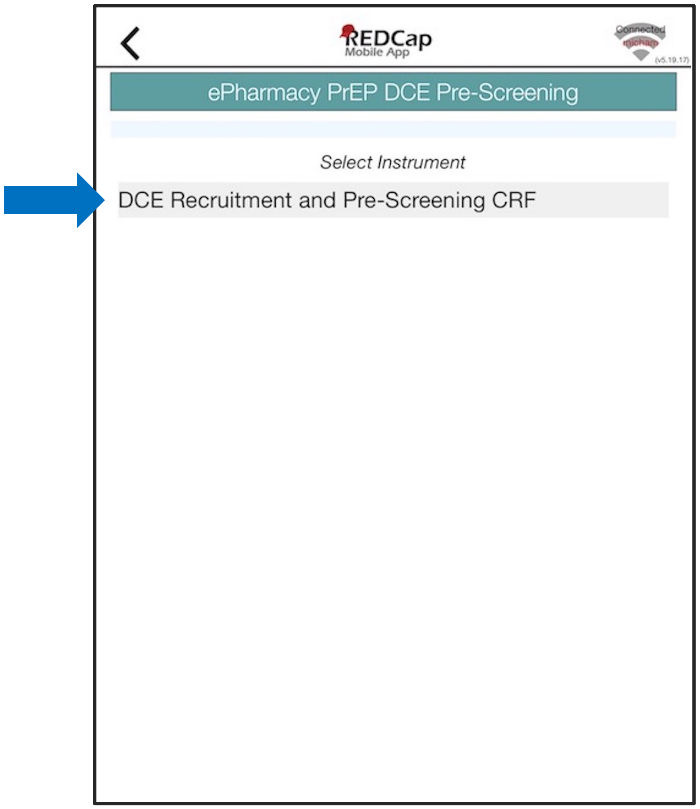

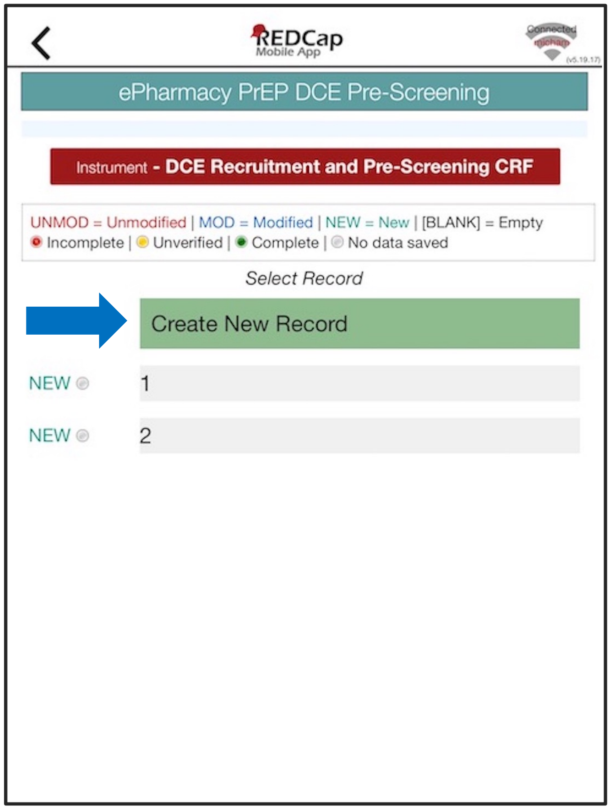

Eligibility Assessment

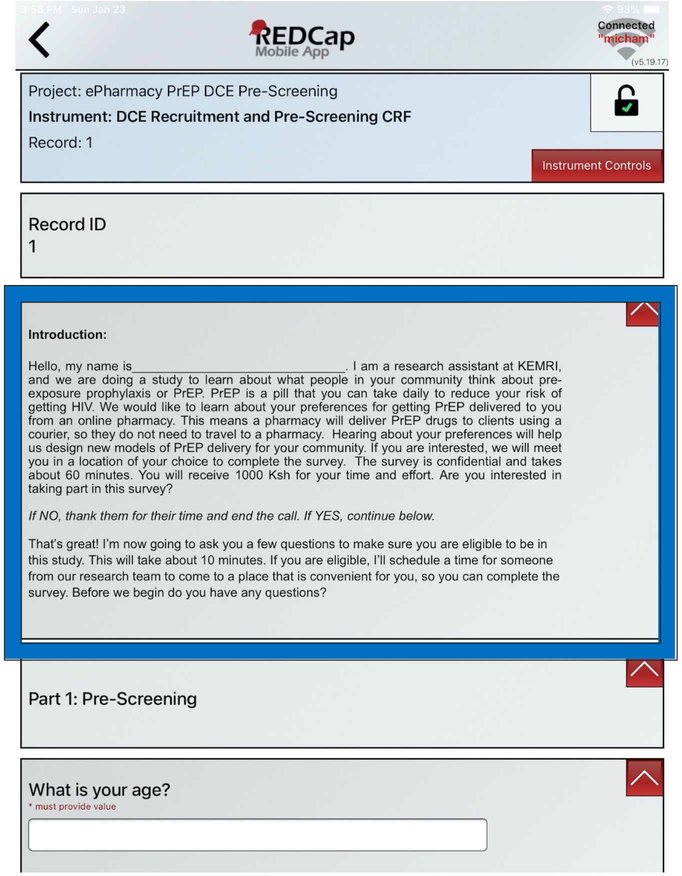

REDCap Mobile App

Connected "micham" (v5.19.17)

Project: ePharmacy PrEP DCE Pre-Screening

Instrument: DCE Recruitment and Pre-Screening CRF

Record: 1

Instrument Controls

Record ID
1

Introduction:

Hello, my name is______________________________. I am a research assistant at KEMRI, and we are doing a study to learn about what people in your community think about pre-exposure prophylaxis or PrEP. PrEP is a pill that you can take daily to reduce your risk of getting HIV. We would like to learn about your preferences for getting PrEP delivered to you from an online pharmacy. This means a pharmacy will deliver PrEP drugs to clients using a courier, so they do not need to travel to a pharmacy. Hearing about your preferences will help us design new models of PrEP delivery for your community. If you are interested, we will meet you in a location of your choice to complete the survey. The survey is confidential and takes about 60 minutes. You will receive 1000 Ksh for your time and effort. Are you interested in taking part in this survey?

If NO, thank them for their time and end the call. If YES, continue below.

That's great! I'm now going to ask you a few questions to make sure you are eligible to be in this study. This will take about 10 minutes. If you are eligible, I'll schedule a time for someone from our research team to come to a place that is convenient for you, so you can complete the survey. Before we begin do you have any questions?

Part 1: Pre-Screening

What is your age?
* must provide value

Eligibility Assessment

Introduction:

Hello, my name is_______________________________. I am a research assistant at KEMRI, and we are doing a study to learn about what people in your community think about pre-exposure prophylaxis or PrEP. PrEP is a pill that you can take daily to reduce your risk of getting HIV. We would like to learn about your preferences for getting PrEP delivered to you from an online pharmacy. This means a pharmacy will deliver PrEP drugs to clients using a courier, so they do not need to travel to a pharmacy. Hearing about your preferences will help us design new models of PrEP delivery for your community. If you are interested, we will meet you in a location of your choice to complete the survey. The survey is confidential and takes about 60 minutes. You will receive 1000 Ksh for your time and effort. Are you interested in taking part in this survey?

If NO, thank them for their time and end the call. If YES, continue below.

That's great! I'm now going to ask you a few questions to make sure you are eligible to be in this study. This will take about 10 minutes. If you are eligible, I'll schedule a time for someone from our research team to come to a place that is convenient for you, so you can complete the survey. Before we begin do you have any questions?

Eligibility Assessment – Not eligible

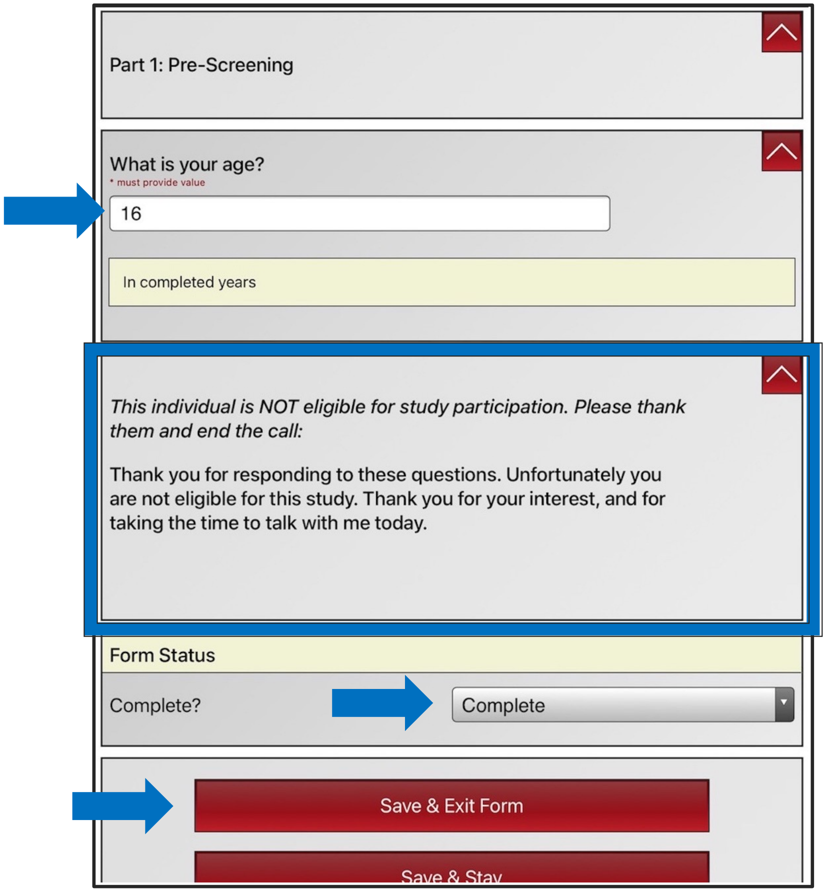

Eligibility Assessment – Not eligible

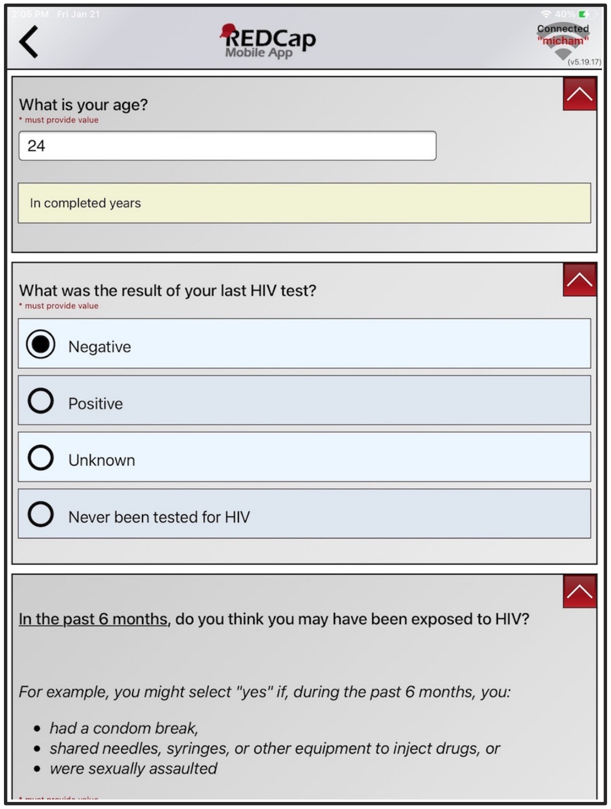

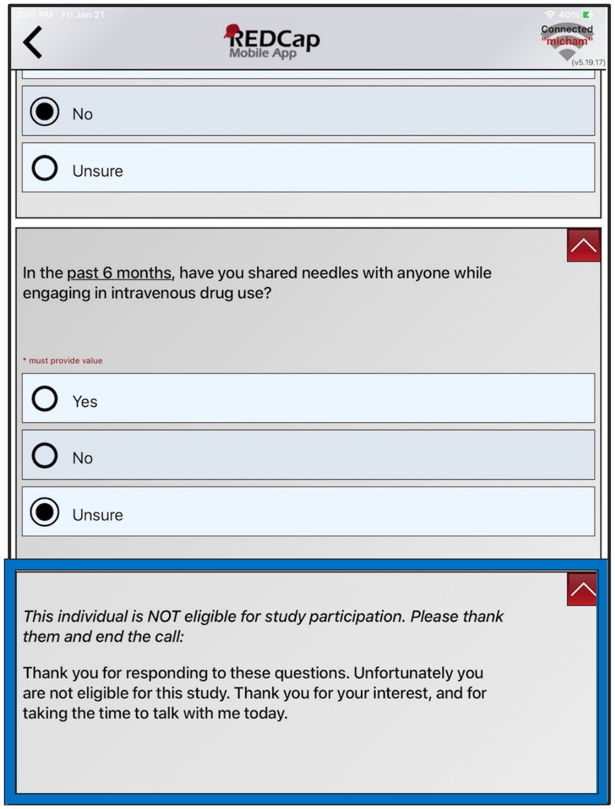

Eligibility Assessment – Eligible

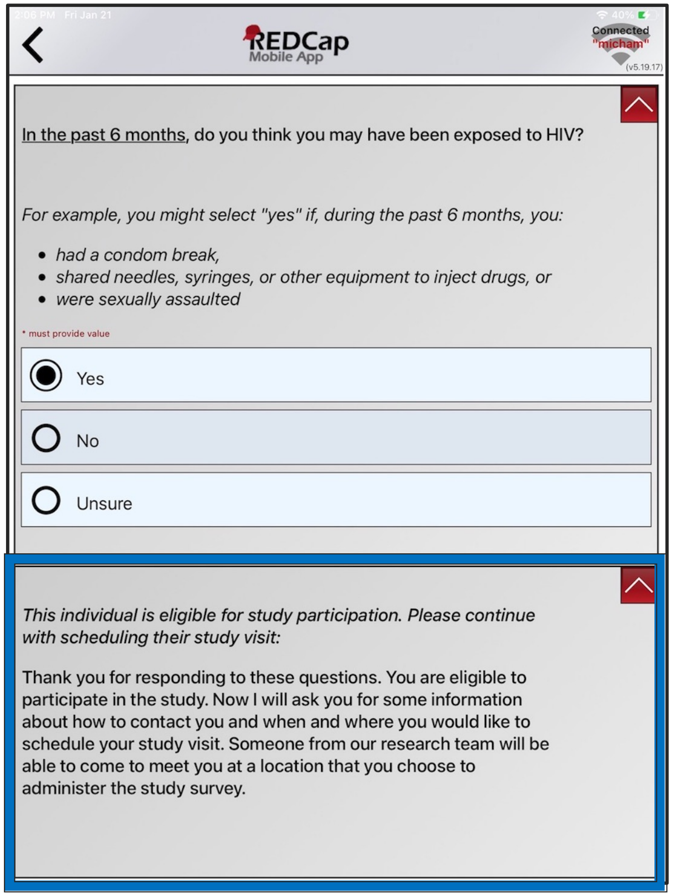

Eligibility Assessment – Scheduling and Contact Info

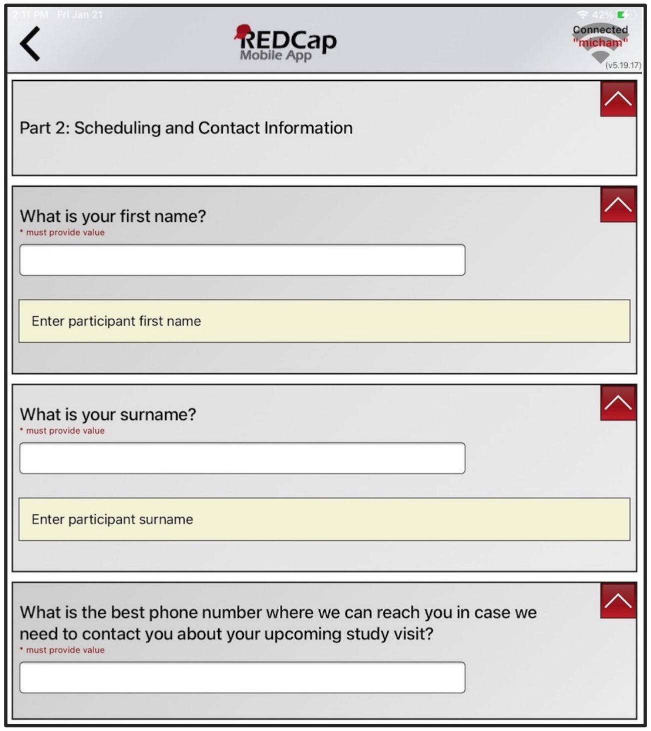

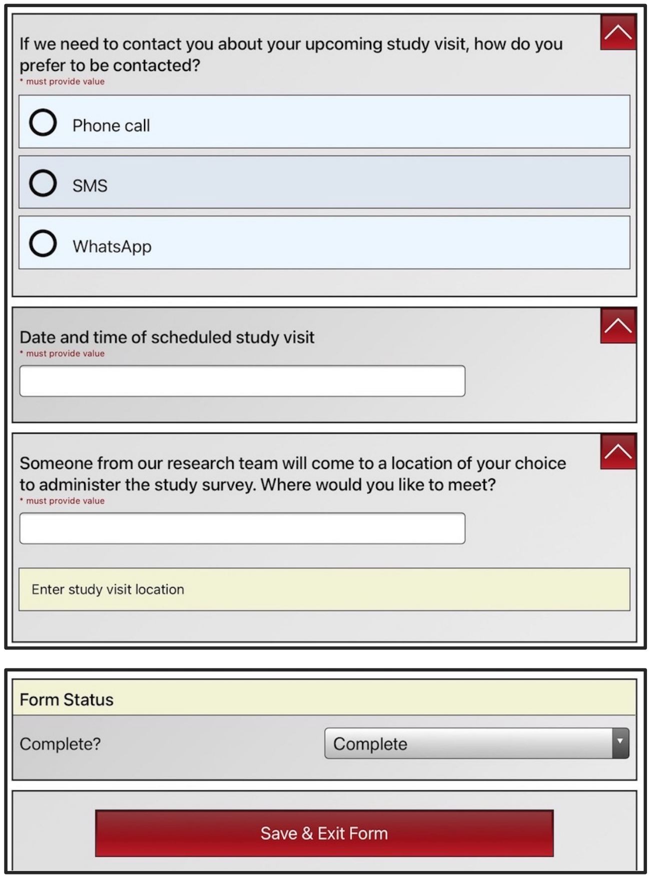

Consent procedures

- Research assistant will meet participant with tablet to administer ICF and questionnaire
- eConsent via REDCap app
- Review ICF with participant and answer questions
- Participant signs ICF on tablet

eConsent

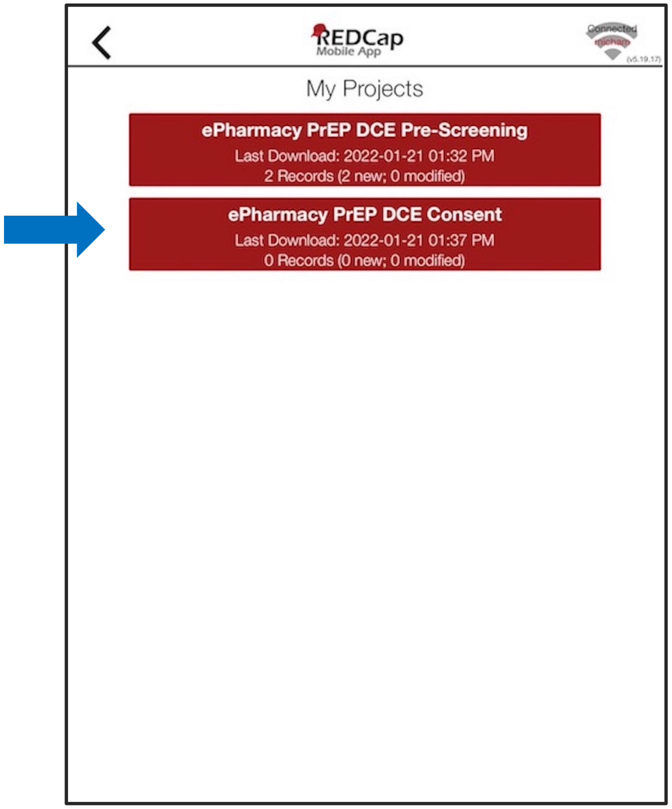
REDCap
Mobile App
My Projects
ePharmacy PrEP DCE Pre-Screening
Last Download: 2022-01-21 01:32 PM
2 Records (2 new; 0 modified)
ePharmacy PrEP DCE Consent
Last Download: 2022-01-21 01:37 PM
0 Records (0 new; 0 modified)

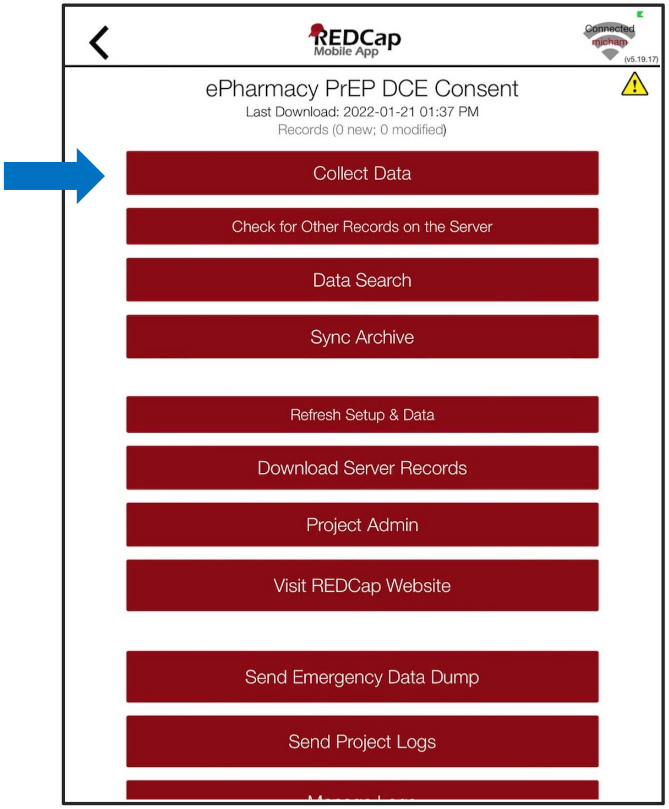
REDCap
Mobile App
ePharmacy PrEP DCE Consent
Last Download: 2022-01-21 01:37 PM
Records (0 new; 0 modified)
Collect Data
Check for Other Records on the Server
Data Search
Sync Archive
Refresh Setup & Data
Download Server Records
Project Admin
Visit REDCap Website
Send Emergency Data Dump
Send Project Logs

eConsent

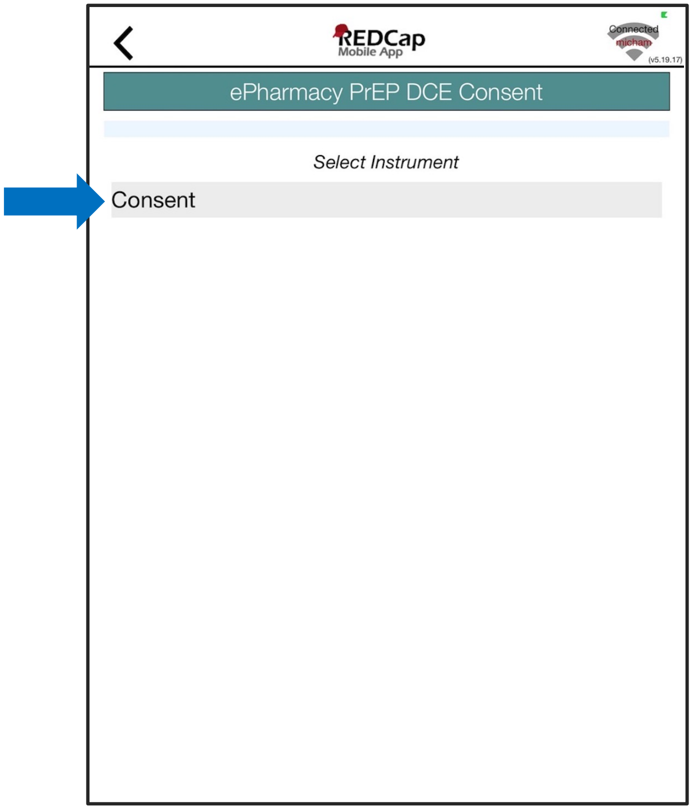
REDCap
Mobile App
ePharmacy PrEP DCE Consent
Select Instrument
Consent

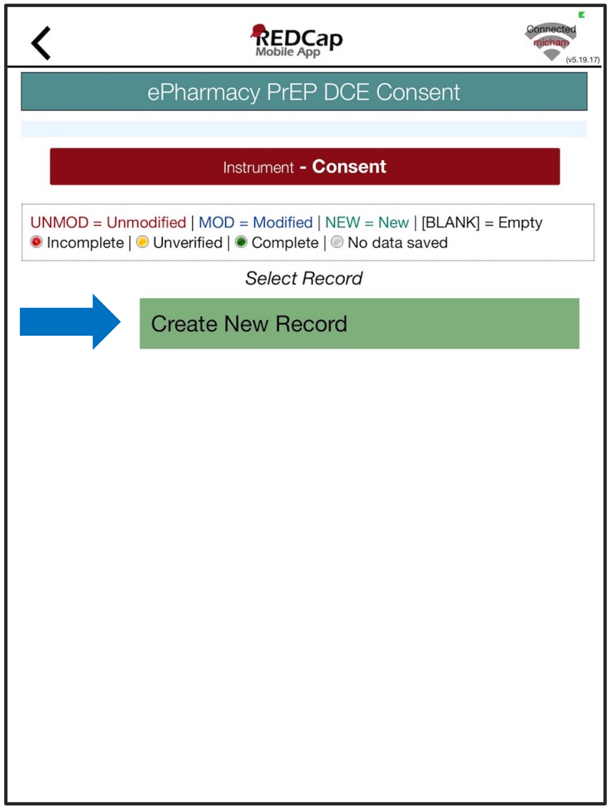
REDCap
Mobile App
ePharmacy PrEP DCE Consent
Instrument - Consent
UNMOD = Unmodified | MOD = Modified | NEW = New | [BLANK] = Empty
Incomplete | Unverified | Complete | No data saved
Select Record
Create New Record

Sawtooth: types of questions

- Labels to be read
- Multiple-Choice
- Checklist
- Entering a Number or Text or Date
- DCE Scenarios

For peer review only - http://bmjopen.bmj.com/site/about/guidelines.xhtml

Sawtooth: labels to be read

- Labels do not require data entry
- Labels will have information for you and/or the participant
- In some cases, labels can be a script that can read aloud to participants
- For example, in **Questionnaire: Introduction;**

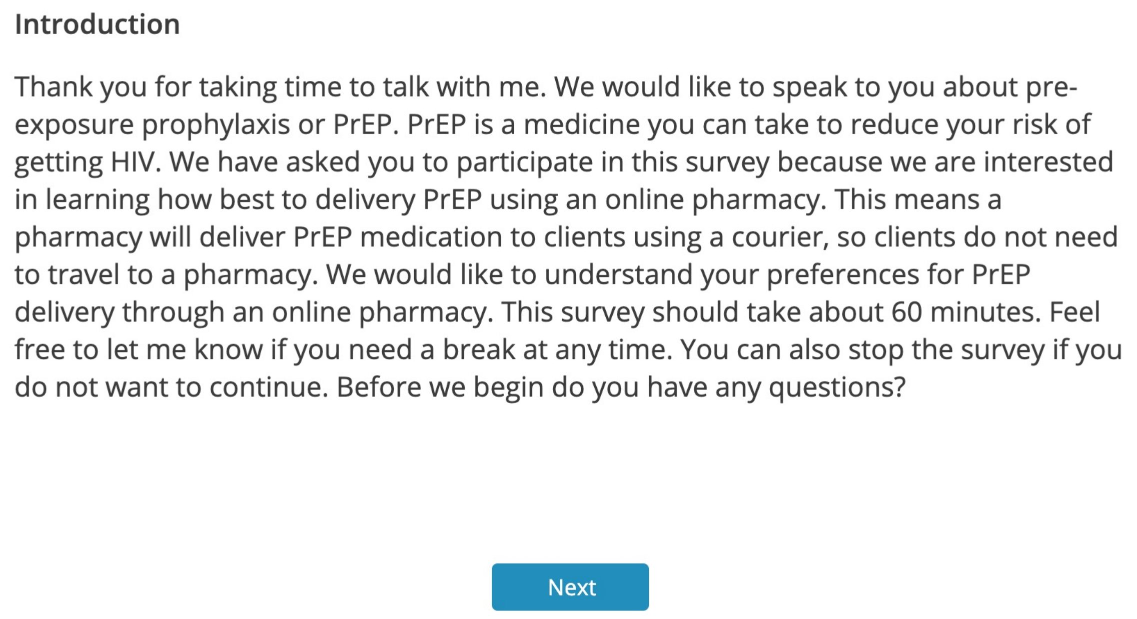

Sawtooth: multiple-choice questions

- You can only select one answer
- Example, **Questionnaire: Part 1: Eligibility Assessment;**

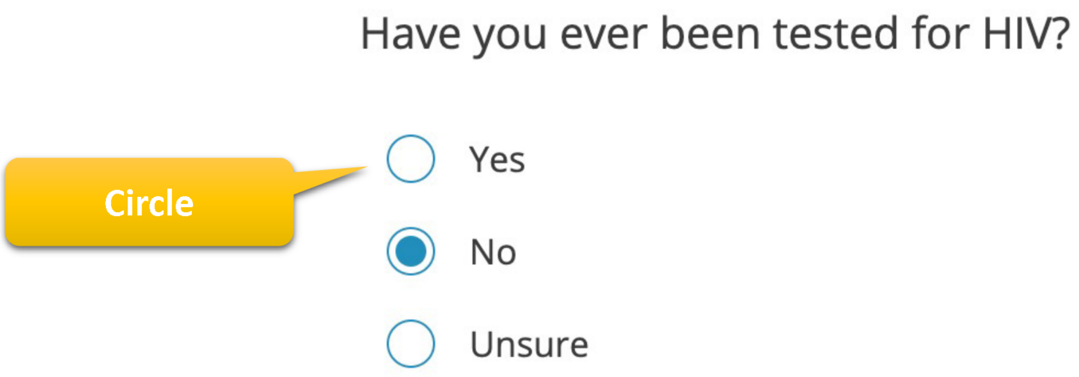

Sawtooth: checklist questions

- Here, you may select as many options as you like
- You should select all the answers that apply
- Example, **Questionnaire: Part 6: E-Pharmacy Engagement and HIV Self-Testing**

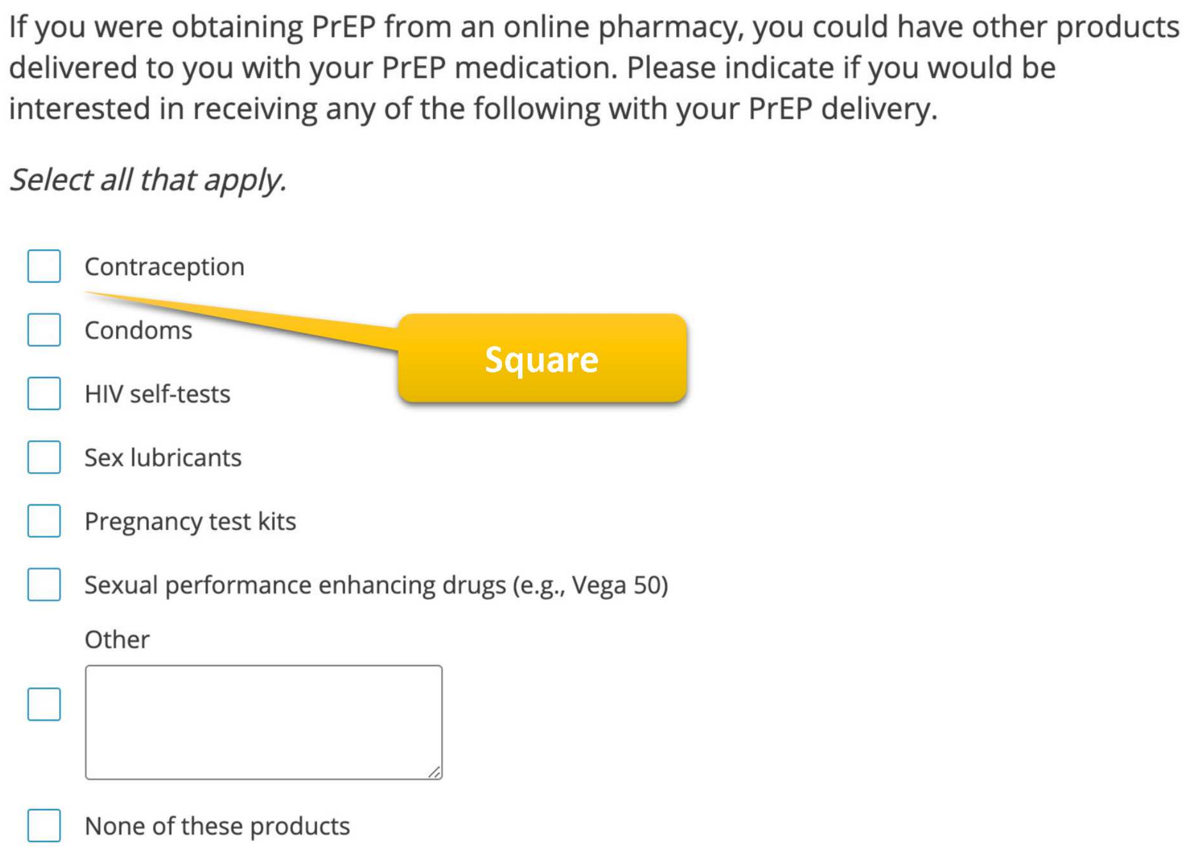

If you were obtaining PrEP from an online pharmacy, you could have other products delivered to you with your PrEP medication. Please indicate if you would be interested in receiving any of the following with your PrEP delivery.

Select all that apply.

- [ ] Contraception
- [ ] Condoms
- [ ] HIV self-tests
- [ ] Sex lubricants
- [ ] Pregnancy test kits
- [ ] Sexual performance enhancing drugs (e.g., Vega 50)
- [ ] Other
- [ ] None of these products

Sawtooth: entering numbers

- Entering number examples

Questionnaire: Part 1: Eligibility Assessment;

What is your age in years? [ ]

Questionnaire: Part 6: E-Pharmacy Engagement and HIV Self-Testing;

For a blood-based HIV self-test, delivered via a courier to a location of your choice (one-time cost):

What is the lowest price you are willing to pay? [ ] KSH

What is the highest price you are willing to pay? [ ] KSH

What price do you consider ideal? [ ] KSH

Sawtooth: entering text

- Entering text example
- Text questions will most often appear to clarify “Other”

If you were obtaining PrEP from an online pharmacy, you could have other products delivered to you with your PrEP medication. Please indicate if you would be interested in receiving any of the following with your PrEP delivery.

Select all that apply.

- ☐ Contraception
- ☐ Condoms
- ☐ HIV self-tests
- ☐ Sex lubricants
- ☐ Pregnancy test kits
- ☐ Sexual performance enhancing drugs (e.g., Vega 50)
- ☐ Other
- ☐ None of these products

Sawtooth: entering date

- Entering date examples
- Date questions will have drop-down menus

Questionnaire: Part 1: Eligibility Assessment

When was your last HIV test?

Month: June Year: 2017

Questionnaire: Part 2: PrEP Knowledge and Interest

When did you first start taking PrEP?

Month: Please select Year: Please select

Sawtooth: required questions

- Almost every question requires an answer
- If you try to move on without providing an answer, a red screen will appear with a reminder that the response is required.

⚠ A response is required.

What is your age in years?

Sawtooth: DCE scenarios

- Explain in the next section

For peer review only - http://bmjopen.bmj.com/site/about/guidelines.xhtml

Structure of the Questionnaire

- Introduction
- Part 1: Eligibility Assessment
- Part 2: PrEP Knowledge and Interest
- Part 3: Introduction to Discrete Choice Experiment
- Part 4: Definition of Characteristics
- Part 5: Scenarios
- Part 6: E-Pharmacy Engagement and HIV Self-Testing
- Part 7: Participant Demographics
- Part 8: Sexual Behavior

For peer review only - http://bmjopen.bmj.com/site/about/guidelines.xhtml

Structure of the Questionnaire

- **Introduction**
- **Part 1: Eligibility Assessment**
- **Part 2: PrEP Knowledge and Interest**
- Part 3: Introduction to Discrete Choice Experiment
- Part 4: Definition of Characteristics
- Part 5: Scenarios
- Part 6: E-Pharmacy Engagement and HIV Self-Testing
- Part 7: Participant Demographics
- Part 8: Sexual Behavior

For peer review only - http://bmjopen.bmj.com/site/about/guidelines.xhtml

Introduction

Introduction

Thank you for taking time to talk with me. We would like to speak to you about pre-exposure prophylaxis or PrEP. PrEP is a medicine you can take to reduce your risk of getting HIV. We have asked you to participate in this survey because we are interested in learning how best to delivery PrEP using an online pharmacy. This means a pharmacy will deliver PrEP medication to clients using a courier, so clients do not need to travel to a pharmacy. We would like to understand your preferences for PrEP delivery through an online pharmacy. This survey should take about 60 minutes. Feel free to let me know if you need a break at any time. You can also stop the survey if you do not want to continue. Before we begin do you have any questions?

Next

Participant ID

- Participant ID should be entered twice
- Same PTID as ICF – links questionnaire responses to ICF

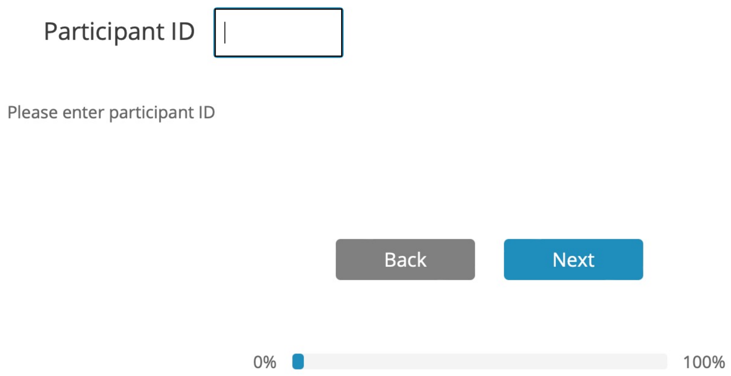

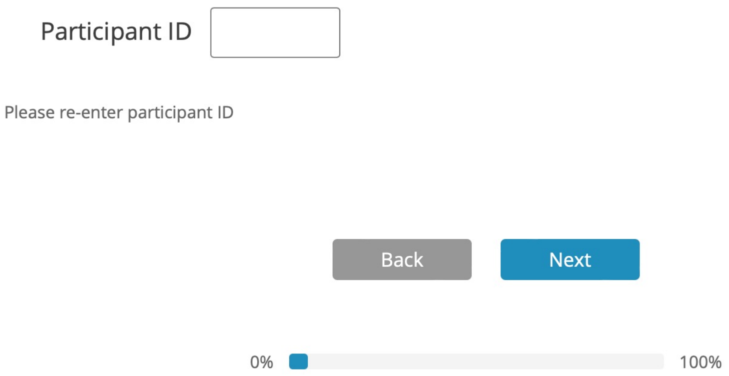

Part 1: Eligibility Assessment

- Eligibility assessment to be completed again at time of questionnaire administration

Part 1: Eligibility Assessment

What is your age in years? [ ]

Back | Next

In the past 6 months, do you think you may have been exposed to HIV?

For example, you might select "yes" if, during the past 6 months, you:

• had a condom break

• shared needles, syringes, or other equipment to inject drugs, or

• were sexually assaulted

- Yes
- No
- Unsure

In the past 6 months, have you had sex with more than one person?

- Yes
- No
- Unsure

Part 2: PrEP Knowledge and Interest

Part 2: PrEP Knowledge and Interest

Have you ever heard of pre-exposure prophylaxis, or PrEP, for HIV prevention before today?

- ◯ Yes
- ◯ No

Back | Next

Structure of the Questionnaire

- Introduction
- Part 1: Eligibility Assessment
- Part 2: PrEP Knowledge and Interest
- **Part 3: Introduction to Discrete Choice Experiment**
- **Part 4: Definition of Characteristics**
- **Part 5: Scenarios**
- **Part 6: E-Pharmacy Engagement and HIV Self-Testing**
- Part 7: Participant Demographics
- Part 8: Sexual Behavior

Part 3: Introduction to Discrete Choice Experiment

As we mentioned, we're designing a new way to deliver PrEP through an online pharmacy. We would like to understand your preferences to help us design this service. Online PrEP delivery includes different services, including an assessment to determine if you are at risk for HIV, HIV testing to confirm you are HIV-negative, and talking to a medical provider to make sure PrEP is safe for you. To determine your preferences for these services, we will show you a series of **9** scenarios with **2** options for online PrEP service delivery. For each of these scenarios, we will ask you to choose the delivery strategy you most prefer.

Back   Next

Part 4: Definition of Characteristics

Here we describe the characteristics of obtaining PrEP through online pharmacy in the order you would experience them. These are the characteristics we will ask you to consider when making a decision about which option you prefer. Please consider ONLY these characteristics when making your choices.

Clinical Consultation

You would then have a clinical consultation with a medical provider to make sure PrEP is safe for you to use. This can be done over the phone or a video chat.

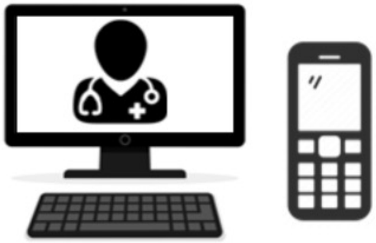

Another option is to see a provider in person at a setting of your choice.

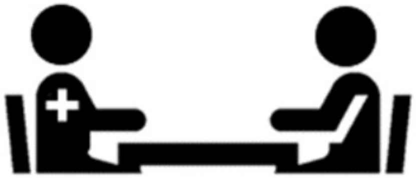

Part 5: Scenarios

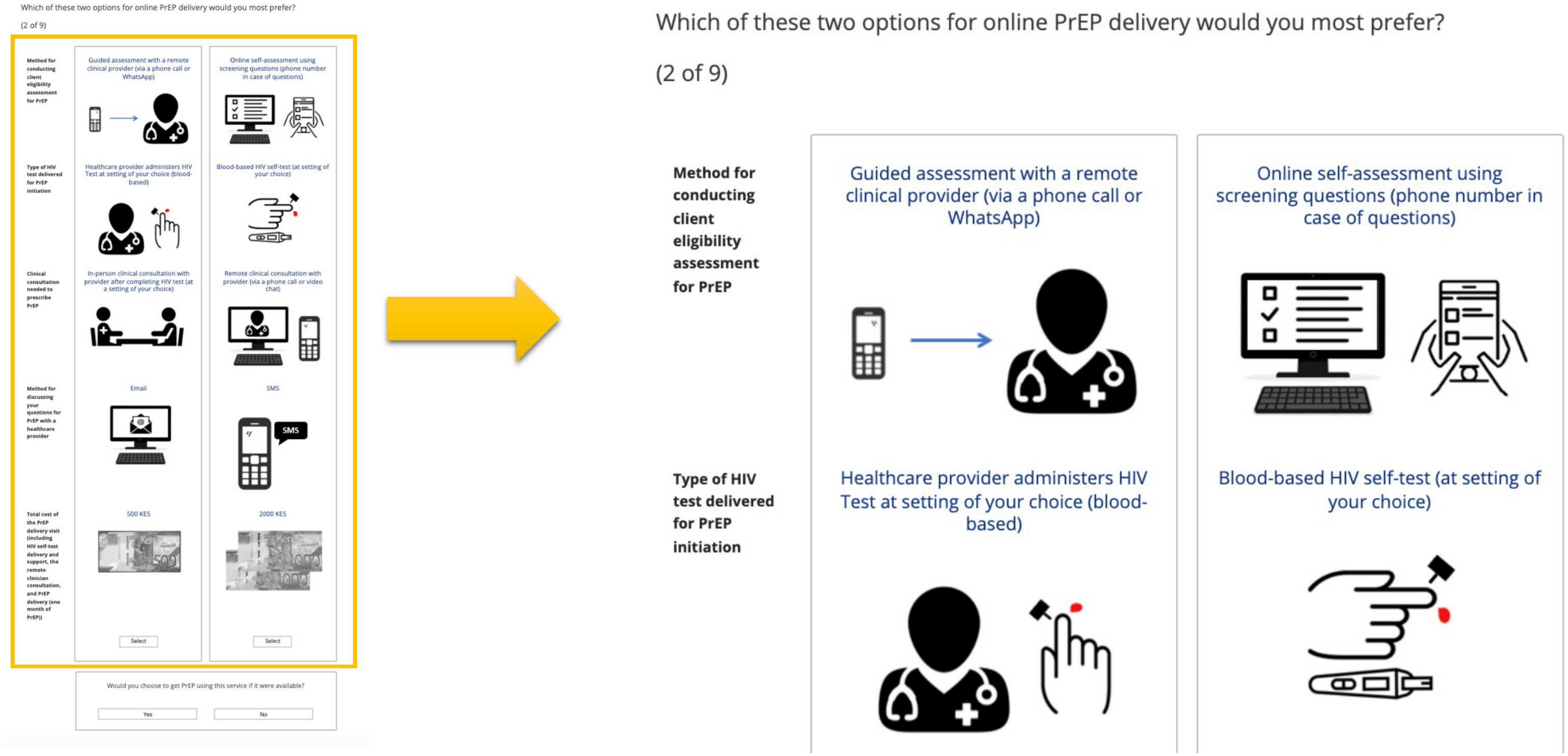

Part 5: Scenarios

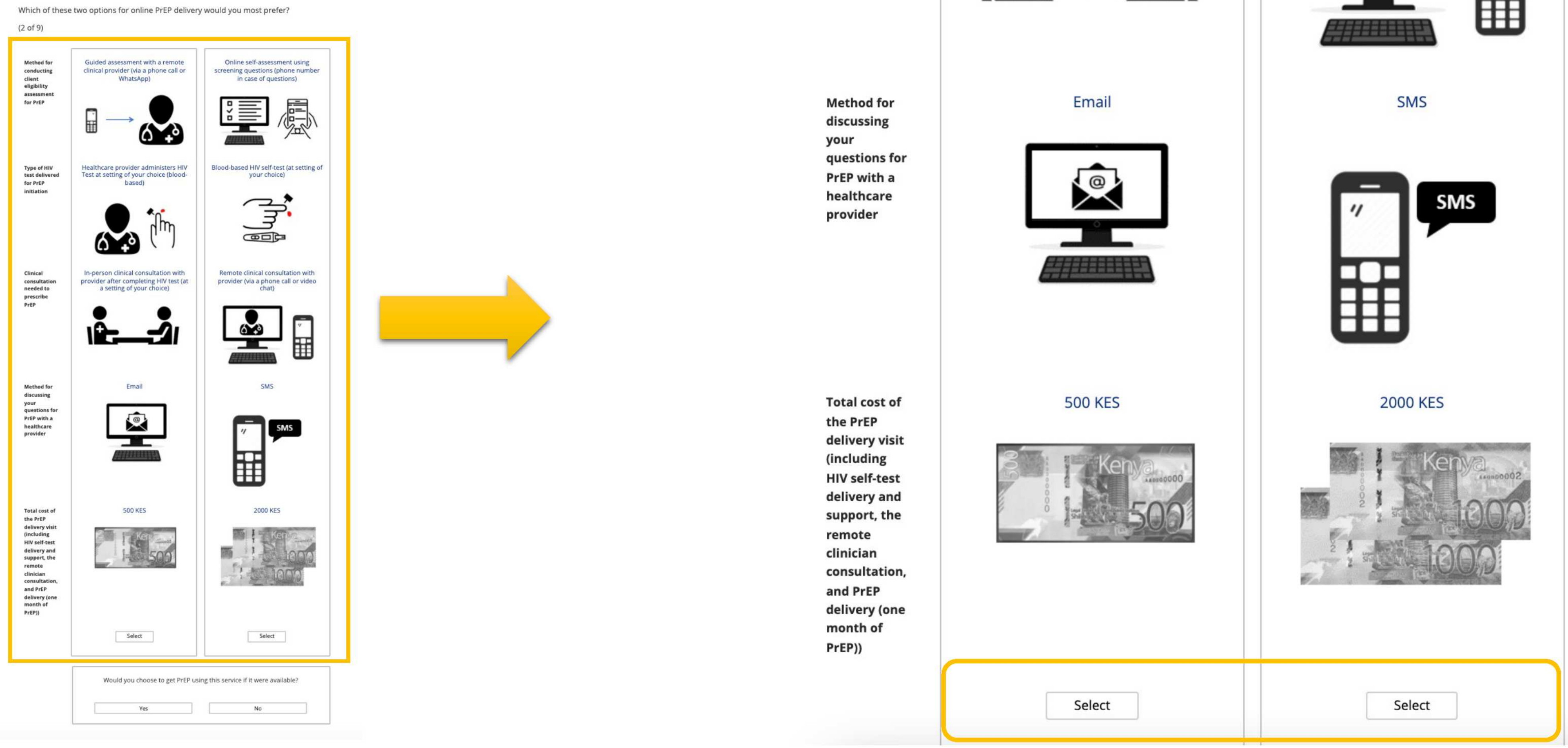

Part 5: Scenarios

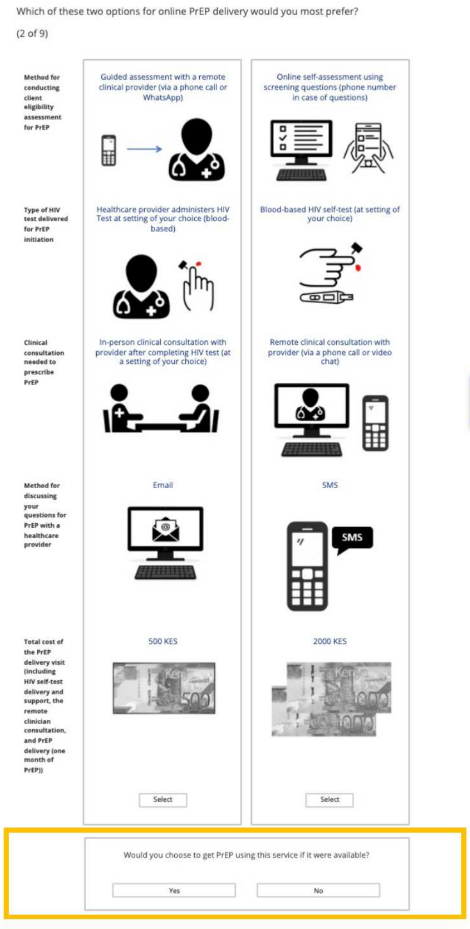

Dual-response question:

Would you choose to get PrEP using this service if it were available?

Yes | No

Back | Next

Part 6: E-Pharmacy Engagement and HIV Self-Testing

- Willingness-to-pay questions

The next set of questions will ask about how much you would like to pay for different parts of online PrEP delivery. For each part, please provide the lowest price, the maximum price and the ideal price you are willing to pay. For the lowest price, please tell us the lowest amount you think the service should cost because otherwise you would be concerned about its quality, and you would not purchase the service. In answering these questions, please consider your usual expenses. Remember that there are no right or wrong answers. We're interested in your preference. You can write zero if you feel that most accurately reflects your preference.

For a blood-based HIV self-test, delivered via a courier to a location of your choice (one-time cost):

What is the lowest price you are willing to pay? [ ] KSH

What is the highest price you are willing to pay? [ ] KSH

What price do you consider ideal? [ ] KSH

Structure of the Questionnaire

- Introduction
- Part 1: Eligibility Assessment
- Part 2: PrEP Knowledge and Interest
- Part 3: Introduction to Discrete Choice Experiment
- Part 4: Definition of Characteristics
- Part 5: Scenarios
- Part 6: E-Pharmacy Engagement and HIV Self-Testing
- **Part 7: Participant Demographics**
- **Part 8: Sexual Behavior**

Part 7: Participant Demographics

Part 7: Participant Demographics

In what region do you live?

- North Eastern
- Nyanza
- Western
- Rift Valley
- Nairobi
- Central
- Coast
- Eastern

What is the highest level of education you have completed?

- Primary
- Secondary
- O Levels
- A Levels
- Technical or vocational school
- University or higher

Part 8: Sexual Behavior

- Prefer not to answer option

Part 8: Sexual Behavior

In the last 3 months, have you had a primary sex partner?

(A primary sex partner is a person you have sex with on a regular basis, or someone you consider to be your main partner.)

- ○ Yes
- ○ No
- ◉ Prefer not to answer

Submitting data

The questionnaire ends here. Thank you for your responses.

0% 100%

Questions?

ePharmacy PrEP: Discrete Choice Experiment Training

Day 1 Session 3

24th-25th January 2022

Cognitive Interviews, Pilot, and DCE Administration

Process

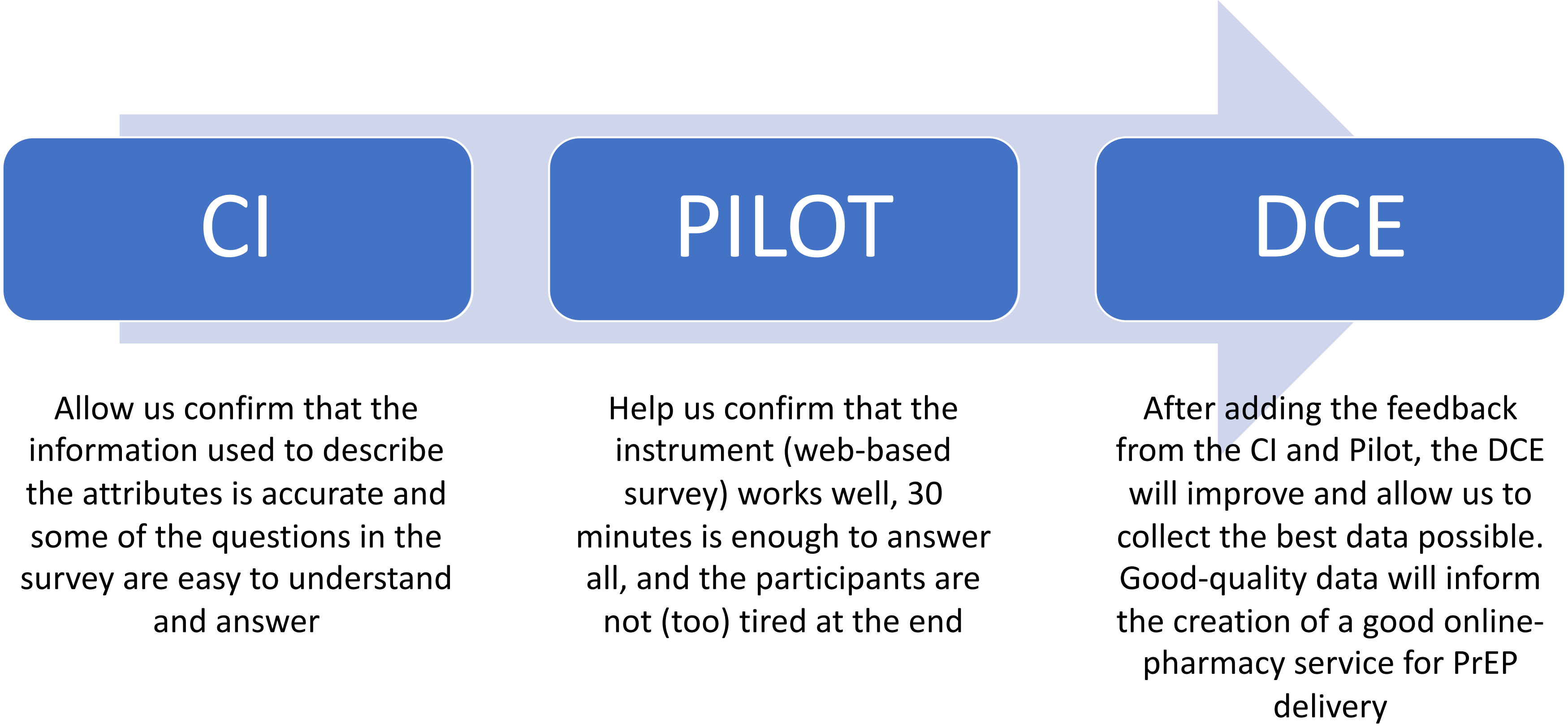
CI
PILOT
DCE
Allow us confirm that the information used to describe the attributes is accurate and some of the questions in the survey are easy to understand and answer
Help us confirm that the instrument (web-based survey) works well, 30 minutes is enough to answer all, and the participants are not (too) tired at the end
After adding the feedback from the CI and Pilot, the DCE will improve and allow us to collect the best data possible. Good-quality data will inform the creation of a good online-pharmacy service for PrEP delivery

Key characteristics

Cognitive Interviews

- Aim to obtain feedback in specific sections of the instrument
- Length: around 1 hour
- Uses a script
- RA very involved

Pilot

- Aim to verify usability and fatigue in the entire instrument
- Length: around 45 min
- No script
- RA involved but less

DCE

- Aims to obtain data of the participants' preferences as unbiased as possible
- Length: 30-45 min
- No script
- RA only involved if participant has questions

Structure – Cognitive Interviews

1. Approach and recruitment
    - In-person
    - Objective: to collect participants' feedback about the DCE attributes and specific sections of the instrument (see script)
    - Length: around 60 minutes
2. Presentation of the instrument
3. Active collection of feedback

Let's see the script

Structure – Pilot

1. Approach and recruitment
   - In-person
   - Objective: to verify if the instrument is friendly and easy to use, if the participants get stuck in any question, if any questions are not clear, how long does it take to complete the instrument, and how tired or bored is the participant at the end
   - Length: around 45 minutes
2. Presentation of the instrument
3. Passive collection of feedback:

   Observe closely while the participant goes through the survey, if at any point there's confusion ask what's happening and offer help. Take notes every time there's confusion or the participant needed clarification, how long it took to finish the survey, and if they were tired and to what extend.

Let's see the instrument

Structure – DCE

1. Approach and recruitment
   - In-person
   - Objective: to collect high-quality data about the participants' preferences.

   We assist but we don't advice
   - Length: around 30-45 minutes
2. Presentation of the instrument
3. Passive collection of feedback:

   After recruitment, present the survey to the participant and let them fill their answers.

Structure – DCE cont.

Clarifying vs. Advice questions

- Clarifying: Related to the meaning of a word or the sense of the question in case of ambiguity or vague meaning
  - Examples:
    - What does ‘pay-by-piece’ mean?
    - Is contraception the same as emergency pills?
    - Where do I click if I don’t know the answer to this question (or don’t want to tell)?
- Advice: asks for your opinion or advice
  - Examples:
    - How can I know if clinical consultation is more important than Cost?
    - Do you think oral HIV testing is easier than when they draw blood?

Summary

- Each step help us have the best possible instrument for the DCE
- Cognitive Interviews require the most involvement to obtain feedback, followed by Pilot, and in the DCE we collect no feedback but just administer the survey
- In the DCE we want to collect only the participants' preferences, so we avoid providing any opinion or advice, even if they ask
- Questions?

For peer review only - http://bmjopen.bmj.com/site/about/guidelines.xhtml

Supplement 3: Study Questionnaire

DISCRETE CHOICE EXPERIMENT QUESTIONNAIRE

Introduction:

Thank you for taking time to talk with me. We would like to speak to you about pre-exposure prophylaxis or PrEP. PrEP is a medicine you can take to reduce your risk of getting HIV. We have asked you to participate in this survey because we are interested in learning how best to delivery PrEP using an online pharmacy. This means a pharmacy will deliver PrEP medication to clients using a courier, so clients do not need to travel to a pharmacy. We would like to understand your preferences for PrEP delivery through an online pharmacy. This survey should take about 45 minutes. Feel free to let me know if you need a break at any time. You can also stop the survey if you do not want to continue. Before we begin do you have any questions?

RA Initials
Research Assistant Initials
(The RA conducting this questionnaire should enter their initials.)

Participant ID
Please enter participant ID from enrollment log.
(This should match the PTID entered on the REDCap consent form.)

Please re-enter participant ID from enrollment log.
(This should match the PTID entered on the REDCap consent form.)

Part 1: Eligibility Assessment

1	What is your age in years?	|__|__| years <18, Stop screening. This individual is <u>NOT</u> eligible for study participation. Thank them for their time
2	Have you ever been tested for HIV?	☐ *Yes* ☐ *No* ☐ *Unsure*
3	When was your last HIV test?	_______ Month ______Year
4	What was the result of your last HIV test?	☐ *Negative*<br>☐ *Positive*<br>☐ *Unknown*<br><br>If positive, this individual is <u>NOT</u> eligible for study participation. Stop screening and thank them for their time.

Part 2: PrEP Knowledge and Interest

5	Have you ever heard of pre-exposure prophylaxis, or PrEP, for HIV prevention before today?	Yes<br>No
6	Have you ever taken PrEP to prevent HIV? *[If 5=Yes]*	Yes<br>No<br>I don't know<br>Prefer not to answer
6.1	Are you currently taking PrEP to prevent HIV? *[If Q6 = Yes]*	Yes<br>No

For peer review only

6.2	When did you first start taking PrEP? *[If Q6 = Yes]*	Month: Year:<br>MM/YYYY
7	Would you be interested in taking PrEP to prevent HIV? *[If Q6 != Yes]*	Yes<br>No<br>I don't know

Persons who answer NO to both of Q6, Q7 are not eligible for participation

If participant is not eligible: Based on your responses, you are not eligible to continue with the questionnaire. Thank you for taking the time to respond.

Part 3: Introduction to Discrete Choice Experiment

As we mentioned, we're designing a new way to deliver PrEP through an online pharmacy. We would like to understand your preferences to help us design this service. Online PrEP delivery includes different services, including an assessment to determine if you are at HIV risk, HIV testing to confirm you are HIV-negative, and talking to a medical provider to make sure PrEP is safe for you. To determine your preferences for these services, we will show you a series of **8** scenarios with 2 options for online PrEP service delivery. For each of these scenarios, we will ask you to choose the delivery strategy you most prefer.

Part 4: Definition of Characteristics

Part 5: Scenarios

We will now ask you about how you would like to get PrEP from an online pharmacy. Please remember there are no wrong answers, we want to know your preferences. When answering each scenario, imagine that you are planning to get PrEP using an online pharmacy. We will also ask you if you would choose to get PrEP using the option you selected. If you would not choose to get PrEP if only these two options were available, you can answer no to this question.

Scenarios

Which of these two options for online PrEP delivery would you most prefer?

If the PrEP service you just chose was available, do you think you would actually use it?

Part 6: E-Pharmacy Engagement and HIV Self-Testing

8	Have you ever purchased medication or other products from an online pharmacy (e-pharmacy)?	Yes<br>No
9	Have you ever purchased any of the following medications or products from an online pharmacy (e-pharmacy)? *[If Q8 = Yes]*<br>*Select all that apply*	Contraception<br>Condoms<br>HIV self-tests<br>Sex lubricants<br>Pregnancy test kits<br>Sexual performance enhancing drugs (e.g., Vega 50)<br>Other<br>None of these services
10	How would you most prefer to obtain your PrEP medication? *[If Q7 = Yes]*	Clinic<br>Community pharmacy or chemist<br>Online pharmacy (e-pharmacy)<br>Community health worker (e.g., home visit)<br>Other

For peer review only

11	If you were obtaining PrEP from an online pharmacy, you could have other products delivered to you with your PrEP medication. Please indicate if you would be interested in receiving any of the following with your PrEP delivery. *Select all that apply*	Pregnancy test kits<br>Contraception<br>Condoms<br>Sex lubricants<br>Sexual performance enhancing drugs (e.g., Vega 50)<br>HIV self-test<br>None of these products<br>Other ______________
12	If you were obtaining PrEP from an online pharmacy, how frequently would you prefer to refill your PrEP medication?	Every 1 month<br>Every 3 months<br>Every 6 months<br>Other ________
The next set of questions will ask about how much you would like to pay for different parts of online PrEP delivery. For each part, please provide the maximum price you are willing to pay. In answering these questions, please consider your usual expenses. Remember that there are no right or wrong answers. We're interested in your preference. You can write zero if you feel that most accurately reflects your preference.		
13	What is the maximum price you are willing to pay for a blood-based HIV self-test?	__________ KSH
14	What is the maximum price you are willing to pay for an oral HIV self-test?	__________ KSH
15	What is the maximum price you are willing to pay for remote/online clinical consultation to obtain a prescription for PrEP based on your HIV self-test results (one-time cost)?	__________ KSH
16	What is the maximum price you are willing to pay for a one-month supply of PrEP medicines?	__________ KSH
17	What is the maximum price you are willing to pay for delivery of PrEP medicines to a setting of your choice (one-time courier fee)?	__________ KSH
18	What is the maximum price you are willing to pay in total for the package of services associated online PrEP delivery (this includes courier delivery of an HIV self-test, a remote clinical consultation, and courier-delivered PrEP)?	__________ KSH
19	How would you most prefer to pay for the package of online PrEP services (e.g., HIV testing, remote consultation, PrEP medication delivery)?	Pay by piece (e.g., pay separately for HIV testing, the remote clinic visit, and PrEP drug delivery)<br>Pay per PrEP visit (including HIV testing, the remote visit, and PrEP medication delivery)<br>Pay a monthly subscription
The next set of questions ask about your preferences for online PrEP delivery		
20	What support, if any, would you be interested in from the online pharmacy provider to help you consistently take your PrEP medication? (select all that apply)	SMS reminders<br>Emails<br>Phone calls<br>Video calls<br>No support needed
21	At what frequency, would you like support for taking your PrEP medication?	Daily<br>Weekly<br>Bi-weekly (every other week)<br>Monthly
22	Have you heard about HIV self-testing as a method for testing for HIV before today?	Yes<br>No<br>Prefer not to answer

For peer review only

23	Have you ever used a self-test to test for HIV?	Yes<br>No<br>Prefer not to answer
24	How much would you like or dislike obtaining PrEP though an online platform?	Strongly dislike<br>Somewhat dislike<br>No opinion<br>Somewhat like<br>Strongly like
25	How much effort do you think it would take you to obtain PrEP online?	No effort<br>A little effort<br>A moderate effort<br>A huge amount of effort<br>No opinion
26	How confident do you feel about your ability to navigate an online website to obtain PrEP?	Very unconfident<br>Somewhat unconfident<br>No opinion<br>Somewhat confident<br>Very confident
27	How much do you agree or disagree with the following statement: Online PrEP delivery would help reduce HIV in my community.	Strongly disagree<br>Somewhat disagree<br>No opinion<br>Somewhat agree<br>Strongly agree
28	Which media sources do you most frequently use to get general information; such as daily news, important events, etc.	Newspapers<br>Radio<br>Television<br>WhatsApp groups<br>Social networks (e.g., Facebook, Instagram, Tik Tok)<br>Internet browsing (e.g., google, news' websites)
29	Which media sources do you most frequently use to get healthcare information; such as healthcare campaigns, interventions, new medicines, etc.	Newspapers<br>Radio<br>Television<br>WhatsApp groups<br>Social networks (e.g., Facebook, Instagram, Tik Tok)<br>Internet browsing (e.g., google, news' websites)

Part 7: Participant Demographics

30	In what region do you live?	North Eastern<br>Nyanza<br>Western<br>Rift Valley<br>Nairobi<br>Central<br>Coast<br>Eastern
31	What is your gender?	Male<br>Female<br>Other
32	Are you currently enrolled in school?	Yes<br>No
33	What is the highest level of education you have completed?	Primary<br>Secondary<br>O Levels

		A Levels<br>Technical or vocational school<br>University or higher<br>Prefer not to answer
34	Do you have regular employment?	Yes, I work full time<br>Yes, I work part time<br>Yes, I work multiple jobs<br>Yes, seasonal employment<br>No, I do not work<br>Prefer not to answer
35	How much money do you usually earn in a month?	|__|__|__|__|__| KES<br>Prefer not to answer
36	Have you ever been married?	Yes<br>No
37	What is your current relationship status?	Married<br>Steady boyfriend or girlfriend<br>Single<br>Widowed<br>Divorced or separated<br>Prefer not to answer
38	Have you ever been pregnant? *[If Q31 = female]*	Yes<br>No
39	Are you using any family planning method to delay or avoid pregnancy?<br>*[If Q31 = female]*	Yes<br>No<br>Prefer not to answer
40	What method(s) are you using? *[If Q39 = Yes]*	Oral pills<br>IUD<br>Implants<br>Injections<br>Condoms<br>Withdrawal<br>Rhythm or periodic abstinence<br>Emergency contraception<br>Tubal ligation/hysterectomy<br>Vasectomy<br>Traditional methods<br>Don’t know<br>Prefer not to answer<br>Other ________________
41	Where do you prefer to access these family planning methods? *[If Q39 = Yes]*	Community pharmacy/chemist<br>Online pharmacy<br>Health facility<br>Friend or family member<br>Sexual partner(s)<br>Other ________________

Part 8: Sexual Behavior

42	<u>In the last 3 months</u>, have you had a primary sex partner? (A primary sex partner is a person you have sex with on a regular basis, or someone you consider to be your main partner.)	Yes<br>No<br>Prefer not to answer
43	How long have you been with your primary partner? *[If Q42 = Yes]*	|__|__| years |__|__| months

For peer review only - http://bmjopen.bmj.com/site/about/guidelines.xhtml

44	In the past 3 months, have you had sex?	Yes<br>No
45	In the past 3 months, what types of sexual partners have you had? *[If Q44 = Yes]*	One primary partner only (no other partners)<br>One primary partner and casual partner(s)<br>Casual partner(s) only<br>Other ___________<br>Prefer not to answer
46	In the past 3 months, how many different people have you had sex with? *[If Q44 = Yes]*	|__|__|__| people<br>888 = Don't know<br>999 = Prefer not to answer
47	In the past 3 months, how many of these people were new sexual partners (i.e., you had never had sex with them before)? *[If Q44 = Yes]*	|__|__|__| people<br>888 = Don't know<br>999 = Prefer not to answer
48	In the past month, how many times did you have sex?	|__|__|__| times
49	When you had sex in the past month, how many times did you use a condom? *[If Q47 > 0]*	|__|__|__| times
The next set of questions ask about your sexual behavior in the past 6 months		
50	In the past 6 months, have you had sex without a condom?	☐ *Yes* ☐ *No* ☐ *Unsure*
51	In the past 6 months, do you think you may have been exposed to HIV?<br>*For example, you might select "yes" if, during the past 6 months, you:*<br>• *had a condom break*<br>• *shared needles, syringes, or other equipment to inject drugs, or*<br>• *were sexually assaulted*	☐ *Yes* ☐ *No* ☐ *Unsure*
52	In the past 6 months, have you had sex with more than one person?	☐ *Yes* ☐ *No* ☐ *Unsure*
53	Do you have any sex partners who are HIV-positive and:<br>• are not currently taking antiretroviral therapy (ART),<br>• have been taking antiretroviral therapy (ART) for less than 6 months,<br>• have been missing doses of their antiretroviral therapy (ART),<br>• have a detectable HIV viral load, or<br>• the two of you are trying to get pregnant?<br>*Select "yes" if ANY of the above statements are true about any of your sex partners who are HIV-positive.*	☐ *Yes* ☐ *No* ☐ *Unsure*

For peer review only

54	Do you have any sex partner(s) who you think are at high risk for HIV and whose HIV status you do not know?<br>*Some example behaviors that may make your partner at high risk for HIV include:*<br>• *if he or she has other partners whose HIV status is unknown*<br>• *if he or she exchanges sex for money/gifts or money/gifts for sex*<br>• *if he or she engages in intravenous drug use*	☐ *Yes* ☐ *No* ☐ *Unsure*
55	In the past 6 months, have you used post-exposure prophylaxis (PEP) two times or more?<br>*Post-exposure prophylaxis is a medication that is taken very soon after a possible exposure to HIV to prevent becoming HIV-positive.*	☐ *Yes* ☐ *No* ☐ *Unsure*
56	In the past 6 months, have you been diagnosed with or treated for a sexually transmitted infection (STI)?<br>*Examples of sexually transmitted infections include chlamydia and gonorrhea.*	☐ *Yes* ☐ *No* ☐ *Unsure*
57	In the past 6 months, have you had sex while under the influence of drugs or alcohol?	☐ *Yes* ☐ *No* ☐ *Unsure*
58	In the past 6 months, have you been forced to have sex against your will or physically assaulted, including assault by your sex partner?	☐ *Yes* ☐ *No* ☐ *Unsure*
59	In the past 6 months, have you had sex with someone in exchange for money or a gift?	☐ *Yes* ☐ *No* ☐ *Unsure*
60	In the past 6 months, have you given someone money or a gift in exchange for sex?	☐ *Yes* ☐ *No* ☐ *Unsure*
61	In the past 6 months, have you shared needles with anyone while engaging in intravenous drug use?	☐ *Yes* ☐ *No* ☐ *Unsure*

The questionnaire ends here. Thank you for your responses.

For peer review only

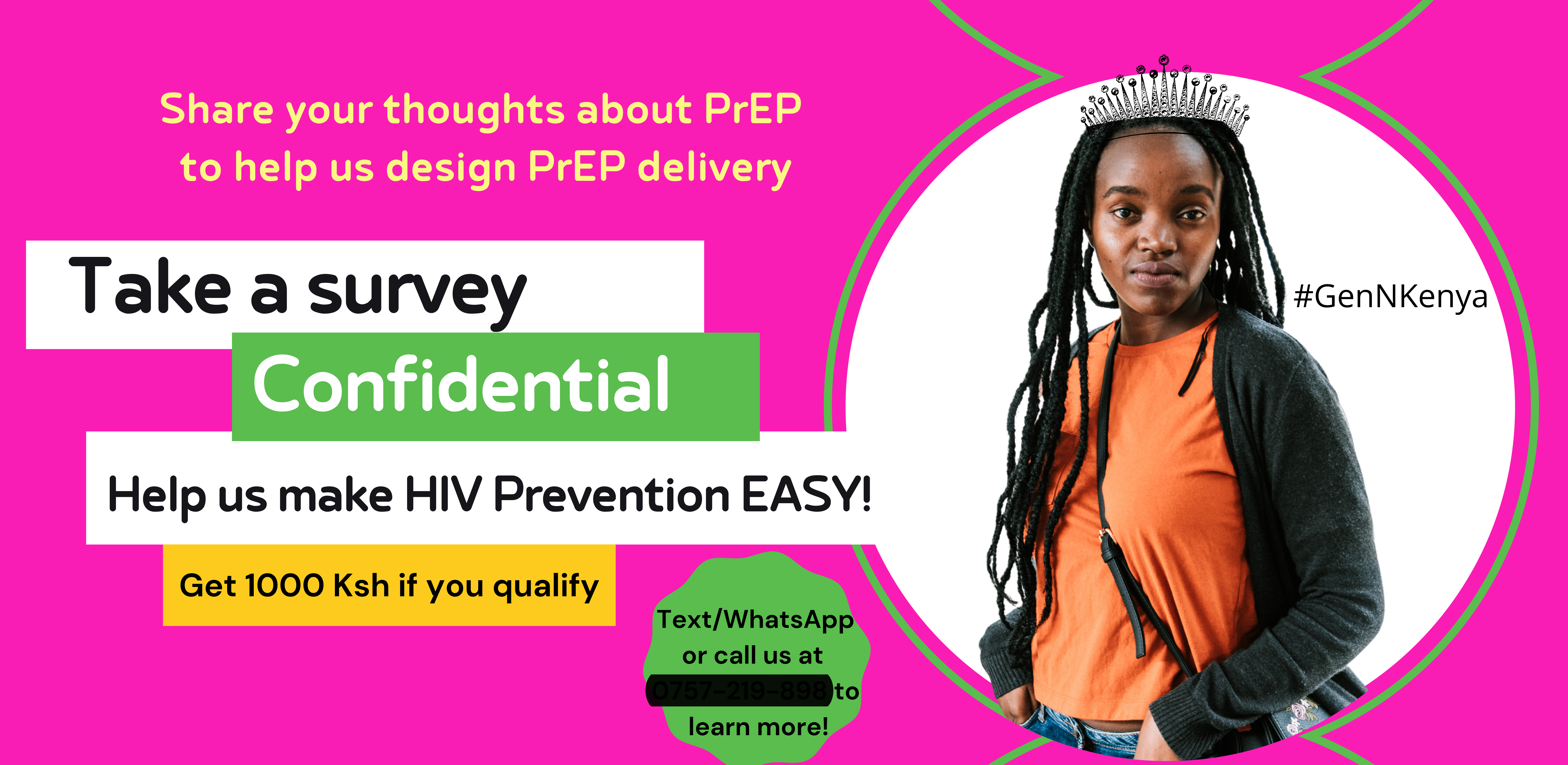
Share your thoughts about PrEP
to help us design PrEP delivery
Take a survey
Confidential
Help us make HIV Prevention EASY!
Get 1000 Ksh if you qualify
Text/WhatsApp
or call us at
[redacted] to
learn more!
#GenNKenya

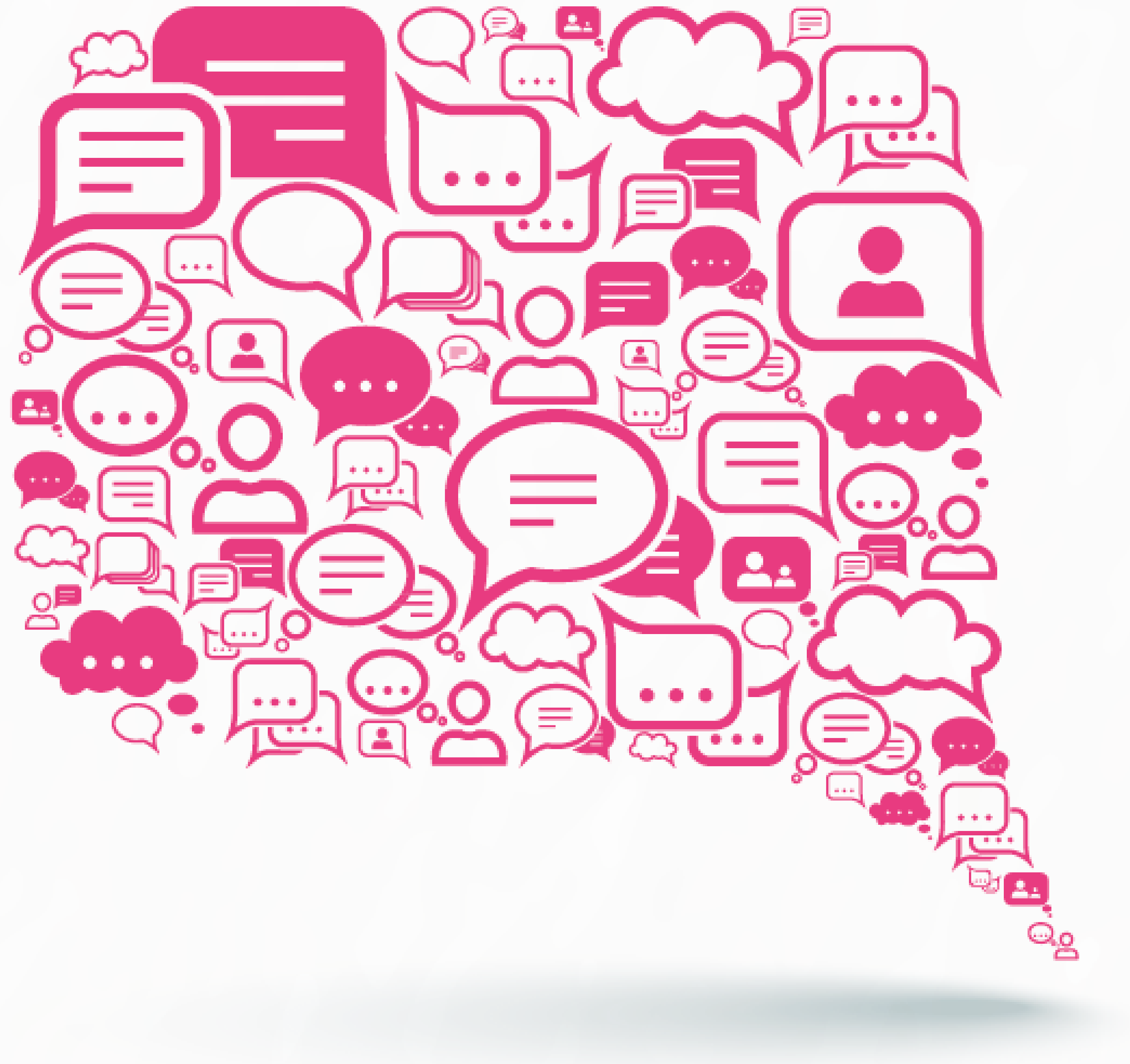

We are doing a survey about **PrEP service delivery.**
PrEP is a pill that you can take daily to reduce your risk of getting HIV.

We would like to understand what you think about getting PrEP delivered to you from an online pharmacy, like **MYDAWA.**

Hearing about your **preferences** will help us design new models of **PrEP delivery** for your community.

If you are interested, we will meet you in a **location of your choice** to complete the survey. The survey is confidential and takes about **45 minutes.** You will **receive 1,000 Ksh** for your time.

If you are interested in taking part in the survey or have any questions, please send a message via **text/WhatsApp or call: 0757-219-898**

Supplement 5: DCE Pre-screening Script

DCE RECRUITMENT SCRIPT

Hello, my name is…I'm a Research Assistant at KEMRI and we are doing a study to learn about what people in your community think about pre-exposure or PrEP. PrEP is a pill that you can take daily to reduce your risk of getting HIV.

We would like to learn about your preferences for getting them delivered to you from an online pharmacy. This means a pharmacy will deliver drugs to clients using a courier, so they do not need to travel to a pharmacy. Hearing about your preferences will help us design new models of PrEP delivery for your community.

If you are interested, we will meet you in a location of your choice to complete the survey. The survey is confidential and takes about 60 minutes. You will receive Ksh 1000 for your time and effort.

Are you interested in taking part in this survey?

[IF NO] Thank you very much and have a good [morning/afternoon evening]

[IF YES] That's great! I'm now going to ask you questions to make sure that you are eligible to be in this study. This will take about 10 minutes. If you are eligible, I'll schedule a time for someone from our research team to come to a place that is convenient for you, so you can complete the survey.

Before we begin, do you have any questions?

[Verifying Source of information] Thank you, tell me how you got to know about us?

[If they do not know, advise them on how they can access the study information on the MyDawa website]

[If the participant knows about PrEP study, go ahead and start the pre-screening process]

[Pre-screening process]

[What is your age?] If below the age of 18, inform the participant they are ineligible and explain the reasons why.

[What was the result of your last HIV test?] If positive, inform the participant they are ineligible for the survey.

Are you currently taking PrEP to prevent HIV?

[If eligible for the study participation. Please continue with scheduling their study visit]
Thank you for responding to the questions. You are eligible to participate in the study. I would like to ask you for some information on how I can contact you and when and where we can schedule the study.

For peer review only

[Write down the first name and surname]

[Write done the phone number where we can reach them and how they would prefer to be contacted] Through a phone call, SMS, or WhatsApp.

[Which day of the week are you available for the interview] Write down the day given by the participant.

[Time of scheduled study visit] Someone from our research team will come to a location of your choice to administer the study survey. Where would you like to meet?

[Enter study visit] Inform the study participant of the need for privacy, security, and confidentiality.

Thank you so much for your time and for agreeing to participate. Someone from our research team will contact you on the day of the survey for final arrangements. If you have any questions before the day of the interview you can reach the study through our contact number.

For peer review only

Supplement 6: DCE Data Management R Codes

```
---
title: "DCE Data Check - R Script"
date: "`r Sys.Date()`"
output:
  html_document:
    toc: true
    toc_float: true
---

# Load dataset
rm(list=ls())

# Set up R environment
## install.packages("stringr", "tidyverse", "dplyr", "pander")
library(stringr)
library(tidyverse)
library(dplyr)
library(pander)

# Set up functions
`%notin%` <- Negate(`%in%`)
table_na <- function(x){table(x, useNA = "always")}

# **NOTE**: Change my_path to your own directory
## my_path = "C:/Users/Yilin Chen/Desktop/2021 DCE ePharm/Data collection"
my_path = "~/Desktop/My projects/2021 DCE ePharm/Data collection" # Change this to the
path of your folder
setwd(my_path)

# Load data
## **NOTE**: Please change the dataset name accordingly
file_name = "GatesePharmDCESurvey_data_pre-launch_5.5.csv" # Change file_name to the
name of csv file
dat <- read.csv(file_name)
dat$x = seq(nrow(dat))

# Select relevant survey records in the data collection time window
dat$day <- str_split_fixed(dat$sys_StartTime, " ", 4)[,1]
dat$month <- str_split_fixed(dat$sys_StartTime, " ", 4)[,2]
dat$year <- str_split_fixed(dat$sys_StartTime, " ", 4)[,3]
dat$date <- as.Date(with(dat,paste(year,month,day,sep="-")),"%Y-%b-%d")
```

For peer review only

```
## **NOTE**: Please change the date_start and date_end below for specific data collection time window
date_start = "2022-03-09" # Change this to the start of time window
date_end = "2022-04-26"   # Change this to the end of time window
dat2 <- dat %>% filter(date >= date_start & date <= date_end) # example: from 2022-03-09 to 2022-04-26

# Select relevant survey records that contain "230" in the PTID
## **NOTE**: Please revise "230" to the starting number/word used in the actual survey
num_start = "230" # Change this to the actual starting number/word
dat2 <- dat2[str_detect(dat2$PTID1, num_start), ]
dat2 <- dat2[complete.cases(dat2[ , c('PTID1')]), ]
nrow(dat2)

# Data checks
## 1. Check for number of interviews

#####################################
# 1. Check for number of interviews #
#####################################

# Show PTIDs - first check if there are any missing PTIDs
unique(dat2$PTID1)

# Count the total number of interviews
length(unique(dat2$PTID1))

# Count the total number of interviews by RAs
dat2 %>% group_by(RAInitials) %>% tally() %>% pander()

## 2. Check for duplicate PTIDs and incomplete surveys

#####################################################
# 2. Check for duplicate PTIDs and incomplete surveys #
#####################################################

# Lists all duplicate records using the variable PTID1 and print out these PTIDs
count_pid <- as.data.frame(table(dat2$PTID1))
dup_pid <- as.character(count_pid$Var1[(count_pid$Freq > 1)])
dup_pid

# Create a subset with duplicate PTIDs
dat3a <- dat2 %>% filter(PTID1 %in% dup_pid) %>% select(sys_RespNum, sys_StartTime, sys_EndTime, sys_LastQuestion, RAInitials, PTID1, PTID2)
```

For peer review only

```
dat3a <- dat3a[order(dat3a$PTID1), ]

# Incomplete surveys: show PTIDs when the last answered question is not
"TeminateQuestionnaire" or "TerminateEligibility"
incomplete_id <- dat2 %>%
  filter(sys_LastQuestion %notin% c("TerminateQuestionnaire", "TerminateEligibility")) %>%
  select(x)

# List PTIDs of incomplete surveys
dat3 <- dat2 %>% filter(x %in% incomplete_id$x)
incomplete_pid <- unique(dat3$PTID1)
incomplete_pid

# Create a subset with incomplete surveys
dat3b <- dat3 %>% select(sys_RespNum, sys_StartTime, sys_EndTime, sys_LastQuestion,
RAInitials, PTID1, PTID2)
dat3b <- dat3b[order(dat3b$PTID1), ]

# Combine incomplete records and duplicate records
dat3c <- dat3a %>% full_join(dat3b, by = c("sys_RespNum", "sys_StartTime", "sys_EndTime",
"sys_LastQuestion", "RAInitials", "PTID1", "PTID2"))
dat3c$status = ifelse(dat3c$sys_LastQuestion %notin% c("TerminateQuestionnaire",
"TerminateEligibility"), "incomplete", "complete")
dat3c

# Save duplicate surveys to a csv file and add a date to the file name
## Most of duplicate surveys might be due to incompleteness
## **NOTE**: Change the date_now below to the current date
date_now = "05.12.2022" # Change to the current date
write.csv(dat3c, paste0("GatesePharmDCESurvey_duplicate_", date_now, ".csv"))

# You could also review these duplicate records on Sawtooth or original csv file, and figure out
reasons for duplicates
## Admin site link: https://ePharmacyPrEP.sawtoothsoftware.com/english/admin.html


## 3. Check for specific responses
### Part 1: Eligibility Assessment

#####################################
# 3. Check for specific responses #
#####################################

# Create a subset of eligible participants and interested variables for exploratory analysis
```

For peer review only

```
dat4 <- dat2 %>% filter(sys_LastQuestion == "TerminateQuestionnaire")
length(unique(dat4$PTID1)) # check number of unique participants
dat5 <- dat4[, 19:156] # check if this includes all variables of interest (starting from "RA initials"
variable)

# Part 1: Eligibility Assessment (Q1-4)
apply(dat5[, 4:8], 2, table_na)

## AgeYears: should be >= 18 years old
dat5$PTID1[dat5$AgeYears < 18]
## HIVTestDate_Month: should not be NA and should be numeric from 1-12
dat5$PTID1[is.na(dat5$HIVTestDate_Month)]
## HIVTestDate_Year: should not be NA and should be year
dat5$PTID1[is.na(dat5$HIVTestDate_Year)]
## HIVTestResult: should not include 2 - "Positive"
dat5$PTID1[dat5$HIVTestResult == 2]

### Part 2: PrEP Knowledge and Interest

# Part 2: PrEP Knowledge and Interest (Q5-7)
apply(dat5[, 9:14], 2, table_na)

### Part 3: Discrete Choice Experiment

# Part 3: Discrete Choice Experiment
apply(dat5[, 15:30], 2, table_na) # shouldn't have any NAs

### Part 6: E-Pharmacy Engagement and HIV Self-Testing

# Part 6: E-Pharmacy Engagement and HIV Self-Testing (Q8-29)
## e-Pharmacy engagement
apply(dat5[, 31:53], 2, table_na)

## ePharmEngDetail_7: if "Other" category = 1,  there should be text answers
table(dat5$ePharmEngDetail_7)
## PreferObtainPrEP_5_other: if PreferObtainPrEP == 5, there should be text answers
table(dat5$PreferObtainPrEP_5_other[dat5$PreferObtainPrEP == 5])
## AddlProducts_7: if "Other" category = 1,  there should be text answers
table(dat5$AddlProducts_7)
## PrEPRefillPref_4_other: if PrEPRefillPref == 4, there should be text answers
table(dat5$PrEPRefillPref_4_other[dat5$PrEPRefillPref == 4])

## WTP questions
apply(dat5[, 54:60], 2, table_na)
```

For peer review only

```
## Preferences for online PrEP delivery
apply(dat5[, 61:84], 2, table_na)

### Part 7: Participant Demographics

# Part 7: Participant Demographics (Q30-41)
apply(dat5[, 85:112], 2, table_na)

## FamPlanMethod_14_other: if FamPlanMethod_14 == 1, there should be text answers
table(dat5$FamPlanMethod_14_other[dat5$FamPlanMethod_14 == 1])
## AccessFamPlan_6_other: if AccessFamPlan == 6, there should be text answers
table(dat5$AccessFamPlan_6_other[dat5$AccessFamPlan == 6])

### Part 8: Sexual Behaviors

# Part 8: Sexual Behaviors (Q42-61)
apply(dat5[, 113:138], 2, table_na)

## SexPartType3Mo_4_other: if SexPartType3Mo == 4, there should be text answers
table(dat5$SexPartType3Mo_4_other[dat5$SexPartType3Mo == 4])
```

For peer review only
